# Streamlining the automated discovery of porous organic cages†

**DOI:** 10.1039/d3sc06133g

**Published:** 2024-03-13

**Authors:** Annabel R. Basford, Steven K. Bennett, Muye Xiao, Lukas Turcani, Jasmine Allen, Kim. E. Jelfs, Rebecca L. Greenaway

**Affiliations:** a Department of Chemistry, Molecular Sciences Research Hub, Imperial College London White City Campus, 82 Wood Lane W12 0BZ UK k.jelfs@imperial.ac.uk r.greenaway@imperial.ac.uk

## Abstract

Self-assembly through dynamic covalent chemistry (DCC) can yield a range of multi-component organic assemblies. The reversibility and dynamic nature of DCC has made prediction of reaction outcome particularly difficult and thus slows the discovery rate of new organic materials. In addition, traditional experimental processes are time-consuming and often rely on serendipity. Here, we present a streamlined hybrid workflow that combines automated high-throughput experimentation, automated data analysis, and computational modelling, to accelerate the discovery process of one particular subclass of molecular organic materials, porous organic cages. We demonstrate how the design and implementation of this workflow aids in the identification of organic cages with desirable properties. The curation of a precursor library of 55 tri- and di-topic aldehyde and amine precursors enabled the experimental screening of 366 imine condensation reactions experimentally, and 1464 hypothetical organic cage outcomes to be computationally modelled. From the screen, 225 cages were identified experimentally using mass spectrometry, 54 of which were cleanly formed as a single topology as determined by both turbidity measurements and ^1^H NMR spectroscopy. Integration of these characterisation methods into a fully automated Python pipeline, named *cagey*, led to over a 350-fold decrease in the time required for data analysis. This work highlights the advantages of combining automated synthesis, characterisation, and analysis, for large-scale data curation towards an accessible data-driven materials discovery approach.

## Abstract 2

Efficiently merging low-cost high-throughput screening and characterisation, automated data analysis, computational modelling, and *cagey* – a custom database analysis tool, enhances large-scale data curation and accelerates discovery of organic cages.

## Graphical abstract

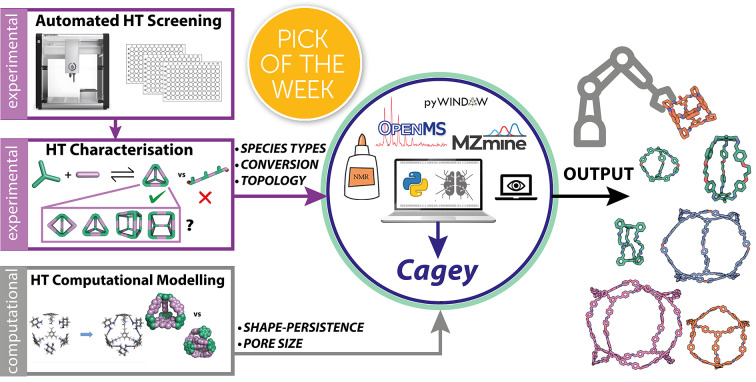

## Introduction

**Fig. 1:**
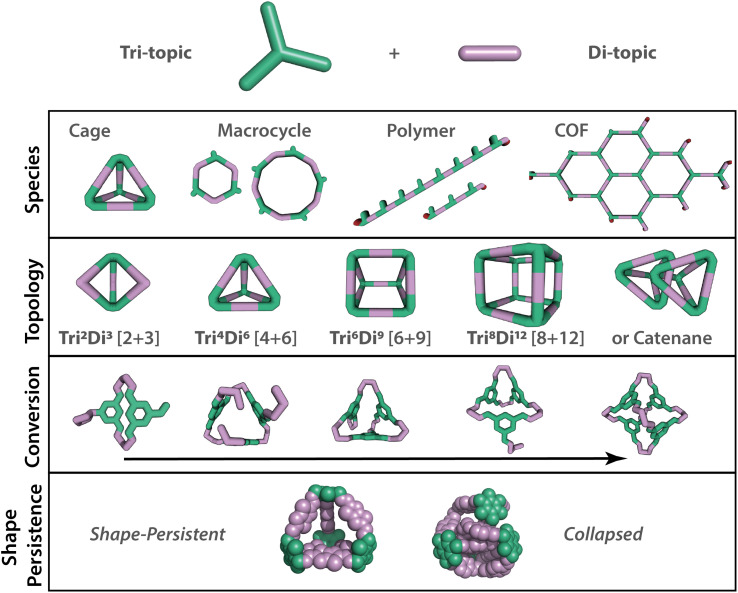
Range of possible outcomes that may occur from relatively simple building blocks, such as tri- and di-topic precursor combinations, in dynamic covalent reactions. When targeting an organic cage, a range of other supramolecular species may also be accessible. Even if an organic cage forms, further complexity arises in the range of potential topologies that may form, depicted by the notation Tri^*n*^Di^*m*^ or [*n* + *m*], where *n* = number of tri-topic building blocks and *m* = number of di-topic building blocks in the cage structure. Additionally, conversion in dynamic systems can vary, with the question being will the building blocks assemble fully, converting to a single targeted organic cage, or will smaller partially assembled cage oligomers be formed. The final target cage also needs to have the desired properties, such as shape-persistency, which is key in porous organic cages.

There has been a recent revolution in high-throughput materials synthesis using automation, enabling materials to be screened and discovered at a faster rate.^1^ The acceleration of materials discovery will play a crucial role in addressing the global problems of today, particularly in relation to molecular separations, which account for 10–15% of the world's energy consumption.^2^ Porous materials have emerged as a favourable option to address such challenges, by creating systems with void spaces or pores that can be readily tailored for specific separations. Porous materials, including metal–organic frameworks (MOFs), covalent–organic frameworks (COFs), porous polymers, and zeolites, have many applications as porous solids, with surface areas reaching as high as 7140 m^2^ g^−1^ for the MOF NU-110E.^3^ Another sub-class of porous materials are porous organic cages (POCs) – discrete molecules with permanent internal cavities accessible through windows, which may pack in the solid-state to form extended porous structures, but also typically exhibit solution processibility in common organic solvents.^4^ These advantageous features have gained POCs traction in the literature for their competitive porosities and guest selectivities, along with attractive applications in catalysis, chemical sensing, in thin-film membranes for gas and molecular separations, and as porous liquids.^5–8^ Despite this growing interest in POCs, the targeted design and realisation of new species has been a key challenge in the field, exacerbated by the: (i) complexity of the species types that can form (*i.e.*, thermodynamic *vs.* kinetic products, polymeric *vs.* discrete molecules, different cage topologies, Fig. 1); (ii) differing structural and thermodynamic stability of the resulting species (for example, subsequent cage catenation can occur after cage formation); (iii) packing and potential polymorphism of the individual species; and (iv) the sensitivity of the properties to all of the above.

POCs, and organic cages more broadly, are formed from the assembly of precursor building blocks of complimentary functionalities, typically through dynamic covalent chemistries (DCC), and in particular, imine condensations have been popular.^9^ DCC involves the reversible formation of covalent bonds in a dynamic equilibrium, combining the advantages of the reversibility typically associated with supramolecular assembly with the robustness of covalent bonding, *i.e.*, the building blocks may combine and then exchange in an ‘error-correcting’ manner, until the most thermodynamically stable product is formed.^10^ Due to the robustness of these imine condensations, Greenaway *et al.* were able to translate the conventional batch synthesis of organic cages into an automated high-throughput screening (HTS) workflow to drastically accelerate their synthesis, albeit using expensive Chemspeed platforms.^11^ HTS facilitated the exploration of a reasonably broad synthetic space through the parallelisation of 78 precursor combinations, resulting in the identification of 32 new species. However, bottlenecks remained in the experimental HTS workflow, including manual sample preparation for characterisation, and the analysis of the resulting large experimental datasets which were still conducted manually. In addition, HTS approaches can be inelegant and inefficient if applied blindly, with discoveries still reliant on small iterative changes or serendipity, so here our approach was combined with computational modelling to explore the potential advantages of a hybrid workflow.

Computational modelling has previously been used to provide *a priori* prediction and *a posteriori* rationalisation for experimental studies of POCs.^12^ High-throughput (HT) computational workflows can rapidly assemble precursor building blocks into cages of commonly observed topologies and assess both their shape-persistence (*i.e.*, a target POC should have a permanent internal cavity which is not lost on the removal of solvent) and properties prior to synthesis.^13^ For example, Turcani *et al.* previously created the current largest computational database of >60 000 POC structures, with model assembly automated using the open-source Python package *supramolecular toolkit* (*stk*) and property calculation using the open-source Python package *pyWindow*, giving predictions on pore diameter, volume, and the number and size of cage windows.^14,15^ Automated assembly and prediction of cage properties can yield information on trends and the influence of precursor design on POC properties. However, the transition from computational prediction to experimental realisation is a common hurdle in the discovery of novel cages with desirable predicted properties. In an attempt to overcome this, Bennett *et al.* developed a computational HT workflow which classified 12 553 molecules as either ‘easy’ or ‘difficult’ to synthesise by expert chemists to then train a machine learning (ML) model (*MPScore*) to make this classification.^16^ This was incorporated into a computational HT screening workflow to screen for POCs formed from synthetically favourable precursor building blocks, and which possessed desirable predicted cage properties.^16^

Despite the ability to predict the properties of hypothetical POCs using an automated computational approach, difficulty still arises in predicting the type and/or topology of species that will be formed in a DCC reaction, as introduced in the challenges above. Due to the complexity of the dynamic system within a DCC reaction, a range of possible species may form. The species formed may be molecular in nature, such as the desired POC, or a kinetically trapped side product - these side products are frequently oligomeric or polymeric in nature and therefore precipitate out of the dynamic system, no longer benefitting from the error-correction mechanism. Even if a molecular cage species does form, Santolini *et al.* enumerated 20 probable topologies that POCs may form, further highlighting the difficulty in predicting and targeting a specific reaction outcome.^17^ In that work, density functional theory (DFT) was used to calculate bond formation energies for cages of different topologies to post-rationalise the experimental formation of a mixture of topologies. Preferential formation of a single cage topology is likely attributed to it having the lowest formation energy, making it the most thermodynamically favoured, whereas, if a mixture of topologies is observed, it may be attributed to multiple cage topologies having a similar formation energy and thus no strong thermodynamic preference towards one topology is found. However, this approach does not guarantee successful cage formation, and does not consider experimental factors such as the solvent, overall concentration, reaction temperature or reaction time, all of which can affect the reaction outcome and lead to the formation of different species in DCC reactions.^18^ Data-led approaches combining experimental HTS and machine learning could rapidly accelerate this discovery process, but first, to do this, a HT workflow is needed for streamlined data collection and interpretation, ensuring a robust and reliable dataset is curated.

Here, we present a streamlined, hybrid, automated workflow for the accelerated discovery of organic cages and POCs, incorporating a low-cost automated liquid handling platform for both reaction and sample preparation, and open-source scripts for data analysis and computational modelling. This transferable approach enabled a precursor library of 55 multitopic amines and aldehydes to be screened across 366 imine condensations, consisting of both commercially available and ‘easy-to-synthesise’ precursors, some of which were selected from our previous ML approach to assess synthetically accessible POCs. Three HT characterisation techniques were employed to classify the reaction outcomes, based upon a need to determine the: (i) type of species formed; (ii) reaction conversion; and (iii) topological outcome. To streamline the overall workflow and remove human bias, an automated analysis procedure was developed and carried out, including the use of computer vision for determining the formation of insoluble precipitate, and the use of Python software to automate the analysis of both ^1^H NMR and mass spectra, to yield the reaction outcomes in a machine-readable format. HT computational predictions were incorporated into the workflow to predict cage properties, and in combination with the automated reaction outcome assessment, to narrow down the range of successfully formed organic cages and to help identify precursor combinations that had formed shape-persistent POCs.

## Results & discussion

### Building block selection

**Fig. 2:**
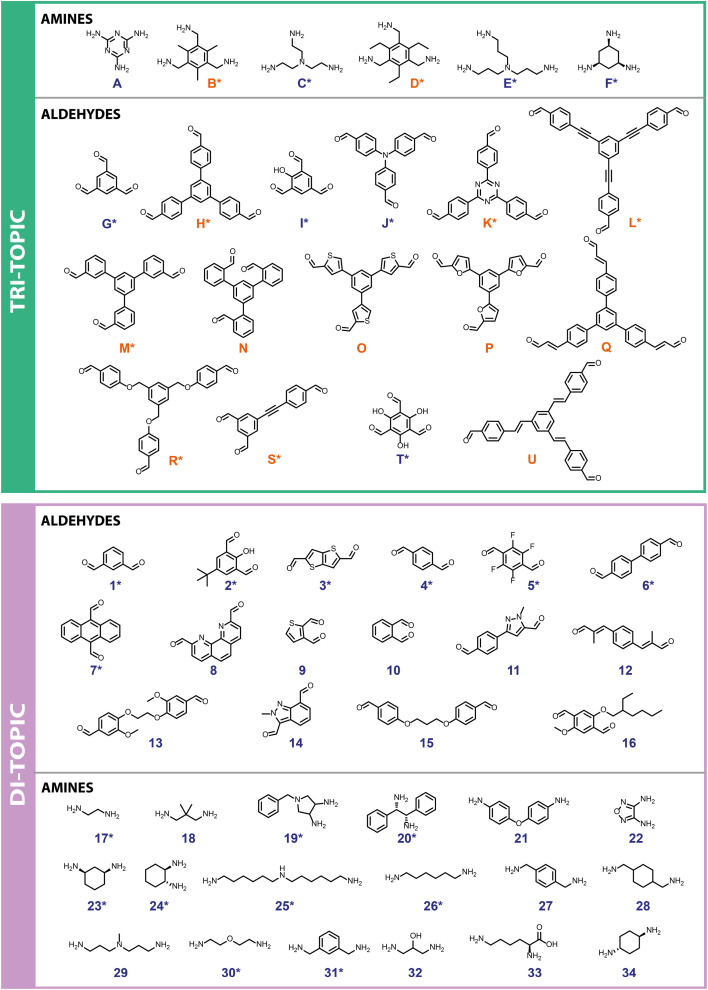
Precursor library containing 55 molecules: tri-topic precursors of both amines and aldehydes (top) were screened against di-topic precursors of aldehydes and amines (bottom), respectively, leading to 366 imine condensation reactions. Precursors were synthesised (orange) or commercially available (blue). If a precursor has previously been reported in the literature as a building block for POC formation it is marked with an asterisk (*).

First, a precursor library (Fig. 2) was assembled, with a focus on imine condensations and the **Tri**^***x***^**Di**^***y***^ family of cage topologies, where **Tri** represents tritopic precursors, **Di** represents ditopic precursors, and the superscript ***x*** and ***y*** represent the number of each precursor type incorporated into the final cage species. A range of aldehydes and amines were selected, which included a combination of commercially available building blocks and synthetically simple and accessible molecules, some of which were previously identified as potentially promising POC precursors that were classified as ‘easy-to-synthesise’ using our *MPScore*. Although our previous computational ML work on synthetically accessible POCs focused only on identifying trialdehydes and diamines for cage synthesis, we expanded the library to also include triamines and dialdehydes to increase the diversity of the precursor library.^16^ In addition, since precursor design, such as sterics, electronics, flexibility, and topicity (*i.e.*, the number of functional groups on a precursor), can influence the topological outcome of a reaction or the shape-persistence of the resulting cage, the precursor library was selected to ensure molecular diversity, featuring molecules of varying ring sizes, chain lengths, heteroatoms, and functionalities, to explore how precursor-level structural effects may influence the reaction outcome. Precursors that were either previously reported (asterisked, Fig. 2) in organic cage synthesis, and known combinations of these, were also selected to validate the workflow, and unreported precursors and combinations were selected to aid novel cage discovery. For example, the use of vicinal diamines as precursors, such as **17**, **20** and **24**, is common in the literature and they have formed a range of POCs, including the **Tr**i^**4**^**Di**^**6**^ POC **CC3**, synthesised from the 12-fold imine condensation between 1,3,5-triformylbenzene (**G**) and (1*R*,2*R*)-cyclohexane-1,2-diamine (**24**).^19^ In total, 31 precursors from our library have previously been reported to form a POC, and 24 precursors are unreported in the literature. In this way, we minimise dataset bias. Overall, this led to 15 tritopic aldehydes which were combined with 18 ditopic amines, and 6 tritopic amines combined with 16 ditopic aldehydes, totalling 366 imine condensation reactions.

### Experimental high-throughput workflow

**Fig. 3:**
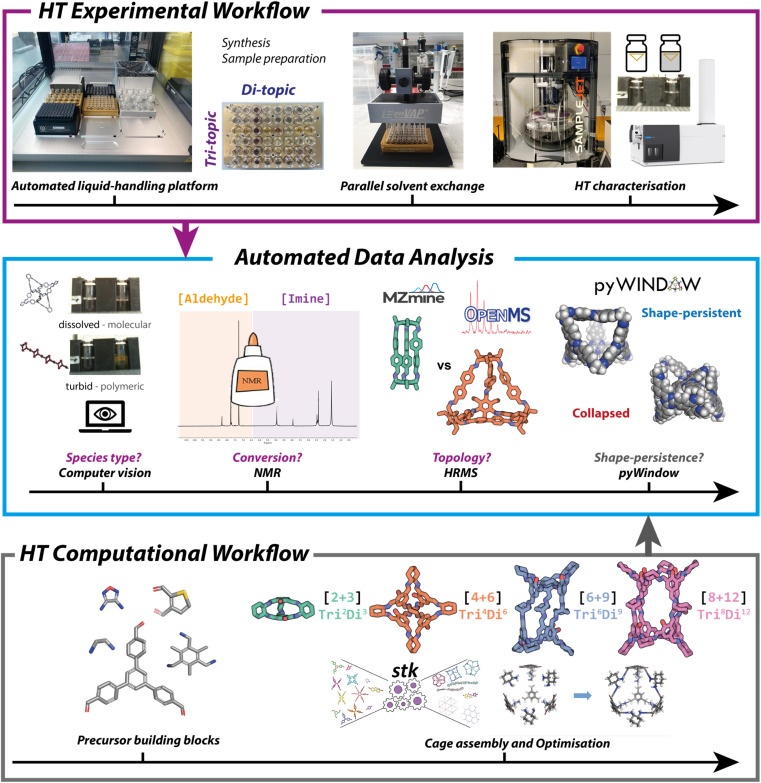
(Top) High-throughput experimental workflow, including the setup on the automated liquid-handling OT-2 platform. Rapid parallel solvent evaporation was achieved using an EquaVAP, and the sample preparation for characterisation was undertaken on the OT-2 prior to HT measurements being carried out. (Middle) High-throughput experimental data (turbidity, ^1^H NMR and high-resolution mass spectrometry) was automatically analysed to assign the reaction outcomes of the 366 imine condensations by species type, conversion, topological outcome, and in combination with the HT computational data (pore size analysis) led to the range of successfully formed organic cages being narrowed down further to identify shape-persistent POCs. (Bottom) High-throughput computational workflow for the optimisation and loading of each precursor building block, followed by the assembly into organic cages of the four most common topologies ([2 + 3] **Tri**^**2**^**Di**^**3**^, [4 + 6] **Tri**^**4**^**Di**^**6**^, [6 + 9] **Tri**^**6**^**Di**^**9**^, [8 + 12] **Tri**^**8**^**Di**^**12**^), and an optimisation process giving the lowest energy conformer.

To accelerate HT experimental screening, an Opentrons liquid handling platform (OT-2) was employed (Fig. 3, top), and the reactions miniaturised. The OT-2 deck consists of standard micro-titre plate (MTP) positions and is traditionally used in aqueous-based biological settings but was selected here as a low-cost and open-source accessible solution that was adapted to tolerate harsher chemicals. Previously, HT organic cage synthesis has only been reported using high-cost robotic platforms, arguably limiting the uptake of automation as a tool in materials discovery. The OT-2, in contrast, offers a low-cost suitable alternative. Protocols for both HT synthesis and sample preparation for characterisation were written in Python using the Opentrons open-source Python package and application programming interface (API). A general Python input script was written that included adjusted aspirate, dispense and gantry speeds, along with functions to pre-wet and swell the pipette tips and lower the maximum volumes of the pipettes – this was required to prevent damage to the pipette, uncontrolled dripping of higher density or more volatile solvents across the platform deck into untargeted wells, and most importantly, to ensure accurate and precise dispenses of the non-aqueous solvents and precursor stock solutions (see Table S2 and Fig. S7† for further details and solvent calibrations). This script then calls upon dictionaries containing precursor stock locations and volumes with their corresponding transfer steps for each plate.

Overall, 366 precursor combinations were screened using the same reaction conditions (0.0092 M, 1 mL total volume, CHCl_3_, RT, 5 days) in 48-well plates. These reaction conditions were first scaled-down from a previous high-throughput workflow,^11^ and expanded to include both classes of tri-topic and di-topic combinations for amines and aldehydes. To ensure applicability of the conditions across both combinations, the concentration, precursor ratios, and reaction temperature were screened for two representative and previously reported cage species (**B4** and **G24**), prior to carrying out the large scale high-throughput screen using the subsequently optimised set of reaction conditions (see ESI Section S4† for further information). Stock solutions (5–6 mg mL^−1^) of each precursor were transferred from 24-well plates (max. 8 mL volume) into the reaction vials (max. 2 mL volume) in the 48-well plates, before being made up to a total volume of 1 mL using the reaction solvent, ensuring the same overall concentration for each combination. Full experimental conditions are given in the Tables S3–S5.† In addition to the 366 precursor combinations, reactions were repeated across the separate runs in different plate positions to confirm the reproducibility of the workflow. On reaction completion, the turbidity was determined (see Automated analysis section), before an EquaVAP was used to remove the reaction solvent from all wells in parallel under a flow of nitrogen. The reaction well-plates were subsequently returned to the OT-2 deck to prepare the samples for characterisation. Both NMR and mass spectroscopy samples were prepared on the platform and dispensed directly into tubes and vials in either a custom 3D-printed 96-NMR tube rack or compatible 54-well plates for the mass spectrometer, respectively. The prepared samples were subsequently submitted for analysis, using a Bruker SampleJET autosampler allowing up to 5 × 96 samples to be queued for ^1^H NMR analysis, and direct injection on a high-resolution mass spectrometer (HRMS). Full details for each step of the automated high-throughput screen and data curation are outlined in the ESI† and available on GitHub (https://github.com/GreenawayLab/Streamlining-Automated-Discovery-POCs).

### Automated data analysis

To determine the overall reaction outcome, three experimental characterisation techniques were selected: turbidity, ^1^H NMR spectroscopy, and HRMS. These characterisation methods were chosen to each give different information about the reaction and were used in conjunction to best determine the reaction outcome. For example, the turbidity data gave an indication of the type of species formed, where any observed precipitation was assumed to suggest the presence of insoluble oligomeric or polymeric side-products – a homogenous reaction mixture was targeted through a low overall concentration to ensure promotion of the ‘error-correction’ mechanism of the DCC reaction, where full equilibration of the dynamic system can occur to the thermodynamic product or mixture in solution. Next, ^1^H NMR spectroscopy allowed us to assess the reaction conversion, and the use of HRMS identified if a cage formed, alongside which topology.

Having selected the key characterisation techniques, we addressed the largest bottleneck of the HT workflow – data processing and interpretation. In the initial HT workflow for organic cage screening reported by Greenaway *et al.*, this was completed manually, and was solely reliant on the speed of the researcher and susceptible to human bias. Therefore, automated analysis focused on reducing human reliance, increasing the rate of interpretation to further streamline the overall workflow, and formatting data into a machine-readable format.

### Computer vision turbidity species analysis

First, the turbidity of the reactions was screened using computer vision software adapted from the open-source Python package *Heinsight*^20^ which, broadly, uses the difference in brightness between the region of interest (ROI) of a reference, here the reaction solvent chloroform was used, and the measured sample in recorded images to determine whether a solution is turbid. Using the dissolved reference ROI measurement, a turbidity threshold was determined. If the sample's measured turbidity was below this threshold, it was characterized as ‘dissolved’ and therefore assumed to be a molecular species. Whereas if the measured sample's turbidity was above this threshold, it was characterized as ‘not dissolved’ and as such, it was assumed that an insoluble oligomeric, polymeric, or COF side-product had been formed. Each of the latter outcomes is generally undesirable for POC synthesis, removing species from equilibrium, reducing the formation and subsequent yield of any associated POC, and necessitate, at the very least, an additional filtration step to isolate the desired soluble cage species. Further extraction and processing of the data was conducted using an in-house Python script that categorised whether a measured sample was ‘dissolved’ or ‘not dissolved’ and if it matched researcher assessment. Typically, cases where the computer vision outcome did not match researcher observation were where small, clear crystals had formed on the side of the vials, above the selected ROI, which may alternatively be attributed to POC crystallisation. However, computer vision successfully categorised 96% of the reaction outcomes when compared to researcher assessment (Fig. S10†) and we deemed this level of accuracy to be sufficient for taking the method forward.

### ^1^H NMR conversion analysis

Next, due to the vast number of precursor combinations, we further developed an automated Python workflow for analysing the reaction outcome of each precursor combination. For assessing conversion based on aldehyde consumption, we automated the analysis of the ^1^H NMR spectra by writing an in-house Python script, adapted from the Python package *nmrglue*.^21^ This open-source library allows for processing, manipulating, and analysing NMR spectra directly within Python, facilitating HT analysis. First, the script was adapted to remove the chloroform solvent peak and corresponding satellites (selected as the analysis solvent), preventing false positives arising due to the misidentification of the solvent peak in the aromatic region, and a minimum relative peak intensity threshold was determined to prevent baseline peak-picking. While POCs are complex assemblies formed from multiple building blocks, due to their highly symmetrical nature, they typically exhibit only one or two imine ^1^H NMR signals. Therefore, the region between 6 and 9 ppm was analysed, a region typical of these imine signals in POCs, to assess whether a reaction had occurred. However, identification of the imine peaks can become unclear when non-symmetrical precursors are used, resulting in the formation of unsymmetrical species or a mixture of cages which can lead to splitting of the imine signals or the broadening of peaks. As this region is also typical of aromatic peaks, false positives may occur. Therefore, a secondary check was conducted to assess the reaction conversion – as an excess of amine was used in an attempt to drive the DCC reactions to completion, the assumption was made that the observation of any aldehyde signals between 9 and 11 ppm would indicate the presence of residual unreacted precursor, and therefore that the reaction had not gone to completion. Overall, this resulted in a 98% accuracy between the automated analysis compared to researcher manual assessment, validating the automated approach.

### HRMS topology analysis

Finally, automated HRMS peak detection and assignment was developed as part of the automated data analysis workflow to identify mass ions corresponding to the different potential POC species in the mass spectra. However, this was a non-trivial problem to solve since the main mass ion (*m*/*z*, 100%) for a doubly charged species is identical to that of a singly charged species of half the size (for example, a doubly charged **Tri**^**8**^**Di**^**12**^ species has the same calculated mass ion as a singly charged **Tri**^**4**^**Di**^**6**^ species). Therefore, to account for screening of multi-charged species, identification of the spacing between peaks in the isotopic distribution was also included. For example, detection of 0.5 *m*/*z* peak separation would be required to differentiate between a doubly charged species and a singly charged species. In addition, HRMS cannot differentiate between a **Tri**^**8**^**Di**^**12**^ species and a catenated **Tri**^**4**^**Di**^**6**^ species, both of which would have the same mass. Therefore, we opted to screen for the discrete building block assemblies that would constitute full reactivity of all functionalities, namely [2 + 3], [4 + 6], [6 + 9] and [8 + 12], over the specific **Tri**^**2**^**Di**^**3**^, **Tri**^**4**^**Di**^**6**^, **Tri**^**6**^**Di**^**9**^, and **Tri**^**8**^**Di**^**12**^ topologies. Overall, this meant that analysis of the HRMS data through file pre-processing and topological outcome assignment was the most challenging of the three experimental characterisation methods to automate.

The proprietary Agilent data format was initially converted to the open-source mzML format using the *MSConvert* tool of the ProteoWizard software suite.^22^ The mzML format is a standardised open data format designed to store raw unprocessed data generated by MS instruments to be read by open-source MS software for further processing. The first step of the HRMS workflow involved extracting mass spectra from the total ion count chromatogram. To achieve this, the open-source *mzMine* package was used to automate the extraction of *m*/*z* values into a computer-processable format.^23^ The package *mzMine* is designed for performing analysis on large datasets of raw mass spectra data. The process involves detecting the masses above a certain threshold, extracting the ion chromatograms for each mass ion peak, and using an in-built pipeline method to calculate the intensity of each *m*/*z* peak over time which is written to a text file. The extracted mass ion peaks were then compared with those expected for each of the four topologies for a given pair of building blocks, with the number of water molecules lost for each imine condensation subtracted and possible mass ions with common ESI adducts incorporated, using pyOpenMS within the Python workflow.^24^ Of the 366 precursor combinations, 5 false positives (where POC species were incorrectly identified) and 14 false negatives (where POC species were not identified but had formed) were observed using the automated HRMS analysis, giving an overall 95% accuracy. From the 19 reaction combinations where researcher manual analysis and automated analysis did not agree (Fig. S10 and Table S7†), 12 (63%) were due to the misidentification of mixtures of topologies – the automated analysis correctly identified one topology, but on manual inspection a mixture of two species was observed. These false positives and negatives were likely observed due to the presence of other relatively low intensity mass ions that had similar *m*/*z* values to the cage topology peaks. Despite this, an overall 95% accuracy remained sufficient for this methodology to be carried forward with confidence in the automated analysis workflow, especially given the time savings that this approach affords for further HT screens.

### Managing high-throughput data in a cage database

Finally, the Python pipeline *cagey* takes the individual automated analysis outcomes for each characterisation technique for each precursor combination (**A1** to **U34**), along with their associated experiment code for reference, into a single machine-readable dataframe. Full details for each step are outlined in the ESI† and the Python scripts are available on GitHub (https://github.com/GreenawayLab/cagey).

### High-throughput experimental screening results

**Fig. 4:**
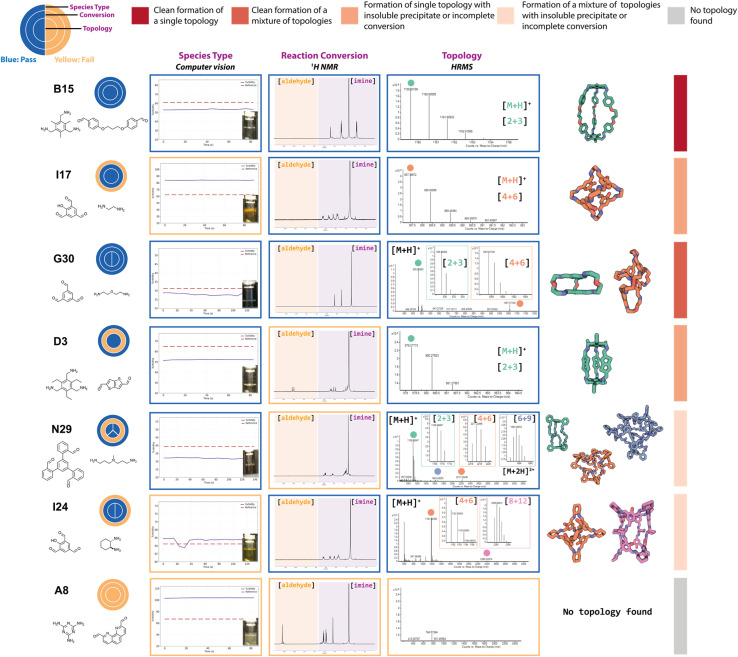
Examples of DCC reaction outcomes from the high-throughput screen targeting organic cages. The three circles are for the ‘pass’ (blue) or ‘fail’ (yellow) of each experimental analysis (computer vision turbidity analysis, ^1^H NMR spectroscopy, and high-resolution mass spectrometry (HRMS), to indicate the species type, conversion, and cage topology, respectively). The splitting of the inner ring indicates the number of cage topologies identified by HRMS. The observed experimental data for each characterisation method is shown in the boxes coloured by their characterisation pass/fail check. Each reaction outcome was categorised as follows (identified by a coloured bar on right-hand side): single topology observed to form cleanly (dark red) or incompletely (orange); mixture of topologies observed to form cleanly (red) or incompletely (coral); and no topology observed (grey). The turbidity reference is shown as a red dashed line and the observed turbidity as a purple line, if the line is below the reference, the sample passes and is in solution, if it is above the line then it fails and there is presence of insoluble precipitate. The ^1^H NMR orange boxes over the spectra indicate the region between 9 and 11 ppm which is searched for residual aldehyde precursor and the purple box for the region searched for the presence of imine peaks. The HRMS spectra are shown to the right and the peak for an observed cage topology is indicated as a coloured dot and the spectra zoomed in accordingly to show the splitting – green for [2 + 3] **Tri**^**2**^**Di**^**3**^, orange for [4 + 6] **Tri**^**4**^**Di**^**6**^, blue for [6 + 9] **Tri**^**6**^**Di**^**9**^, and pink for [8 + 12] **Tri**^**8**^**Di**^**12**^ cage topologies. The predicted cage structures for the observed topologies are shown on the right with the carbons in the same colour – hydrogens are omitted for clarity.

The complexity of DCC and the different possible reaction outcomes is clearly shown by the diversity of results we see when targeting organic cage formation, highlighting the requirement for the use of all three experimental characterisation methods within the automated workflow to fully describe the reaction outcome. By taking into account the automated turbidity analysis of the reaction as a ‘pass’ (no precipitate) or ‘fail’ (precipitate), the automated ^1^H NMR analysis as a ‘pass’ only if the aldehyde was fully consumed and a species was present in the aromatic/imine region, and the automated HRMS analysis as a ‘pass’ if a mass ion of at least one targeted cage assembly was detected, it was possible to identify a range of different organic cages. Representative examples of this sequential categorisation for different combinations can be seen in Fig. 4.

In an ideal scenario, the reaction would contain no insoluble precipitate, would have gone to full conversion, and the presence of a mass ion peak corresponding to a single cage species would be apparent, based on the assumption that no catenated species were formed, indicating a clean hit, as found for **B15** (where cage names are given as the precursor combination from which they are formed). From a materials discovery perspective, this outcome is the most desirable, as it requires the least amount of post-processing to isolate the desired cage. Arguably, the next most desirable outcome is one with full conversion to a single cage species, but that fails the turbidity check, as after a simple filtration to remove the insoluble precipitate, the desired cage could be isolated, such as for **I17**. While some precursor combinations, such as **G30**, also pass all of the automated analysis steps in relation to no observed precipitate, full conversion, and identification of a mass ion corresponding to a cage species, in this scenario, a mixture of cages was identified in the HRMS (both [2 + 3] and [4 + 6] cage assemblies). While the formation of mixtures of cages has been previously reported in the literature,^11,18^ this is a less desirable outcome, as it is often difficult to isolate both species individually, especially when taking into account the dynamic nature of the imine bonds, typically requiring selective crystallisation or purification by preparative HPLC. Partial conversion to single cage species can also occur, such as in the case of **D3**, where the NMR check fails due to the presence of residual aldehyde, or even more complex mixtures identified by HRMS, such as **I23** (containing [4 + 6], [6 + 9] and [8 + 12] species) or **N29** (containing [2 + 3], [4 + 6] and [6 + 9] species). Reaction outcomes that contain mixtures can be desirable when accessing cages with larger, less common topologies, but there is a trade-off due to the requirement for additional purification steps to isolate the desired cage(s). Finally, precursor combinations may also result in outcomes where no cage formation is observed, such as in the case of **A8**, along with no suggestion of reactivity due to the failure of both the turbidity and NMR checks.

**Fig. 5:**
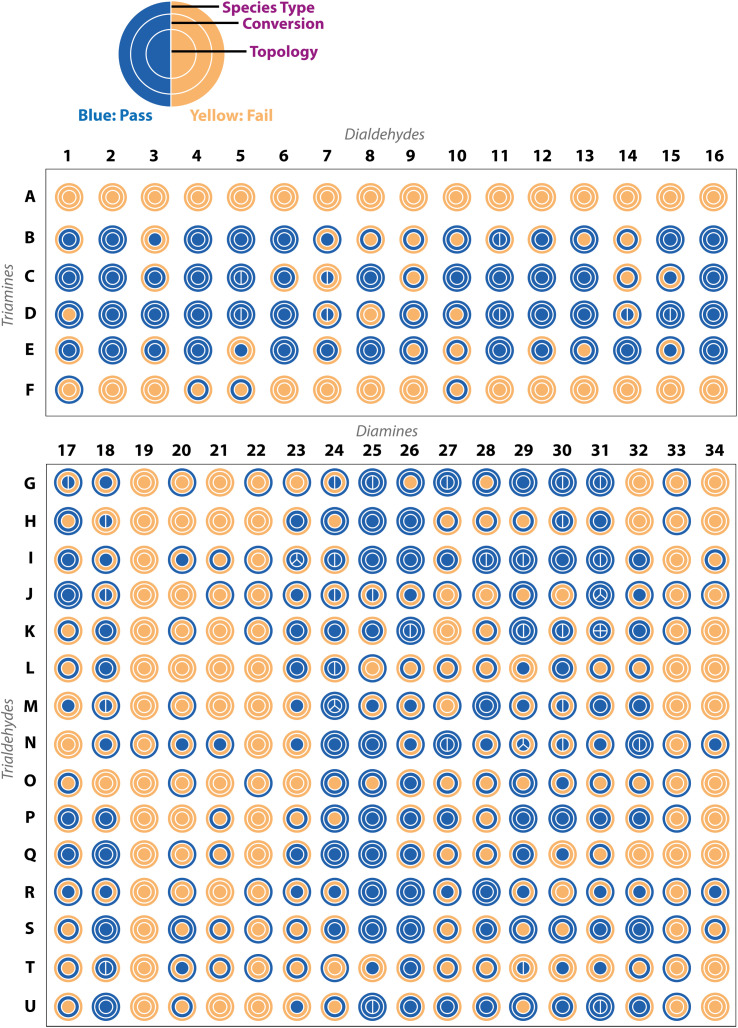
Experimental results for all 366 precursor combinations from the automated analysis, based on either ‘pass’ (blue) or ‘fail’ (yellow) of the computer vision turbidity analysis, ^1^H NMR spectroscopy, and high-resolution mass spectrometry (HRMS), to indicate the species type, conversion, and cage topology, respectively. The splitting of the inner ring indicates the number of cage topologies identified by HRMS.

Applying these ‘pass’/‘fail’ categorisations across all 366 precursor combinations (Fig. 5), some general trends can be observed about the potential of each precursor to form a cage. For example, no combinations with triamines **A** and **F**, and diamines **19**, **22**, and **33**, formed any identifiable cage species. In contrast, we can identify highly promising precursors whose combinations resulted in a large proportion of successful cage formation, as identified by HRMS, including dialdehydes **2**, **4** and **16** (66%, but passing all categorisations), diamines **25** (86%) and **26** (86%), triamine **C** (88%), triamine **D** (75%), triamine **E** (81%), trialdehyde **I** (72%), trialdehyde **N** (78%) and trialdehyde **R** (67%).

Precursor combinations that led to cage mixtures being identified are shown by the splitting of the inner ring by the number of observed topologies in the mass spectra (HRMS topology check, Fig. 5). The majority of the mixtures observed in the HT screen were of [2 + 3] (assumed to be **Tri**^**2**^**Di**^**3**^) and [4 + 6] (assumed to be **Tri**^**4**^**Di**^**6**^) species (78%), the two most commonly reported topologies in the literature.^17^ Of the 366 precursor combinations, four reactions resulted in the formation of a mixture of three cage topologies – **I23** ([4 + 6] **Tri**^**4**^**Di**^**6**^, [6 + 9] **Tri**^**6**^**Di**^**9**^ and [8 + 12] **Tri**^**8**^**Di**^**12**^), and **J31**, **N29** and **M24** ([2 + 3] **Tri**^**2**^**Di**^**3**^, [4 + 6] **Tri**^**4**^**Di**^**6**^ and [6 + 9] **Tri**^**6**^**Di**^**9**^) – and only a single combination, **K31**, resulted in all four screened cage topologies being identified ([2 + 3] **Tri**^**2**^**Di**^**3**^, [4 + 6] **Tri**^**4**^**Di**^**6**^, [6 + 9] **Tri**^**6**^**Di**^**9**^ and [8 + 12] **Tri**^**8**^**Di**^**12**^). Both **J31** and **M24** combinations resulted in the clean formation of mixtures with full conversion and no insoluble precipitate observed, whereas **N29** formed a mixture with no insoluble precipitate, but not full conversion, indicating the DCC reactions could potentially further equilibrate to one of the more thermodynamically favourable topologies.

**Fig. 6:**
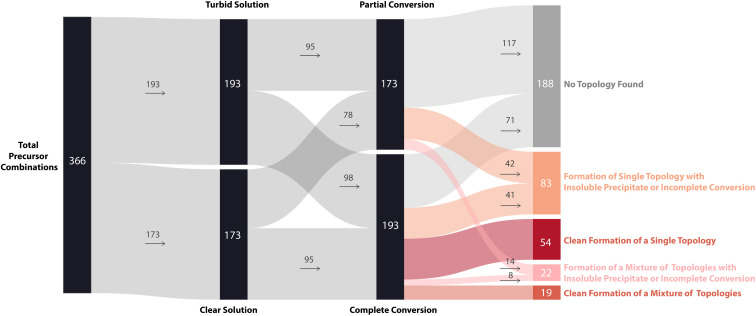
Sankey diagram illustrating the overall reaction outcomes of the high-throughput screen based on categorisation. Each reaction outcome was categorised as follows: single topology observed to form cleanly (dark red) or incompletely (orange); mixture of topologies observed to form cleanly (red) or incompletely (coral); and no topology observed (grey). The thickness of each connecting line is proportional to the number of precursor combinations leading to each outcome, and the numbers inside or next to each rectangle show the number of precursor combinations assigned to that outcome.

To further display the range of reaction outcomes of the 366 precursor combinations, a Sankey diagram was used (Fig. 6), with the proportion of outcomes for each precursor category (triamine, trialdehyde, diamine, and dialdehyde) shown in the ESI (Fig. S13†). In total, 54 precursor combinations (15%) resulted in the clean formation of a single cage topology, the most desired outcome (matching **B15**, Fig. 4). The largest proportion of outcomes (23%, 83 precursor combinations) resulted in the formation of a single topology, but alongside insoluble precipitate or incomplete conversion (including **I17** and **D3**, Fig. 4, respectively). Overall, the distribution of outcomes that resulted in a mixture was considerably less than formation of a single cage topology (clean or not), indicating that there is typically a thermodynamic energetically favoured topology that a DCC system will form. Of the HT screen, 19 (5%) resulted in the clean formation of a mixture of topologies (similar to **G30**, Fig. 4), and 22 (6%) resulted in the formation of a mixture of topologies with insoluble precipitate or incomplete conversion (mirroring **I23** and **N29**, Fig. 4, respectively). The highest proportion of outcomes, 88 combinations (24%), was where no cage topology was identified (analogous to **A8**, Fig. 4) – this includes precursor combinations where both species type and conversion checks may have been passed, but none of the screened cage assemblies were identified in the mass spectra.

Next, we grouped the precursors by both topicity and functional group to investigate how the reaction outcomes are distributed with respect to specific precursors to further identify reactivity trends (Fig. S13†). Generally, a precursor that frequently leads to successful cage formation indicates it is a promising candidate for cage synthesis, accommodating a greater diversity of precursor pairings. The six triamines **A–F** were each screened against dialdehydes **1–16**. Triamine **C** showed the greatest amount of reactivity towards cage formation, whereas both **A** and **F** resulted in no cage topology being observed across all combinations. This follows the lack of literature precedent for **A**, which has not been reported as a precursor building block in organic cages to the best of our knowledge. However, triamine **F** has previously been used as a precursor in combination with dialdehyde **2** by Skowronek and coworkers, resulting in the successful formation of an imine **Tri**^**8**^**Di**^**12**^ porous organic cage.^25^ In their study, the role of entropy of symmetry as a driving force for cage assembly and structural stability is emphasised. However, the reaction conditions used in this HT screen differ from the reported successful cage synthesis, both in relation to the reaction solvent, temperature, concentration, and reagent stoichiometry, indicating, as expected, that these variables play a contributing role in directing the outcome of the DCC reaction.

Trialdehydes **G–U** were each screened against 18 diamines, of which trialdehyde **S** was the best performing, with 6 (33%) combinations yielding the most desirable outcome of clean formation of a single topology. Trialdehydes **I**, **P**, **Q** and **R** also showed good reactivity, with trialdehydes **I** and **R** previously reported in organic cage formation,^26,27^ but **P** and **Q** being novel building blocks not previously reported as organic cage precursors. Trialdehyde **O** showed the least reactivity with 17 (94%) of its combinations with diamines affording no observed cage topology, followed by trialdehydes **L** and **T**, which saw no clean formation of either a single or mixture of topologies.

The dialdehydes **1–16** were only screened with the six triamines, with dialdehydes **2**, **4** and **16** yielding the best reactivity where 4 (67%) reactions led to the clean formation of a single topology, but with no topology consistently observed in combination with triamines **A** and **F**. However, the successful reactivity of these dialdehydes with the other triamines can be expected as they have all previously been reported in the literature as organic cage precursors, albeit not in the same precursor combinations (Fig. 2).^19,25,26,28^ Clean formation of a single topology was also observed for dialdehydes **8**, **11**, **12** and **13** (50%), whereas dialdehyde **9** exhibited no reactivity across all reaction combinations. Finally, diamines **17–34** were each screened across 15 reactions, with **19**, **20** and **33** showing no reactivity across any combinations, followed by **20**, **21** and **34** where ∼80% displayed no cage formation and the remaining ∼20% yielded the formation of a single topology with either insoluble precipitate or incomplete conversion. Diamine **18** was the most successful diamine building block, with 14 (93%) of its reactions indicating formation of an organic cage across the three categorisations. Following this, **25** and **26** showed similar success with 13 (87%) of their combinations indicating evidence of cage formation *via* HRMS, arguably suggesting that **25** was the most successful diamine overall with 7 (47%) of its combinations resulting in the clean formation of a single cage topology.

From the observed trends in the HT screen, we can identify building blocks that are generally poor candidates for cage synthesis and those that are more likely to result in cage formation (as discussed below), directing future synthesis. However, to try and obtain a fuller picture of the effect of sterics, flexibility, and topicity of the precursor building blocks on cage formation and cage properties, we first carried out computational modelling to determine the predicted structures and shape-persistency of the screened combinations, and therefore to identify any structure–reactivity and/or structure–property relationships.

### Computational modelling and automated analysis

The computational workflow is outlined in Fig. 2 (bottom), with full details given in Section S7.1 and S7.2 of the ESI.† The aim of the computational workflow was to provide computational models of the four most likely cage topologies (**Tri**^**2**^**Di**^**3**^, **Tri**^**4**^**Di**^**6**^, **Tri**^**6**^**Di**^**9**^ and **Tri**^**8**^**Di**^**12**^) that may form for each precursor combination, to then predict whether the structure will have favourable properties, such as shape-persistency. The workflow does not yet predict which cage topology is most likely to form but aims to complement the experimental high-throughput workflow by predicting properties of the experimentally identified cages.

The computational workflow includes the precursor input and quick force-field optimisation process. The optimised molecule should best represent the most common conformer of the precursor, and therefore the one most likely to react in the subsequent imine condensation. Following this, for each of the 366 combinations, precursors were assembled into cage structures, using the open-source supramolecular toolkit (*stk*) package,^14^ of the four screened cage topologies, totalling 1464 cages modelled. A three-step optimisation process with molecular dynamics was undertaken to find the lowest energy conformer of the cage. Following the cage construction and conformer generation procedure, we performed a HT property screen to predict which cages would remain shape-persistent and which would collapse, allowing us to identify cages that were experimentally observed and likely to possess intrinsic porosity. Here, we determined computationally whether a cage had a permanent internal cavity and accessible windows using *pyWindow*, a Python package which is able to analyse the internal pore and windows of discrete cavity-containing molecules.^15^ Incorporating *pyWindow* (Fig. 3, middle) into the automated analysis workflow, we defined each cage as shape-persistent if the cavity diameter was above 1.0 Å and the expected number of windows was present for each topology. Across the four topologies modelled, we expected three windows in a shape-persistent cage in the **Tri**^**2**^**Di**^**3**^ topology, four for **Tri**^**4**^**Di**^**6**^, five for **Tri**^**6**^**Di**^**9**^, and, lastly, six for **Tri**^**8**^**Di**^**12**^. Using these criteria, we were able to computationally predict whether an experimentally observed cage would be likely to remain shape-persistent, and therefore, whether permanent internal cavities are likely to be maintained upon desolvation. Applying these criteria, 307 of the 1464 modelled cages were deemed to be shape-persistent.

However, for the screened **Tri**^**2**^**Di**^**3**^ cage topologies, a large proportion of cages were predicted to have very small cavity sizes, which is to be expected for the small, capsule-like structures, typical for the topological type.^17,29,30^ Small molecular organic cages have been commonly reported in the literature, typically formed from precursors with a greater rigidity and narrower angles between the functional groups – these smaller cages are less likely to collapse, but at the cost of a small internal void size.^30–32^**Tri**^**2**^**Di**^**3**^ organic cages with small internal cavities have still been widely reported in the literature, as despite having a compact structure, often promoted by π–π stacking, they can still assemble in the solid-state to create one-dimensional channels and exhibit extrinsic porosity with high gas selectivity.^29,30^ Other reported applications for these smaller capsular cages also include selective anion and acid binding to remove harmful pollutants and explosives, blending with porous polymers to enhance functions such as the mechanical strength of a polymer of intrinsic microporosity (PIM), and their use as hydrophobic di-receptors to promote the self-assembly of supramolecular polymers.^33–41^ Overall, there were 27 cages that exhibited the correct number of expected windows but had a cavity diameter between 0.1 and 1.0 Å, all with a **Tri**^**2**^**Di**^**3**^ topology, increasing the total of computational non-collapsed cages to 334.

**Fig. 7:**
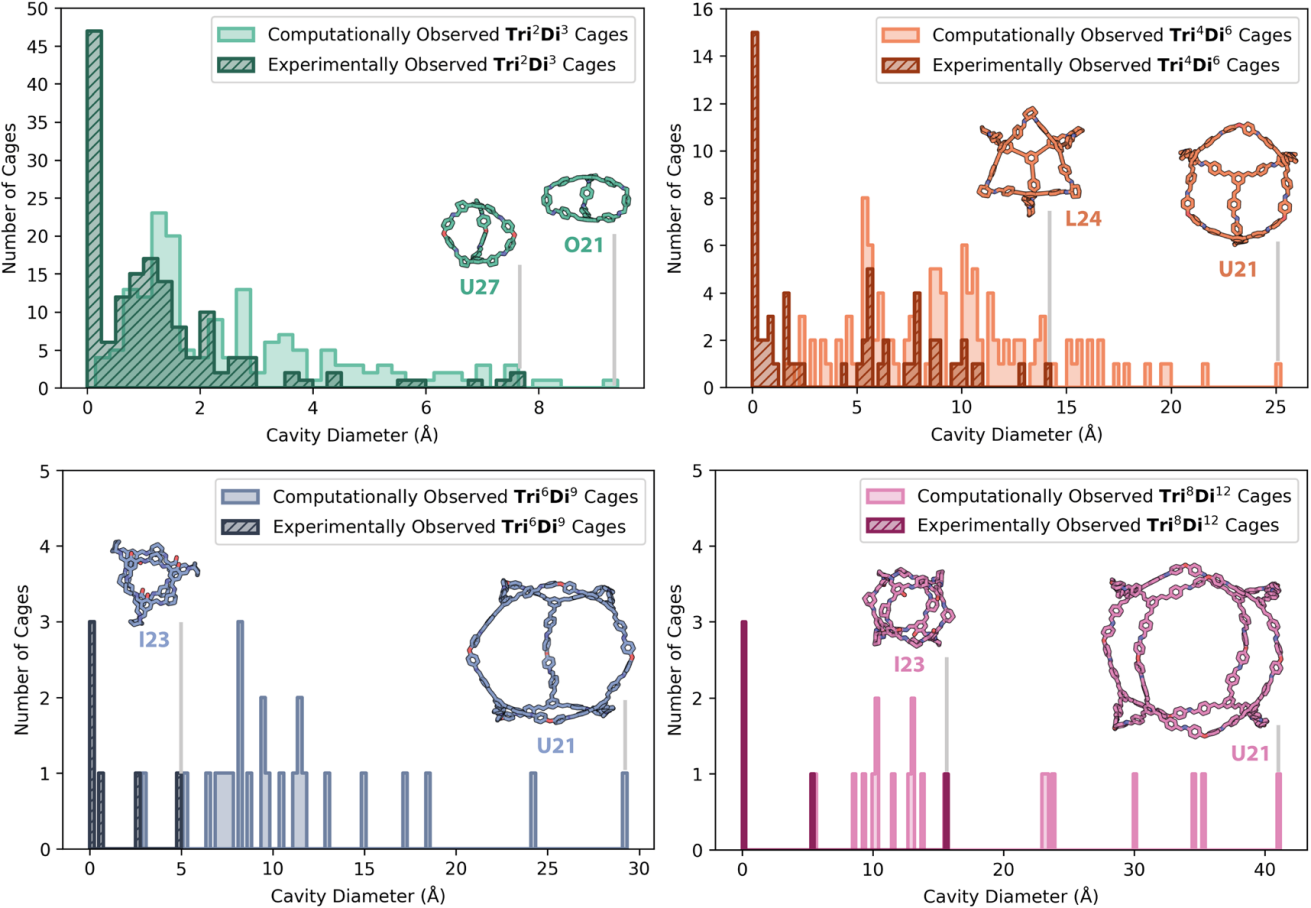
Distribution of cavity diameters for the computationally predicted shape-persistent cages, with a cavity size greater than 0.1 Å and the correct number of windows, for all 366 precursor combinations across four topologies (top left **Tri**^**2**^**Di**^**3**^, top right **Tri**^**4**^**Di**^**6**^, bottom left **Tri**^**6**^**Di**^**9**^, and bottom right **Tri**^**8**^**Di**^**12**^) and for precursor combinations where a specific topology or mixtures of topologies were experimentally observed by HRMS (hatched). The cages with the largest cavity sizes of each topology are shown both for the computationally predicted and experimentally realised structures, labelled according to the precursor combination. Carbon atoms in cages with the **Tri**^**2**^**Di**^**3**^ topology are shown in green, **Tri**^**4**^**Di**^**6**^ in orange, **Tri**^**6**^**Di**^**9**^ in blue, and **Tri**^**8**^**Di**^**12**^ in pink; in addition, nitrogen atoms shown in dark blue, oxygen in red. Hydrogens have been omitted for clarity.

The computationally predicted shape-persistent cages are shown in Fig. 7 for each topology, comprised of 167 (50%) **Tri**^**2**^**Di**^**3**^ cages, 123 (37%) **Tri**^**4**^**Di**^**6**^ cages, 25 (7%) **Tri**^**6**^**Di**^**9**^ cages, and 19 (6%) **Tri**^**8**^**Di**^**12**^ cages. Unsurprisingly, the number of shape-persistent cages decreased with an increasing topology size, following literature trends where larger cages with more chemical bonds, increasing the accumulated flexibility of the discrete molecule, are more prone to pore collapse on removal of solvent molecules in the internal cavity.^42^ The 225 HT experimental results based on the identification of a cage topology by mass spectroscopy, either as a single or as a mix of topologies, are also overlaid for comparison in Fig. 7 (hatched). The highest proportion of experimental hits (154, 68%) were the smallest **Tri**^**2**^**Di**^**3**^ capsule cages, followed by the **Tri**^**4**^**Di**^**6**^ topology (60 combinations, 27%), **Tri**^**6**^**Di**^**9**^ topology (6 combinations, 3%), and finally, the **Tri**^**8**^**Di**^**12**^ topology (5 combinations, 2%), following the same trend as the predicted shape-persistent cages.

Fig. 7 also shows the computationally predicted structures of the shape-persistent cages with the largest internal cavity diameter across each topology, and those that were experimentally observed for comparison, highlighting the difficulty and challenges in experimentally accessing larger cage topologies. **U21** was the most common precursor combination to have the largest predicted cavity size for **Tri**^**4**^**Di**^**6**^, **Tri**^**6**^**Di**^**9**^ and **Tri**^**8**^**Di**^**12**^ cages, with diamine **21** also being in the precursor combination for the largest predicted **Tri**^**2**^**Di**^**3**^ cage (**O21**). However, **O21** and **U21** were not experimentally observed, as only a single combination with diamine **21** resulted in cage formation (**N21** in a **Tri**^**2**^**Di**^**3**^ topology, Fig. 5). As discussed above, trialdehyde **L** performed relatively poorly, with only 27% of **L**'s combinations resulting in any cage formation identified by HRMS. Previously, trialdehyde **L** has only been reported to form [4 + 4] tri-topic + tri-topic combinations that have resulted in POC formation, including combinations with the flexible triamines **B** and **D**.^11^ In this screen, all of its combinations formed insoluble precipitate, failing the turbidity check, which may be attributed to its large rigid structure with four aromatic rings, lowering its solubility, and therefore its availability in solution for DCC reactions. Despite this, the largest experimental **Tri**^**4**^**Di**^**6**^ cage formed was **L24**.

### Precursor trend analysis

**Fig. 8:**
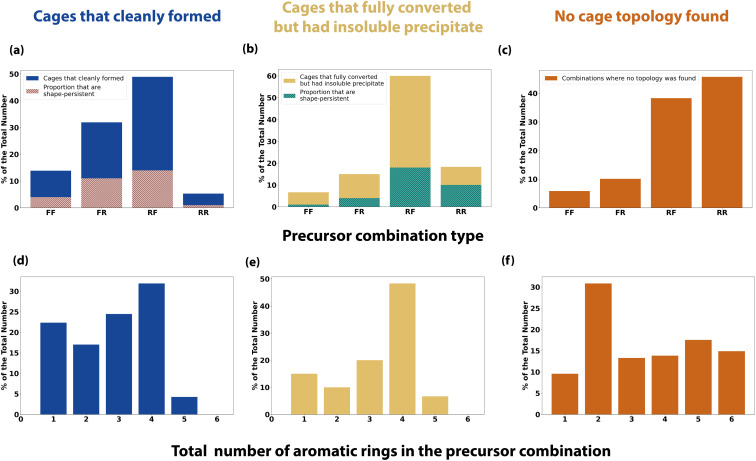
Precursor trends for a subset of the reaction outcomes: (a) and (d) are cages that have formed either as a clean single topology or as a mixture of topologies, where the turbidity, ^1^H NMR and HRMS checks have been passed; (b) and (e) are cages that have formed as either a clean single topology or as a mixture of topologies which have passed the ^1^H NMR and HRMS checks but failed the turbidity checks, showing insoluble precipitate; (c) and (f) are where no cage topology was found, failing the HRMS check. Tri-topic and di-topic precursor combinations are categorised as either flexible–flexible (FF), flexible–rigid (FR), rigid–flexible (RF) or rigid–rigid (RR), and the total number of aromatic rings in the precursor combination is counted. (a) and (b) Both include overlaid percentage of the total number that were also computationally predicted to be shape-persistent (hatched pink and green).

To further analyse the effect of precursor design on the reaction outcome, we defined each precursor as either ‘rigid’ or ‘flexible’ based upon chemical intuition, and counted the number of aromatic rings each had (Table S1†). We then simplified the reaction outcomes into three classifications – (i) clean formation of a cage either as a single topology or as a mixture of topologies (Fig. 8a and d); (ii) formation of a cage either as a single topology or as a mixture of topologies with full conversion, but with insoluble precipitate present (Fig. 8b and e); and (iii) precursor combinations where no cage topology was identified (Fig. 8c and f).

Using these categorisations, we then investigated the effect of precursor rigidity/flexibility and topicity on the reaction outcome by categorising the tri-topic and di-topic precursor combinations as either flexible–flexible (FF), flexible–rigid (FR), rigid–flexible (RF) or rigid–rigid (RR), where the tri-topic precursor's flexibility label is always given first. Fig. 8a shows that for clean formation of an organic cage, either as a single topology or as a mixture of topologies, the largest proportion of successful outcomes incorporated a rigid tri-topic precursor with a flexible di-topic precursor (RF), followed by the inverse, a flexible tri-topic precursor with a rigid di-topic precursor (FR), indicating that there is a preference for clean cage formation when one precursor is rigid and the other flexible. In addition, both these categories resulted in the highest proportion of shape-persistent cages (overlaid hatched bars in Fig. 8a), although still below half of the total number for both RF and FR combinations, whereas a RR precursor combination gave the lowest proportion of cleanly formed cages, along with the lowest proportion of cage shape-persistency. Cage reactions incorporating a FF precursor combination also showed a higher proportion of clean cage outcomes compared to RR, but still of a lower proportion when compared to precursor combinations where rigidity and flexibility are paired.

Fig. 8b shows the combinations where cages formed with full consumption of the aldehyde precursor, but which contained insoluble precipitate, potentially indicating the formation of insoluble polymer or side-products. A RF precursor combination still gave the largest proportion of successful outcomes, following the cleanly formed cages subset. However, a lower disparity between the other categories is observed, especially in relation to the higher proportion of outcomes involving a RR precursor combination. Finally, Fig. 8c shows a reverse trend in the proportion of the precursor combinations that resulted in no cage topology being identified, with the highest proportion of failed outcomes involving a RR precursor combination.

Pre-organisation from precursor design has previously been discussed in the literature as playing a crucial role in successful cage formation and topological outcome, where a higher rigidity can favour the correct orientation of functional groups, promoting cage formation of a certain topology.^11,43,44^ However, Rondelli *et al.* have previously discussed, within the context of boronate ester POCs, that rigid precursors can be prone to geometric mismatches, lowering their adaptability or generalisability in cage formation.^44^ Some degree of flexibility is required to increase the tolerance of the rigid precursor and facilitate cage formation, matching the observation that RF and FR precursor combinations gave the highest proportion of outcomes for reactions that formed both clean cage or cage alongside insoluble precipitate. Flexibility within spacers or linkers in supramolecular chemistry has also been discussed in relation to achieving better binding in host–guest complexes, by overcoming entropic penalties from conformational flexibility.^45^ Therefore, the formation of cages using a RF or FR precursor combination, or indeed a FF precursor combination, may demonstrate improved binding over those formed using a RR precursor combination.

Next, taking into account the total number of aromatic rings in the precursor combinations, for both subsets of cleanly formed cages and cages that fully converted but with insoluble precipitate, a similar trend was observed where the highest proportion of aromatic rings that was typically tolerated was four or less (Fig. 8d–e). However, when there were three or less aromatic rings, clean cage formation without any observed precipitate was more apparent, whereas more combinations containing precipitate were observed when the precursor combination contained at least four aromatic rings. As might be expected, this indicates that a larger number of aromatic rings can result in a greater degree of precipitation, where π stacking may decrease solubility, removing reactants or kinetic side-products from the DCC system, preventing a thermodynamic cage product from forming. This is further illustrated in the combinations where no cage topology was found, with precursor combinations containing six aromatic rings also being present, compared to none being observed in the successful cage formations.

To take one example, trialdehyde **U** has four aromatic rings and was defined as ‘rigid’, with **U21**, the largest computationally predicted cage for **Tri**^**4**^**Di**^**6**^, **Tri**^**6**^**Di**^**9**^ and **Tri**^**8**^**Di**^**12**^ topologies, therefore being a RR precursor combination with a total of 6 aromatic rings – only one combination with **U** resulted in the clean formation of a cage (**U18**, **Tri**^**2**^**Di**^**3**^), but when paired with a complimentary diamine such as **21**, the precursor may have the desired level of preorganisation to promote formation of the three larger topologies. However, we may not experimentally observe any of these cages under the HT screening conditions due to the large number of aromatic rings decreasing the precursor and intermediate solubilities, leading to precipitation and removal of these species from the dynamic reaction in solution. Altering the reaction conditions, such as changing the solvent or further decreasing the overall reaction concentration, may result in accessing these predicted larger, shape-persistent cages. In contrast, **I23** was a combination that went to full conversion but also formed insoluble precipitate and a mixture of topologies, but resulted in both the largest **Tri**^**6**^**Di**^**9**^ and **Tri**^**8**^**Di**^**12**^ experimentally observed cages. Trialdehyde **I** has a single aromatic ring and was defined as ‘rigid’, while diamine **23** has no aromatic rings and was defined as ‘flexible’, following the observation that a degree of flexibility can promote cage formation. In this specific reaction there may be a thermodynamic preference for one cage topology, however, the precipitation of intermediates and oligomeric species may have perturbed the ‘error-correction’ mechanism preventing equilibration towards a single cage topology – a change in the reaction conditions such as the solvent, concentration, or reaction time, may therefore result in a change in the topological outcome.

Overall, from this precursor trend analysis of this specific high-throughput screen, it suggests that a rigid and flexible precursor combination (RF or FR), along with no more than four aromatic rings in the pair, would favour clean cage formation without the presence of insoluble precipitate.

### Hits from the high-throughput workflow

**Fig. 9:**
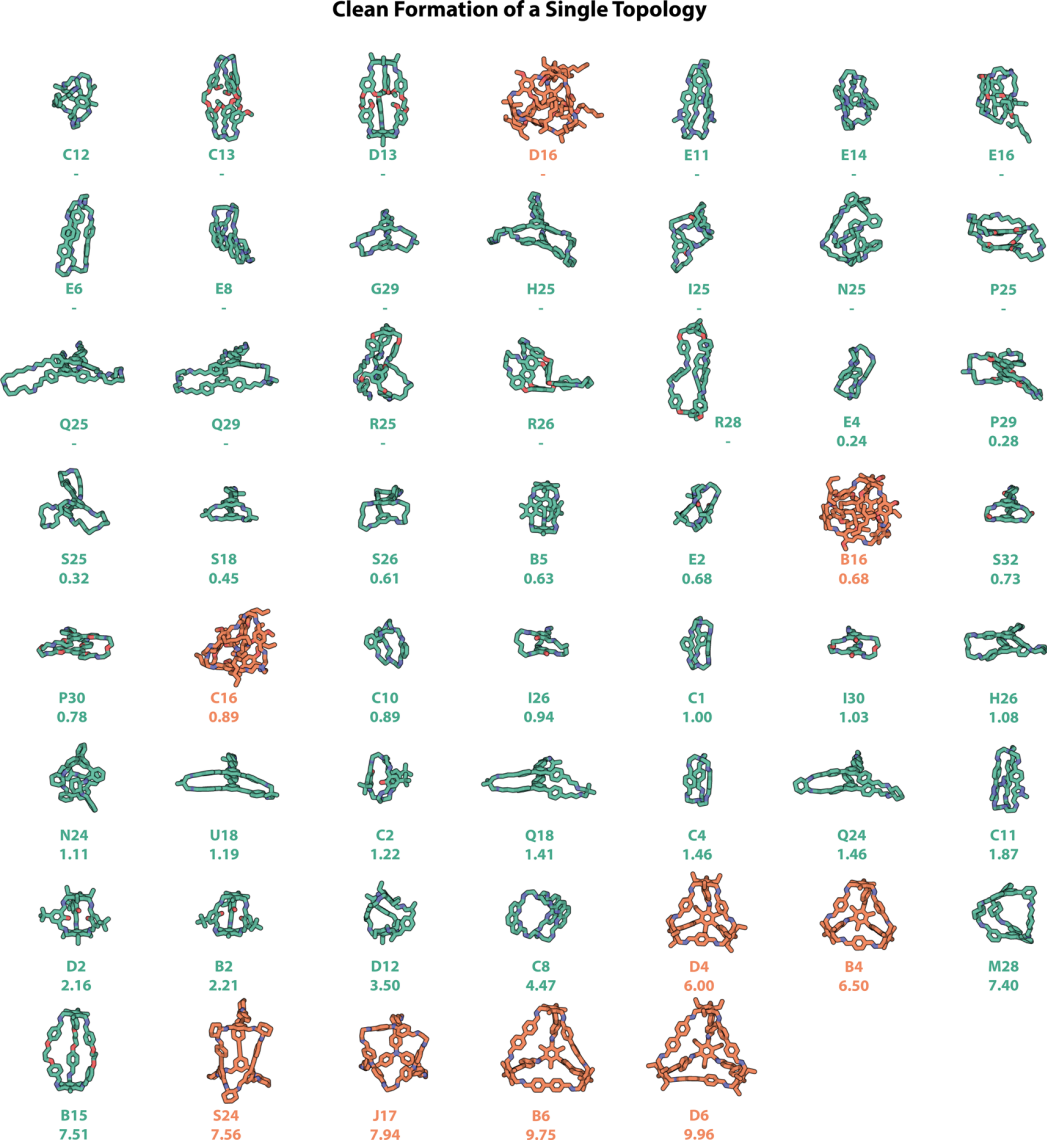
Computationally modelled structures of the precursor combinations that passed computer vision turbidity, NMR and HRMS experimental checks, and therefore were assigned as resulting in the ‘clean formation of a single topology’. Cage cavity diameters are given under the precursor combination label. No cavity is reported if the cage does not have the correct number of windows and/or no cavity diameter could be calculated computationally from the predicted structure, indicating the absence of a shape-persistent internal cavity. Hits are shown in order of increasing cavity diameter if one could be calculated, and alphabetically prior. Carbon atoms in cages with the **Tri**^**2**^**Di**^**3**^ topology are shown in green, and **Tri**^**4**^**Di**^**6**^ in orange; in addition to nitrogen atoms shown in dark blue, oxygen in red. Hydrogens have been omitted for clarity.

The computationally predicted structures of the 54 experimentally realised organic cages that formed one singular topology ‘cleanly’ (where all three experimental methods of automated characterisation resulted in a ‘pass’) are shown in Fig. 9, with those which formed alongside insoluble precipitate shown in Fig. S13,† and those which cleanly formed mixtures of topologies shown in Fig. S14.† Currently, to the best of our knowledge, this study represents the highest number of cleanly formed cages from a single screen, a considerable number when the total number of POCs discovered to date is only in the hundreds.^11^ Of these 54 cages, only the most common **Tri**^**2**^**Di**^**3**^ and **Tri**^**4**^**Di**^**6**^ topologies were observed. However, as discussed above, opportunities and applications can still arise for cages with smaller cavities that may not possess intrinsic porosity, but may pack in the solid-state to create extrinsic pore channels, and find applications in sensing in solution.^29,40,41^

Precursor combinations that have equilibrated to a single molecular species without insoluble precipitate, and that form a shape-persistent cage, would be the most desirable from a materials discovery perspective, and in relation to POCs, would require a pore size over 2.89 Å, the kinetic radius of a hydrogen molecule, in order to more likely find application in gas uptake and separations.^46^ POCs with larger cavities are particularly challenging to synthesise due to the increased likelihood of collapsing on desolvation. In addition, the precursors that lead to shape-persistent organic cages with a cavity large enough for guest molecules, *i.e.*, POCs, are often larger, resulting in greater degrees of freedom and competing synthesis pathways for the formation of alternate products, alongside easier collapse mechanisms. However, using the hybrid workflow and automated data analysis, we identified ten POCs that would be considered porous, based on having a pore size over 2.89 Å – these are shown as the last ten molecules in Fig. 9. The largest of these cages are **B6** and **D6**, with cavities of 9.75 and 9.96 Å respectively – however, while these, alongside the fourth largest pore-containing cage **S24**, have been reported previously,^11,47^ several new organic cages with large pore sizes were discovered including **J17** (7.94 Å) and the **Tri**^**2**^**Di**^**3**^ cages **M28** (7.40 Å) and **B15** (7.51 Å).

In addition, while **16** (2-((2-ethylhexyl)oxy)-5-methoxyterephthalaldehyde) was highlighted as the best performing dialdehyde in relation to the synthetic screen, with 67% of its combinations resulting in the clean formation of a single topology (Fig. 6), it did not lead to any POCs. While the preferential reactivity of dialdehyde **16** follows literature observations where functionalising precursors with either alkyl or ether chains can increase their solubility, and therefore conversion to a single cage species, it can also result in low porosity due to the chains occupying the internal cavity (such as in **B16**, **C16** and **D16** in **Tri**^**4**^**Di**^**6**^ topologies, Fig. 9).^9,48,49^ However, when these side-chains are removed, such as in dialdehyde **4** which has the same terephthalaldehyde core as **16**, in combination with the same triamines **B** and **D**, **Tri**^**4**^**Di**^**6**^ cages may be formed with predicted internal cavity diameters exceeding 6 Å as a result of their structural rigidity and lack of chain occupancy.

Finally, while an excess of tri-topic or di-topic amine was used in the HT screen, the ^1^H NMR and mass spectra of the 54 identified cages were manually inspected and interpreted where possible to confirm the identification of the formed cages. Overall, 42 combinations were characterised (see Section S6.1 and S6.2†), with the spectra of the remaining 12 also included in the latter. Those which were more difficult to interpret typically involved precursors with a reduced symmetry compared to their topicity (such as **S**), or that could form unsymmetrical species (such as **P**), meaning they passed all of the automated data analysis but often contained either broad peaks or complex splitting on manual inspection.

However, perhaps more interestingly, manual inspection also led to the identification of potential competitive reactivity for some precursor combinations. This included precursors **12** and **Q** which contain α,β-unsaturated aldehydes, where addition of a nucleophile, such as the primary amine precursors used in this screen, have the potential to result in competitive 1,4- *vs.* 1,2-addition. Use of a soft nucleophile, such as a primary amine, has previously been shown to preferentially undergo 1,2-addition, yielding products of α,β-unsaturated imines that have been rationalised as the kinetically favoured product.^50^ The absence of the aldehyde peak in the automated ^1^H NMR analysis follows this suggestion, however, we do acknowledge the potential for this competitive pathway to occur, as tautomerisation to an enol from the 1,4-addition would also lead to the absence of an aldehyde peak and have indicated this in Section S6.1.† In addition, we previously reported the potential for competitive aminal formation over imine formation during the synthesis of organic cages,^51^ and we believe this was also observed here in combinations containing precursor **18**, which may be attributed to the Thorpe-Ingold effect where the gem-dimethyl functionality favours intramolecular cyclisation to the aminal over intermolecular imine formation to form a cage species. When combined with trialdehydes **S** and **U**, the ditopic amine precursor **18** passed all of the automated data analysis, with both indicating the formation of a **Tri**^**2**^**Di**^**3**^ topology based on the HRMS analysis. However, on manual inspection of the ^1^H NMR spectra, we observed evidence for both cage and aminal formation, further confirmed through identification of the [1 + 3] aminal-intermediate species in the HRMS spectra (Fig. S162 and S174†).

Overall, this does highlight the potential for missing serendipitous discoveries when automating the data analysis and categorising the reaction outcomes, as the automated workflow identifies the relative reaction outcome of each experimental characterisation technique. However, we believe it does not outweigh the benefits gained from rapid data collation and interpretation as it still accelerates the identification of cages with known topologies, paving the way for a data-led approach in POC discovery. In addition, the automated data analysis could be expanded to include the identification of potential intermediates for each cage topology, and competing chemistries, to get a broader understanding of the reaction pathway and outcome.

## Conclusions

In conclusion, we have developed a hybrid automated workflow combining automated high-throughput experimentation, sample preparation, and characterisation, with automated data analysis, computational modelling, and property prediction, for the accelerated screening of organic cages formed using dynamic covalent chemistry. The synthesis of organic cages has previously been translated to an automated high-throughput experimental method, but fundamental limitations remained around the initial cost outlay of high-throughput automated equipment, methods for sample preparation and characterisation that could tolerate a high number of samples and keep up with throughput, and by far the greatest bottleneck, both here and more broadly in the field of chemical automation, was the data analysis of the large number of generated samples. Here, we applied a combination of low-cost open-source automation, computer vision for turbidity assessment, and software that automated the analysis of ^1^H NMR and HRMS spectra for assessment of the species type, conversion, and cage topology. This provides a low-cost screening and high-throughput assessment of the reaction outcomes on a feasible timescale. By further integrating these experimental characterisation methods into a fully automated Python pipeline, *cagey*, we can drastically reduce the total time of a researcher spent on data analysis with accuracies of above 95%. For example, to now fully characterise all 366 precursor combinations presented here, including file conversion and full analysis, this takes under 15 minutes, which arguably would only be enough time for a researcher to manually analyse a single combination at best, meaning the time required for data analysis is reduced over 350-fold, a significant saving in researcher time, especially for large scale data curation.

Using our streamlined automated workflow, we can direct future synthesis by exploring the computationally predicted structures and properties from initial experimental hits and thus focus future experimental efforts on promising systems. Here, we were also able to use the computational screening to explain reaction outcomes *post-priori*. Of the 366 precursor combinations reported in this work, we observe a large range of outcomes, further highlighting the complexity in predicting DCC reaction outcomes. In addition, by selecting a broad building block library of precursors of varying topicity, functionality, and rigidity, and by automating the synthesis, sample preparation, characterisation, and analysis of the high-throughput screen, we can identify clear trends in the precursors, combinations and combination type, which researchers may consider when targeting POC formation. Additionally, curation of the largest experimental-computational machine-readable dataset of organic cages currently in the literature means that opportunities arise for a data-driven approach towards POC discovery. As discussed, targeting the clean formation of a singular cage topology is ideally the most desirable outcome as it requires the least amount of post-synthetic processing. However, we have also identified interesting combinations where larger cages, which are predicted to be shape-persistent POCs, either formed as mixtures or also formed insoluble precipitate, or did not undergo full conversion. A large chemical space of possible organic cages, and in particular, POCs with large internal cavities, still remains, which can be better navigated using this streamlined, automated workflow on a reasonable timescale due to its low-cost and accessibility. Furthermore, the streamlined workflow is applicable to other supramolecular assemblies, thus allowing accelerated discovery of supramolecular materials more generally.

## Data availability

The experimental procedures and computational details are available within the manuscript and its ESI.† All the raw experimental data files can be found on Zenodo (https://doi.org/10.5281/zenodo.10675206), with a key mapping each precursor combination to each data file path *via* experiment code, plate number and formulation number available. The computational models, 3D printing files, and experimental synthesis and characterisation code can be found on GitHub (https://github.com/GreenawayLab/Streamlining-Automated-Discovery-POCs and https://github.com/GreenawayLab/cagey).

## Author contributions

R. L. G. and K. E. J. conceived the project, with R. L. G. leading the high-throughput automated workflow and K. E. J. leading the computational modelling. A. R. B. and S. K. B. prepared the precursors, carried out the high-throughput synthesis and characterisation, developed the OT-2, automated NMR and HRMS data analysis scripts, and carried out the modelling. L. T. developed the fully automated analysis cagey package with assistance from A. R. B. and S. K. B. M. X. and J. A. developed and carried out the computer-vision based turbidity measurements with assistance from A. R. B. The paper was written by A. R. B., S. K. B., and R. L. G. with input from all authors.

## Conflicts of interest

The authors declare no conflict of interest.

## Supplementary Material

SC-015-D3SC06133G-s001
