# Supplementary material for: Streamlining the automated discovery of porous organic cages
Source: Chem Sci. 2024 Mar 13;15(17):6331–48. doi: 10.1039/d3sc06133g (PMC11062116; doi:10.1039/d3sc06133g)
Supplement: SC-015-D3SC06133G-s001 [file SC-015-D3SC06133G-s001.pdf]

Electronic Supplementary Material (ESI) for Chemical Science.
This journal is © The Royal Society of Chemistry 2024

Supporting Information

Streamlining the Automated Discovery of Porous Organic Cages

Annabel R. Basford,[a] Steven K. Bennett,[a] Muye Xiao,[a] Lukas Turcani,[a] Jasmine Allen,[a] Kim. E. Jelfs,[a]* Rebecca L. Greenaway[a]*

[a] Department of Chemistry, Molecular Sciences Research Hub, Imperial College London, White City Campus, 82 Wood Lane, W12 0BZ, UK

Email: k.jelfs@imperial.ac.uk; r.greenaway@imperial.ac.uk

Contents

S1. General Synthetic & Analytical Methods 3
S2. Building Block Library 5
S3. Synthesis of Precursor Building Blocks 8
S3.1 Procedures and Characterisation Data 8
S3.2 Spectra 15
S4. Experimental High-throughput Workflow 18
S5. Automated Data Analysis 35
S5.1 Turbidity 35
S5.2 ^{1}H NMR Spectra 39
S5.3 High Resolution Mass Spectrometry (HRMS) 41
S6. High-Throughput Experimental Screen 46
S6.1 Characterisation Data 46
S6.2 Spectra 56
S7. Computational Modelling and Automated Analysis 164
S7.1 Cage Construction and Conformational Searching 164
S7.2 Shape-Persistence Evaluation 164
S8. References 166

S1. General Synthetic & Analytical Methods

Materials: Chemicals were purchased from TCI UK, Fluorochem, or Sigma-Aldrich, and used as received. Solvents were reagent or HPLC grade and purchased from Fisher Scientific, with the exception of chloroform-*d* which was purchased from Apollo Scientific. All solvents and chemicals were used as received, unless specified.

Synthesis: All reactions requiring anhydrous or inert conditions were performed in oven-dried apparatus under an inert atmosphere of dry nitrogen, using anhydrous solvents introduced into the flask using disposable needles and syringes. All reactions were stirred magnetically using Teflon-coated stirring bars. Where heating was required, the reactions were warmed using a stirrer hotplate with heating blocks with the stated temperature being measured externally to the reaction flask with an attached probe. Removal of solvents was carried out using a rotary evaporator.

High-throughput cage discovery: High-throughput experimentation and sample preparation was conducted using an Opentrons OT-2 liquid handling platform (robot v3.6.1 and 6.3.1 app version).[1] Protocols were written using OT-2 Python Protocol API Version 2.9, and the code required to replicate the protocol is available at https://github.com/GreenawayLab/Streamlining-Automated-Discovery-POCs/tree/main/Opentrons_Protocols. The OT-2 was fitted with a Single-Channel GEN2 300 µL pipette and Single-Channel GEN2 20 µL pipette and used with Opentrons OT-2 Tips, 300 µL and 20 µL respectively. Both 24-well (for 8 mL vials) and 48-well (for 2 mL vials) reactor blocks were purchased from Analytical Sales, with 12 mm OD rubber mats and 12 mm OD PFA films, and the 6-well ANSI/SLAS microplate holder for DURAN 25 mL bottles was purchased from VWR. Micro PTFE stirrer bars (2 x 5 mm) were purchased from Scientific Laboratory Supplies and used across the high-throughput screens. Parallel solvent evaporation was conducted using an Analytical Sales EquaVAP 48-well evaporator.[2]

TLC: Reactions were monitored by thin layer chromatography (TLC) where required, conducted on pre-coated aluminium-backed plates (Merck Kieselgel 60 with fluorescent indicator UV254). Spots were visualized either by quenching of UV fluorescence or by staining with potassium permanganate.

FTIR: Infrared spectra were recorded using an Agilent Technologies Cary 630 FTIR spectrometer. Spectra were acquired across a 4000-400 cm^{-1} range, with 32 background scans, a resolution of 16 and a Happ-Genzel Apodization.

NMR Spectra: ^{1}H and ^{13}C NMR spectra were obtained using a Bruker AV400 (400 MHz/101 MHz) for precursor characterisation using 7 inch, OD 5.0 mm tubes. High-throughput cage characterisation samples were prepared using the Opentrons OT-2 with chloroform-*d*, and ^{1}H NMR spectra obtained using a 'SampleXpress' automatic sample changer or a Bruker DRX500 (500 MHz/126 MHz) fitted with a SampleJet sample changer that allows both single tube submission and 96 well plate arrays of 5 inch, OD 5.0 mm tubes in a fully automated system.[3] Spectra were acquired using a deuterium lock in $CDCl_3$ (δ = 7.26 ppm) or $(CD_3)_2SO$ (δ = 2.52 ppm) for ^{1}H NMR, and $CDCl_3$ (δ = 77.16 ppm) or $(CD_3)_2SO$ (δ = 39.52 ppm) for ^{13}C NMR. NMR data are presented as follows: chemical shift, peak multiplicity (s = singlet, d = doublet, t = triplet, q = quartet, qu = quintet, m = multiplet, br = broad, app = apparent), coupling constants (J / Hz), and integration. Chemical shifts are expressed in ppm on a δ scale relative to δ_{CDCl3} (7.26 ppm) and coupling constants, J, are given in Hz.

HRMS (ESI+): High-resolution mass spectrometry (HRMS) for precursor characterisation was conducted using a Walters LCT Premier (ES-ToF)/Acquity i-Class in a single mass analysis. High-throughput cage characterisation was carried out on an Agilent 1290 Infinity II with 6530 Q-TOF MS LC/Q-TOF spectrometer. For each cage reaction, a 20 µL volume of reaction mixture was transferred into 1 mL of 50:50 MeOH:DCM. HRMS was undertaken where the mobile solvent was MeOH + 0.1 % formic acid at a flowrate of 0.25 mL/min, capillary voltage 4000 V, fragmentor 225 V, in positive ion mode.

Turbidity: A Raspberry Pi 4 Desktop was connected to a web camera (Microsoft LifeCam HD-3000) and was used to run the computer vision script (https://github.com/GreenawayLab/Streamlining-Automated-Discovery-POCs/tree/main/Turbidity-monitoring-using-computer-vision). A reference sample of the reaction solvent chloroform in a 2 mL vial was positioned within a 3D-printed 2-vial holder, printed with black PLA (https://github.com/GreenawayLab/Streamlining-Automated-Discovery-POCs/tree/main/3D-printing) and placed on a non-reflective black surface, and measurements taken of reaction samples in the other position. During each measurement, the script monitored the sample for a maximum period of two minutes and terminated monitoring whenever a "dissolved" or a "stable" (equilibrium reached but not dissolved) status was detected by the Python package *HeinSight*.[4,5]

S2. Building Block Library

Key

Tri-topic

A 2-(aminomethyl)-2-methylpropane-1,3-diamine
B (2,4,6-trimethylbenzene-1,3,5-triyl)trimethanamine
C *N*1,*N*1-bis(2-aminoethyl)ethane-1,2-diamine
D (2,4,6-triethylbenzene-1,3,5-triyl)trimethanamine
E *N*1,*N*1-bis(3-aminopropyl)propane-1,3-diamine
F (1*S*,3*S*,5*S*)-cyclohexane-1,3,5-triamine
G 1,3,5-triformylbenzene
H 5'-(4-formylphenyl)-[1,1':3',1''-terphenyl]-4,4''-dicarbaldehyde
I 2-hydroxybenzene-1,3,5-tricarbaldehyde
J 4,4',4''-nitrilotribenzaldehyde
K 4,4',4''-(1,3,5-triazine-2,4,6-triyl)tribenzaldehyde
L 4,4',4''-(benzene-1,3,5-triyltris(ethyne-2,1-diyl))tribenzaldehyde
M 5'-(3-formylphenyl)-[1,1':3',1''-terphenyl]-3,3''-dicarbaldehyde
N 5'-(2-formylphenyl)-[1,1':3',1''-terphenyl]-2,2''-dicarbaldehyde
O 4,4',4''-(benzene-1,3,5-triyl)tris(thiophene-2-carbaldehyde)
P 5,5',5''-(benzene-1,3,5-triyl)tris(furan-2-carbaldehyde)
Q 1,3,5-tris-(3-acetaldehydebenzene)-benzene
R 4,4',4''-((benzene-1,3,5 triyltris (methylene))tris(oxy))tribenzaldehyde
S 5-((4-formylphenyl)ethynyl)isophthalaldehyde
T 2,4,6-triformylphloroglucinol
U 1,3,5-tris(*p*-formylstyryl)benzene

Di-topic

1 isophthalaldehyde
2 5-(*tert*-butyl)-2-hydroxyisophthalaldehyde
3 thiophene-2,3-dicarbaldehyde
4 terephthalaldehyde
5 2,3,5,6-tetrafluoroterephthalaldehyde
6 4,4'-biphenyldicarboxaldehyde
7 anthracene-9,10-dicarbaldehyde
8 1,10-phenanthroline-2,9-dicarbaldehyde
9 thiophene-2,3-dicarbaldehyde
10 phthaldialdehyde
11 3-(4-formylphenyl)-1-methyl-1H-pyrazole-5-carbaldehyde
12 (2*Z*,2'*Z*)-3,3'-(1,4-phenylene)bis(2-methylacrylaldehyde)
13 4,4'-(ethane-1,2-diylbis(oxy))bis(3-methoxybenzaldehyde)
14 2-methyl-2*H*-indazole-3,7-dicarbaldehyde
15 4,4'-(propane-1,3-diylbis(oxy))dibenzaldehyde
16 2-((2-ethylhexyl)oxy)-5-methoxyterephthalaldehyde
17 ethane-1,2-diamine
18 2,2-dimethylpropane-1,3-diamine
19 (3*S*,4*S*)-1-benzylpyrrolidine-3,4-diamine
20 (1*R*,2*R*)-1,2-diphenylethane-1,2-diamine
21 4,4'-oxydianiline
22 1,2,5-oxadiazole-3,4-diamine
23 (1*R*,3*S*)-cyclohexane-1,3-diamine
24 (1*R*,2*R*)-cyclohexane-1,2-diamine
25 bis(hexamethylene)triamine
26 hexamethylenediamine
27 *p*-xylylenediamine
28 [4-(aminomethyl)cyclohexyl]methylamine
29 3,3′-diamino-*N*-methyldipropylamine
30 2-(2-aminoethoxy)ethylamine
31 *m*-xylylenediamine
32 1,3-diamino-2-propanol
33 D-lysine
34 *trans*-1,4-diaminocyclohexane

Table S1: Precursor building block property assignments.

Precursor	Rigid or Flexible?	Number of Aromatic Rings
**A**	Rigid	**1**
**B**	Flexible	**1**
**C**	Flexible	**0**
**D**	Flexible	**1**
**E**	Flexible	**0**
**F**	Rigid	**0**
**G**	Rigid	**1**
**H**	Rigid	**4**
**I**	Rigid	**1**
**J**	Rigid	**3**
**K**	Rigid	**4**
**L**	Rigid	**4**
**M**	Rigid	**4**
**N**	Rigid	**4**
**O**	Rigid	**4**
**P**	Rigid	**4**
**Q**	Flexible	**4**
**R**	Flexible	**4**
**S**	Rigid	**2**
**T**	Rigid	**1**
**U**	Rigid	**4**
**1**	Rigid	**1**
**2**	Rigid	**1**
**3**	Rigid	**2**
**4**	Rigid	**1**
**5**	Rigid	**1**
**6**	Rigid	**2**
**7**	Rigid	**3**
**8**	Rigid	**3**
**9**	Rigid	**1**

**10**	Rigid	**1**
**11**	Rigid	**2**
**12**	Flexible	**1**
**13**	Flexible	**2**
**14**	Rigid	**2**
**15**	Flexible	**2**
**16**	Rigid	**1**
**17**	Flexible	**0**
**18**	Flexible	**0**
**19**	Rigid	**1**
**20**	Flexible	**2**
**21**	Rigid	**2**
**22**	Rigid	**1**
**23**	Rigid	**0**
**24**	Rigid	**0**
**25**	Flexible	**0**
**26**	Flexible	**0**
**27**	Flexible	**1**
**28**	Flexible	**0**
**29**	Flexible	**0**
**30**	Flexible	**0**
**31**	Flexible	**1**
**32**	Flexible	**0**
**33**	Flexible	**0**
**34**	Rigid	**0**

S3. Synthesis of Precursor Building Blocks

S3.1 Procedures and Characterisation Data

2,2',2''-((2,4,6-Trimethylbenzene-1,3,5-triyl)tris(methylene))tris(isoindoline-1,3-dione), S1

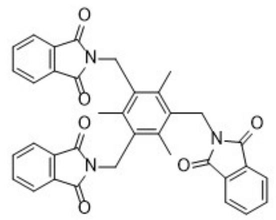

A modification of the procedure of Greenaway *et al.* was used for this reaction.[6] To a solution of 1,3,5-tris(bromomethyl)-2,4,6-trimethylbenzene (5.00 g, 12.53 mmol, 1.0 eq.) and 18-crown-6 (0.99 g, 3.76 mmol, 0.3 eq.) in toluene (160 mL) was added potassium phthalimide (8.35 g, 45.12 mmol, 3.6 eq.). The mixture was heated at 100 ^{o}C under N_2 for 24 h before being allowed to cool to room temperature. The mixture was concentrated *in vacuo* and the resulting solid suspended in water (100 mL) and collected by filtration. The collected solid was further washed with water (100 mL) and MeOH (100 mL) before being dried *in vacuo* to afford the desired product **S1** as a colourless solid which was used without further purification (6.09 g, 10.51 mmol, 81%).

IR (ν_{max}/cm^{-1}) 1698 (C=O), 1850 (ArC-ArC); **^{1}H NMR** (400 MHz, $CDCl_3$) δ_H 7.78 (dd, *J* = 5.4, 3.1 Hz, 6H), 7.68 (dd, *J* = 5.4, 3.0 Hz, 6H), 4.95 (s, 6H), 2.49 (s, 9H); **^{13}C NMR** (101 MHz, $CDCl_3$) δ_C 168.5, 138.7, 134.0, 132.1, 130.3, 123.6, 123.5, 38.6, 17.5. Data in accordance to literature values.[6]

(2,4,6-Trimethylbenzene-1,3,5-triyl)trimethanamine, B

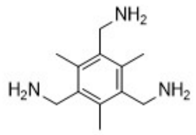

A modification of the procedure of Greenaway *et al.* was used for this reaction.[6] To a suspension of **S1** (6.00 g, 10.04 mmol, 1.0 eq.) in a mixture of toluene (90 mL) and EtOH (185 mL) was added hydrazine hydrate in a single portion (7.5 mL, 50 wt% solution in water, 60.24 mmol, 6.0 eq.). The resulting mixture was heated at 90 ^{o}C for 5 days before being allowed to cool to room temperature. The reaction mixture was concentrated *in vacuo* (not to dryness) and partitioned between aq. KOH solution (200 mL, 40 wt%) and $CHCl_3$ (125 mL). The aqueous layer was further extracted with $CHCl_3$ (2 x 100 mL) before the combined organic layers were dried (Na_2SO_4) and concentrated *in vacuo* to afford the desired triamine **B** as a pale yellow solid which was used without further purification (1.79 g, 8.64 mmol, 86%).

IR (ν_{max}/cm^{-1}) 3350 (br, N-H), 2998 (br, N-H), 2990 (N-H), 2850 (N-H); **^{1}H NMR** (400 MHz, $CDCl_3$) δ_H 3.92 (s, 6H), 2.45 (s, 9H), 1.25 (br, 6H), 0.86 (s, 1H); **^{13}C NMR** (101 MHz, $CDCl_3$) δ_C 138.2, 133.6, 41.0, 15.6; **HRMS** (ESI+) calculated for $C_{12}H_{21}N_3$ 207.1741, found 208.1816 $[M+H]^+$. Data in accordance to literature values.[6]

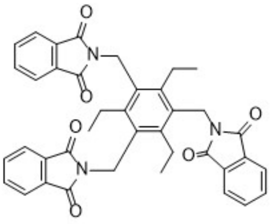

2,2',2''-((2,4,6-Triethylbenzene-1,3,5-triyl)tris(methylene))tris(isoindoline-1,3-dione), S2

A modification of the procedure of Greensaway *et al.* was used for this reaction.[6] To a solution of 1,3,5-tris(bromomethyl)-2,4,6-triethylbenzene (4.83 g, 10.94 mmol, 1.0 eq.) and 18-crown-6 (0.87 g, 3.28 mmol, 0.3 eq.) in toluene (140 mL) was added potassium phthalamide (7.29 g, 39.38 mmol, 3.6 eq.). The mixture was heated at 100 °C under N_2 for 20 h before being allowed to cool to room temperature. The mixture

was concentrated *in vacuo* and the resulting solid suspended in water (100 mL) and collected by filtration. The collected solid was further washed with water (2 × 100 mL) and MeOH (100 mL) before being dried *in vacuo* to afford the desired product **S2** as a colourless solid which was used without further purification (5.89 g, 9.20 mmol, 84%).

IR (v_{max}/cm^{-1}) 2995 (C-H), 1710 (sharp, C=O); **^{1}H NMR** (400 MHz, $CDCl_3$) δ_H 7.80 (dd, *J* = 5.6, 3.1 Hz, 6H), 7.68 (dd, *J* = 5.5, 3.1 Hz, 6H), 4.95 (s, 6H), 3.10 (q, *J* = 7.5 Hz, 6H), 0.96 (t, *J* = 7.2 Hz, 9H); **^{13}C NMR** (101 MHz, $CDCl_3$) δ_C 168.5, 145.5, 134.0, 132.2, 129.6, 123.5, 37.6, 23.5, 16.0. Data in accordance to literature values.[6]

(2,4,6-Triethylbenzene-1,3,5-triyl)trimethanamine, D

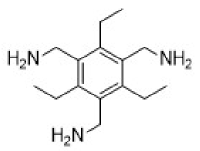

A modification of the procedure of Greenaway *et al.* was used for this reaction.[6] To a suspension of **S2** (5.79 g, 9.05 mmol, 1.0 eq.) in a mixture of toluene (83 mL) and EtOH (170 mL) was added hydrazine hydrate in a single portion (7.5 mL, 50 wt% solution in water, 60.24 mmol, 6.0 eq.). The resulting mixture was heated at 90 °C for 44 h before being allowed to cool to room temperature. The reaction mixture was concentrated *in vacuo* (not to dryness), and then partitioned between aq. KOH solution (200 mL, 40 wt%) and $CHCl_3$ (125 mL). The aqueous layer was further extracted with $CHCl_3$ (2 x 100 mL) before the combined organic layers were dried (Na_2SO_4) and concentrated *in vacuo* to afford the desired product **D** as a pale pink solid which was used without further purification (2.13 g, 8.54 mmol, 94%).

IR (v_{max}/cm^{-1}) 3450 (N-H), 3250 (N-H), 2995 (sharp, N-H); **^{1}H NMR** (400 MHz, $CDCl_3$) δ_H 3.88 (s, 6H), 2.83 (q, *J* = 7.5 Hz, 6H), 1.34 (br, 6H), 1.23 (t, *J* = 7.5 Hz, 9H); **^{13}C NMR** (101 MHz, $CDCl_3$) δ_C 140.5, 137.5, 39.5, 22.7, 16.0; **HRMS** (ESI+) calculated for $C_{15}H_{27}N_3$ 249.2210, found 250.2274 $[M+H]^+$. Data in accordance with literature values.[6]

5'-(4-Formylphenyl)-[1,1':3',1''-terphenyl]-4,4''-dicarbaldehyde, H

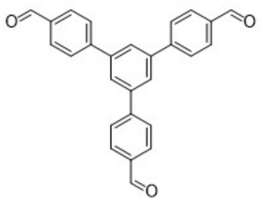

H had been previously synthesised by Greenaway *et al.* and was used directly in this work.[6]

4,4',4''-(1,3,5-Triazine-2,4,6-triyl)tribenzaldehyde, K

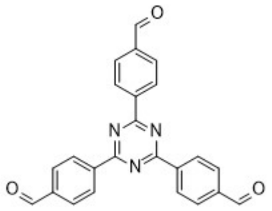

To a stirred, degassed solution (N_2 bubbling) of cyanuric chloride (1.55 g, 8.41 mmol, 1 eq.) in 1,4-dioxane (124 mL), was added 4-formylphenylboronic acid (4.42 g, 29.48 mmol, 3.5 eq.), K_2CO_3 (10.47 g, 75.75 mmol, 9 eq.), H_2O (31 mL), and $Pd(dppf)Cl_2$.DCM (687 mg, 0.841 mmol, 0.1 eq.). The resulting mixture was stirred at 90 °C overnight under N_2 before being allowed to cool to room temperature. The reaction mixture was concentrated *in vacuo*, and the product partitioned between water (200 mL) and $CHCl_3$ (300 mL). The aqueous layer was further extracted with $CHCl_3$ (300 mL) before the combined organic layers were dried ($NaSO_4$) and concentrated *in vacuo* to ~20 mL. Hexane (200 mL) was subsequently added and a

pale brown solid precipitated. The resulting suspension was further concentrated to 100 mL and the solid collected by filtration before drying *in vacuo* to yield **K** as a pale brown solid (0.493 g, 1.23 mmol, 15%).

IR (v_{max}/cm^{-1}) 3052 (w), 2726 (w), 1692 (m, C=O), 1582 (w), 1503 (s), 1354 (m), 1189 (s), 1116 (w), 1011 (w), 803 (s, ArC-H) =, 695 (w, ArC-H); **^{1}H NMR** (400 MHz, $CDCl_3$) δ_H 10.20 (s, 3H), 8.96 (d, *J* = 8.0 Hz, 6H), 8.14 (d, *J* = 7.9 Hz, 6H); **^{13}C NMR** (101 MHz, $CDCl_3$) δ_C 192.0, 140.8, 139.2, 130.0, 129.5. Data in accordance with literature values.[7]

4,4',4''-(Benzene-1,3,5-triyltris(ethyne-2,1-diyl))tribenzaldehyde, L

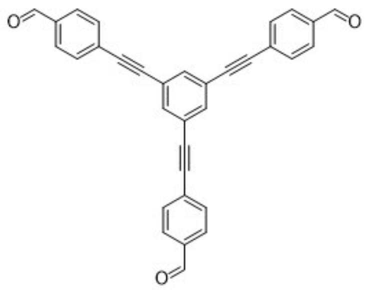

A modification of the procedure of Greenaway *et al.* was used for this reaction.[6] To an oven-dried round bottom flask equipped with stirrer bar was added 1,3,5-tribromobenzene (2.88 g, 9.14 mmol, 1.0 eq.), copper iodide (350 mg, 1.84 mmol, 0.2 eq.), triphenylphosphine (482 mg, 1.84 mmol, 0.2 eq.), and 4-ethynylbenzaldehyde (5.00 g, 38.39 mmol, 4.2 eq.). The flask was evacuated under vacuum for 10 min, before being refilled with N_2. Triethylamine (200 mL) was added and the mixture degassed (N_2 bubbling, 20 mins) prior to the addition of $PdCl_2(PPh_3)_2$ (642 mg, 0.914 mmol, 0.1 eq.). The resulting suspension was heated at reflux overnight before being allowed to cool to room temperature. The reaction was diluted with water (300 mL) and the precipitated solid collected by filtration, washed with water, and dried on the filter. The solid was triturated in EtOAc (300 mL) and the resulting suspension filtered to collect the solid, which was further washed with EtOAc to yield the desired product **L** as a brown powder (3.89 g, 8.40 mmol, 92%).

IR (v_{max}/cm^{-1}) 2750 (C-H), 2610 (C≡C), 2340, 1690 (C=O), 1600 (ArC-ArC); **^{1}H NMR** (400 MHz, $CDCl_3$) δ_H 10.05 (s, 3H), 7.90 (d, *J* = 8.1 Hz, 6H), 7.74 (s, 3H), 7.75 (d, *J* = 8.1 Hz, 6H); **^{13}C NMR** (101 MHz, $CDCl_3$) $\delta_{C,}$ 192.3, 137.0, 134.0, 132.2, 129.9, 128.5, 123.8, 91.3, 90.1; **HRMS** (ESI+) calculated for $C_{33}H_{18}O_3$ 461.1261, found 263.0992 $[M+CH_3OH+H]^{2+}$. Data in accordance with literature values.[6]

5'-(3-Formylphenyl)-[1,1':3',1''-terphenyl]-3,3''-dicarbaldehyde, M

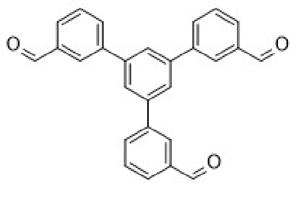

A modification of the procedure of Greenaway *et al.* was used for this reaction.[6] To an oven-dried round bottomed flask equipped with stirrer bar was added 1,3,5-tribromobenzene (2.04 g, 6.48 mmol, 1.0 eq.) and 3-formylphenylboronic acid (3.50 g, 23.33 mmol, 3.6 eq.) before the flask was evacuated and refilled with N_2 (×3). Isopropanol (40 mL) was added and the mixture degassed (N_2 bubbling, 15 mins) prior to the addition of $Pd(PPh_3)_4$ (374 mg, 0.324 mmol, 0.05 eq.). The resulting suspension was heated at 50 °C until all the solids had dissolved, at which point a 2 M aqueous $NaHCO_3$ solution (15 mL) was added. The resulting mixture was heated at reflux for 5 days before being allowed to cool to room temperature. The precipitated solid was collected by filtration and washed with water (30 mL) and Et_2O (60 mL) before being dissolved in $CHCl_3$ (150 mL). The resulting solution was dried ($MgSO_4$), filtered, and concentrated *in vacuo* to afford **M** as a light grey solid which was used without further purification (1.53 g, 3.92 mmol, 62%).

IR ($ν_{max}$/cm^{-1}) 3010 (ArC-ArC), 2720 (ArC-ArC), 2700 (ArC-ArC), 1695 (C=O), 1350 (O=C-H); **^{1}H NMR** (400 MHz, $CDCl_3$) $δ_H$ 10.15 (s, 3H), 8.03 (dq, *J* = 8, 3.9 Hz, 3H), 7.95 (dt, *J* = 8, 4.1 Hz, 3H), 7.89 (s, 3H), 7.70 (t, *J* = 8.1 Hz, 3H); **^{13}C NMR** (101 MHz, $CDCl_3$) $δ_C$ 125.7, 128.0, 129.5, 129.8, 133.3, 141.5, 192.2; **HRMS** (ESI+) calculated for $C_{27}H_{18}O_3$ 390.1256, found 391.2821 $[M+H]^+$. Data in accordance with literature values.[8]

5'-(2-Formylphenyl)-[1,1':3',1''-terphenyl]-2,2''-dicarbaldehyde, N

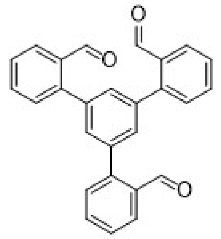

A modification of the procedure of Greenaway *et al.* was used for this reaction.[6] To an oven-dried round bottomed flask equipped with stirrer bar was added 1,3,5-tribromobenzene (2.04 g, 6.48 mmol, 1.0 eq.) and 2-formylphenylboronic acid (3.50 g, 23.33 mmol, 3.6 eq.) before the flask was evacuated and refilled with N_2 (×3). Isopropanol (40 mL) was added and the mixture degassed (N_2 bubbling, 15 mins) prior to the addition of $Pd(PPh_3)_4$ (374 mg, 0.324 mmol, 0.05 eq.). The resulting suspension was heated at 50 °C until all the solids had dissolved, at which point a 2 M aqueous $NaHCO_3$ solution (15 mL) was added. The resulting mixture was heated at reflux for 5 days before being allowed to cool to room temperature. The precipitated solid was collected by filtration and washed with water (30 mL) and Et_2O (60 mL) before being dissolved in $CHCl_3$ (150 mL). The resulting solution was dried ($MgSO_4$), filtered, and concentrated *in vacuo* to afford **N** as a grey solid which was used without further purification (1.86 g, 4.77 mmol, 74%).

IR ($ν_{max}$/cm^{-1}) 2805 (O=C-H), 1690 (C=O), 1180 (C-H) 720 (ArC-ArC); **^{1}H NMR** (400 MHz, $CDCl_3$) $δ_H$ 10.15 (s, 3H), 8.06 (dd, *J* = 8, 1.5 Hz, 3H), 7.70 (td, *J* = 7.2, 1.5 Hz, 3H), 7.55 (m, *J* = 8.2, 3H), 7.50 (s, 3H); **^{13}C NMR** (101 MHz, $CDCl_3$) $δ_C$ 128.6, 131.0, 133.8, 133.9, 138.6, 144.11, 191.7; **HRMS** (ESI+) calculated for $C_{27}H_{18}O_3$ 390.1256, found 391.1351 $[M+H]^+$. Data in accordance with literature values.[9]

4,4',4''-(Benzene-1,3,5-triyl)tris(thiophene-2-carbaldehyde), O

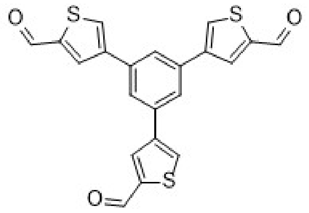

To an oven-dried round bottomed flask equipped with stirrer bar was added 1,3,5-tribromobenzene (1.96 g, 6.23 mmol, 1.0 eq.) and 2-formylthiophene-4-boronic acid (3.50 g, 22.44 mmol, 3.6 eq.) before the flask was evacuated and refilled with N_2 (×3). Isopropanol (40 mL) was added and the mixture degassed (N_2 bubbling, 15 mins) prior to the addition of $Pd(PPh_3)_4$ (358 mg, 0.31 mmol, 0.05 eq.). The resulting suspension was heated at 50 °C until all the solids had dissolved, at which point a 2 M aqueous $NaHCO_3$ solution (15 mL) was added. The resulting mixture was heated at reflux for 5 days before being allowed to cool to room temperature. The precipitated solid was collected by filtration and washed with water (30 mL) and Et_2O (60 mL) before being dissolved in $CHCl_3$ (150 mL). The resulting solution was dried ($MgSO_4$), filtered and concentrated *in vacuo* to afford **O** as a brown solid which was used without further purification (0.86 g, 2.73 mmol, 34%).

IR ($ν_{max}$/cm^{-1}) 3100 (C=C), 2820 (O=C-H), 1650 (C=O), 1570 (C-H); **^{1}H NMR** (400 MHz, $CDCl_3$) $δ_H$ 10.01 (app d, *J* = 1.3 Hz, 3H), 8.09 (app d, *J* = 1.6 Hz, 3H), 7.92 (app t, *J* = 1.4 Hz, 3H), 7.58 (br m, 3H); **^{13}C NMR** (101 MHz, $CDCl_3$) $δ_C$ 124.4, 126.0, 129.9, 130.0, 134.6, 135.4, 182.8; **HRMS** (ES+) calculated for $C_{21}H_{12}S_3O_3$ 407.9949, found 447.3459 $[M+K+H]^{2+}$.

5,5',5''-(Benzene-1,3,5-triyl)tris(furan-2-carbaldehyde), P

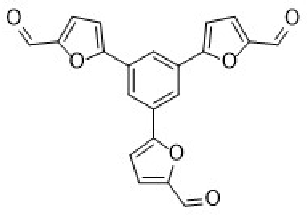

To an oven-dried round bottomed flask equipped with stirrer bar was added 1,3,5-tribromobenzene (3.645 g, 11.58 mmol, 1.0 eq.) and 2-formylfuran-5-boronic acid (5.83 g, 41.69 mmol, 3.6 eq.) before the flask was evacuated and refilled with N_2 (×3). Isopropanol (70 mL) was added and the mixture degassed (N_2 bubbling, 15 mins) prior to the addition of $Pd(PPh_3)_4$ (0.67 g, 0.58 mmol, 0.05 eq.). The resulting suspension was heated at 50 °C until all the solids had dissolved, at which point a 2 M aqueous $NaHCO_3$ solution (22 mL) was added. The resulting mixture was heated at reflux for 5 days before being allowed to cool to room temperature. The precipitated solid was collected by filtration and washed with water (50 mL) and Et_2O (100 mL) before being dissolved in $CHCl_3$ (250 mL). The resulting solution was dried ($MgSO_4$), filtered, and concentrated *in vacuo* to afford **P** as a colourless solid which was used without further purification (1.00 g, 2.78 mmol, 24%).

IR (ν_{max}/cm^{-1}) 1680 (C=O), 1500 (ArC-ArC), 1400 (ArC-O); **^{1}H NMR** (400 MHz, $CDCl_3$) δ_H 9.69 (s, 3H), 8.36 (s, 3H), 7.72 (m, 6H), 7.58 (dd, 6H); **^{13}C NMR** (101 MHz, $CDCl_3$) δ_C 177.5, 157.5, 152.7, 131.0, 123.5, 122.4, 109.4; **HRMS** (ESI+) calculated for $C_{21}H_{12}O_6$ 360.0634, found 361.0700 [M+H]$^+$. Data in accordance with literature values.[10]

1,3,5-Tris-(3-acrylaldehydebenzene)-benzene, Q

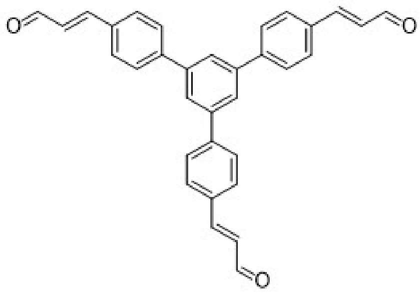

To an oven-dried round bottomed flask equipped with stirrer bar was added 1,3,5-phenyltriboronic acid tris(pinacol) ester (1.51 g, 3.31 mmol, 1.0 eq.) and 3-(4-bromophenyl)acrylaldehyde (2.50 g, 11.91 mmol, 3.6 eq.) before the flask was evacuated and refilled with N_2 (×3). Isopropanol (20 mL) was added and the mixture degassed (N_2 bubbling, 15 mins) prior to the addition of $Pd(PPh_3)_4$ (191 mg, 0.166 mmol, 0.05 eq.). The resulting suspension was heated at 50 °C until all the solids had dissolved, at which point a 2 M aqueous $NaHCO_3$ solution (6.2 mL) was added. The resulting mixture was heated at reflux for 5 days before being allowed to cool to room temperature. The precipitated solid was collected by filtration and washed with water (15 mL) and Et_2O (30 mL) before being dissolved in $CHCl_3$ (70 mL). The resulting solution was dried ($MgSO_4$), filtered, and concentrated *in vacuo*. The resulting solid was slurried in Et_2O (60 mL), stirred at room temperature overnight, collected by filtration and dried *in vacuo* to afford the desired product as a brown solid which was used without further purification (1.19 g, 2.54 mmol, 76%).

IR (ν_{max}/cm^{-1}) 1675(C=O), 1595(ArC-ArC), 1150(C-O), 9952, 810; **^{1}H NMR** (400 MHz, $CDCl_3$) δ_H 9.77 (d, 3H), 7.86 (s, 3H), 7.72 (d, 6H), 7.70 (d, 6H), 7.54 (d, 3H), 6.81 (q, 3H); **^{13}C NMR** (101 MHz, $CDCl_3$) $\delta_{C,}$ 193.6, 151.9, 143.4, 141.6, 133.6, 129.2, 128.8, 127.9, 125.5; **HRMS** (ESI+) calculated for $C_{33}H_{24}O_3$ 468.1725, found 469.1762 [M+H]$^+$.

4,4'',4'''-((Benzene-1,3,5-triyltris(methylene))tris(oxy))tribenzaldehyde, R

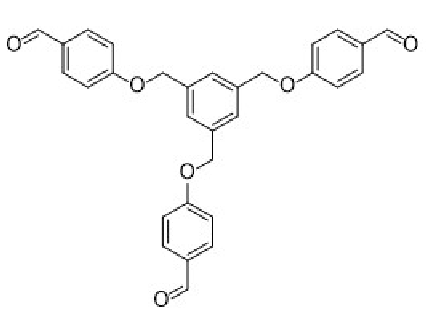

To a stirred solution of 1,3,5-tris(bromomethyl) benzene (500 mg, 1.40 mmol, 1.0 eq.) in THF (25 mL) was added 4-hydroxybenzaldehyde (546 mg, 4.47 mmol, 3.2 eq.) and anhydrous K_2CO_3 (1.36 g, 9.84 mmol, 7 eq.). The mixture was refluxed at 70 °C for 24 h before being allowed to cool to room temperature. The mixture was concentrated *in vacuo* and partitioned between saturated aqueous $NaHCO_3$ solution and $CHCl_3$ (3 x 25 mL). The resulting combined $CHCl_3$ layers were dried ($MgSO_4$), filtered, and concentrated *in vacuo* to afford the desired product as a colourless solid which was used without further purification (547 mg, 1.14 mmol, 81%).

IR (ν_{max}/cm^{-1}): 1685 (C=O), 1583 (ArC-ArC), 1250 (ArC-O), 1213 (ArC-O), 1156 (C-O); **^{1}H NMR** (400 MHz, $CDCl_3$) δ_H 9.89 (s, 3H), 7.84 (d, 6H), 7.50 (s, 3H), 7.07 (d, 6H), 5.19 (s, 6H); **^{13}C NMR** (101 MHz, $CDCl_3$) δ_C 190.78, 163.43, 137.31, 132.06, 130.33, 126.24, 115.13, 69.74; **HRMS** (ES-) calculated for $C_{30}H_{24}O_6$ 480.1573, found [M+Cl]$^-$ 515.1275. Data in accordance with literature values.[11]

5-((4-Formylphenyl)ethynyl)isophthalaldehyde, S

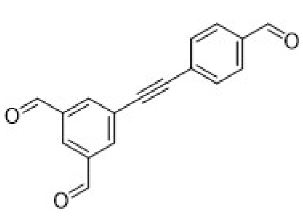

S had been previously synthesised by Berardo *et al.* and was used directly in this work.[12]

4,4',4''-((1*E*,1'1*E*,1''*E*)-Benzene-1,3,5-triyltris(ethene-2,1-diyl))tribenzaldehyde, U

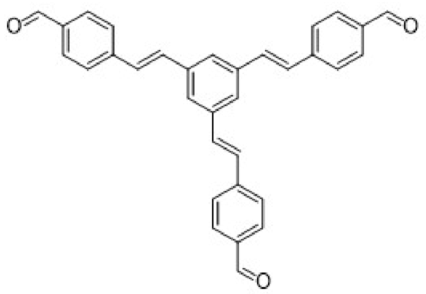

To an oven-dried and degassed round-bottomed flask equipped with stirrer bar was added 1,3,5-tris(bromomethyl)benzene (2.00 g, 5.61 mmol, 1.0 eq.) and triethyl phosphite (12 mL, 69.97 mmol, 2.5 eq.). The resulting solution was stirred at 150 °C under N_2 atmosphere. After cooling to room temperature, excess triethyl phosphite was removed *in vacuo* to afford a viscous orange oil. The mixture was cooled (0 °C) using an ice bath followed by the addition of 4-(diethoxymethyl)benzaldehyde (3.50 g, 16.81 mmol, 3.0 eq.). To the resulting solution was slowly added KOtBu (5.66 g, 50.50 mmol, 9.0 eq.) under N_2 atmosphere at 0 °C. The mixture was stirred for 12 h at room temperature. To the resulting mixture was added 1M aq. HCl (40 mL) and the reaction stirred for an additional 20 minutes at room temperature. The resulting mixture was extracted with DCM (3 x 30 mL) and washed with brine (2 x 30 mL), before being dried with $MgSO_4$, filtered and concentrated *in vacuo* to afford a yellow solid. The crude product was recrystallised using 1:1 $CHCl_3$:MeOH (140 mL), before being dried in a vacuum oven (50 °C) to yield the pure product as a yellow solid (2.02 g, 4.31 mmol, 77%).

IR (ν_{max}/cm^{-1}): 1687 (C=O), 1590 (C=C), 1160 (ArC-ArC); **^{1}H NMR** (400 MHz, $(CD_3)_2SO$) δ_H 10.02 (s, 3H), 7.98-7.87 (m, 15H), 7.84 (s, 6H); **^{13}C NMR** (101 MHz, $(CD_3)_2SO$) δ_C 197.71, 148.16, 142.79, 140.45,

136.54, 135.36, 133.56, 132.32, 130.57; **HRMS** (ES-) calculated for $C_{33}H_{24}O_3$ 468.1730, found $[M+Cl]^-$ 503.1422. Data in accordance with literature values.[13]

S3.2 Spectra

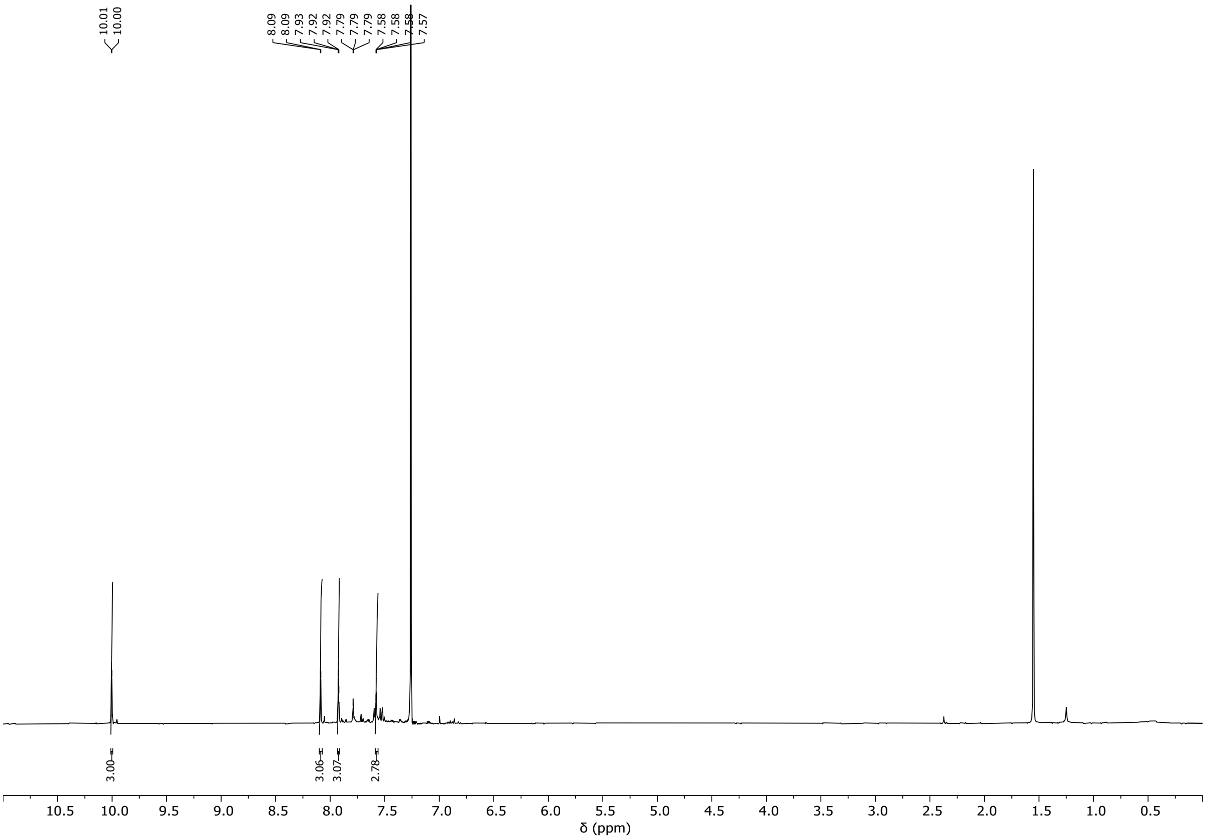

Figure S1: ^{1}H NMR ($CDCl_3$) spectrum of tri-topic precursor **O**.

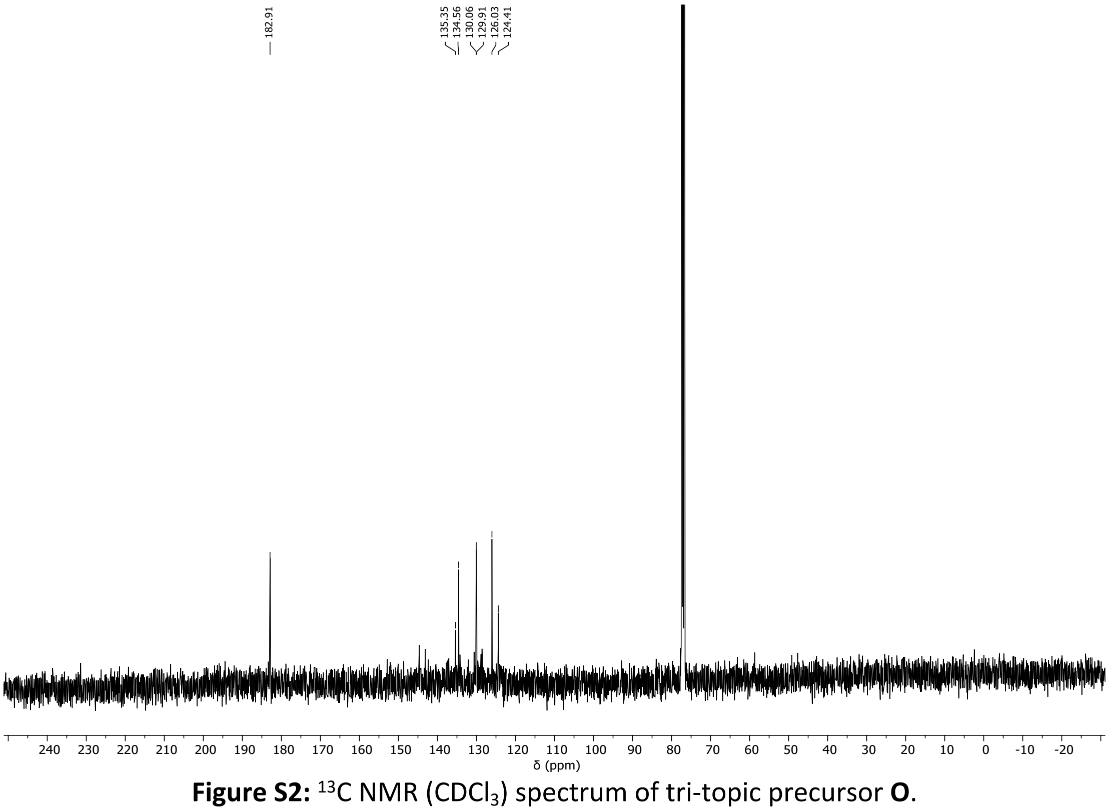

Figure S2: ^{13}C NMR ($CDCl_3$) spectrum of tri-topic precursor **O**.

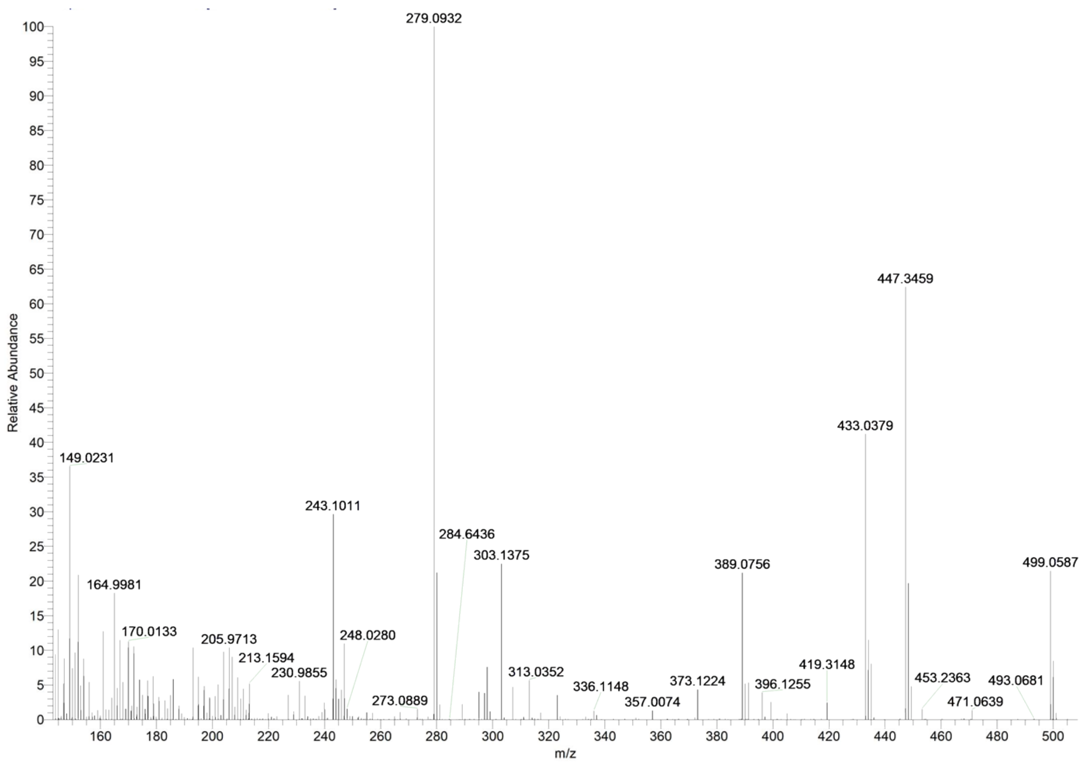

Figure S3: HRMS ESI(+) of precursor **O**, calculated for $C_{21}H_{12}S_3O_3$ 407.9949, found 447.3459 $[M+K+H]^{2+}$

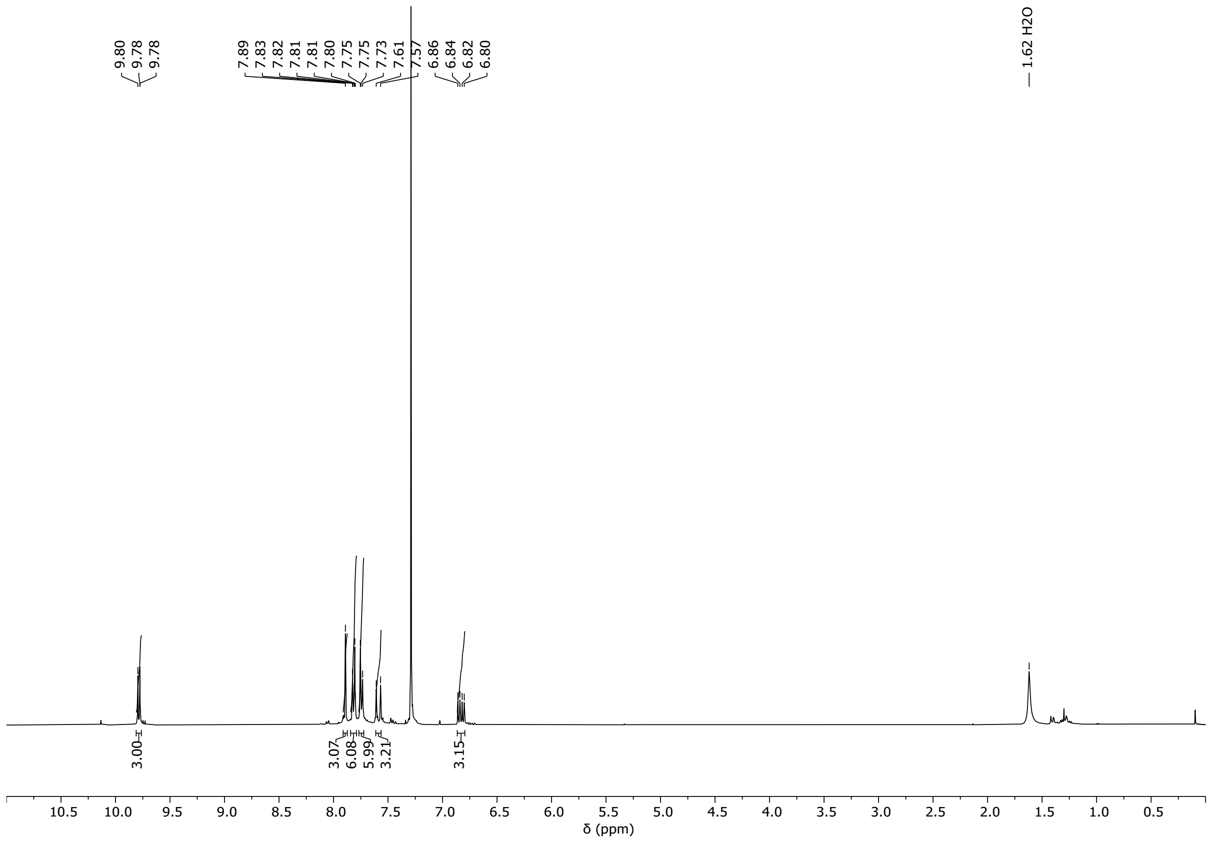

Figure S4: ^{1}H NMR ($CDCl_3$) spectrum of tri-topic precursor **Q**.

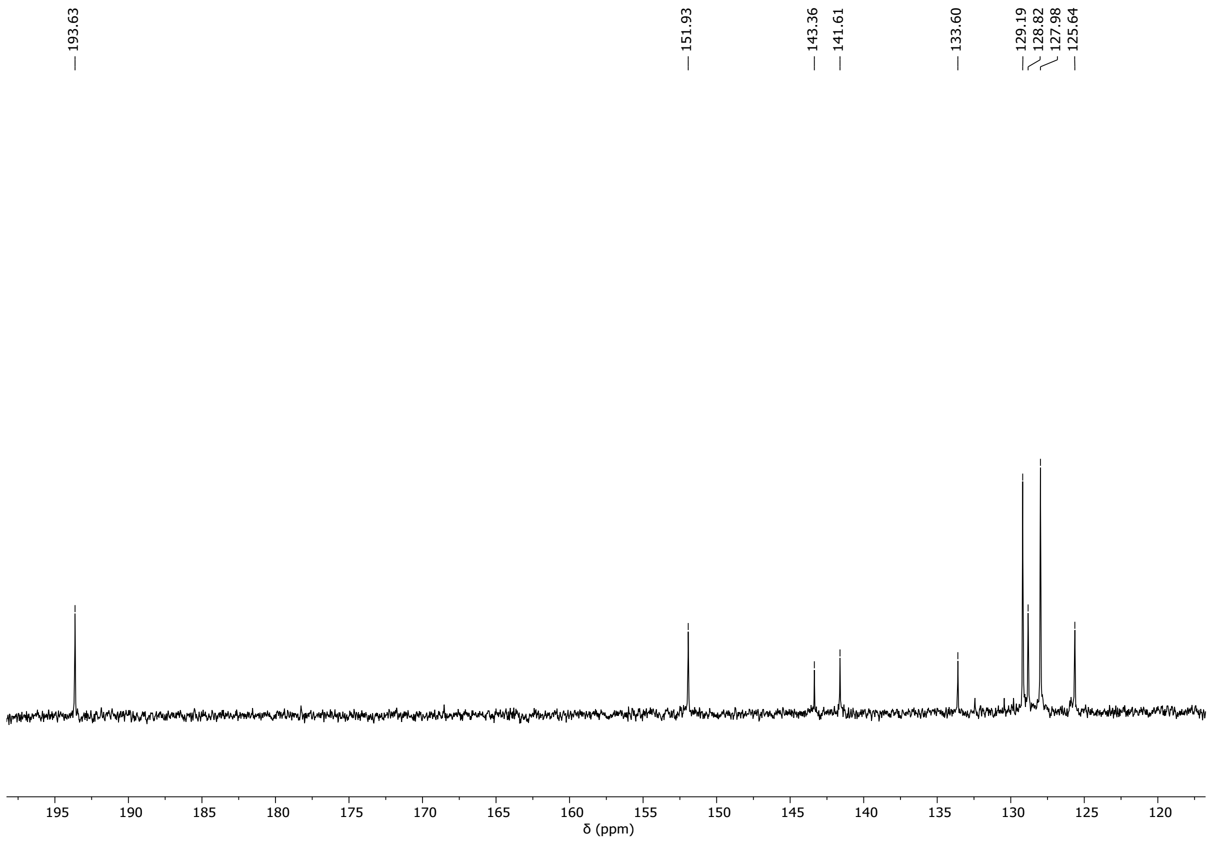

Figure S5: ^{13}C NMR spectrum of tri-topic precursor **Q**.

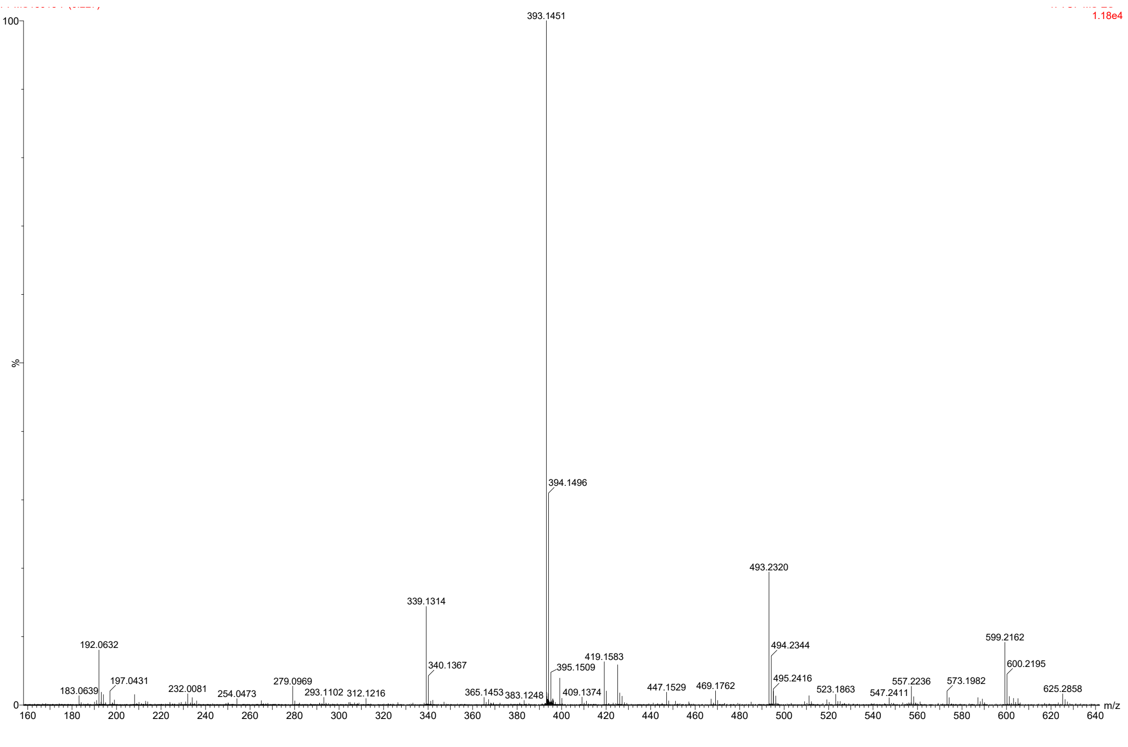

Figure S6: HRMS ESI(+) of precursor **Q**, calculated for $C_{33}H_{24}O_3$ 468.1725, found 469.1762 $[M+H]^+$

S4. Experimental High-throughput Workflow

Reaction condition screening: To investigate the transferability of our previously optimised high-throughput reaction conditions,[6] a small screen was carried out using the precursor combinations of trialdehyde **G** and diamine **24**, and triamine **B** and dialdehyde **4**, both selected from the literature (**CC3**[14] and **B11**,[6] respectively, with the latter also synthesized in our previous high-throughput workflow). Conditions were screened offline using stock solutions to mimic the high-throughput approach and assessed for turbidity, conversion (^1H NMR) and species formation (HRMS). A representative example is shown for **G24** below:

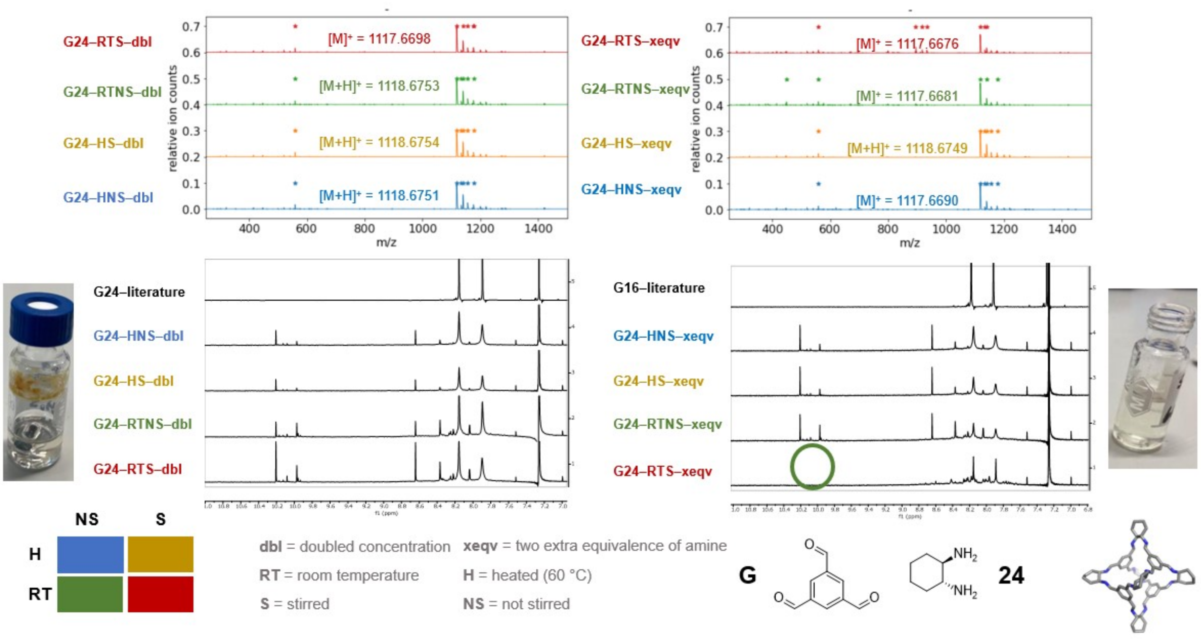

Using chloroform as the reaction solvent, a slightly increased concentration was investigated (0.0092 M vs 0.0184 M, left, to try and increase the quantity of material synthesized for characterization due to the desired miniaturization of the reaction screens, while minimizing precipitate formation), alongside the reaction temperature (room temperature (green/red) vs heating at 60 °C (blue/yellow), with the latter employed in our previous high-throughput workflow), and precursor stoichiometric ratio (right, as previously we had found an excess of amine was required to increase conversion and was well tolerated compared to an excess of aldehyde). In addition, the requirement for stirring was investigated. The desired **Tri⁴Di⁶** mass ion peaks for **CC3** were identified across all conditions screened (top), but full conversion was only observed when two extra equivalences of diamine **24** were used, at room temperature and with stirring (bottom, shown in the green circle by absence of residual aldehyde **G** peak). These reaction conditions were also successful for **B4**.

Due to the additional 2 equivalents of amine precursor required for cage formation, and using the **Tri^{4}Di^6** stoichiometric ratio, the resulting precursor ratios for the high-throughput screen were therefore 4:8 for trialdehydes:diamines and 6:6 for triamines:dialdehydes.

High-throughput screen general method: High-throughput experimental screening was conducted using the Opentrons OT-2 liquid handling platform (robot v6.3.1 and 6.3.1 app version), with settings based on the optimisation and calibration of the system with the bulk reaction solvent (in this case, chloroform) used for screening (Table S2 and Figure S7 - gantry speed of 250 mms^{-1}, and plunger flow rates (for aspirating and dispensing) of 70 μLs^{-1} using the 300 µL Opentrons proprietary pipette. Before transferring a new substance stock solution, 100 µL of the solution was aspirated into the pipette which was then lifted to the top of the vial, followed by a 10 s delay, and dispensed back into the same vial. This was done to pre-saturate and swell the pipette tip, which was found to reduce the dripping during transfer across the deck. A maximum volume of 230 µL of a stock solution was transferred at a time, using a 15 µL air gap. Where more than 225 µL of a substance was required, multiple transfers were made. Protocols were written using OT-2 Python Protocol API Version 2.9 and simulated prior to conducting each high-throughput run using the Opentrons Python package - the code required to replicate the protocol is available at https://github.com/GreenawayLab/Streamlining-Automated-Discovery-POCs/tree/main/Opentrons_Protocols. Following these settings, the OT-2 transferred volumes of the tri-topic stock solutions, followed by volumes of the di-topic stock solutions, from a 24-well plate and topped up to a total volume of 1 mL with $CHCl_3$ from a 6-well solvent plate (Figure S8) – stock solution concentrations, reaction ratios and stock solution volumes are found in Tables S3, S4 and S5. Reactions were stirred at room temperature for 5 days and then directly analysed via computer vision turbidity methods (see Section S5.1). Following this, and again using the OT-2 platform, 20 µL of each reaction sample was transferred to 2 mL sample vials in a 54-well plate and diluted to 1 mL with 1:1 DCM:MeOH and HRMS analysis carried out (see Section S5.3). The bulk reaction samples were then placed in a 48-well EquaVAP parallel solvent evaporator before being returned to the OT-2 deck and redissolved in 700 µL $CDCl_3$ - 600 µL of this solution was then transferred into a 3D printed 96-well NMR tube holder plate (https://github.com/GreenawayLab/Streamlining-Automated-Discovery-POCs/tree/main/3D-printing) and ^1H NMR analysis undertaken (see Section S5.2).

The raw turbidity, HRMS and ^1H NMR files can be found on Zenodo (https://doi.org/10.5281/zenodo.10675206), with a key mapping each precursor combination to each data file path via experiment code, plate number and formulation number available on GitHub (https://github.com/GreenawayLab/Streamlining-Automated-Discovery-POCs).

Table S2: Final calibration of Opentrons OT-2 with chloroform across volumes 20 – 230 µL. Dispenses were carried out three times per targeted volume and averaged. Gantry speeds were set to 250 mm s^{-1} for the X, Y and Z axes. The 300 µL pipette aspiration and dispense flowrate was set to 70 µL s^{-1} and transfers included a pre-saturation step (100 µL aspiration, 10 s delay, 100 µL dispense back into the source vial) to prevent dripping and allow accurate transfers. Vials were pre-weighed before the dispense and after, with the actual dispensed volume calculated using the density of chloroform and the accuracy against the target volume calculated.

Target Dispense Volume / µL	Average Dispensed Volume / µL	Δvol / µL	Standard deviation
0	0	0	0.00
20	14	-6	0.34
40	30	-10	1.04
60	53	-7	1.24
80	72	-8	3.43
100	93	-7	0.17
120	110	-10	0.30
140	133	-7	3.56
160	153	-7	0.13
180	175	-5	5.10
190	184	-6	6.98
200	191	-9	3.06
205	202	-3	7.42
210	201	-9	2.82
220	217	-3	9.74
225	225	0	8.76
230	229	-1	10.24

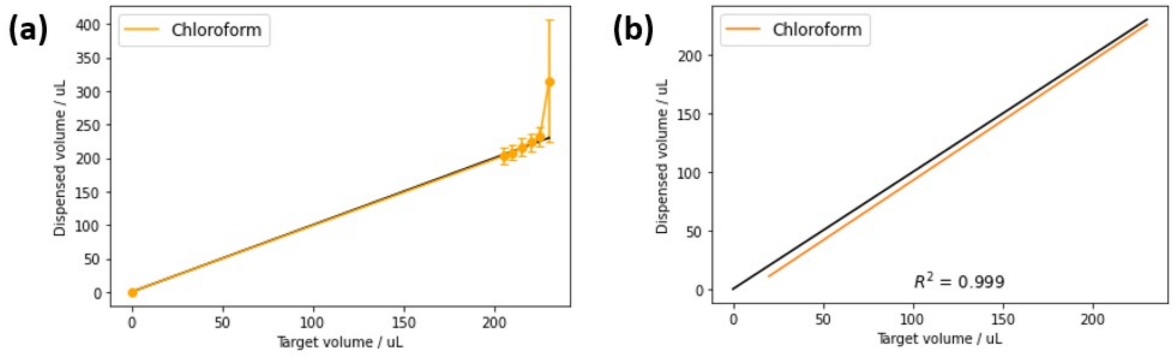

Figure S7: Calibration of the Opentrons OT-2 with chloroform (orange) compared to the targeted dispense volumes (black). (a) Dispenses were measured and averaged up to 250 µL with error bars included showing the standard deviation, showing a large increase in the dispense accuracy and precision at 250 µL; (b) Dispenses are capped at 230 µL to ensure accuracy, resulting in an R^2 value of 0.999 – higher dispenses above this maximum volume led to increased error in the dispensed volume.

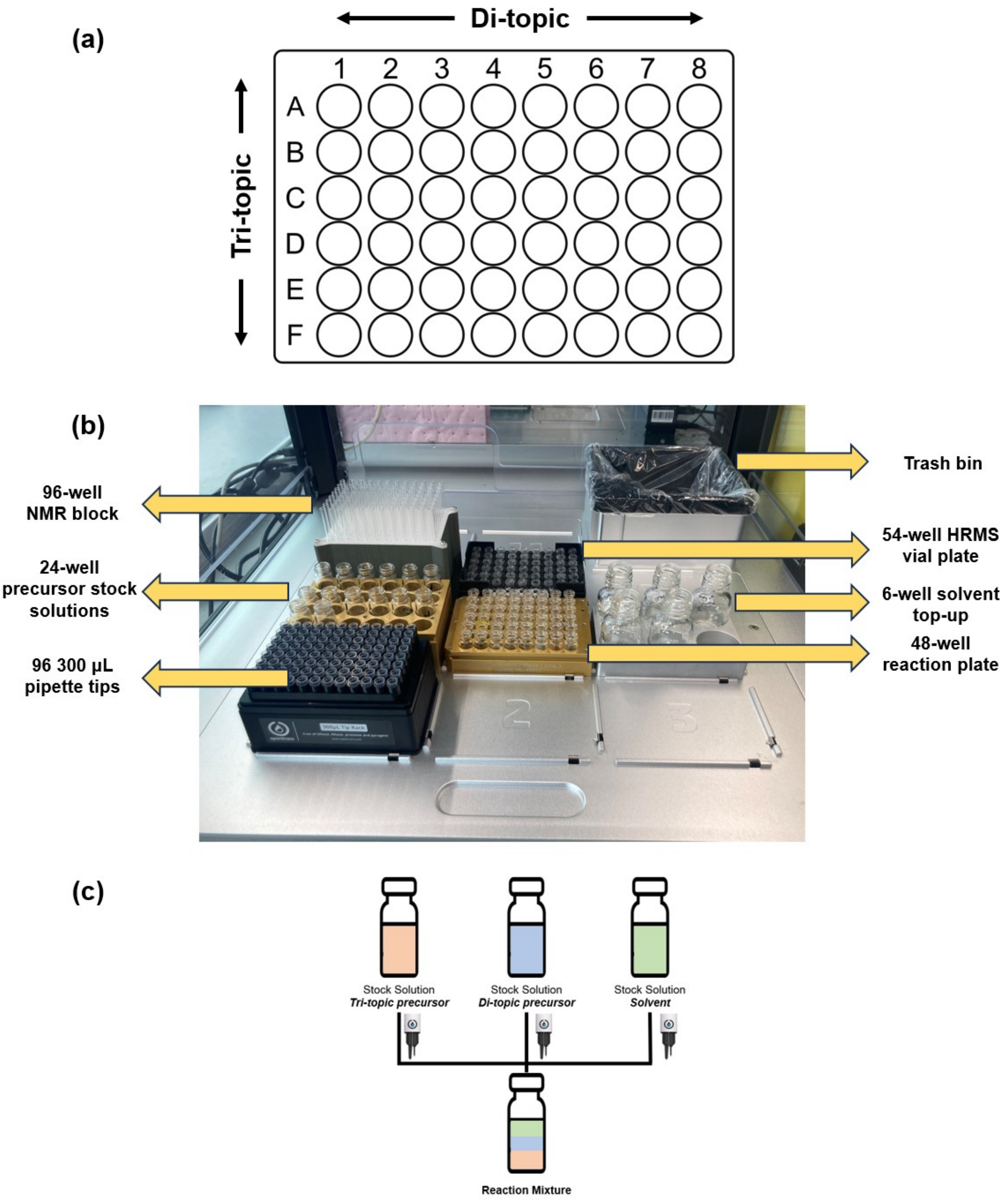

Figure S8: (a) Exemplar reaction plate (48-well) where each row is a different tri-topic precursor (trialdehydes or triamines, labelled as letters **A-U**) screened against each column containing different di-topic precursors (dialdehydes or diamines, labelled as numbers **1-34**); (b) General Opentrons OT-2 deck layout used for the high-throughput screen and sample preparation for analysis; (c) A 300 µL pipette was used for liquid transfers from the 24-well stock solution plates of the tri- and di-topic precursors into the 48-well reaction plate, with each reaction combination then topped up to the same volume and overall concentration using the 6-well solvent top-up plate.

Table S3: Precursor stock solutions in $CHCl_3$ for high-throughput screening.

Precursor	Molecular weight / g mol^{-1}	Stock Solution concentration / mg mL^{-1}	Stock Solution concentration / mmol mL^{-1}
**A**	126.12	5	0.0396
**B**	207.32	5	0.0241
**C**	146.23	5	0.0342
**D**	353.78	6	0.0170
**E**	188.31	5	0.0266
**F***	129.21	5	0.0387
**G**	162.14	5	0.0396
**H**	390.40	5	0.1542
**I**	178.14	5	0.0640
**J**	329.35	5	0.1403
**K**	393.39	5	0.0759
**L**	462.50	5	0.0108
**M**	390.44	5	0.0128
**N**	390.44	5	0.0128
**O**	408.50	5	0.0122
**P**	360.32	5	0.0139
**Q**	468.55	5	0.0107
**R**	432.52	5	0.0520
**S**	262.26	5	0.0191
**T**	210.14	5	0.0238
**U**	468.55	5	0.0107
**1**	134.13	5	0.0373
**2**	206.24	5	0.0242
**3**	196.25	5	0.0255
**4**	134.13	5	0.0373
**5**	206.09	5	0.0243
**6**	210.23	5	0.0238
**7**	234.25	5	0.0213
**8**	236.22	5	0.0212
**9**	140.16	5	0.0357
**10**	134.13	5	0.0373
**11**	214.33	5	0.0233
**12**	214.26	5	0.0233
**13**	330.34	5	0.0151
**14**	188.19	5	0.0266

**15**	284.31	5	0.0176
**16**	292.38	5	0.0171
**17**	60.1	5	0.0832
**18**	102.18	5	0.0489
**19**	191.27	5	0.0261
**20**	212.29	5	0.0236
**21**	200.34	5	0.0250
**22**	100.08	5	0.0500
**23**	114.19	5	0.0438
**24**	114.19	5	0.0438
**25**	215.38	5	0.1161
**26**	116.21	5	0.2151
**27**	136.2	5	0.1836
**28**	142.25	5	0.1757
**29**	145.25	5	0.1721
**30**	104.15	5	0.2400
**31**	136.2	5	0.1836
**32**	90.13	5	0.2774
**33**	146.19	5	0.1710
**34***	114.19	5	0.2189

* Precursors **F** and **34** were trihydrochloride salt derivatives and therefore triethylamine (4 equiv.) was added to each of these stock solutions.

Table S4: Stock solution and solvent volumes used in each reaction on the Opentrons OT-2 platform for 6 triamines screened against 16 dialdehydes.

Cage	Triamine	Volume triamine stock solution (mL)	Amount of triamine (mmol)	Dialdehyde	Ratio of equiv. used	Amount of dialdehyde (mmol)	Volume dialdehyde stock solution (mL)	Volume of solvent ($CHCl_3$) top-up (total = 1 mL)
**A1**	**A**	0.120	0.0048	**1**	6:6	0.0048	0.128	0.752
**A2**	**A**	0.120	0.0048	**2**	6:6	0.0048	0.196	0.684
**A3**	**A**	0.120	0.0048	**3**	6:6	0.0048	0.187	0.693
**A4**	**A**	0.120	0.0048	**4**	6:6	0.0048	0.128	0.752
**A5**	**A**	0.120	0.0048	**5**	6:6	0.0048	0.196	0.684
**A6**	**A**	0.120	0.0048	**6**	6:6	0.0048	0.200	0.680
**A7**	**A**	0.120	0.0048	**7**	6:6	0.0048	0.223	0.657
**A8**	**A**	0.120	0.0048	**8**	6:6	0.0048	0.225	0.655
**A9**	**A**	0.120	0.0048	**9**	6:6	0.0048	0.133	0.747
**A10**	**A**	0.120	0.0048	**10**	6:6	0.0048	0.128	0.752
**A11**	**A**	0.120	0.0048	**11**	6:6	0.0048	0.204	0.676
**A12**	**A**	0.120	0.0048	**12**	6:6	0.0048	0.204	0.676
**A13**	**A**	0.120	0.0048	**13**	6:6	0.0048	0.314	0.566
**A14**	**A**	0.120	0.0048	**14**	6:6	0.0048	0.179	0.701
**A15**	**A**	0.120	0.0048	**15**	6:6	0.0048	0.271	0.609
**A16**	**A**	0.120	0.0048	**16**	6:6	0.0048	0.278	0.602
**B1**	**B**	0.200	0.0048	**1**	6:6	0.0048	0.128	0.671
**B2**	**B**	0.200	0.0048	**2**	6:6	0.0048	0.196	0.601
**B3**	**B**	0.200	0.0048	**3**	6:6	0.0048	0.187	0.611
**B4**	**B**	0.200	0.0048	**4**	6:6	0.0048	0.128	0.671
**B5**	**B**	0.200	0.0048	**5**	6:6	0.0048	0.196	0.601
**B6**	**B**	0.200	0.0048	**6**	6:6	0.0048	0.200	0.597
**B7**	**B**	0.200	0.0048	**7**	6:6	0.0048	0.223	0.574
**B8**	**B**	0.200	0.0048	**8**	6:6	0.0048	0.225	0.572
**B9**	**B**	0.200	0.0048	**9**	6:6	0.0048	0.135	0.665
**B10**	**B**	0.200	0.0048	**10**	6:6	0.0048	0.129	0.671
**B11**	**B**	0.200	0.0048	**11**	6:6	0.0048	0.207	0.593
**B12**	**B**	0.200	0.0048	**12**	6:6	0.0048	0.207	0.593
**B13**	**B**	0.200	0.0048	**13**	6:6	0.0048	0.319	0.481
**B14**	**B**	0.200	0.0048	**14**	6:6	0.0048	0.182	0.618
**B15**	**B**	0.200	0.0048	**15**	6:6	0.0048	0.274	0.526
**B16**	**B**	0.200	0.0048	**16**	6:6	0.0048	0.282	0.518

**C1**	**C**	0.140	0.0048	**1**	6:6	0.0048	0.128	0.732
**C2**	**C**	0.140	0.0048	**2**	6:6	0.0048	0.196	0.663
**C3**	**C**	0.140	0.0048	**3**	6:6	0.0048	0.187	0.672
**C4**	**C**	0.140	0.0048	**4**	6:6	0.0048	0.128	0.732
**C5**	**C**	0.140	0.0048	**5**	6:6	0.0048	0.196	0.663
**C6**	**C**	0.140	0.0048	**6**	6:6	0.0048	0.200	0.659
**C7**	**C**	0.140	0.0048	**7**	6:6	0.0048	0.223	0.636
**C8**	**C**	0.140	0.0048	**8**	6:6	0.0048	0.225	0.634
**C9**	**C**	0.140	0.0048	**9**	6:6	0.0048	0.134	0.726
**C10**	**C**	0.140	0.0048	**10**	6:6	0.0048	0.128	0.732
**C11**	**C**	0.140	0.0048	**11**	6:6	0.0048	0.205	0.655
**C12**	**C**	0.140	0.0048	**12**	6:6	0.0048	0.205	0.655
**C13**	**C**	0.140	0.0048	**13**	6:6	0.0048	0.316	0.544
**C14**	**C**	0.140	0.0048	**14**	6:6	0.0048	0.180	0.680
**C15**	**C**	0.140	0.0048	**15**	6:6	0.0048	0.272	0.588
**C16**	**C**	0.140	0.0048	**16**	6:6	0.0048	0.280	0.580
**D1**	**D**	0.285	0.0048	**1**	6:6	0.0048	0.128	0.585
**D2**	**D**	0.285	0.0048	**2**	6:6	0.0048	0.196	0.516
**D3**	**D**	0.285	0.0048	**3**	6:6	0.0048	0.187	0.525
**D4**	**D**	0.285	0.0048	**4**	6:6	0.0048	0.128	0.585
**D5**	**D**	0.285	0.0048	**5**	6:6	0.0048	0.196	0.516
**D6**	**D**	0.285	0.0048	**6**	6:6	0.0048	0.200	0.512
**D7**	**D**	0.285	0.0048	**7**	6:6	0.0048	0.223	0.489
**D8**	**D**	0.285	0.0048	**8**	6:6	0.0048	0.225	0.487
**D9**	**D**	0.285	0.0048	**9**	6:6	0.0048	0.135	0.580
**D10**	**D**	0.285	0.0048	**10**	6:6	0.0048	0.130	0.585
**D11**	**D**	0.285	0.0048	**11**	6:6	0.0048	0.207	0.508
**D12**	**D**	0.285	0.0048	**12**	6:6	0.0048	0.207	0.508
**D13**	**D**	0.285	0.0048	**13**	6:6	0.0048	0.319	0.396
**D14**	**D**	0.285	0.0048	**14**	6:6	0.0048	0.182	0.533
**D15**	**D**	0.285	0.0048	**15**	6:6	0.0048	0.275	0.440
**D16**	**D**	0.285	0.0048	**16**	6:6	0.0048	0.283	0.432
**E1**	**E**	0.180	0.0048	**1**	6:6	0.0048	0.128	0.692
**E2**	**E**	0.180	0.0048	**2**	6:6	0.0048	0.196	0.623
**E3**	**E**	0.180	0.0048	**3**	6:6	0.0048	0.187	0.632
**E4**	**E**	0.180	0.0048	**4**	6:6	0.0048	0.128	0.692
**E5**	**E**	0.180	0.0048	**5**	6:6	0.0048	0.196	0.623
**E6**	**E**	0.180	0.0048	**6**	6:6	0.0048	0.200	0.619

**E7**	**E**	0.180	0.0048	**7**	6:6	0.0048	0.223	0.596
**E8**	**E**	0.180	0.0048	**8**	6:6	0.0048	0.225	0.594
**E9**	**E**	0.180	0.0048	**9**	6:6	0.0048	0.134	0.686
**E10**	**E**	0.180	0.0048	**10**	6:6	0.0048	0.128	0.692
**E11**	**E**	0.180	0.0048	**11**	6:6	0.0048	0.205	0.615
**E12**	**E**	0.180	0.0048	**12**	6:6	0.0048	0.205	0.615
**E13**	**E**	0.180	0.0048	**13**	6:6	0.0048	0.316	0.504
**E14**	**E**	0.180	0.0048	**14**	6:6	0.0048	0.180	0.640
**E15**	**E**	0.180	0.0048	**15**	6:6	0.0048	0.272	0.548
**E16**	**E**	0.180	0.0048	**16**	6:6	0.0048	0.279	0.541
**F1**	**F**	0.125	0.0048	**1**	6:6	0.0048	0.128	0.745
**F2**	**F**	0.125	0.0048	**2**	6:6	0.0048	0.196	0.675
**F3**	**F**	0.125	0.0048	**3**	6:6	0.0048	0.187	0.685
**F4**	**F**	0.125	0.0048	**4**	6:6	0.0048	0.128	0.745
**F5**	**F**	0.125	0.0048	**5**	6:6	0.0048	0.196	0.676
**F6**	**F**	0.125	0.0048	**6**	6:6	0.0048	0.200	0.672
**F7**	**F**	0.125	0.0048	**7**	6:6	0.0048	0.223	0.648
**F8**	**F**	0.125	0.0048	**8**	6:6	0.0048	0.225	0.646
**F9**	**F**	0.125	0.0048	**9**	6:6	0.0048	0.136	0.739
**F10**	**F**	0.125	0.0048	**10**	6:6	0.0048	0.130	0.745
**F11**	**F**	0.125	0.0048	**11**	6:6	0.0048	0.207	0.668
**F12**	**F**	0.125	0.0048	**12**	6:6	0.0048	0.207	0.668
**F13**	**F**	0.125	0.0048	**13**	6:6	0.0048	0.320	0.555
**F14**	**F**	0.125	0.0048	**14**	6:6	0.0048	0.182	0.693
**F15**	**F**	0.125	0.0048	**15**	6:6	0.0048	0.275	0.600
**F16**	**F**	0.125	0.0048	**16**	6:6	0.0048	0.283	0.592

Table S5: Stock solution and solvent volumes used in each reaction on the Opentrons OT-2 platform for 15 trialdehydes screened against 18 diamines.

Cage	Trialdehyde	Volume trialdehyde stock solution (mL)	Amount of trialdehyde (mmol)	Diamine	Ratio of equiv. used	Amount of diamine (mmol)	Volume diamine stock solution (mL)	Volume of solvent ($CHCl_3$) top-up (total = 1 mL)
**G17**	**G**	0.100	0.0031	**17**	4:8	0.0062	0.074	0.826
**G18**	**G**	0.100	0.0031	**18**	4:8	0.0062	0.126	0.774
**G19**	**G**	0.100	0.0031	**19**	4:8	0.0062	0.236	0.664
**G20**	**G**	0.100	0.0031	**20**	4:8	0.0062	0.262	0.638
**G21**	**G**	0.100	0.0031	**21**	4:8	0.0062	0.247	0.653
**G22**	**G**	0.100	0.0031	**22**	4:8	0.0062	0.123	0.777
**G23**	**G**	0.100	0.0031	**23**	4:8	0.0062	0.141	0.759
**G24**	**G**	0.100	0.0031	**24**	4:8	0.0062	0.141	0.759
**G25**	**G**	0.100	0.0031	**25**	4:8	0.0062	0.268	0.631
**G26**	**G**	0.100	0.0031	**26**	4:8	0.0062	0.145	0.754
**G27**	**G**	0.100	0.0031	**27**	4:8	0.0062	0.171	0.727
**G28**	**G**	0.100	0.0031	**28**	4:8	0.0062	0.179	0.719
**G29**	**G**	0.100	0.0031	**29**	4:8	0.0062	0.183	0.715
**G30**	**G**	0.100	0.0031	**30**	4:8	0.0062	0.131	0.767
**G31**	**G**	0.100	0.0031	**31**	4:8	0.0062	0.171	0.727
**G32**	**G**	0.100	0.0031	**32**	4:8	0.0062	0.113	0.785
**G33**	**G**	0.100	0.0031	**33**	4:8	0.0062	0.184	0.714
**G34**	**G**	0.100	0.0031	**34**	4:8	0.0062	0.144	0.754
**H17**	**H**	0.250	0.0031	**17**	4:8	0.0062	0.074	0.686
**H18**	**H**	0.250	0.0031	**18**	4:8	0.0062	0.126	0.634
**H19**	**H**	0.250	0.0031	**19**	4:8	0.0062	0.235	0.525
**H20**	**H**	0.250	0.0031	**20**	4:8	0.0062	0.261	0.499
**H21**	**H**	0.250	0.0031	**21**	4:8	0.0062	0.246	0.514
**H22**	**H**	0.250	0.0031	**22**	4:8	0.0062	0.123	0.637
**H23**	**H**	0.250	0.0031	**23**	4:8	0.0062	0.140	0.620
**H24**	**H**	0.250	0.0031	**24**	4:8	0.0062	0.140	0.620
**H25**	**H**	0.250	0.0031	**25**	4:8	0.0062	0.265	0.485
**H26**	**H**	0.250	0.0031	**26**	4:8	0.0062	0.143	0.607
**H27**	**H**	0.250	0.0031	**27**	4:8	0.0062	0.171	0.584
**H28**	**H**	0.250	0.0031	**28**	4:8	0.0062	0.179	0.576
**H29**	**H**	0.250	0.0031	**29**	4:8	0.0062	0.182	0.573
**H30**	**H**	0.250	0.0031	**30**	4:8	0.0062	0.131	0.624
**H31**	**H**	0.250	0.0031	**31**	4:8	0.0062	0.171	0.584

**H32**	**H**	0.250	0.0031	**32**	4:8	0.0062	0.113	0.642
**H33**	**H**	0.250	0.0031	**33**	4:8	0.0062	0.183	0.572
**H34**	**H**	0.250	0.0031	**34**	4:8	0.0062	0.143	0.612
**I17**	**I**	0.110	0.0031	**17**	4:8	0.0062	0.074	0.816
**I18**	**I**	0.110	0.0031	**18**	4:8	0.0062	0.126	0.764
**I19**	**I**	0.110	0.0031	**19**	4:8	0.0062	0.236	0.654
**I20**	**I**	0.110	0.0031	**20**	4:8	0.0062	0.262	0.628
**I21**	**I**	0.110	0.0031	**21**	4:8	0.0062	0.247	0.643
**I22**	**I**	0.110	0.0031	**22**	4:8	0.0062	0.124	0.766
**I23**	**I**	0.110	0.0031	**23**	4:8	0.0062	0.141	0.749
**I24**	**I**	0.110	0.0031	**24**	4:8	0.0062	0.141	0.749
**I25**	**I**	0.110	0.0031	**25**	4:8	0.0062	0.264	0.627
**I26**	**I**	0.110	0.0031	**26**	4:8	0.0062	0.142	0.749
**I27**	**I**	0.110	0.0031	**27**	4:8	0.0062	0.171	0.717
**I28**	**I**	0.110	0.0031	**28**	4:8	0.0062	0.179	0.709
**I29**	**I**	0.110	0.0031	**29**	4:8	0.0062	0.183	0.705
**I30**	**I**	0.110	0.0031	**30**	4:8	0.0062	0.131	0.757
**I31**	**I**	0.110	0.0031	**31**	4:8	0.0062	0.171	0.717
**I32**	**I**	0.110	0.0031	**32**	4:8	0.0062	0.113	0.775
**I33**	**I**	0.110	0.0031	**33**	4:8	0.0062	0.184	0.704
**I34**	**I**	0.110	0.0031	**34**	4:8	0.0062	0.144	0.744
**J17**	**J**	0.205	0.0031	**17**	4:8	0.0062	0.075	0.720
**J18**	**J**	0.205	0.0031	**18**	4:8	0.0062	0.127	0.668
**J19**	**J**	0.205	0.0031	**19**	4:8	0.0062	0.238	0.557
**J20**	**J**	0.205	0.0031	**20**	4:8	0.0062	0.264	0.531
**J21**	**J**	0.205	0.0031	**21**	4:8	0.0062	0.249	0.546
**J22**	**J**	0.205	0.0031	**22**	4:8	0.0062	0.125	0.670
**J23**	**J**	0.205	0.0031	**23**	4:8	0.0062	0.142	0.653
**J24**	**J**	0.205	0.0031	**24**	4:8	0.0062	0.142	0.653
**J25**	**J**	0.205	0.0031	**25**	4:8	0.0062	0.271	0.522
**J26**	**J**	0.205	0.0031	**26**	4:8	0.0062	0.146	0.647
**J27**	**J**	0.205	0.0031	**27**	4:8	0.0062	0.171	0.622
**J28**	**J**	0.205	0.0031	**28**	4:8	0.0062	0.179	0.614
**J29**	**J**	0.205	0.0031	**29**	4:8	0.0062	0.183	0.610
**J30**	**J**	0.205	0.0031	**30**	4:8	0.0062	0.131	0.662
**J31**	**J**	0.205	0.0031	**31**	4:8	0.0062	0.171	0.622
**J32**	**J**	0.205	0.0031	**32**	4:8	0.0062	0.113	0.680
**J33**	**J**	0.205	0.0031	**33**	4:8	0.0062	0.184	0.609

**J34**	**J**	0.205	0.0031	**34**	4:8	0.0062	0.144	0.649
**K17**	**K**	0.247	0.0031	**17**	4:8	0.0062	0.075	0.635
**K18**	**K**	0.247	0.0031	**18**	4:8	0.0062	0.128	0.582
**K19**	**K**	0.247	0.0031	**19**	4:8	0.0062	0.240	0.470
**K20**	**K**	0.247	0.0031	**20**	4:8	0.0062	0.266	0.444
**K21**	**K**	0.247	0.0031	**21**	4:8	0.0062	0.251	0.459
**K22**	**K**	0.247	0.0031	**22**	4:8	0.0062	0.126	0.584
**K23**	**K**	0.247	0.0031	**23**	4:8	0.0062	0.143	0.567
**K24**	**K**	0.247	0.0031	**24**	4:8	0.0062	0.143	0.567
**K25**	**K**	0.247	0.0031	**25**	4:8	0.0062	0.270	0.483
**K26**	**K**	0.247	0.0031	**26**	4:8	0.0062	0.146	0.607
**K27**	**K**	0.247	0.0031	**27**	4:8	0.0062	0.171	0.582
**K28**	**K**	0.247	0.0031	**28**	4:8	0.0062	0.179	0.574
**K29**	**K**	0.247	0.0031	**29**	4:8	0.0062	0.182	0.571
**K30**	**K**	0.247	0.0031	**30**	4:8	0.0062	0.131	0.622
**K31**	**K**	0.247	0.0031	**31**	4:8	0.0062	0.171	0.582
**K32**	**K**	0.247	0.0031	**32**	4:8	0.0062	0.113	0.640
**K33**	**K**	0.247	0.0031	**33**	4:8	0.0062	0.184	0.569
**K34**	**K**	0.247	0.0031	**34**	4:8	0.0062	0.143	0.610
**L17**	**L**	0.290	0.0031	**17**	4:8	0.0062	0.075	0.635
**L18**	**L**	0.290	0.0031	**18**	4:8	0.0062	0.128	0.582
**L19**	**L**	0.290	0.0031	**19**	4:8	0.0062	0.240	0.470
**L20**	**L**	0.290	0.0031	**20**	4:8	0.0062	0.266	0.444
**L21**	**L**	0.290	0.0031	**21**	4:8	0.0062	0.251	0.459
**L22**	**L**	0.290	0.0031	**22**	4:8	0.0062	0.126	0.584
**L23**	**L**	0.290	0.0031	**23**	4:8	0.0062	0.143	0.567
**L24**	**L**	0.290	0.0031	**24**	4:8	0.0062	0.143	0.567
**L25**	**L**	0.290	0.0031	**25**	4:8	0.0062	0.270	0.440
**L26**	**L**	0.290	0.0031	**26**	4:8	0.0062	0.146	0.564
**L27**	**L**	0.290	0.0031	**27**	4:8	0.0062	0.171	0.539
**L28**	**L**	0.290	0.0031	**28**	4:8	0.0062	0.178	0.532
**L29**	**L**	0.290	0.0031	**29**	4:8	0.0062	0.182	0.528
**L30**	**L**	0.290	0.0031	**30**	4:8	0.0062	0.131	0.579
**L31**	**L**	0.290	0.0031	**31**	4:8	0.0062	0.171	0.539
**L32**	**L**	0.290	0.0031	**32**	4:8	0.0062	0.113	0.597
**L33**	**L**	0.290	0.0031	**33**	4:8	0.0062	0.183	0.528
**L34**	**L**	0.290	0.0031	**34**	4:8	0.0062	0.143	0.568
**M17**	**M**	0.240	0.0031	**17**	4:8	0.0062	0.074	0.686

**M18**	**M**	0.240	0.0031	**18**	4:8	0.0062	0.126	0.634
**M19**	**M**	0.240	0.0031	**19**	4:8	0.0062	0.235	0.525
**M20**	**M**	0.240	0.0031	**20**	4:8	0.0062	0.261	0.499
**M21**	**M**	0.240	0.0031	**21**	4:8	0.0062	0.246	0.514
**M22**	**M**	0.240	0.0031	**22**	4:8	0.0062	0.123	0.637
**M23**	**M**	0.240	0.0031	**23**	4:8	0.0062	0.140	0.620
**M24**	**M**	0.240	0.0031	**24**	4:8	0.0062	0.140	0.620
**M25**	**M**	0.240	0.0031	**25**	4:8	0.0062	0.265	0.495
**M26**	**M**	0.240	0.0031	**26**	4:8	0.0062	0.143	0.617
**M27**	**M**	0.240	0.0031	**27**	4:8	0.0062	0.167	0.593
**M28**	**M**	0.240	0.0031	**28**	4:8	0.0062	0.175	0.585
**M29**	**M**	0.240	0.0031	**29**	4:8	0.0062	0.179	0.581
**M30**	**M**	0.240	0.0031	**30**	4:8	0.0062	0.128	0.632
**M31**	**M**	0.240	0.0031	**31**	4:8	0.0062	0.167	0.593
**M32**	**M**	0.240	0.0031	**32**	4:8	0.0062	0.111	0.649
**M33**	**M**	0.240	0.0031	**33**	4:8	0.0062	0.183	0.572
**M34**	**M**	0.240	0.0031	**34**	4:8	0.0062	0.143	0.612
**N17**	**N**	0.240	0.0031	**17**	4:8	0.0062	0.074	0.686
**N18**	**N**	0.240	0.0031	**18**	4:8	0.0062	0.126	0.634
**N19**	**N**	0.240	0.0031	**19**	4:8	0.0062	0.235	0.525
**N20**	**N**	0.240	0.0031	**20**	4:8	0.0062	0.261	0.499
**N21**	**N**	0.240	0.0031	**21**	4:8	0.0062	0.246	0.514
**N22**	**N**	0.240	0.0031	**22**	4:8	0.0062	0.123	0.637
**N23**	**N**	0.240	0.0031	**23**	4:8	0.0062	0.140	0.620
**N24**	**N**	0.240	0.0031	**24**	4:8	0.0062	0.140	0.620
**N25**	**N**	0.240	0.0031	**25**	4:8	0.0062	0.265	0.495
**N26**	**N**	0.240	0.0031	**26**	4:8	0.0062	0.143	0.617
**N27**	**N**	0.240	0.0031	**27**	4:8	0.0062	0.167	0.593
**N28**	**N**	0.240	0.0031	**28**	4:8	0.0062	0.175	0.585
**N29**	**N**	0.240	0.0031	**29**	4:8	0.0062	0.179	0.581
**N30**	**N**	0.240	0.0031	**30**	4:8	0.0062	0.128	0.632
**N31**	**N**	0.240	0.0031	**31**	4:8	0.0062	0.167	0.593
**N32**	**N**	0.240	0.0031	**32**	4:8	0.0062	0.111	0.649
**N33**	**N**	0.240	0.0031	**33**	4:8	0.0062	0.183	0.572
**N34**	**N**	0.240	0.0031	**34**	4:8	0.0062	0.143	0.612
**O17**	**O**	0.220	0.0031	**17**	4:8	0.0062	0.074	0.676
**O18**	**O**	0.220	0.0031	**18**	4:8	0.0062	0.126	0.625
**O19**	**O**	0.220	0.0031	**19**	4:8	0.0062	0.235	0.516

**O20**	**O**	0.220	0.0031	**20**	4:8	0.0062	0.261	0.490
**O21**	**O**	0.220	0.0031	**21**	4:8	0.0062	0.246	0.505
**O22**	**O**	0.220	0.0031	**22**	4:8	0.0062	0.123	0.628
**O23**	**O**	0.220	0.0031	**23**	4:8	0.0062	0.140	0.610
**O24**	**O**	0.220	0.0031	**24**	4:8	0.0062	0.140	0.610
**O25**	**O**	0.220	0.0031	**25**	4:8	0.0062	0.264	0.486
**O26**	**O**	0.220	0.0031	**26**	4:8	0.0062	0.142	0.608
**O27**	**O**	0.220	0.0031	**27**	4:8	0.0062	0.167	0.583
**O28**	**O**	0.220	0.0031	**28**	4:8	0.0062	0.174	0.576
**O29**	**O**	0.220	0.0031	**29**	4:8	0.0062	0.178	0.572
**O30**	**O**	0.220	0.0031	**30**	4:8	0.0062	0.127	0.623
**O31**	**O**	0.220	0.0031	**31**	4:8	0.0062	0.167	0.583
**O32**	**O**	0.220	0.0031	**32**	4:8	0.0062	0.110	0.640
**O33**	**O**	0.220	0.0031	**33**	4:8	0.0062	0.184	0.559
**O34**	**O**	0.220	0.0031	**34**	4:8	0.0062	0.144	0.599
**P17**	**P**	0.290	0.0031	**17**	4:8	0.0062	0.074	0.707
**P18**	**P**	0.290	0.0031	**18**	4:8	0.0062	0.126	0.655
**P19**	**P**	0.290	0.0031	**19**	4:8	0.0062	0.235	0.546
**P20**	**P**	0.290	0.0031	**20**	4:8	0.0062	0.261	0.521
**P21**	**P**	0.290	0.0031	**21**	4:8	0.0062	0.246	0.535
**P22**	**P**	0.290	0.0031	**22**	4:8	0.0062	0.123	0.658
**P23**	**P**	0.290	0.0031	**23**	4:8	0.0062	0.140	0.641
**P24**	**P**	0.290	0.0031	**24**	4:8	0.0062	0.140	0.641
**P25**	**P**	0.290	0.0031	**25**	4:8	0.0062	0.263	0.517
**P26**	**P**	0.290	0.0031	**26**	4:8	0.0062	0.142	0.638
**P27**	**P**	0.290	0.0031	**27**	4:8	0.0062	0.166	0.614
**P28**	**P**	0.290	0.0031	**28**	4:8	0.0062	0.174	0.606
**P29**	**P**	0.290	0.0031	**29**	4:8	0.0062	0.177	0.603
**P30**	**P**	0.290	0.0031	**30**	4:8	0.0062	0.127	0.653
**P31**	**P**	0.290	0.0031	**31**	4:8	0.0062	0.166	0.614
**P32**	**P**	0.290	0.0031	**32**	4:8	0.0062	0.110	0.670
**P33**	**P**	0.290	0.0031	**33**	4:8	0.0062	0.184	0.589
**P34**	**P**	0.290	0.0031	**34**	4:8	0.0062	0.144	0.629
**Q17**	**Q**	0.270	0.0031	**17**	4:8	0.0062	0.074	0.636
**Q18**	**Q**	0.270	0.0031	**18**	4:8	0.0062	0.126	0.584
**Q19**	**Q**	0.270	0.0031	**19**	4:8	0.0062	0.235	0.473
**Q20**	**Q**	0.270	0.0031	**20**	4:8	0.0062	0.261	0.447
**Q21**	**Q**	0.270	0.0031	**21**	4:8	0.0062	0.246	0.462

**Q22**	**Q**	0.270	0.0031	**22**	4:8	0.0062	0.123	0.586
**Q23**	**Q**	0.270	0.0031	**23**	4:8	0.0062	0.140	0.569
**Q24**	**Q**	0.270	0.0031	**24**	4:8	0.0062	0.140	0.569
**Q25**	**Q**	0.270	0.0031	**25**	4:8	0.0062	0.267	0.443
**Q26**	**Q**	0.270	0.0031	**26**	4:8	0.0062	0.144	0.566
**Q27**	**Q**	0.270	0.0031	**27**	4:8	0.0062	0.169	0.541
**Q28**	**Q**	0.270	0.0031	**28**	4:8	0.0062	0.176	0.534
**Q29**	**Q**	0.270	0.0031	**29**	4:8	0.0062	0.180	0.530
**Q30**	**Q**	0.270	0.0031	**30**	4:8	0.0062	0.129	0.581
**Q31**	**Q**	0.270	0.0031	**31**	4:8	0.0062	0.169	0.541
**Q32**	**Q**	0.270	0.0031	**32**	4:8	0.0062	0.112	0.598
**Q33**	**Q**	0.270	0.0031	**33**	4:8	0.0062	0.184	0.521
**Q34**	**Q**	0.270	0.0031	**34**	4:8	0.0062	0.144	0.561
**R17**	**R**	0.302	0.0031	**17**	4:8	0.0062	0.080	0.655
**R18**	**R**	0.302	0.0031	**18**	4:8	0.0062	0.128	0.570
**R19**	**R**	0.302	0.0031	**19**	4:8	0.0062	0.240	0.458
**R20**	**R**	0.302	0.0031	**20**	4:8	0.0062	0.267	0.431
**R21**	**R**	0.302	0.0031	**21**	4:8	0.0062	0.250	0.480
**R22**	**R**	0.302	0.0031	**22**	4:8	0.0062	0.126	0.572
**R23**	**R**	0.302	0.0031	**23**	4:8	0.0062	0.144	0.554
**R24**	**R**	0.302	0.0031	**24**	4:8	0.0062	0.076	0.622
**R25**	**R**	0.302	0.0031	**25**	4:8	0.0062	0.271	0.427
**R26**	**R**	0.302	0.0031	**26**	4:8	0.0062	0.146	0.552
**R27**	**R**	0.302	0.0031	**27**	4:8	0.0062	0.170	0.530
**R28**	**R**	0.302	0.0031	**28**	4:8	0.0062	0.178	0.522
**R29**	**R**	0.302	0.0031	**29**	4:8	0.0062	0.181	0.519
**R30**	**R**	0.302	0.0031	**30**	4:8	0.0062	0.130	0.570
**R31**	**R**	0.302	0.0031	**31**	4:8	0.0062	0.170	0.530
**R32**	**R**	0.302	0.0031	**32**	4:8	0.0062	0.113	0.587
**R33**	**R**	0.302	0.0031	**33**	4:8	0.0062	0.183	0.517
**R34**	**R**	0.302	0.0031	**34**	4:8	0.0062	0.143	0.557
**S17**	**S**	0.165	0.0031	**17**	4:8	0.0062	0.076	0.759
**S18**	**S**	0.165	0.0031	**18**	4:8	0.0062	0.129	0.706
**S19**	**S**	0.165	0.0031	**19**	4:8	0.0062	0.241	0.594
**S20**	**S**	0.165	0.0031	**20**	4:8	0.0062	0.267	0.568
**S21**	**S**	0.165	0.0031	**21**	4:8	0.0062	0.252	0.583
**S22**	**S**	0.165	0.0031	**22**	4:8	0.0062	0.126	0.709
**S23**	**S**	0.165	0.0031	**23**	4:8	0.0062	0.144	0.691

**S24**	**S**	0.165	0.0031	**24**	4:8	0.0062	0.076	0.759
**S25**	**S**	0.165	0.0031	**25**	4:8	0.0062	0.271	0.564
**S26**	**S**	0.165	0.0031	**26**	4:8	0.0062	0.146	0.689
**S27**	**S**	0.165	0.0031	**27**	4:8	0.0062	0.171	0.664
**S28**	**S**	0.165	0.0031	**28**	4:8	0.0062	0.179	0.656
**S29**	**S**	0.165	0.0031	**29**	4:8	0.0062	0.183	0.652
**S30**	**S**	0.165	0.0031	**30**	4:8	0.0062	0.131	0.704
**S31**	**S**	0.165	0.0031	**31**	4:8	0.0062	0.171	0.664
**S32**	**S**	0.165	0.0031	**32**	4:8	0.0062	0.113	0.722
**S33**	**S**	0.165	0.0031	**33**	4:8	0.0062	0.184	0.651
**S34**	**S**	0.165	0.0031	**34**	4:8	0.0062	0.144	0.691
**T17**	**T**	0.132	0.0031	**17**	4:8	0.0062	0.076	0.792
**T18**	**T**	0.132	0.0031	**18**	4:8	0.0062	0.128	0.740
**T19**	**T**	0.132	0.0031	**19**	4:8	0.0062	0.240	0.628
**T20**	**T**	0.132	0.0031	**20**	4:8	0.0062	0.267	0.601
**T21**	**T**	0.132	0.0031	**21**	4:8	0.0062	0.252	0.616
**T22**	**T**	0.132	0.0031	**22**	4:8	0.0062	0.126	0.742
**T23**	**T**	0.132	0.0031	**23**	4:8	0.0062	0.143	0.725
**T24**	**T**	0.132	0.0031	**24**	4:8	0.0062	0.076	0.792
**T25**	**T**	0.132	0.0031	**25**	4:8	0.0062	0.271	0.597
**T26**	**T**	0.132	0.0031	**26**	4:8	0.0062	0.146	0.722
**T27**	**T**	0.132	0.0031	**27**	4:8	0.0062	0.171	0.697
**T28**	**T**	0.132	0.0031	**28**	4:8	0.0062	0.179	0.689
**T29**	**T**	0.132	0.0031	**29**	4:8	0.0062	0.182	0.686
**T30**	**T**	0.132	0.0031	**30**	4:8	0.0062	0.131	0.737
**T31**	**T**	0.132	0.0031	**31**	4:8	0.0062	0.171	0.697
**T32**	**T**	0.132	0.0031	**32**	4:8	0.0062	0.113	0.755
**T33**	**T**	0.132	0.0031	**33**	4:8	0.0062	0.184	0.684
**T34**	**T**	0.132	0.0031	**34**	4:8	0.0062	0.143	0.725
**U17**	**U**	0.295	0.0031	**17**	4:8	0.0062	0.076	0.629
**U18**	**U**	0.295	0.0031	**18**	4:8	0.0062	0.129	0.576
**U19**	**U**	0.295	0.0031	**19**	4:8	0.0062	0.241	0.464
**U20**	**U**	0.295	0.0031	**20**	4:8	0.0062	0.267	0.438
**U21**	**U**	0.295	0.0031	**21**	4:8	0.0062	0.252	0.453
**U22**	**U**	0.295	0.0031	**22**	4:8	0.0062	0.126	0.579
**U23**	**U**	0.295	0.0031	**23**	4:8	0.0062	0.144	0.561
**U24**	**U**	0.295	0.0031	**24**	4:8	0.0062	0.076	0.629
**U25**	**U**	0.295	0.0031	**25**	4:8	0.0062	0.271	0.434

**U26**	**U**	0.295	0.0031	**26**	4:8	0.0062	0.146	0.559
**U27**	**U**	0.295	0.0031	**27**	4:8	0.0062	0.172	0.533
**U28**	**U**	0.295	0.0031	**28**	4:8	0.0062	0.179	0.526
**U29**	**U**	0.295	0.0031	**29**	4:8	0.0062	0.183	0.522
**U30**	**U**	0.295	0.0031	**30**	4:8	0.0062	0.131	0.574
**U31**	**U**	0.295	0.0031	**31**	4:8	0.0062	0.172	0.533
**U32**	**U**	0.295	0.0031	**32**	4:8	0.0062	0.113	0.592
**U33**	**U**	0.295	0.0031	**33**	4:8	0.0062	0.184	0.521
**U34**	**U**	0.295	0.0031	**34**	4:8	0.0062	0.144	0.561

S5. Automated Data Analysis

Analysis of the three experimental characterisation methods (computer vision turbidity, ^{1}H NMR and HRMS) have been integrated into the package *cagey* and documentation given within the GitHub repository (https://github.com/GreenawayLab/cagey).

The package automates each step from each characterisation method's raw data files:

- turbidity_measurement.json and turbidity_data.csv for the turbidity computer vision (details given in section S5.1)
- NMR folders of each reaction (details given in section S5.2)
- Data files from Agilent for each HRMS sample (details given in section S5.3)

The precursor combinations (**A1-U34**) have an associated experiment code, plate number and formulation number (position on plate). For each individual characterisation method, automated analysis is outputted as a dataframe with callable functions which show the full analyses. The user-friendly interface is described within the README file and corresponding documentation. The combination of all experimental methods are outputted as a machine-readable dataframe which can be exported as CSV file (along with all dataframe within *cagey*) and further used as the user wishes.

S5.1 Turbidity

A simple setup was assembled for the turbidity measurement, depicted in Figure S9. In a 3D printed vial holder for 2 x 2 mL vials, the vial on the left was filled with 1 mL of chloroform as the reference solvent, and the reaction samples were placed on the right with the same total reaction volume. The vial holder was secured with tape to a Thermo Scientific Cimarec stirrer plate, and a Microsoft LifeCam HD-3000 web camera was positioned opposite. On the stirrer plate, a grey rubber cone was used for the normalisation region and a matt black case for eliminating reflection. The web camera was connected to a Raspberry Pi 4 Desktop that ran the computer vision script. The measurements scripts (https://github.com/GreenawayLab/Streamlining-Automated-Discovery-POCs/tree/main/Turbidity-monitoring-using-computer-vision) were written in the detect_turbidity_solubility.py file and the measurements conducted using mxTurbidityMonitor function for user friendly operations of the *HeinSight* Python package.[4]

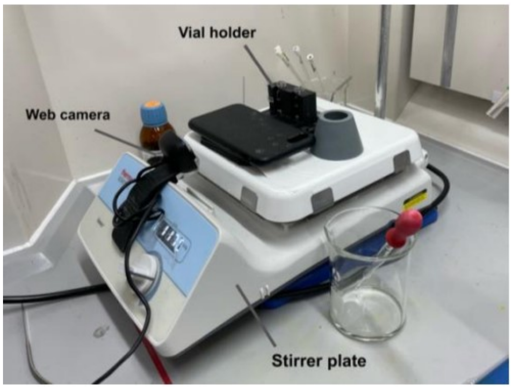

Figure S9: Basic setup for the computer vision turbidity measurements in the high-throughput screen. A custom 3D printed vial holder was secured with tape to a Thermo Scientific Cimarec stirrer plate, and a Microsoft LifeCam HD-3000 web camera was positioned opposite. The left of the vial holder

contained a vial with 1 mL of $CHCl_3$ as a reference. The right of the vial holder was the position of each reaction vial for measurement. A grey rubber cone for the normalisation region and a matt black case for eliminating reflection were included.

During a measurement, the script monitored the sample for a maximum period of two minutes and terminated monitoring whenever a “dissolved” or a “stable” (equilibrium reached but not dissolved) status was detected by *HeinSight*. If all samples were stirred for enough time prior to the measurements, and if the result remained unstable, the sample was identified as not dissolved. The state of each reaction solution was assigned by *HeinSight* according to a set of parameters listed in Table S6, which was tuned according to the actual lab environment. Researcher judgements based on manual observation were also recorded at the same time – it was not assumed that researcher judgements were correct for every sample due to bias of the individual, and therefore to lower this bias, the judgements were made by two independent researchers and validated by a third researcher if necessary.

Table S6: Parameters used by *HeinSight* for the determination of the status (dissolved or not dissolved) of each sample from the high-throughput screen.

Parameter	Definition
tm_n_minutes	Period of turbidity measurements to use
tm_std_max	Maximum standard deviation the data can have to be determined as stable
tm_sem_max	Maximum standard error the table can have to be determined as stable
tm_upper_limit	The turbidity measurement value that the mean and mode of the data is allowed to be above the known solvent reference for determining if the data has stabilised
tm_lower_limit	The turbidity measurement value that the mean and mode of the data is allowed to be below the known solvent reference for determining if the data has stabilised
tm_range_limit	The absolute difference in terms of the turbidity measurement, that the mode and mean of the data can have to be defined as stable but not dissolved
slope_upper_limit	How much above zero the slope from doing a linear regression can be to be determined as stable
slope_lower_limit	How much below zero the slope from doing a linear regression can be to be determined as stable

An overall accuracy of 96% was observed (Figure S10), with differences between the computer vision and researcher judgment attributed to highly coloured solutions where the colour arises from the light being absorbed by the material – in a turbid sample, the light reflected by the sample could be absorbed and weakened by coloured compounds, impacting the measured turbidity. Inhomogeneous samples were also challenging for the computer vision, where larger aggregates of solid could float to the top of the solution or stick to the vial wall, leading to the sample being incorrectly assigned as

‘dissolved’. Human eyes could normally recognise this to make the correct judgement by inspecting the entire vial. Instead, the computer vision only measures the selected region of interest, which might not include the area being affected.

Within the automated workflow, a function was written and incorporated into *cagey* to read the outputs of the turbidity_measurement.json and tubidity_data.csv, processing them to identify the timestamps and convert to a datetime format. It then calculated rolling statistics (mean and standard deviation) for a one-minute time window for a specific data column. The code also determines the stability of the data within this time window and categorizes it as 'dissolved,' 'undissolved,' or 'not determined' based on the mean turbidity value. It identifies a stable one-minute interval in the data and classifies the mixture based on this interval. If no stable interval is found, it raises a runtime error and returns the start and end times of the stable interval and the determined category. Full categorisation of the computer vision turbidity is integrated into the automated analysis package *cagey* (https://github.com/GreenawayLab/cagey).

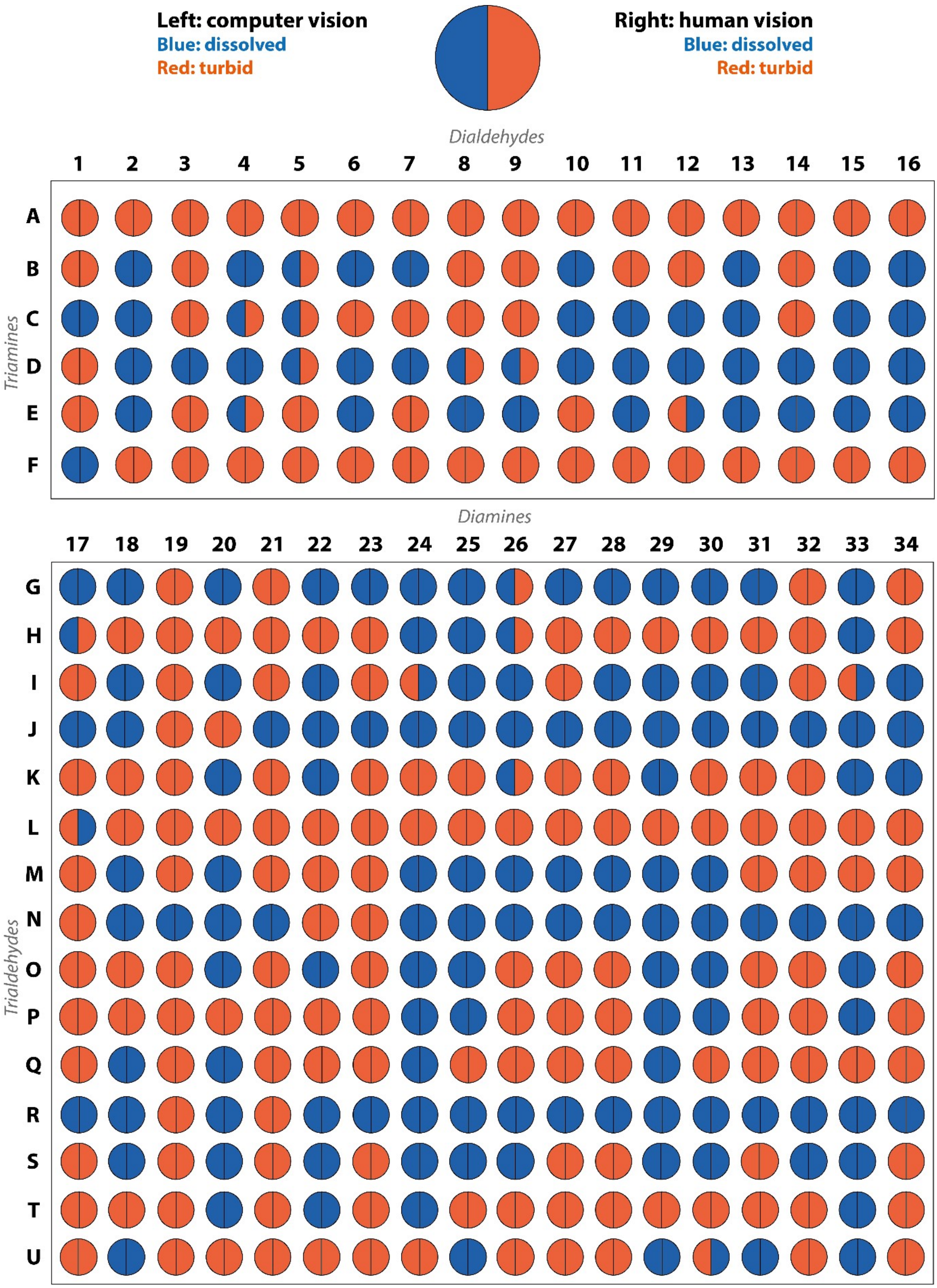

Figure S10: Turbidity analysis using both computer vision (left) and researcher manual judgement (right) for all precursor combinations. Pass (blue) is when no precipitate was observed within the reaction vial, and fail (red) is when precipitate was observed.

S5.2 ^{1}H NMR Spectra

The raw ^{1}H NMR spectra were subjected to an initial standardised pre-processing procedure which involved baseline correction, phase correction, and Fourier transformation in the proprietary Bruker TopSpin software. Subsequently, the pre-processed spectrum was analysed using the *nmrglue* package within an automated Python workflow *cagey*.[15] *nmrglue* is an open-source package for processing, manipulating, and analysing NMR spectra within Python, and provides several utility functions for reading Bruker NMR files, peak picking, and integrating NMR data. Using this software, aldehyde peaks were identified by performing peak picking in the chemical shift region of 9.0-11.0 ppm at a threshold of 10000 a.u. using the peak picking function within the *nmrglue* package, using the 'connected' algorithm. The 'connected' algorithm identifies all nodes (peaks) that are above a certain threshold, iteratively determining whether each of those nodes are separated from one another, defining each separate node as an individual peak. Imine peaks and other peaks within the aromatic region were detected within the range of 6.5-9.0 ppm, using the same peak picking algorithm and threshold. The range of 9-11 ppm was selected for detecting the aldehyde peak to accommodate the additional deshielding effects of the aromatic ring in close proximity to the aldehyde proton, present in many of the trialdehyde precursors. The range of 6.5-9.0 ppm was selected for the imine peaks due to their known occurrence across a wide range of shifts influenced by external factors that affect the proton environment, such as hydrogen bonding. To avoid detecting false positive peaks from the NMR solvent chloroform, the peak corresponding to this solvent at 7.26 ppm and its satellites were filtered after peak picking. Overall, this resulted in a 98% accuracy between the automated analysis compared to researcher manual assessment, validating the automated approach. All NMR analysis is integrated with the full automated analysis package *cagey* (https://github.com/GreenawayLab/cagey).

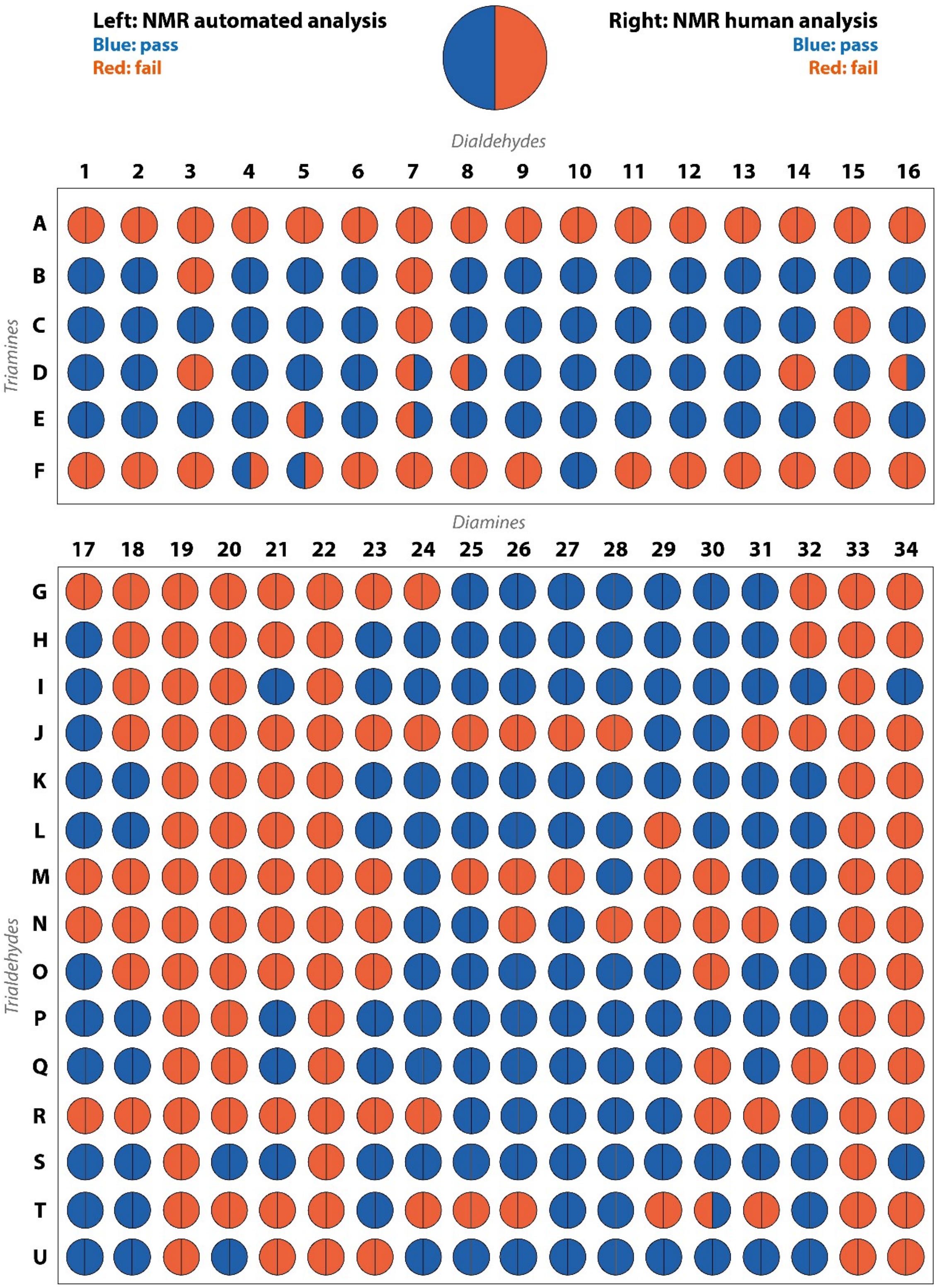

Figure S11: Comparison of the ^{1}H NMR results between the automated (left) and researcher analysis (right). A pass (blue) is when there is presence of an imine peak(s) and absence of an aldehyde peak(s), with fail (red) if only one or neither of these criteria are met.

S5.3 High Resolution Mass Spectrometry (HRMS)

Firstly integrated within *cagey*, the raw Agilent .d data files were converted to mzML files using MSConvert.[16] Mass detection was then carried out using *mzmine* software with the 'centroid' method, which assumes each signal above a certain noise level is a detected ion.[17] This requires centroid data, where each m/z peak is a discrete value with a line width of zero. A noise threshold of 1000 a.u. was used for detecting mass peaks initially. The extracted ion chromatograms for each mass ion peak were constructed using the Automated Data Analysis Pipeline (ADAP). Broadly, the goal of ADAP is to calculate the intensity of each m/z peak over time. The parameters used in this workflow included a minimum group size of 3, which is the number of consecutive scans where the intensity of each m/z peak in the group must be above a pre-defined intensity threshold of 3000 a.u. The minimum highest intensity of an m/z peak in a group was 1000 a.u., and each m/z ion peak was extracted based on a tolerance of 5 ppm within the scans. Finally, the m/z peaks in each extracted ion chromatogram were written to a text file for comparison with the expected m/z peaks for each cage topology. The extracted m/z peaks were compared with the expected m/z peak for each topology and precursor combination using a Python workflow. The expected mass ions of [2 + 3], [4 + 6], [6 + 9], and [8 + 12] reaction stoichiometries were calculated for each combination, considering the loss of 6, 12, 18, and 24 water molecules, respectively. The m/z peaks corresponding to singly and doubly charged species were also considered, along with the H, 2H, Na or NH_4 adducts which form during the interaction with the ionisation source. The *pyOpenMS* library was used to calculate each expected m/z peak, and each peak was compared with the extracted m/z peaks within a tolerance of 5 ppm to detect the presence of each topology.[18] All HRMS analysis is integrated within the full automated analysis package *cagey* (https://github.com/GreenawayLab/cagey).

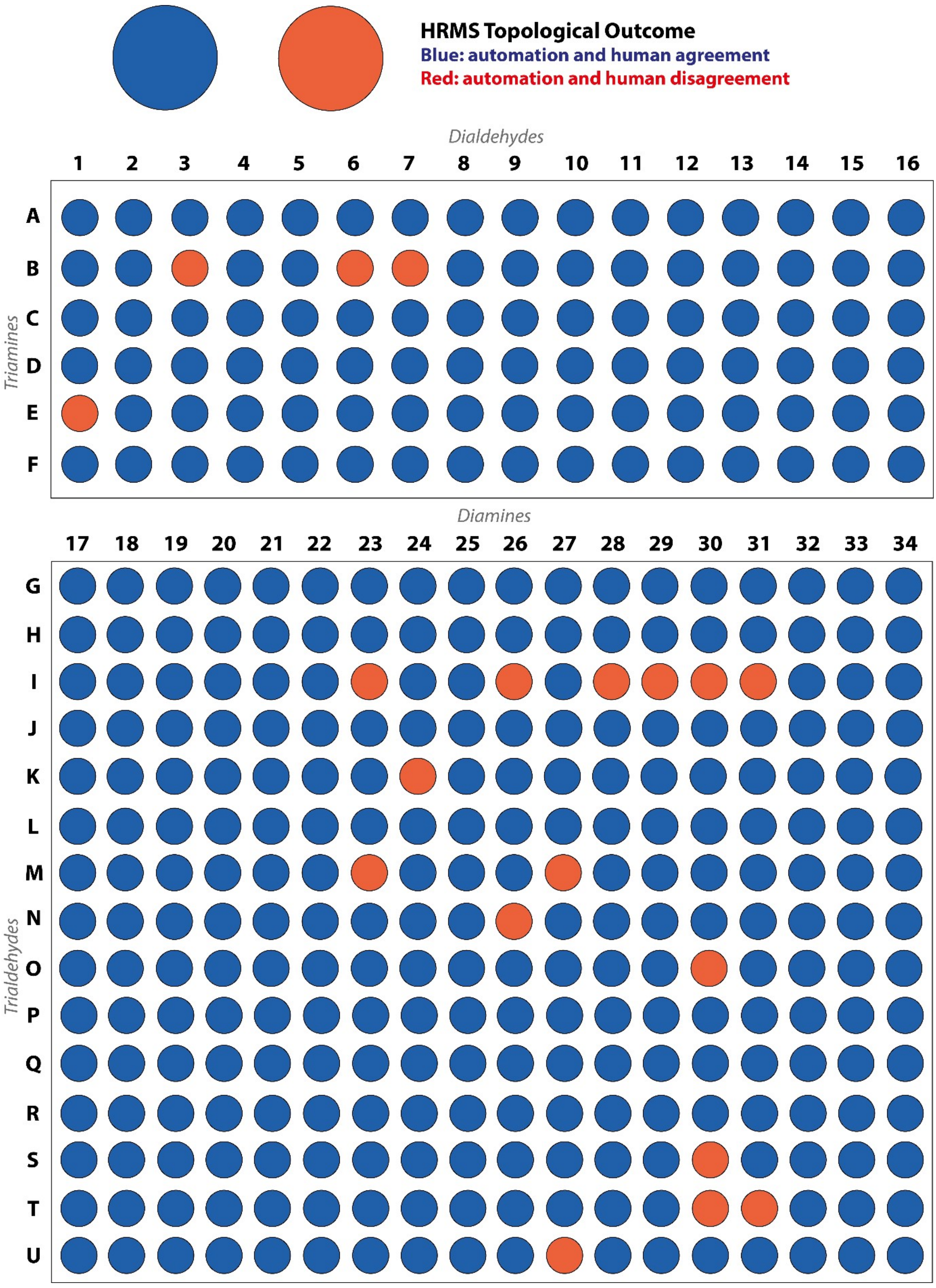

Figure S12: Comparison of topological outcomes from 366 precursor combinations between the automated and human analysis of the HRMS spectra. Blue shows agreement and red disagreement.

Table S7: Table of the outcomes where the human and automated analysis of the HRMS data did not agree.

Combination	Human Topological Outcome	Automated Topological Outcome
**B3**	[2+3, 4+6]	[4+6]
**B6**	[2+3, 4+6]	[4+6]
**B7**	[2+3, 4+6]	[2+3]
**E1**	[2+3, 4+6]	[4+6]
**I23**	[2+3, 4+6, 6+9, 8+12]	[4+6, 6+9, 8+12]
**I26**	[2+3, 4+6]	[2+3]
**I28**	[4+6]	[2+3, 4+6]
**I29**	[2+3]	[2+3, 4+6]
**I30**	[2+3, 4+6]	[2+3]
**I31**	[2+3]	[2+3, 4+6]
**K24**	[2+3, 4+6]	[4+6]
**M23**	[2+3, 4+6]	[4+6]
**M27**	[2+3]	[None]
**N26**	[2+3, 4+6]	[4+6]
**O30**	[None]	[2+3]
**S30**	[2+3]	[None]
**T30**	[2+3, 4+6]	[2+3]
**T31**	[2+3, 4+6]	[2+3]
**U27**	[None]	[2+3]

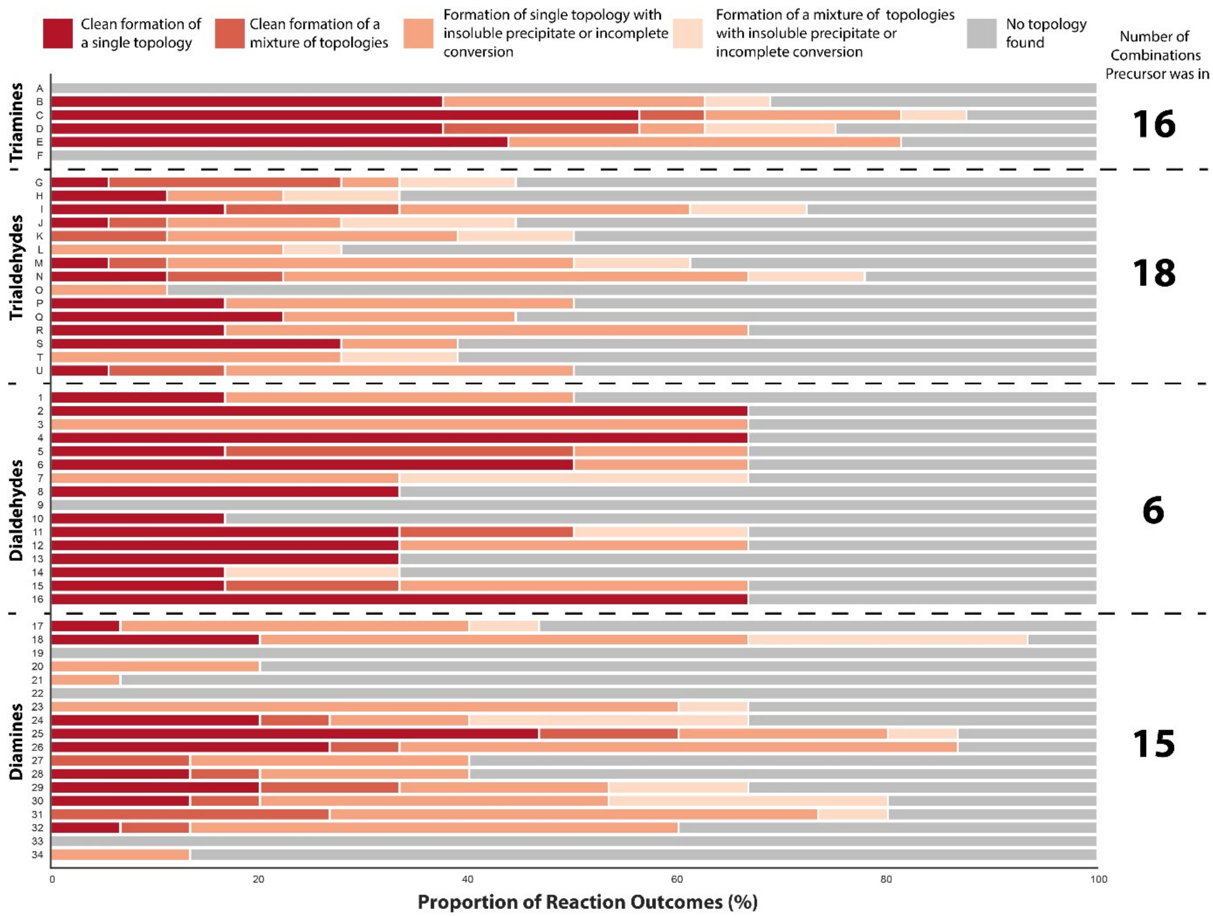

Figure S13: Each reaction outcome was categorised as follows: single topology observed to form cleanly (dark red) or incompletely (orange); mixture of topologies observed to form cleanly (red) or incompletely (coral); and no topology observed (grey). The proportion of outcomes is relative to the total number of reactions a precursor was involved in – the number of total combinations each precursor was in is given on the right. Each triamine precursor was combined with 16 dialdehydes and each trialdehyde was combined with 18 diamines, whereas each dialdehyde was combined with 6 triamines and each diamine was combined with 15 trialdehydes in the precursor library.

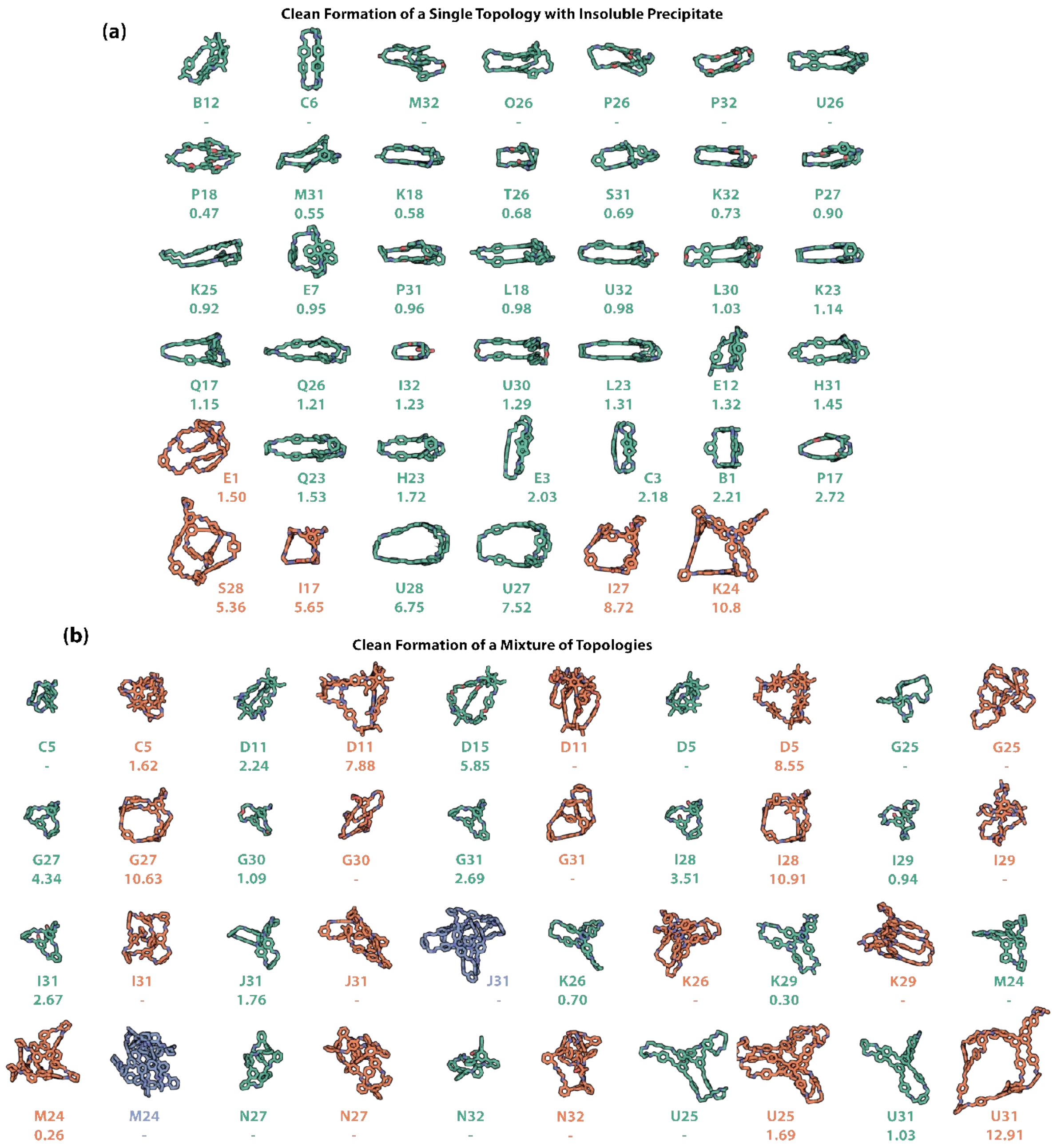

Figure S14: (a) Computational models of the 41 cages that showed full consumption of the aldehyde (by ^{1}H NMR) and formed one single cage topology (by HRMS) but showed insoluble precipitate (by computer vision turbidity check). Hits are shown by increasing predicted cavity size*;* (b) Computational models of the 19 precursor combinations that showed no insoluble precipitate (by computer vision turbidity check), full consumption of the aldehyde (by ^{1}H NMR), but multiple cage topologies were identified by HRMS, totalling 40 cages. Cage cavity diameters are given under the precursor combination label. No cavity is reported if the cage does not have the correct number of windows and/or no cavity diameter could be calculated computationally from the predicted structure, indicating the absence of a shape-persistent internal cavity*, at which point, hi*ts are shown alphabetically by increasing topology Tri+Di count. Carbon atoms in cages with the **Tri**^{2}**Di**^{3} topology are shown in green, and **Tri**^{4}**Di**^{6} in orange; in addition to nitrogen atoms shown in dark blue, oxygen in red. Hydrogens have been ommited for clarity.

S6. High-Throughput Experimental Screen

S6.1 Characterisation Data

Following the high-throughput screen, of the 366 precursor combinations, 54 combinations were identified as 'clean formation of a single topology' where the computer vision turbidity, ^{1}H NMR and HRMS checks were all 'passed'. While the automated analysis removed human bias and processed the data into a machine-readable format, the process was not quantitative and often residual amine remained due to its use in excess. However, the data of these clean hits was then manually inspected and where clear, analysed as follows:

B2$_{[2+3]}$

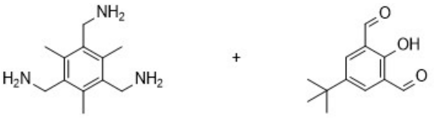

Synthesised according to the general procedure using (2,4,6-trimethylbenzene-1,3,5-triyl)trimethanamine **B** (0.0048 mmol, 6 equiv.) and 5-(tert-butyl)-2-hydroxyisophthalaldehyde **2** (0.0048 mmol, 6 equiv.) in $CHCl_3$ for 5 days to afford **B2**. **^{1}H NMR** (400 MHz, $CDCl_3$) δ_H 8.42 (s, 6H), 7.70 (s, 6H), 4.93 (s, 12H), 2.45 (m, 18H), 1.28 (m, 27H); **HRMS** (ES+) calc for [2+3] cage $C_{60}H_{72}N_6O_3$ 924.5660, found $[M+H]^+$ 925.6796.

B4$_{[4+6]}$

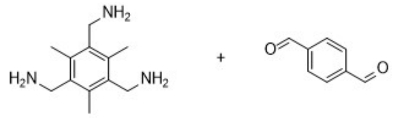

Synthesised according to the general procedure using (2,4,6-trimethylbenzene-1,3,5-triyl)trimethanamine **B** (0.0048 mmol, 6 equiv.) and terephthalaldehyde **4** (0.0048 mmol, 6 equiv.) in $CHCl_3$ for 5 days to afford **B4**. **^{1}H NMR** (400 MHz, $CDCl_3$) δ_H 8.30 (s, 12H), 7.75 (s, 24H), 4.92 (s, 24H), 2.36 (m, 36H); **HRMS** (ES+) calc for [4+6] cage $C_{96}H_{96}N_{12}$ 1416.7881, found $[M+H]^+$ 1417.84456.

B6$_{[4+6]}$

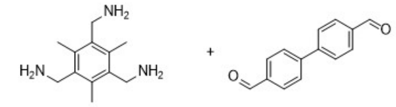

Synthesised according to the general procedure using (2,4,6-trimethylbenzene-1,3,5-triyl)trimethanamine **B** (0.0048 mmol, 6 equiv.) and 4,4'-biphenyldicarboxaldehyde **6** (0.0048 mmol, 6 equiv.) in $CHCl_3$ for 5 days to afford **B6**. **^{1}H NMR** (400 MHz, $CDCl_3$) δ_H 8.26 (m, 12H), 7.87 (m, 24H), 7.53 (m, 24H), 4.97 (m, 24H), 2.43 (m, 36H); **HRMS** (ES+) calc for [4+6] cage $C_{132}H_{120}N_{12}$ 1872.9759, found $[M+2H]^{2+}$ 937.49689.

B15$_{[2+3]}$

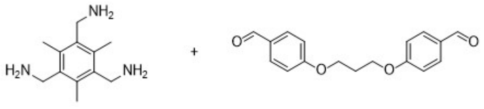

Synthesised according to the general procedure using (2,4,6-trimethylbenzene-1,3,5-triyl)trimethanamine **B** (0.0048 mmol, 6 equiv.) and 4,4'-(propane-1,3-diylbis(oxy))dibenzaldehyde **15** (0.0048 mmol, 6 equiv.) in $CHCl_3$ for 5 days to afford **B15**. **^{1}H NMR** (400 MHz, $CDCl_3$) δ_H 8.17 (s, 6H), 7.63 (d, J = 8.4 Hz, 12H), 6.87 (d, J = 8.3 Hz, 12H), 4.91 (s, 12H), 4.13 (t, J = 6.4 Hz, 12H), 2.41 (s, 18H), 2.26 (qu., J = 6.2 Hz, 6H); **HRMS** (ES+) calc for [2+3] cage $C_{75}H_{78}N_6O_6$ 1158.5977, found $[M+H]^+$ 1159.60198.

B16$_{[4+6]}$

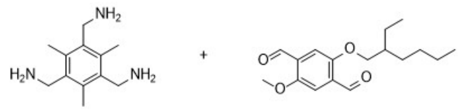

Synthesised according to the general procedure using (2,4,6-trimethylbenzene-1,3,5-triyl)trimethanamine **B** (0.0048 mmol, 6 equiv.) and 2-((2-ethylhexyl)oxy)-5-methoxyterephthalaldehyde **16** (0.0048 mmol, 6 equiv.) in $CHCl_3$ for 5 days to afford **B16**. **^{1}H NMR** (400 MHz, $CDCl_3$) – unsymmetrical nature of **16** leads to an unsymmetrical product and resulting multiplets, δ_H 8.63 (m, 12H), 7.46 (m, 12H), 4.88 (m, 24H), 3.76 (m, 30H), 2.44 (m, 36H), 1.45-0.55 (m, 90H); **HRMS (ESI+)** calc for [4+6] cage $C_{150}H_{204}N_{12}O_{12}$ 2365.5722, found $[M+H]^+$ 2366.57771.

C1$_{[2+3]}$

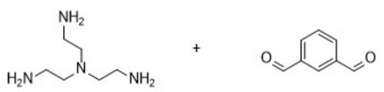

Synthesised according to the general procedure using *N*1,*N*1-bis(2-aminoethyl)ethane-1,2-diamine **C** (0.0048 mmol, 6 equiv.) and isophthalaldehyde **1** (0.0048 mmol, 6 equiv.) in $CHCl_3$ for 5 days to afford **C1**. **^{1}H NMR** (400 MHz, $CDCl_3$) δ_H 8.19 (d, *J* = 7.6 Hz, 6H), 7.59 (s, 6H), 7.53 (t, *J* = 7.6 Hz, 3H), 5.34 (s, 3H), 3.78 (br s, 6H), 3.29 (br s, 6H), 2.93 (br s, 6H), 2.72 (br s, 6H); **HRMS** (ES+) calc for [2+3] cage $C_{36}H_{42}N_8$ 586.3532, found $[M+H]^+$ 587.36775.

C2$_{[2+3]}$

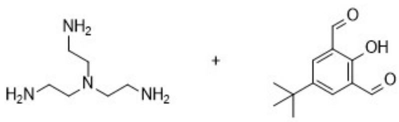

Synthesised according to the general procedure using N1,N1-bis(2-aminoethyl)ethane-1,2-diamine **C** (0.0048 mmol, 6 equiv.) and 5-(tert-butyl)-2-hydroxyisophthalaldehyde **2** (0.0048 mmol, 6 equiv.) in $CHCl_3$ for 5 days to afford **C2**. **^{1}H NMR** (400 MHz, $CDCl_3$) δ_H 14.13 (s, 2H), 8.50 (br m, 6H), 3.66 (br s, 12H), 2.93 (br s, 6H), 2.85 (br s, 12H), 2.74-2.59 (br m, 6H), 1.27 (app d, *J* = 36.3 Hz, 27H); **HRMS** (ES+) calc for [2+3] cage $C_{48}H_{66}N_8O_3$ 802.5258, found $[M+H]^+$ 803.53645.

C4$_{[2+3]}$

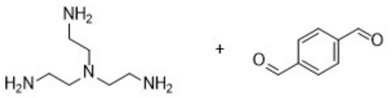

Synthesised according to the general procedure using *N*1,*N*1-bis(2-aminoethyl)ethane-1,2-diamine **C** (0.0048 mmol, 6 equiv.) and terephthalaldehyde **4** (0.0048 mmol, 6 equiv.) in $CHCl_3$ for 5 days to afford **C4**. **^{1}H NMR** (400 MHz, $CDCl_3$) δ_H 8.17 (s, 6H), 7.17 (s, 12H), 3.77 (br m, 12H), 2.77 (br m, 12H); **HRMS** (ES+) calc for [2+3] cage $C_{36}H_{42}N_8$ 586.3532, found $[M+H]^+$ 587.36496.

C8$_{[2+3]}$

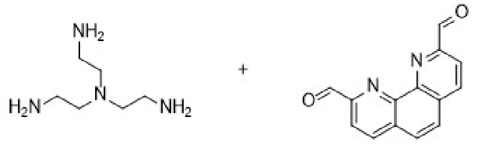

Synthesised according to the general procedure using *N*1,*N*1-bis(2-aminoethyl)ethane-1,2-diamine **C** (0.0048 mmol, 6 equiv.) and 1,10-phenanthroline-2,9-dicarbaldehyde **8** (0.0048 mmol, 6 equiv.) in $CHCl_3$ for 5 days to afford **C8**. **^{1}H NMR** (400 MHz, $CDCl_3$) – single unsymmetrical cage app in agreement with computationally predicted model, δ_H 8.92 (s, 4H), 8.89 (s, 2H), 8.33 (d, *J* = 8.4 Hz, 4H), 8.24 (d, *J* = 8.3 Hz, 4H), 8.09 (d, *J* = 8.4 Hz, 2H), 7.92 (d, *J* = 8.3 Hz, 2H), 7.79 (s, 4H), 7.49 (s, 2H), 3.98 (br m, 4H), 3.91 (app t, *J* = 7.0 Hz, 8H), 3.16 (br m, 4H), 2.97 (app t, *J* = 6.9 Hz, 8H); **HRMS** (ES+) calc for [2+3] cage $C_{54}H_{48}N_{14}$ 892.4186, found $[M+H]^+$ 893.42318.

C10[2+3]

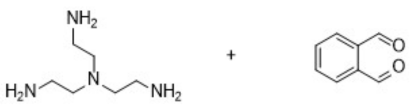

Synthesised according to the general procedure using *N*1,*N*1-bis(2-aminoethyl)ethane-1,2-diamine **C** (0.0048 mmol, 6 equiv.) and phthaldialdehyde **10** (0.0048 mmol, 6 equiv.) in $CHCl_3$ for 5 days to afford **C10**. **^{1}H NMR** (400 MHz, $CDCl_3$) δ_H 7.97 (s, 6H), 7.41 (t, *J* = 6.3 Hz, 12H), 7.32 (d, *J* = 7.0Hz, 6H), 4.52 (s, 12H), 4.03-3.87 (m, 32H), 2.99 (q, *J* = 5.8 Hz, 24H), 2.77 (t, *J* = 6.1 Hz, 12H), 2.64 (dd, *J* = 16.7, 5.5 Hz, 32H), 2.52 (t, *J* = 6.1 Hz, 12H); **HRMS** (ES+) calc for [2+3] cage $C_{36}H_{42}N_8$ 586.3532, found $[M+H]^+$ 587.35921.

C11[2+3]

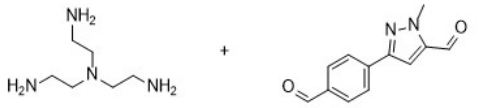

Synthesised according to the general procedure using *N*1,*N*1-bis(2-aminoethyl)ethane-1,2-diamine **C** (0.0048 mmol, 6 equiv.) and 3-(4-formylphenyl)-1-methyl-1H-pyrazole-5-carbaldehyde **11** (0.0048 mmol, 6 equiv.) in $CHCl_3$ for 5 days to afford **C11**. **^{1}H NMR** (400 MHz, $CDCl_3$) δ_H 7.98 (s, 1H), 7.91 (s, 2H), 7.84 (s, 2H), 7.79 (s, 1H), 7.46 (d, *J* = 7.9 Hz, 2H), 7.37 (d, *J* = 8.0 Hz, 4H), 7.31 (d, *J* = 8.2 Hz, 1H), 7.14 (d, *J* = 8.0 Hz, 1H), 7.01 (app t, *J* = 6.8 Hz, 4H), 5.70 (s, 1H), 5.52 (s, 2H), 4.36 – 4.16 (m, 9H), 3.77-3.68 (br m, 6H), 3.68-3.53 (br m, 6H), 2.77 (app br t, *J* = 5.6 Hz, 12H); **HRMS** (ES+) calc for [2+3] cage $C_{48}H_{54}N_{14}$ 826.4656, found $[M+H]^+$ 827.47644.

C13[2+3]

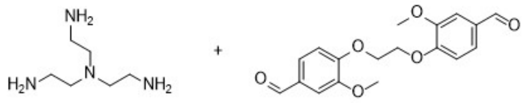

Synthesised according to the general procedure using *N*1,*N*1-bis(2-aminoethyl)ethane-1,2-diamine **C** (0.0048 mmol, 6 equiv.) and 4,4'-(ethane-1,2-diylbis(oxy))bis(3-methoxybenzaldehyde) **13** (0.0048 mmol, 6 equiv.) in $CHCl_3$ for 5 days to afford **C13**. **^{1}H NMR** (400 MHz, $CDCl_3$) δ_H 7.94 (s, 4H), 7.75 (s, 2H), 7.37 (d, *J* = 18.1 Hz, 2H), 7.11 (s, 4H), 6.99 (d, *J* = 4.4 Hz, 2H), 6.89 (s, 2H), 6.76 (d, *J* = 5.6 Hz, 4H), 6.57 (dd, *J* = 36.8, 8.1 Hz, 4H), 4.41 (s, 12H), 3.74 (app d, *J* = 34.7 Hz, 18H), 3.61 (br m, 12H), 2.85-2.74 (br m, 12H); **HRMS** (ES+) calc for [2+3] cage $C_{66}H_{78}N_8O_{12}$ 1174.57392, found $[M+H]^+$ 1175.58214.

C16[4+6]

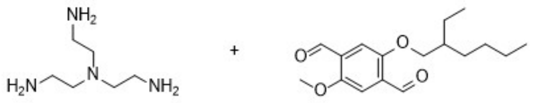

Synthesised according to the general procedure using *N*1,*N*1-bis(2-aminoethyl)ethane-1,2-diamine **C** (0.0048 mmol, 6 equiv.) and 2-((2-ethylhexyl)oxy)-5-methoxyterephthalaldehyde **16** (0.0048 mmol, 6 equiv.) in $CHCl_3$ for 5 days to afford **C16**. **^{1}H NMR** (400 MHz, $CDCl_3$) δ_H 8.53 (s, 4H), 8.44 (s, 4H), 8.37 (s, 4H), 7.10 (app d, *J* = 5.8 Hz, 6H), 6.95 (app d, *J* = 10.4 Hz, 6H), 3.90 (br m, 12H), 3.74 (s, 18H), 3.67 (s, 12H), 3.61-3.47 (br m, 12H), 3.24-3.07 (br m, 12H), 2.45-2.31 (br m, 12H), 1.37-0.82 (br m, 90H); **HRMS** (ES+) calc for [4+6] cage $C_{126}H_{192}N_{16}O_{12}$ 2121.49057, found $[M+H]^+$ 2122.48969.

D2[2+3]

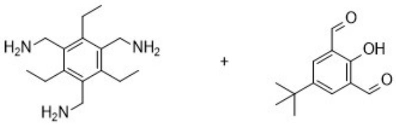

Synthesised according to the general procedure using (2,4,6-triethylbenzene-1,3,5-triyl)trimethanamine **D** (0.0048 mmol, 6 equiv.) and 5-(tert-butyl)-2-hydroxyisophthalaldehyde **2** (0.0048 mmol, 6 equiv.) in $CHCl_3$ for 5 days to afford **D2**. **^{1}H NMR** (400

MHz, $CDCl_3$) δ_H 8.41 (s, 6H), 7.70 (s, 6H), 4.92 (s, 12H), 2.51 (app d, *J* = 7.8 Hz, 12H), 1.34 (s, 27H), 1.18 (t, *J* = 7.0 Hz, 18H); **HRMS** (ES+) calc for [2+3] cage $C_{66}H_{84}N_6O_3$ 1008.6605, found $[M+H]^+$ 1009.67230.

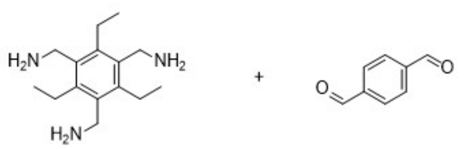

D4$_{[4+6]}$

Synthesised according to the general procedure using (2,4,6-triethylbenzene-1,3,5-triyl)trimethanamine **D** (0.0048 mmol, 6 equiv.) and terephthalaldehyde **4** (0.0048 mmol, 6 equiv.) in $CHCl_3$ for 5 days to afford **D4**. **^{1}H NMR** (400 MHz, $CDCl_3$) δ_H 8.29 (s, 12H), 7.72 (s, 24H), 4.94 (s, 24H), 2.73 (q, *J* = 7.5 Hz, 24H), 1.29-1.20 (m, 36H); **HRMS** (ES+) calc for [4+6] cage $C_{108}H_{120}N_{12}$ 1584.97589, found $[M+H]^+$ 1585.97975.

D6$_{[4+6]}$

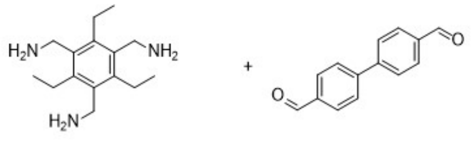

Synthesised according to the general procedure using (2,4,6-triethylbenzene-1,3,5-triyl)trimethanamine **D** (0.0048 mmol, 6 equiv.) and 4,4'-biphenyldicarboxaldehyde **6** (0.0048 mmol, 6 equiv.) in $CHCl_3$ for 5 days to afford **D6**. **^{1}H NMR** (400 MHz, $CDCl_3$) δ_H 8.45-8.16 (m, 12H), 7.88-7.40 (m, 48H), 5.17-4.89 (m, 24H), 2.91-2.72 (m, 24H), 1.37-1.10 (m, 36H); **HRMS** (ES+) calc for [4+6] cage $C_{144}H_{144}N_{12}$ 2041.16369, found $[M+H]^+$ 2042.16486.

D13$_{[2+3]}$

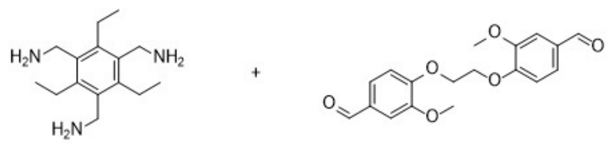

Synthesised according to the general procedure using (2,4,6-triethylbenzene-1,3,5-triyl)trimethanamine **D** (0.0048 mmol, 6 equiv.) and 4,4'-(ethane-1,2-diylbis(oxy))bis(3-methoxybenzaldehyde) **13** (0.0048 mmol, 6 equiv.) in $CHCl_3$ for 5 days to afford **D13**. **^{1}H NMR** (400 MHz, $CDCl_3$) δ_H 8.11-7.93 (m, 6H), 7.37 (app d, *J* = 21.4 Hz, 6H), 7.13-7.00 (m, 6H), 6.91 (app t, *J* = 9.3 Hz, 6H), 4.94 (app d, *J* = 11.9 Hz, 12H), 4.39 (app d, *J* = 10.2 Hz, 12H), 3.96-3.76 (m, 18H), 2.95-2.51 (m, 12H), 1.32-1.18 (m, 18H); **HRMS** (ES+) calc for [2+3] cage $C_{84}H_{96}N_6O_{12}$ 1380.7086, found $[M+H]^+$ 1381.71728.

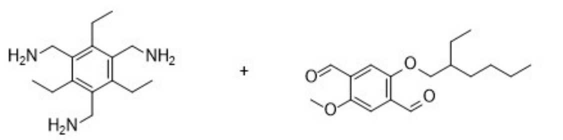

D16$_{[4+6]}$

Synthesised according to the general procedure using (2,4,6-triethylbenzene-1,3,5-triyl)trimethan-amine **D** (0.0048 mmol, 6 equiv.) and 2-((2-ethylhexyl)oxy)-5-methoxyterephthalaldehyde **16** (0.0048 mmol, 6 equiv.) in $CHCl_3$ for 5 days to afford **D16**. **^{1}H NMR** (400 MHz, $CDCl_3$) δ_H 8.68 (m, 12H), 7.45 (m, 12H), 4.99-4.81 (m, 24H), 3.83-3.63 (m, 30H), 2.82 (app q, *J* = 7.4 Hz, 24H – residual TriB present), 1.34-0.69 (br m, 126H – residual TriB present); **HRMS** (ES+) calc for [4+6] cage $C_{162}H_{228}N_{12}O_{12}$ 2533.75997, found $[M+H]^+$ 2534.76407.

E2$_{[2+3]}$

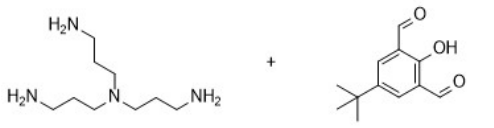

Synthesised according to the general procedure using *N*1,*N*1-bis(3-aminopropyl)propane-1,3-diamine **E** (0.0048 mmol, 6 equiv.) and 5-(tert-butyl)-2-hydroxyisophthalaldehyde **2** (0.0048 mmol, 6 equiv.) in $CHCl_3$ for 5 days to afford **E2**. **^{1}H NMR** (400 MHz, $CDCl_3$) δ_H 8.53 (br d, *J* = 24.4 Hz, 6H), 7.64 (br s, 6H), 3.63 (br s, 12H), 2.56-2.49 (br m, 12H), 1.92-1.78 (br m, 12H), 1.35-1.22 (br m, 27H); **HRMS** (ES+) calc for [2+3] cage $C_{54}H_{78}N_8O_3$ 886.6196, found $[M+H]^+$ 887.63308.

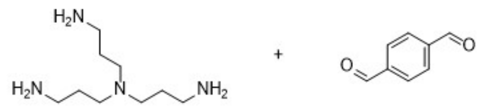

E4$_{[2+3]}$

Synthesised according to the general procedure using *N*1,*N*1-bis(3-aminopropyl)propane-1,3-diamine **E** (0.0048 mmol, 6 equiv.) and terephthalaldehyde **4** (0.0048 mmol, 6 equiv.) in $CHCl_3$ for 5 days to afford **E4**. **^{1}H NMR** (400 MHz, $CDCl_3$) δ_H 8.19 (s, 4H), 8.05 (s, 2H), 7.56 (s, 8H), 7.36 (s, 4H), 3.64 (app d, *J* = 6.2 Hz, 12H), 2.53-2.48 (m, 12H), 1.86-1.78 (m, 12H); **HRMS** (ES+) calc for [2+3] cage $C_{42}H_{54}N_8$ 670.4471, found $[M+H]^+$ 671.45820.

E6$_{[2+3]}$

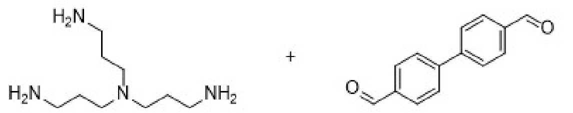

Synthesised according to the general procedure using *N*1,*N*1-bis(3-aminopropyl)propane-1,3-diamine **E** (0.0048 mmol, 6 equiv.) and 4,4'-biphenyldicarboxaldehyde **6** (0.0048 mmol, 6 equiv.) in $CHCl_3$ for 5 days to afford **E6**. **^{1}H NMR** (400 MHz, $CDCl_3$) δ_H 8.23 (s, 6H), 7.61 (d, *J* = 7.9 Hz, 12H), 7.39 (d, *J* = 8.2 Hz, 12H), 3.65 (t, *J* = 6.9 Hz, 12H), 2.52 (app q, *J* = 6.5 Hz, 12H), 1.86 (t, *J* = 6.6 Hz, 12H); **HRMS** (ES+) calc for [2+3] cage $C_{60}H_{66}N_8$ 898.5410, found $[M+H]^+$ 899.54966.

E8$_{[2+3]}$

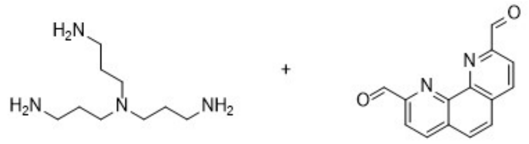

Synthesised according to the general procedure using *N*1,*N*1-bis(3-aminopropyl)propane-1,3-diamine **E** (0.0048 mmol, 6 equiv.) and 1,10-phenanthroline-2,9-dicarbaldehyde **8** (0.0048 mmol, 6 equiv.) in $CHCl_3$ for 5 days to afford **E8**. **^{1}H NMR** (400 MHz, $CDCl_3$) – single unsymmetrical cage app in agreement with computationally predicted model δ_H 8.80 (app d, *J* = 14.3 Hz, 6H), 8.30 (d, *J* = 8.6 Hz, 2H), 8.15 (d, *J* = 8.6 Hz, 6H), 7.98 (d, *J* = 8.2 Hz, 4H), 7.69 (s, 2H), 7.54 (s, 4H), 3.87-3.81 (br m, 12H), 2.61-2.54 (br m, 12H), 1.99-1.90 (br m, 12H); **HRMS** (ES+) calc for [2+3] cage $C_{60}H_{60}N_{14}$ 976.5125, found $[M+H]^+$ 899.54966.

E11$_{[2+3]}$

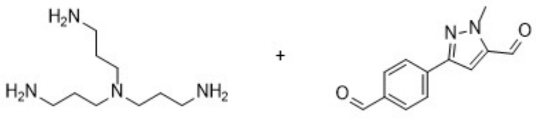

Synthesised according to the general procedure using *N*1,*N*1-bis(3-aminopropyl)propane-1,3-diamine **E** (0.0048 mmol, 6 equiv.) and 3-(4-formylphenyl)-1-methyl-1H-pyrazole-5-carbaldehyde **11** (0.0048 mmol, 6 equiv.) in $CHCl_3$ for 5 days to afford **E11**. **^{1}H NMR** (400 MHz, $CDCl_3$) δ_H 8.25 (s, 1H), 8.15 (s, 2H), 8.05 (s, 1H), 7.99 (d, *J* = 6.0 Hz, 1H), 7.89 (s, 1H), 7.63-7.38 (m, 11H), 6.58 (s, 1H), 6.53 (s, 1H), 6.23 (s,

1H), 6.07 (s, 1H), 4.23-4.05 (m, 9H), 3.76-3.49 (m, 12H), 2.60-2.43 (m, 12H), 1.94-1.76 (m, 12H); **HRMS** (ES+) calc for [2+3] cage $C_{54}H_{66}N_{14}$ 910.5595, found $[M+H]^+$ 911.56642.

E14$_{[2+3]}$

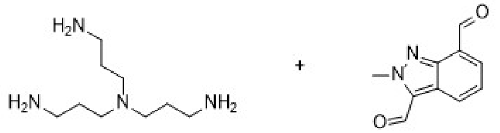

Synthesised according to the general procedure using *N*1,*N*1-bis(3-aminopropyl)propane-1,3-diamine **E** (0.0048 mmol, 6 equiv.) and 2-methyl-2H-indazole-3,7-dicarbaldehyde **14** (0.0048 mmol, 6 equiv.) in $CHCl_3$ for 5 days to afford **E14**. **^{1}H NMR** (400 MHz, $CDCl_3$) δ_H 8.79 (d, *J* = 35.4 Hz, 3H), 8.39 (d, *J* = 31.0 Hz, 3H), 7.76 (d, *J* = 8.1 Hz, 3H), 7.61 (dd, *J* = 41.4, 7.1 Hz, 3H), 6.99-6.89 (m, 3H), 4.48 (d, J = 12.3 Hz, 3H), 4.09 (d, *J* = 14.7 Hz, 6H), 3.78-3.63 (br m, 12H), 2.67-2.55 (br m, 12H), 1.96-1.84 (br m, 12H); **HRMS** (ES+) calc for [2+3] cage $C_{48}H_{60}N_{14}$ 832.5125, found $[M+H]^+$ 833.5273.

E16$_{[2+3]}$

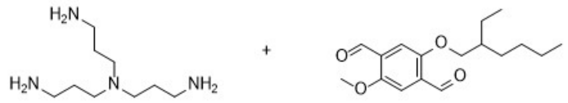

Synthesised according to the general procedure using *N*1,*N*1-bis(3-aminopropyl)propane-1,3-diamine **E** (0.0048 mmol, 6 equiv.) and 2-((2-ethylhexyl)oxy)-5-methoxyterephthalaldehyde **16** (0.0048 mmol, 6 equiv.) in $CHCl_3$ for 5 days to afford **E16**. **^{1}H NMR** (400 MHz, $CDCl_3$) δ_H 8.68 (s, 2H), 8.58 (s, 2H), 8.51 (d, *J* = 13.4 Hz, 3H), 8.44 (s, 2H), 7.51-7.45 (m, 3H), 3.96-3.57 (m, 27H), 2.57-2.48 (br m, 12H), 1.88-1.77 (br m, 12H), 1.39-0.82 (br m, 45H); **HRMS** (ES+) calc for [2+3] cage $C_{69}H_{108}N_8O_6$ 1144.8392, found $[M+H]^+$ 1145.84835.

G29$_{[2+3]}$

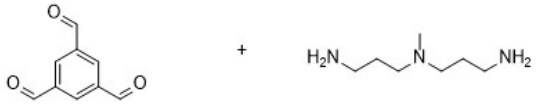

Synthesised according to the general procedure using 1,3,5-triformylbenzene **G** (0.0031 mmol, 4 equiv.) and 3,3′-diamino-*N*-methyldipropylamine **29** (0.0062 mmol, 8 equiv.) in $CHCl_3$ for 5 days to afford **G29**. **^{1}H NMR** (400 MHz, $CDCl_3$) δ_H 8.16 (s, 6H), 7.89 (s, 6H), 3.64 (t, *J* = 6.6 Hz, 12H), 2.38 (t, *J* = 6.8 Hz, 12H), 2.27 (s, 9H), 2.03 – 1.76 (m, 12H); **HRMS** (ES+) calc for [2+3] cage $C_{39}H_{57}N_9$ 651.4736, found $[M+H]^+$ 652.48336.

H25$_{[2+3]}$

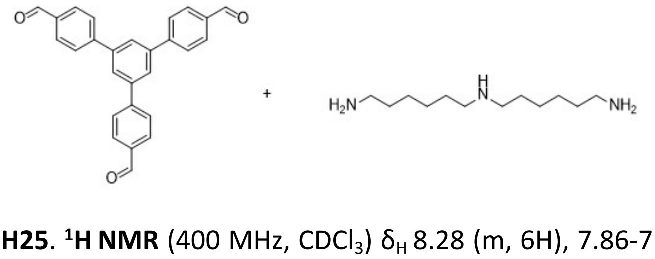

Synthesised according to the general procedure using 5'-(4-formylphenyl)-[1,1':3',1''-terphenyl]-4,4''-dicarbaldehyde **H** (0.0031 mmol, 4 equiv.) and bis(hexamethylene)triamine **25** (0.0062 mmol, 8 equiv.) in $CHCl_3$ for 5 days to afford **H25**. **^{1}H NMR** (400 MHz, $CDCl_3$) δ_H 8.28 (m, 6H), 7.86-7.66 (m, 30H), 3.69-3.55 (br m, 12H), 2.71-2.51 (br m, 24H), 1.80-1.28 (br m, 36H); **HRMS** (ES+) calc for [2+3] cage $C_{90}H_{111}N_9$ 1317.8962, found $[M+H]^+$ 1318.90093.

H26$_{[2+3]}$

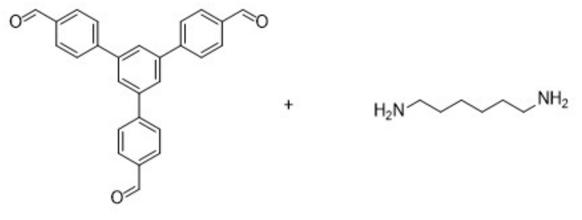

Synthesised according to the general procedure using 5'-(4-formylphenyl)-[1,1':3',1''-terphenyl]-4,4''-dicarbaldehyde **H** (0.0031 mmol, 4 equiv.) and hexamethylenediamine **26** (0.0062 mmol, 8 equiv.) in $CHCl_3$ for 5 days to afford **H26**. **^{1}H NMR** (400 MHz, $CDCl_3$) δ_H 8.32 (d, *J* = 11.2 Hz, 6H), 7.87-7.60 (br m, 24H), 7.55 (d, *J* = 8.1 Hz, 2H), 7.31 (d, *J* = 8.4 Hz, 4H), 3.65 (s, 12H), 1.74 (br s, 12H), 1.54-1.36 (br m, 12H); **HRMS** (ES+) calc for [2+3] cage $C_{72}H_{72}N_6$ 1020.58184, found $[M+H]^+$ 1021.58434.

I25$_{[2+3]}$

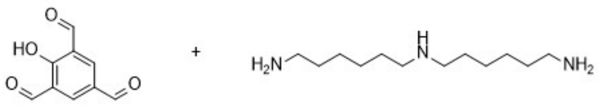

Synthesised according to the general procedure using 2-hydroxybenzene-1,3,5-tricarbaldehyde **I** (0.0031 mmol, 4 equiv.) and bis(hexamethylene)triamine **25** (0.0062 mmol, 8 equiv.) in $CHCl_3$ for 5 days to afford **I25**. **^{1}H NMR** (400 MHz, $CDCl_3$) δ_H 8.20 (s, 6H), 7.99 (br s, 4H), 3.72-3.52 (m, 24H), 1.78-1.25 (br m, 48H); **HRMS** (ES+) calc for [2+3] cage $C_{54}H_{87}N_9O_2$ 893.6983, found $[M+H]^+$ 894.70371.

I26$_{[2+3]}$

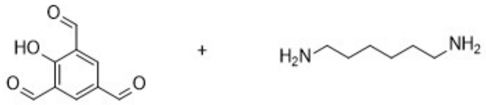

Synthesised according to the general procedure using 2-hydroxybenzene-1,3,5-tricarbaldehyde **I** (0.0031 mmol, 4 equiv.) and hexamethylenediamine **26** (0.0062 mmol, 8 equiv.) in $CHCl_3$ for 5 days to afford **I26**. **^{1}H NMR** (400 MHz, $CDCl_3$) δ_H 8.25-7.84 (br m, 10H), 3.69-3.50 (br m, 12H), 1.75-1.64 (br s, 12H), 1.53-1.30 (br m, 12H); **HRMS** (ES+) calc for [2+3] cage $C_{36}H_{48}N_6O_2$ 596.38387, found $[M+H]^+$ 597.89510.

I30$_{[2+3]}$

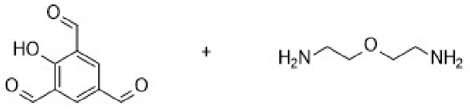

Synthesised according to the general procedure using 2-hydroxybenzene-1,3,5-tricarbaldehyde **I** (0.0031 mmol, 4 equiv.) and 2-(2-aminoethoxy)ethylamine **30** (0.0062 mmol, 8 equiv.) in $CHCl_3$ for 5 days to afford **I30**. **^{1}H NMR** (400 MHz, $CDCl_3$) δ_H 7.98 (app d, *J* = 10.9 Hz, 4H), 7.67 (br s, 6H), 3.74 (br s, 24H); **HRMS** (ES+) calc for [2+3] cage $C_{30}H_{36}N_6O_5$ 560.2747, found $[M+H]^+$ 561.28474.

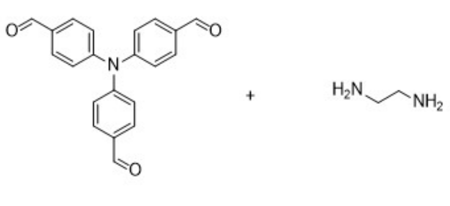

J17$_{[4+6]}$

Synthesised according to the general procedure using 4,4',4''-nitrilotribenzaldehyde **J** (0.0031 mmol, 4 equiv.) and ethane-1,2-diamine **17** (0.0062 mmol, 8 equiv.) in $CHCl_3$ for 5 days to afford **J17**. **^{1}H NMR** (400 MHz, $CDCl_3$) – symmetrical cage species present alongside other potential side-products δ_H 8.0. (s, 12H), 7.49 (d, *J* = 8.0 Hz, 24H), 7.05 (d, *J* = 8.0 Hz, 24H), 3.94 (s, 24H); **HRMS** (ES+) calc for [4+6] cage $C_{96}H_{84}N_{16}$ 1460.7065, found $[M+H]^+$ 1461.70981.

M28$_{[2+3]}$

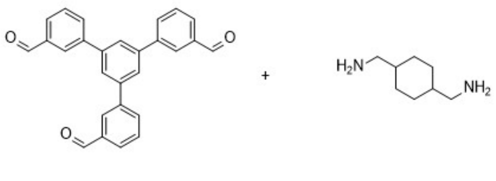

Synthesised according to the general procedure using 5'-(3-formylphenyl)-[1,1':3',1''-terphenyl]-3,3''-dicarbalde-hyde **M** (0.0031 mmol, 4 equiv.) and [4-

(aminomethyl)cyclohexyl]methylamine **28** (0.0062 mmol, 8 equiv.) in $CHCl_3$ for 5 days to afford **M28**. **^{1}H NMR** (400 MHz, $CDCl_3$) δ_H 8.56 (d, *J* = 6.1 Hz, 3H), 8.40 (d, *J* = 6.4 Hz, 3H), 8.33 (app d, *J* = 35.1 Hz, 2H), 8.11 (d, *J* = 8.2 Hz, 2H), 8.05 (d, *J* = 9.1 Hz, 2H), 7.88-7.69 (m, 12H), 7.55-7.37 (m, 12H), 3.70 (br s, 12H), 2.88 (t, *J* = 7.7 Hz, 6H), 2.75 (td, *J* = 6.8, 3.5 Hz, 4H), 2.49-2.37 (m, 8H), 1.92 (br m, 12H); **HRMS** (ES+) calc for [2+3] cage $C_{78}H_{78}N_6$ 1098.6287, found $[M+H]^+$ 1099.63859.

N24$_{[2+3]}$

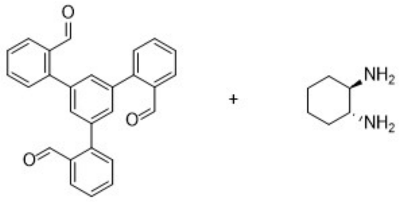

Synthesised according to the general procedure using 5'-(2-formylphenyl)-[1,1':3',1''-terphenyl]-2,2''-dicarbaldehyde **N** (0.0031 mmol, 4 equiv.) and (1*R*,2*R*)-cyclohexane-1,2-diamine **24** (0.0062 mmol, 8 equiv.) in $CHCl_3$ for 5 days to afford **N24**. **^{1}H NMR** (400 MHz, $CDCl_3$) δ_H 8.57 (s, 2H), 8.40 (s, 4H), 8.17 (d, *J* = 7.6 Hz, 2H), 7.77 (d, *J* = 7.6 Hz, 4H), 7.56-7.40 (m, 24H), 3.94 (br s, 6H), 2.99-2.91 (m, 3H), 2.80 (app q, *J* = 8.2 Hz, 3H), 2.03 (br s, 6H), 1.92 (br d, *J* = 13.7 Hz, 3H), 1.79-1.73 (app d, *J* = 13.7 Hz, 3H), 1.47-1.39 (br m, 6H); **HRMS** (ES+) calc for [2+3] cage $C_{72}H_{66}N_6$ 1014.5348, found $[M+H]^+$ 1015.54291.

N25$_{[2+3]}$

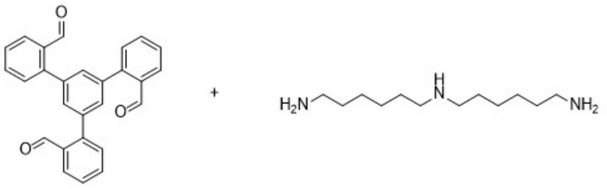

Synthesised according to the general procedure using 5'-(2-formylphenyl)-[1,1':3',1''-terphenyl]-2,2''-dicarbaldehyde **N** (0.0031 mmol, 4 equiv.) and bis(hexamethylene)triamine **25** (0.0062 mmol, 8 equiv.) in $CHCl_3$ for 5 days to afford **N25**. **^{1}H NMR** (400 MHz, $CDCl_3$) δ_H 8.42-8.30 (m, 6H), 8.11-8.01 (m, 6H), 7.52-7.28 (m, 24H), 3.55-3.37 (m, 12H), 1.75-1.08 (br m, 60H); **HRMS** (ES+) calc for [2+3] cage $C_{90}H_{111}N_9$ 1317.8962, found $[M+H]^+$ 1318.89995.

R25$_{[2+3]}$

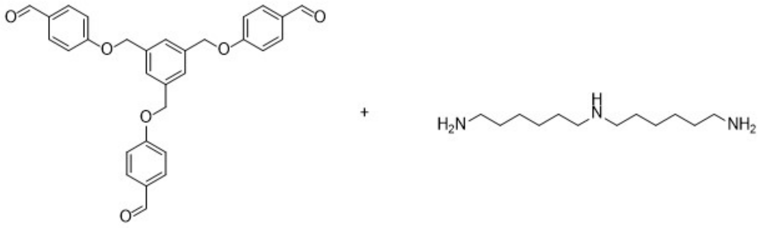

Synthesised according to the general procedure using 4,4',4''-((benzene-1,3,5 triyltris (methylene))tris(oxy))tribenz-aldehyde **R** (0.0031 mmol, 4 equiv.) and bis(hexamethylene)-triamine **25** (0.0062 mmol, 8 equiv.) in $CHCl_3$ for 5 days to afford **R25**. **^{1}H NMR** (400 MHz, $CDCl_3$) δ_H 8.21-8.14 (m, 4H), 8.13 (s, 2H), 7.69-7.61 (m, 8H), 7.55 (d, *J* = 8.2 Hz, 4H), 7.48-7.41 (m, 4H), 7.35 (s, 2H), 6.98 (d, *J* = 9.0 Hz, 8H), 6.83 (d, *J* = 8.1 Hz, 4H), 5.19 (s, 4H), 5.14-5.05 (m, 8H), 3.56 (br s, 12H), 2.61-2.51 (br m, 12H – residual Di25 present), 1.68 (br s, 12H), 1.54-1.28 (br m, 36H – residual Di25 present); **HRMS** (ES+) calc for [2+3] cage $C_{96}H_{123}N_9O_6$ 1497.9596, found $[M+H]^+$ 1498.95867.

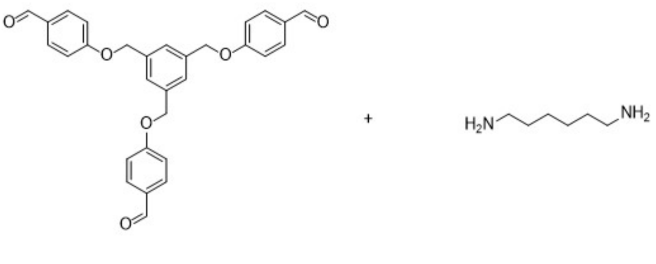

R26$_{[2+3]}$

Synthesised according to the general procedure using 4,4',4''-((benzene-1,3,5

triyltris (methylene))tris(oxy))tribenzalde-hyde **R** (0.0031 mmol, 4 equiv.) and hexamethylenediamine **26** (0.0062 mmol, 8 equiv.) in $CHCl_3$ for 5 days to afford **R26**.
^{1}H NMR (400 MHz, $CDCl_3$) δ_H 8.23-8.09 (m, 6H), 7.70-7.45 (m, 12H), 7.44-7.37 (m, 5H), 7.31 (s, 1H), 7.02-6.87 (m, 9H), 6.70 (d, *J* = 8.5 Hz, 3H), 5.24-5.01 (m, 12H), 3.57 (br s, 12H), 1.68 (br s, 12H), 1.55-1.30 (br m, 12H); **HRMS** (ES+) calc for [2+3] cage $C_{78}H_{84}N_6O_6$ 1200.6452 found, $[M+H]^+$ 1201.64396.

R28$_{[2+3]}$

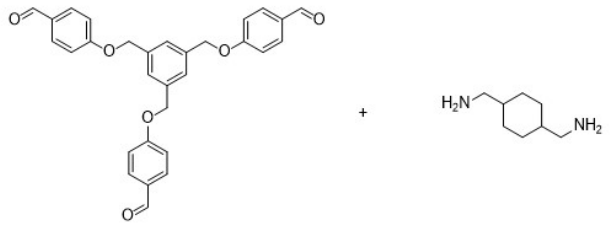

Synthesised according to the general procedure using 4,4',4''-((benzene-1,3,5 triyltris (methylene))tris(oxy))tribenzaldehyde **R** (0.0031 mmol, 4 equiv.) and [4-(aminomethyl)cyclohexyl]methylamine **28** (0.0062 mmol, 8 equiv.) in $CHCl_3$ for 5 days to afford **R28**. **^{1}H NMR** (400 MHz, $CDCl_3$) δ_H 8.18-8.09 (m, 6H), 7.71-7.54 (m, 12H), 7.50-7.40 (m, 6H), 7.04-6.89 (m, 12H), 5.12 (s, 12H), 3.57-3.40 (m, 12H), 2.00-0.78 (br m, 30H – present alongside residual Di28); **HRMS** (ES+) calc for [2+3] cage $C_{84}H_{90}N_6O_6$ 1278.69218, found $[M+H]^+$ 1279.69963.

S25$_{[2+3]}$

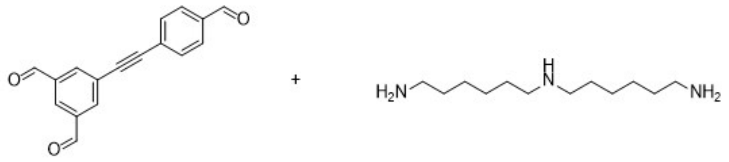

Synthesised according to the general procedure using 5-((4-formylphenyl)ethynyl)isophthal-aldehyde **S** (0.0031 mmol, 4 equiv.) and bis(hexamethylene)triamine **25** (0.0062 mmol, 8 equiv.) in $CHCl_3$ for 5 days to afford **S25**. **^{1}H NMR** (400 MHz, $CDCl_3$) δ_H 8.28 (d, *J* = 9.2 Hz, 6H), 7.97 (d, *J* = 16.4 Hz, 4H), 7.78 (s, 2H), 7.71 (d, *J* = 8.3 Hz, 4H), 7.55 (d, *J* = 7.6 Hz, 4H), 5.68 (s, 12H), 1.82-1.29 (br m, 60H – residual Di25 present); **HRMS** (ES+) calc for [2+3] cage $C_{70}H_{95}N_9$ 1061.7710, found $[M+H]^+$ 1062.7687.

Competitive reactivity: In addition, on closer manual inspection of the ^{1}H NMR and HRMS spectra of some of the clean hits, we believe competitive reactivity may have occurred in some instances:

C12$_{[2+3]}$ - risk of competitive 1,4- vs 1,2-addition to the unsaturated aldehyde in **12**.

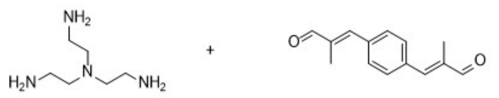

Synthesised according to the general procedure using *N*1,*N*1-bis(2-aminoethyl)ethane-1,2-diamine **C** (0.0048 mmol, 6 equiv.) and (2*Z*,2'*Z*)-3,3'-(1,4-phenylene)bis(2-methylacrylaldehyde) **12** (0.0048 mmol, 6 equiv.) in $CHCl_3$ for 5 days to afford **C12**. **^{1}H NMR** (400 MHz, $CDCl_3$) δ_H 7.84 (app d, *J* = 27.3 Hz, 6H), 6.97 (app d, *J* = 66.3 Hz, 12H), 6.46 (app d, *J* = 49.6 Hz, 6H), 3.61 (app br d, *J* = 20.5 Hz, 12H), 2.86 – 2.70 (br m, 12H), 2.13 (app d, *J* = 34.7 Hz, 18H); **HRMS** (ES+) calc for [2+3] cage $C_{54}H_{66}N_8$ 826.54104, found $[M+H]^+$ 827.55163.

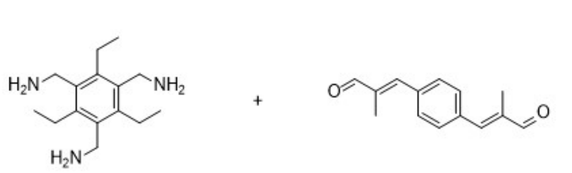

D12$_{[2+3]}$ - risk of competitive 1,4- vs 1,2-addition to the unsaturated aldehyde in **12**.

Synthesised according to the general procedure using (2,4,6-triethylbenzene-1,3,5-triyl)trimethanamine **D** (0.0048 mmol, 6 equiv.) and (2*Z*,2'*Z*)-3,3'-(1,4-phenylene)bis(2-methylacrylaldehyde) **12** (0.0048 mmol, 6 equiv.) in $CHCl_3$ for 5 days to afford **D12**. **^{1}H NMR** (400 MHz, $CDCl_3$) δ_H 6.97 (s, 3H), 6.70 (s, 12H), 6.29 (s, 3H), 6.09 (s, 6H), 5.05 (s, 12H), 2.37 (q, *J* = 7.5 Hz, 12H), 2.24 (s, 12H), 2.15 (s, 6H), 1.28 – 1.18 (m, 18H); **HRMS** (ES+) calc for [2+3] cage $C_{72}H_{84}N_6$ 1032.6757, found $[M+H]^+$ 1033.68405.

Q29$_{[2+3]}$ - risk of competitive 1,4- vs 1,2-addition to the unsaturated aldehyde in **Q**.

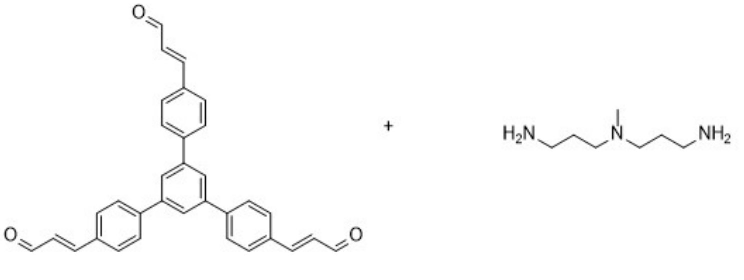

Synthesised according to the general procedure using 1,3,5-tris-(3-acetaldehydebenzene)-benzene) **Q** (0.0031 mmol, 4 equiv.) and 3,3'-diamino-N-methyldipropylamine **29** (0.0062 mmol, 8 equiv.) in $CHCl_3$ for 5 days to afford **Q29**. **^{1}H NMR** (400 MHz, $CDCl_3$) δ_H 7.96 (d, *J* = 8.2 Hz, 6H), 7.42 (s, 6H), 7.22 (d, *J* = 8.2 Hz, 12H), 7.13 (d, *J* = 8.0 Hz, 12H), 6.88-6.73 (m, 12H), 3.77-3.68 (br d, *J* = 12.1 Hz, 24H), 3.50 (app. q, *J* = 5.3 Hz, 6H), 2.87 (app q, *J* = 5.4 Hz, 6H), 1.34-1.22 (m, 9H); **HRMS** (ES+) calc for [2+3] cage $C_{87}H_{93}N_9$ 1263.7553, found $[M+H]^+$ 1264.75788.

S6.2 Spectra

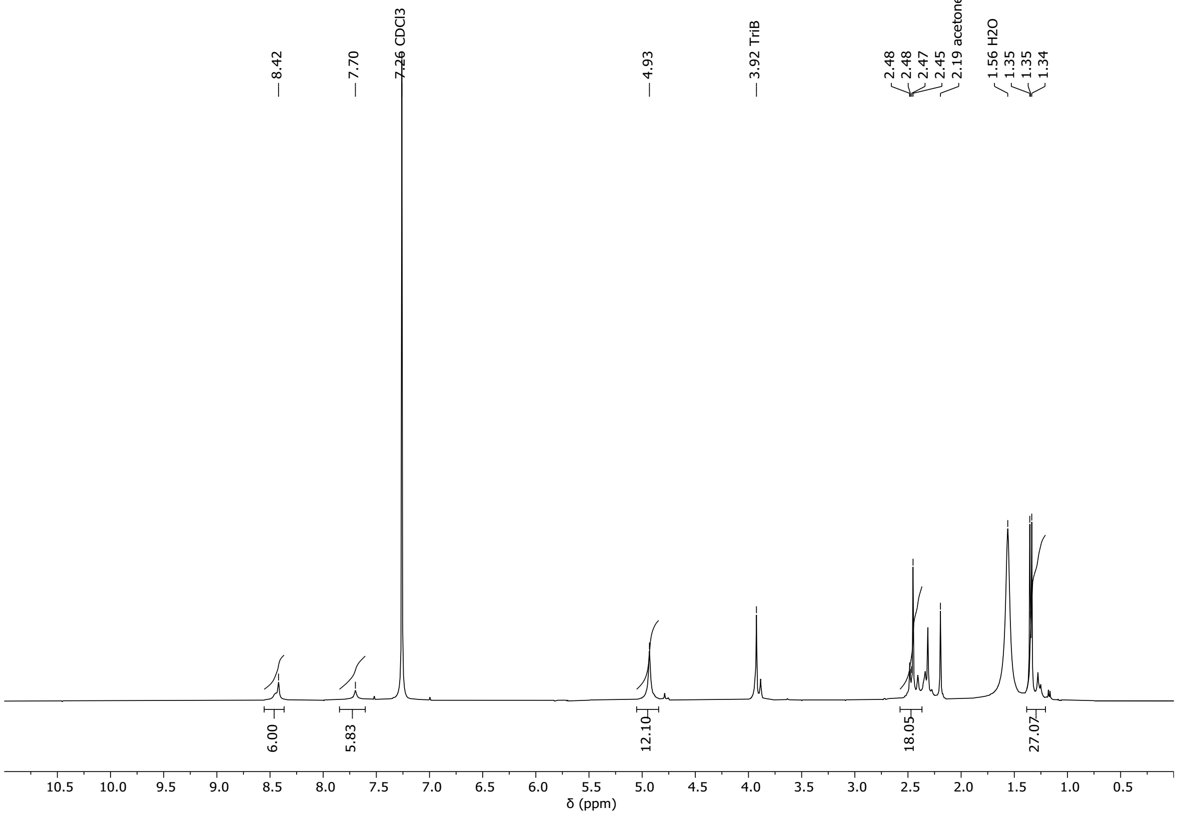

Figure S15: ^{1}H NMR ($CDCl_3$) spectrum for **B2$_{[2+3]}$**

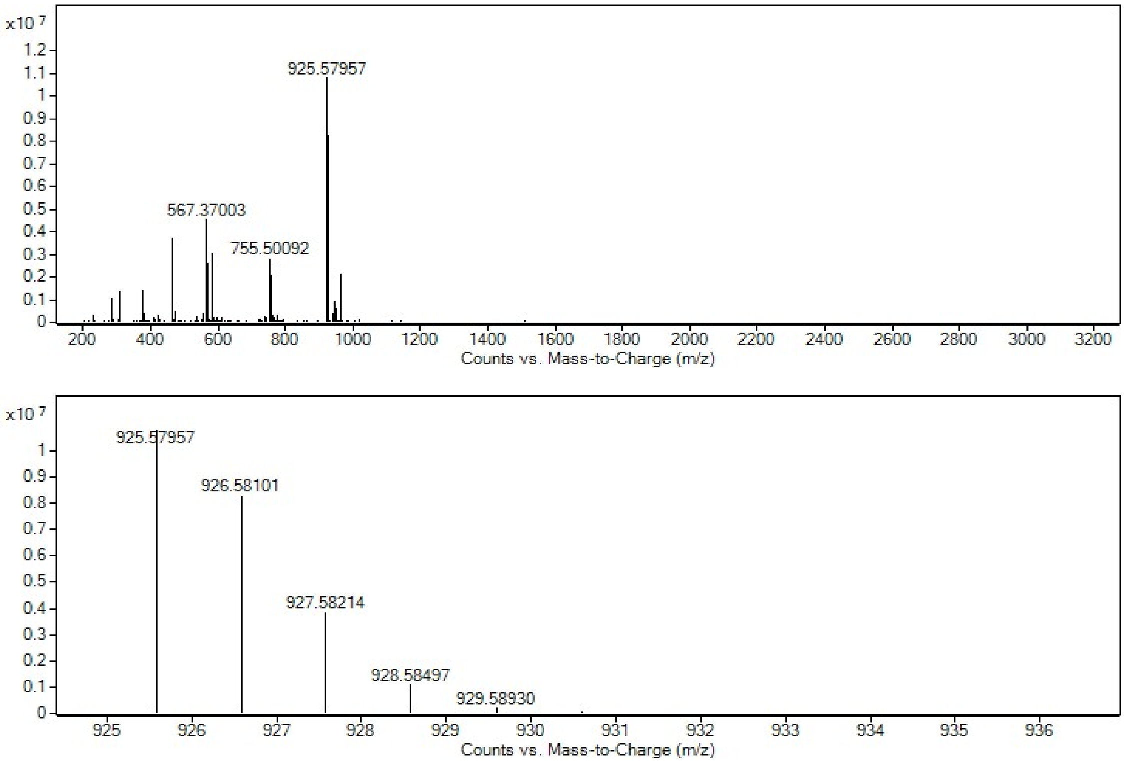

Figure S16: HRMS spectrum for **B2$_{[2+3]}$**

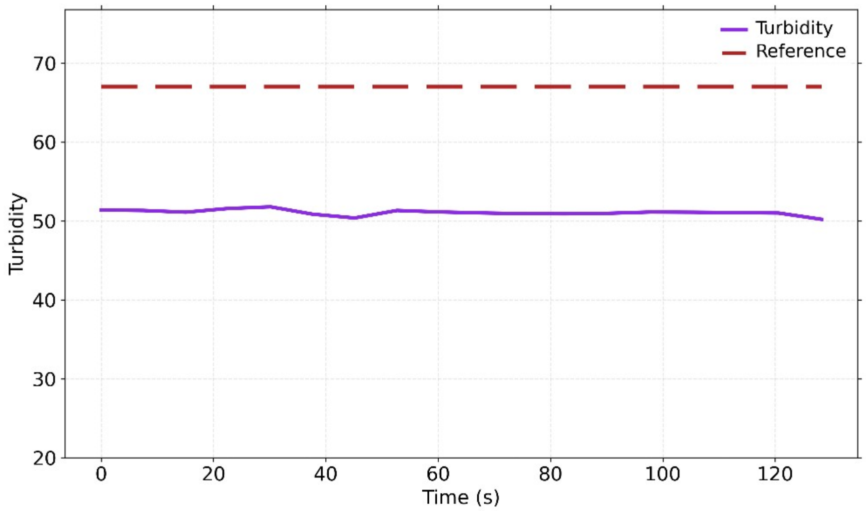

Figure S17: Turbidity vs time (s) for $\mathbf{B2}_{[2+3]}$

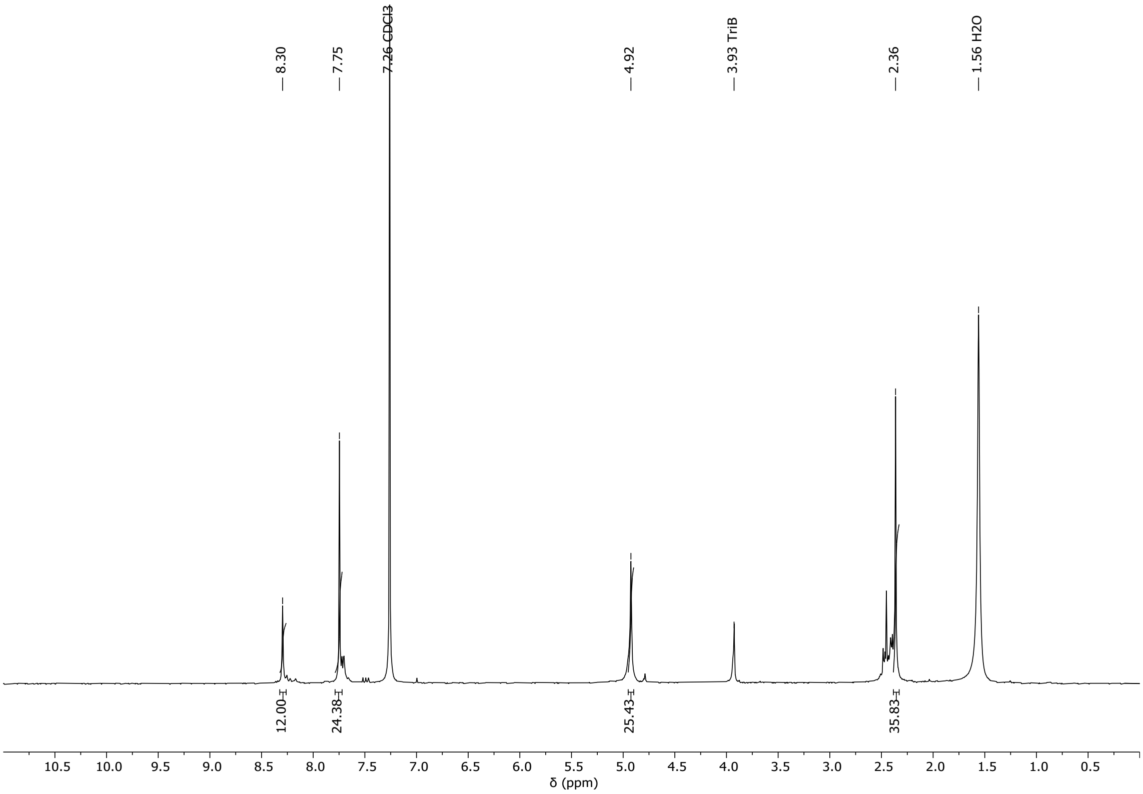

Figure S18: ^{1}H NMR ($CDCl_3$) spectrum for **B4$_{[4+6]}$**

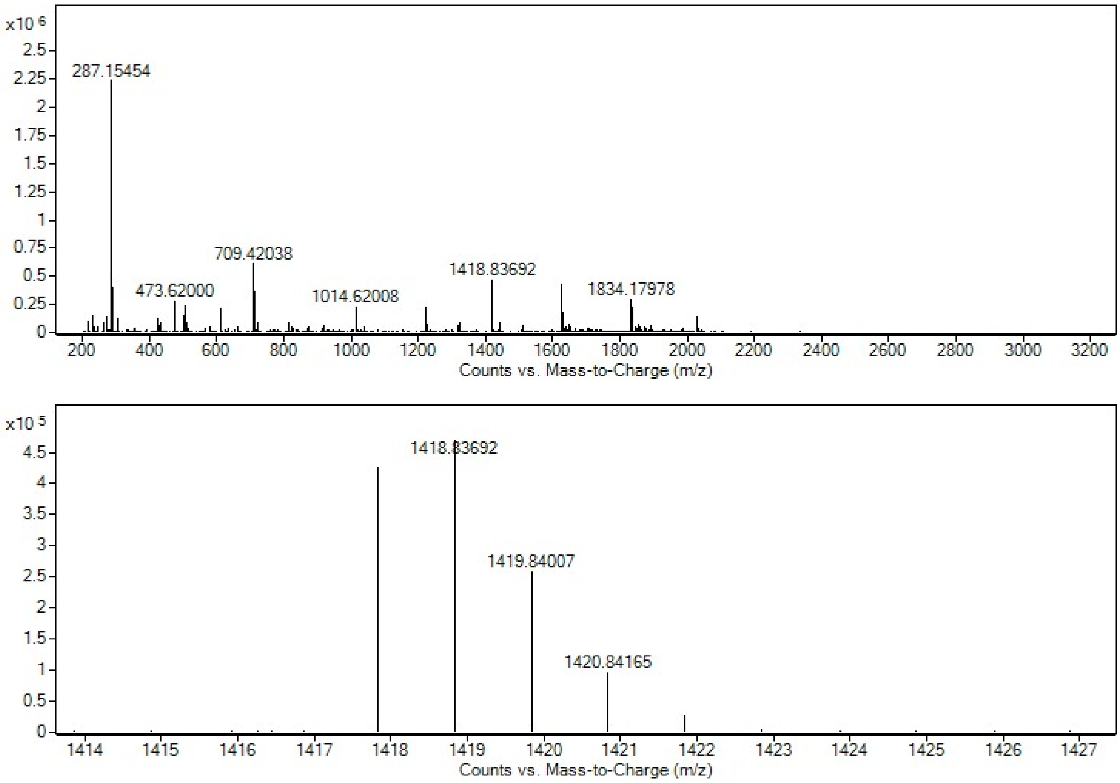

Figure S19: HRMS spectrum for **B4$_{[4+6]}$**

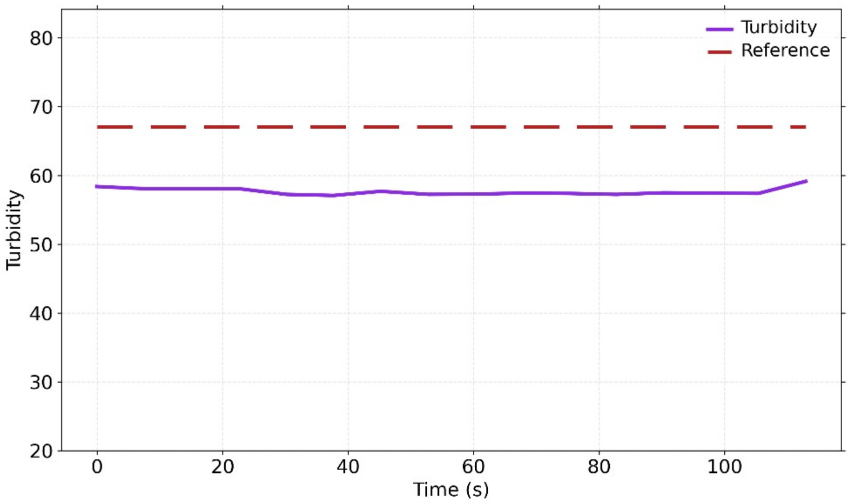

Figure S20: Turbidity vs time (s) for **B4$_{[4+6]}$**

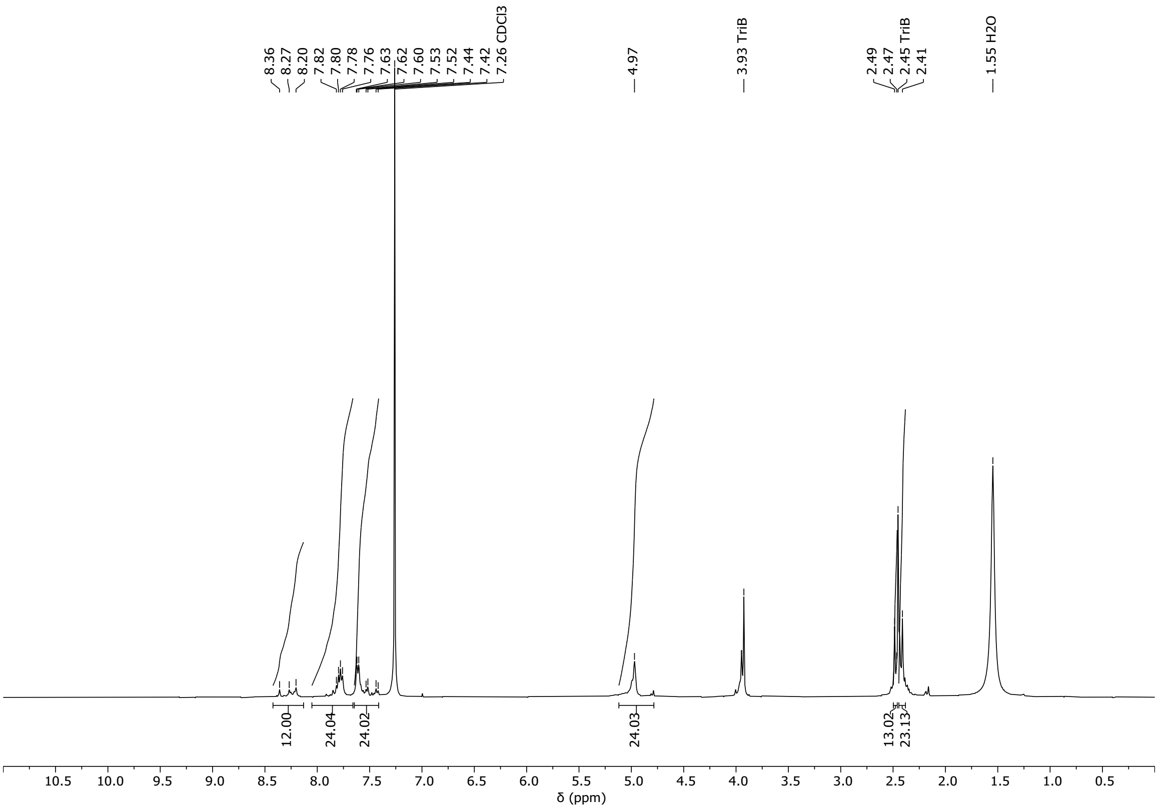

Figure S21: ^{1}H NMR ($CDCl_3$) spectrum for **B6$_{[4+6]}$**

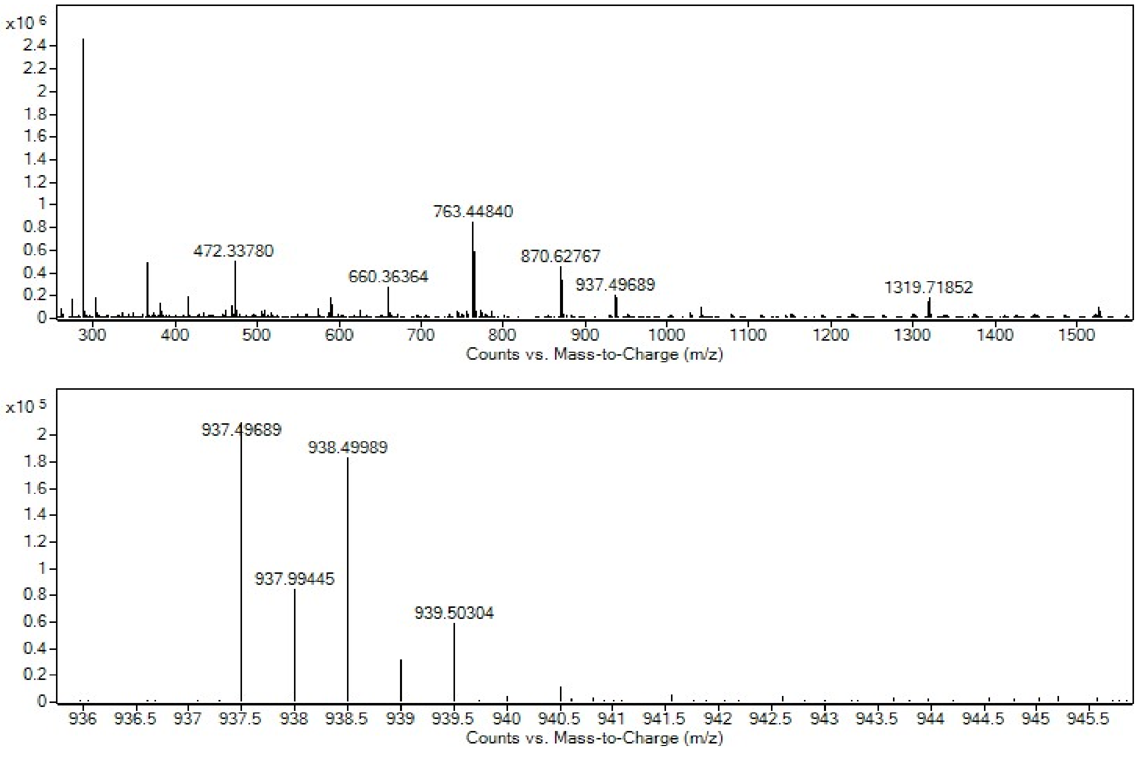

Figure S22: HRMS spectrum for **B6$_{[4+6]}$**

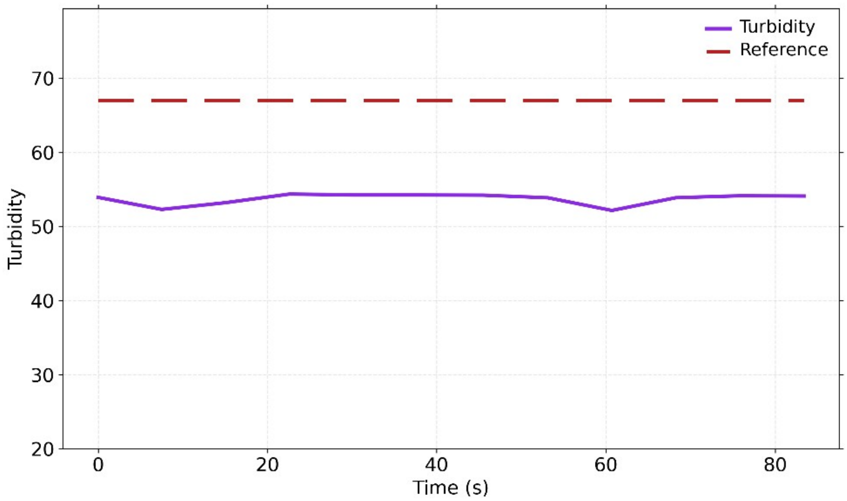

Figure S23: Turbidity vs time (s) for **B6$_{[4+6]}$**

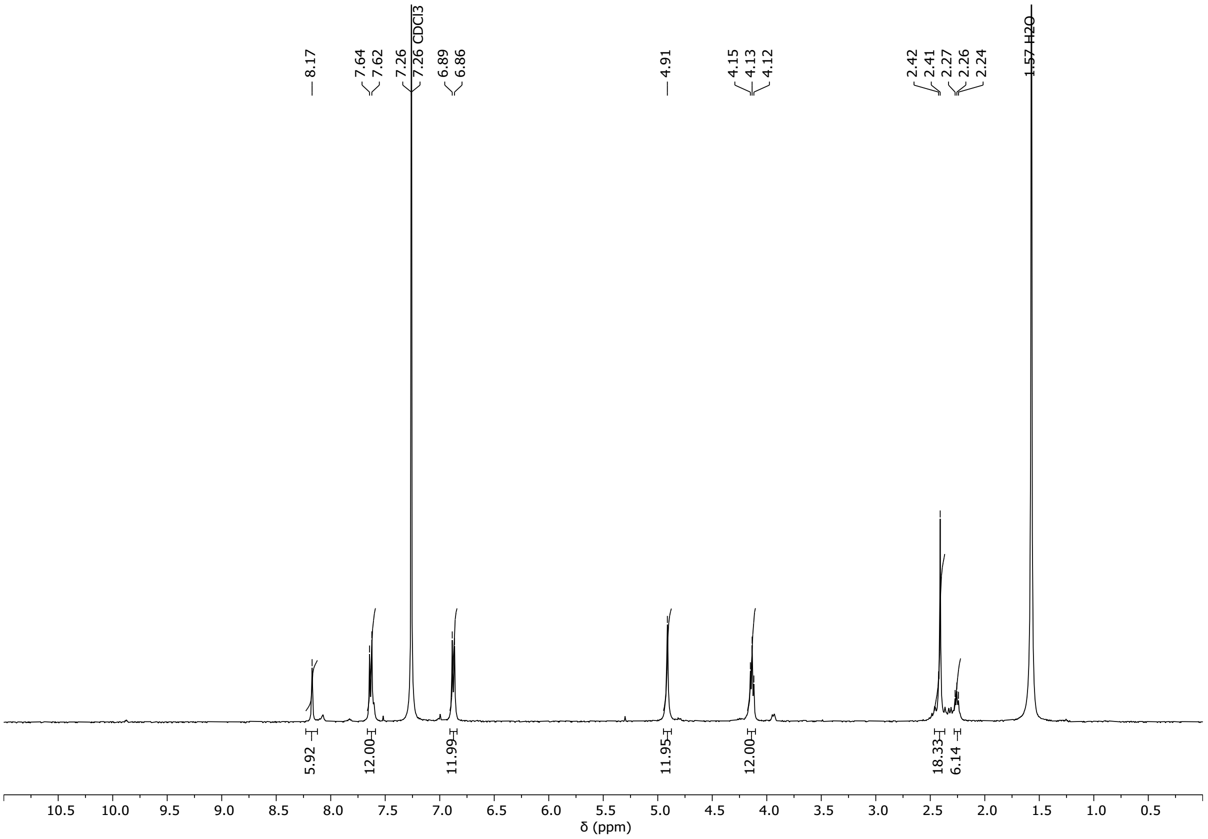

Figure S24: ^{1}H NMR ($CDCl_3$) spectrum for **B15$_{[2+3]}$**

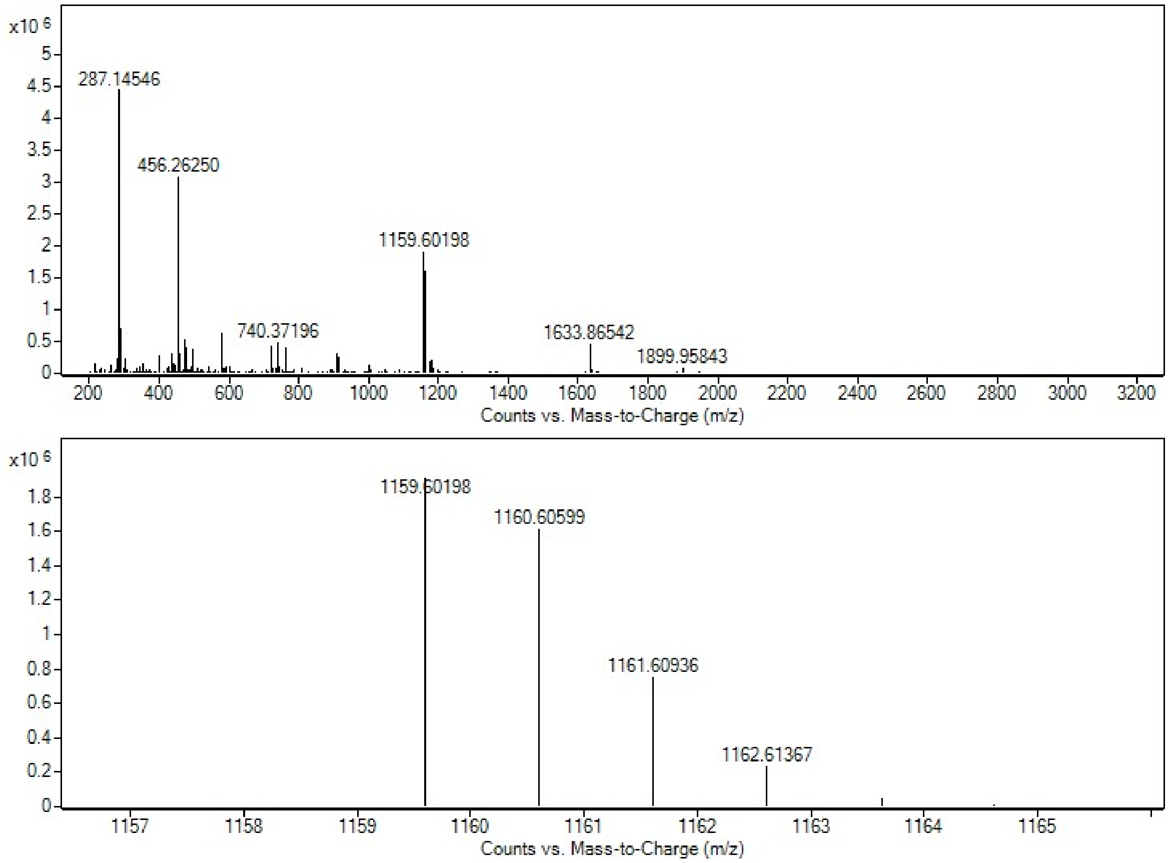

Figure S25: HRMS spectrum for **B15$_{[2+3]}$**

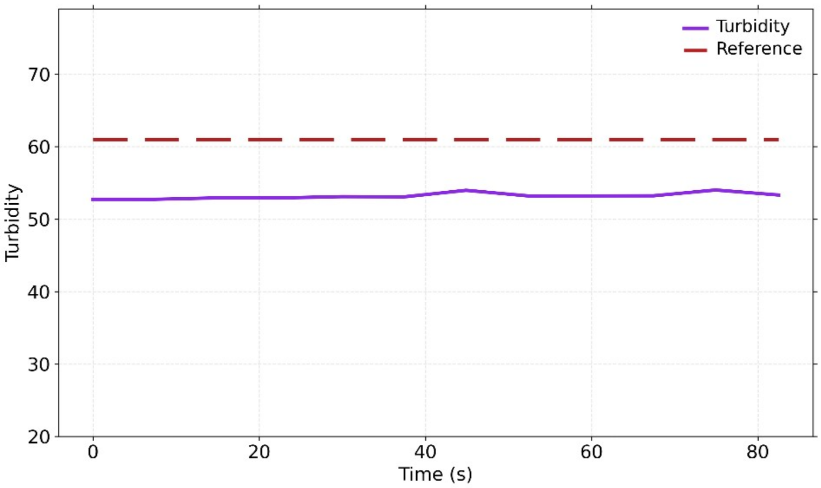

Figure S26: Turbidity vs time (s) for **B15$_{[2+3]}$**

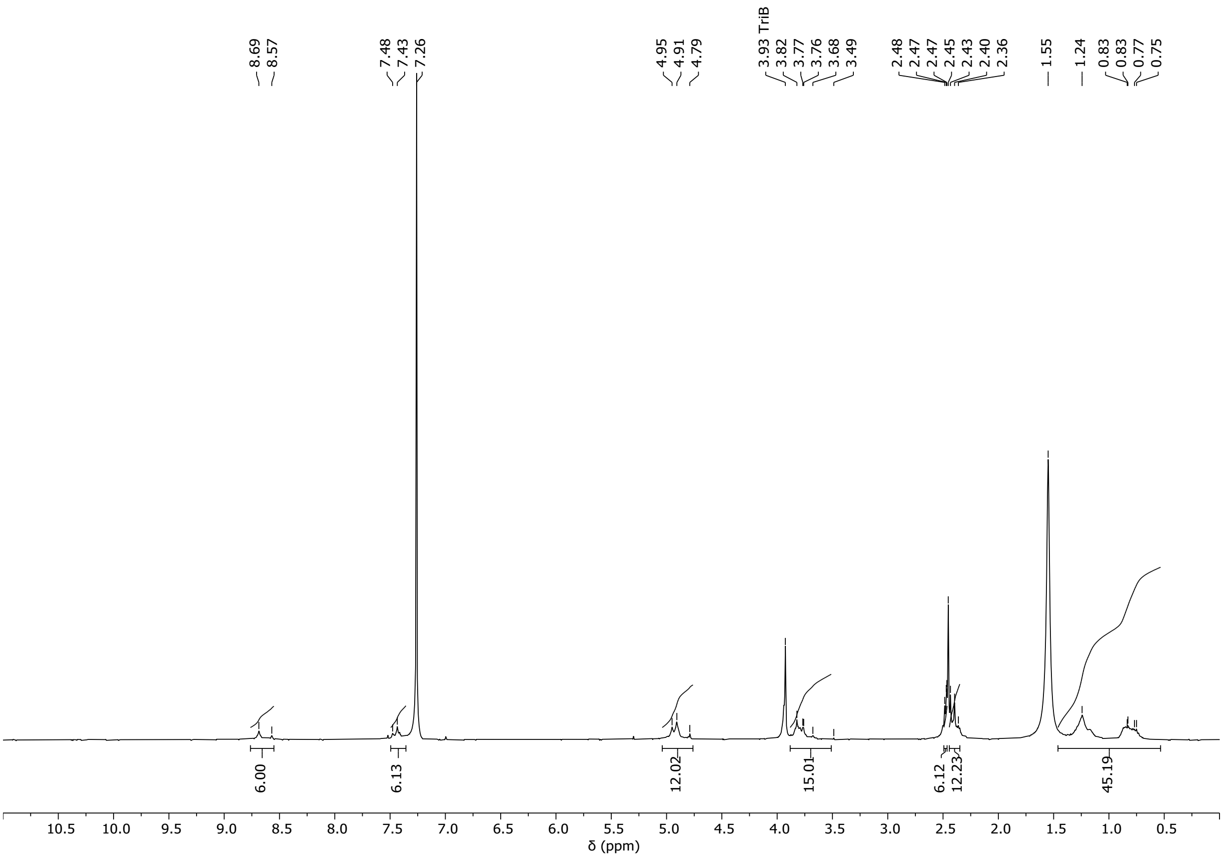

Figure S27: ^{1}H NMR ($CDCl_3$) spectrum for **B16$_{[4+6]}$**

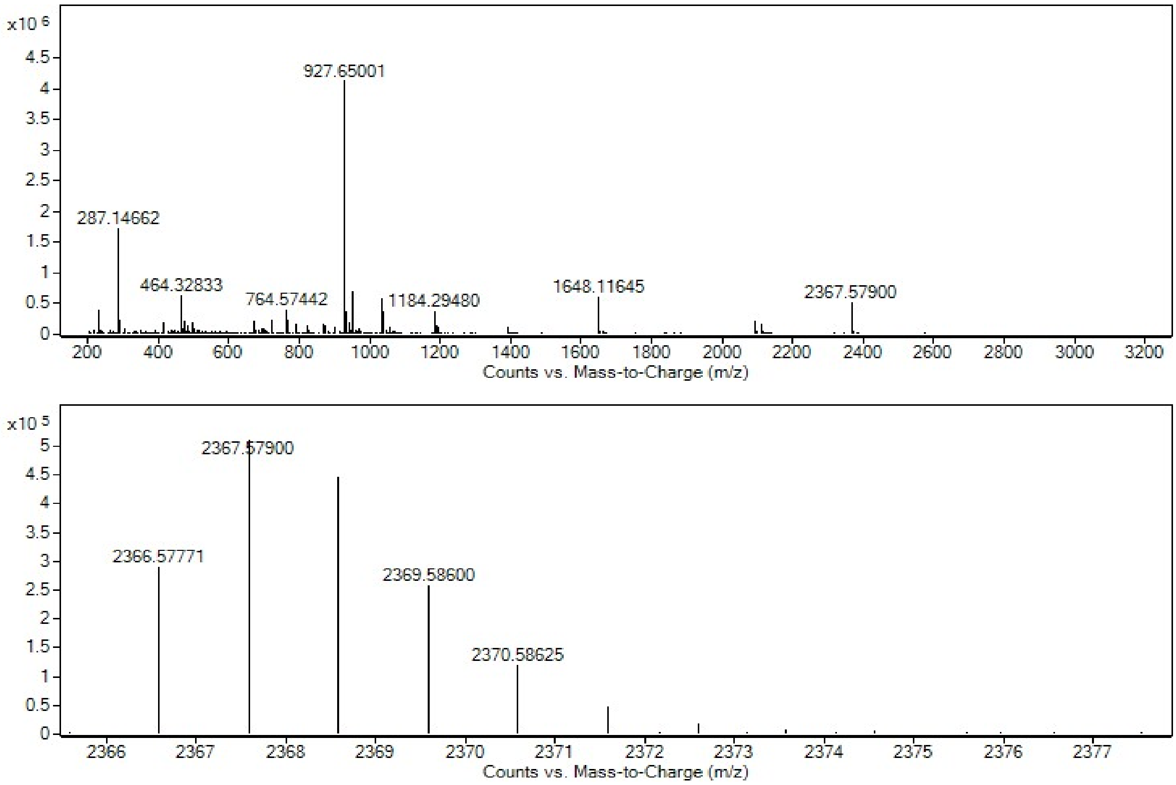

Figure S28: HRMS spectrum for **B16$_{[4+6]}$**

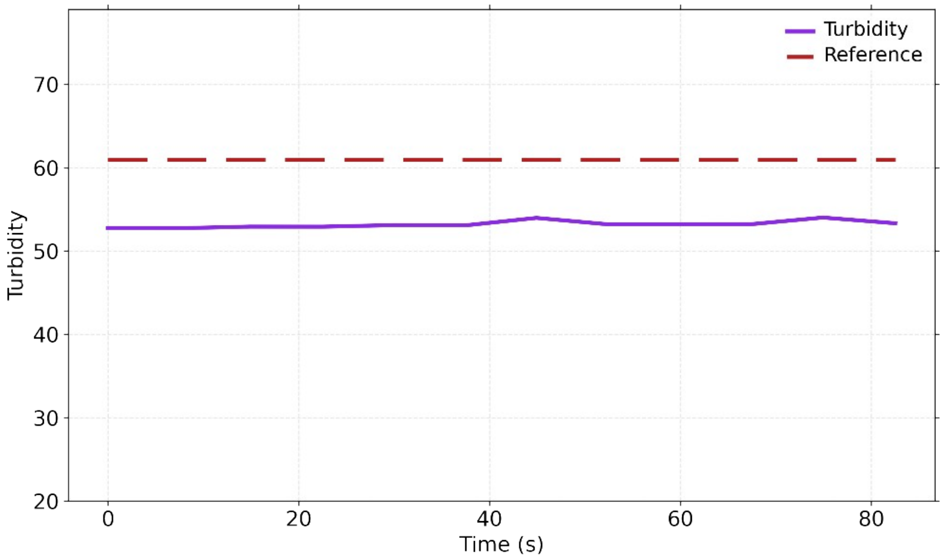

Figure S29: Turbidity vs time (s) for **B16$_{[4+6]}$**

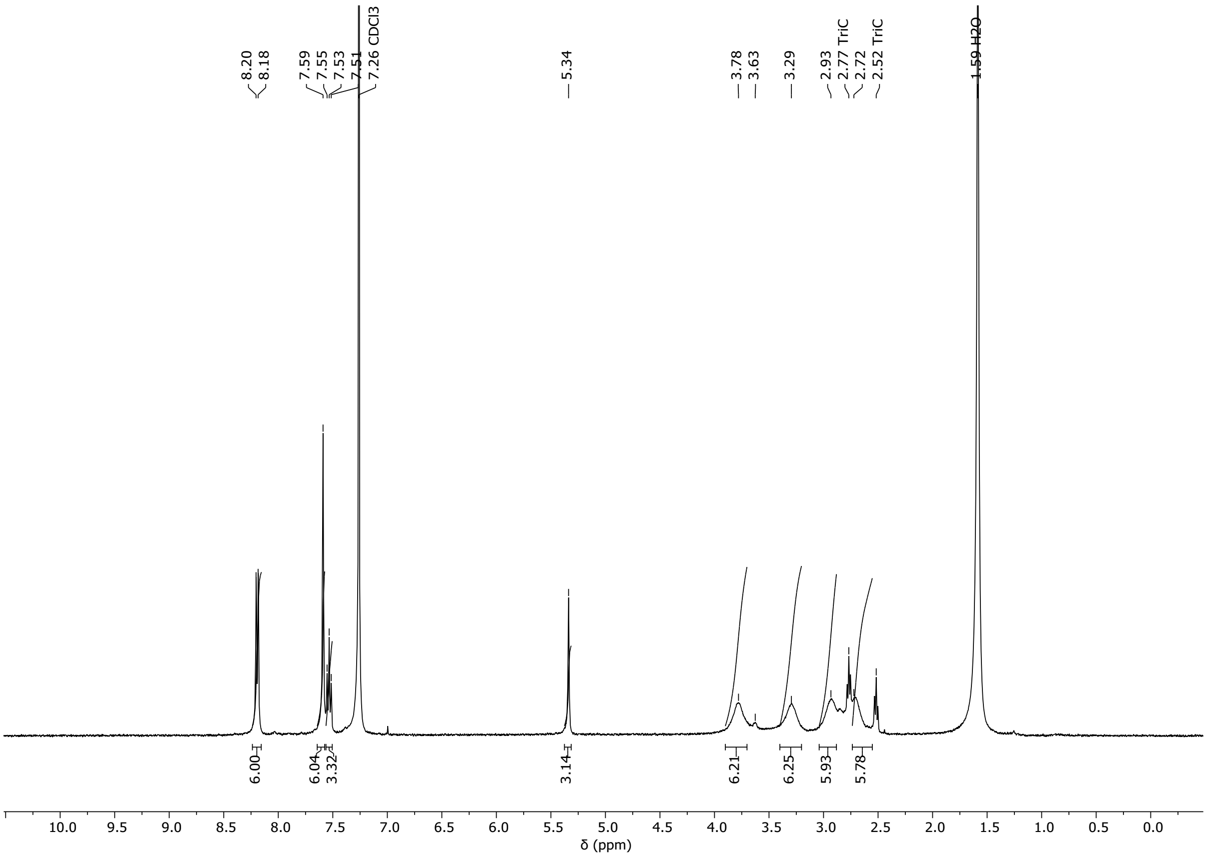

Figure S30: ^{1}H NMR ($CDCl_3$) spectrum for $\mathbf{C1_{[2+3]}}$

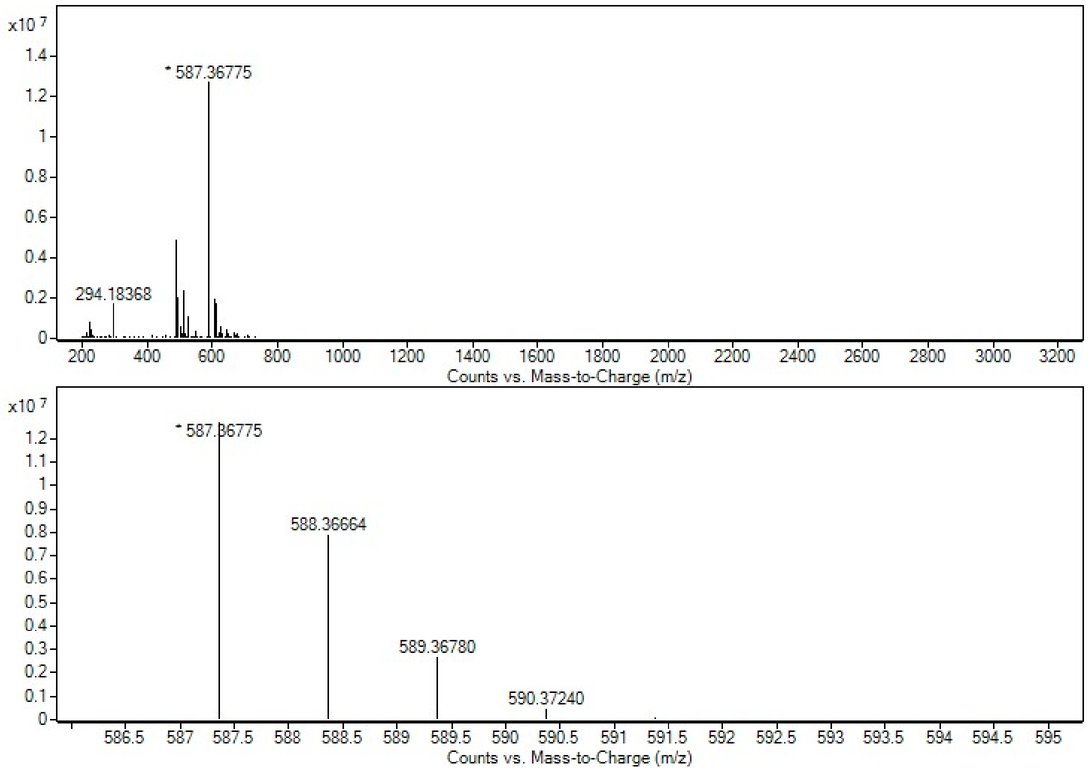

Figure S31: HRMS spectrum for $\mathbf{C1_{[2+3]}}$

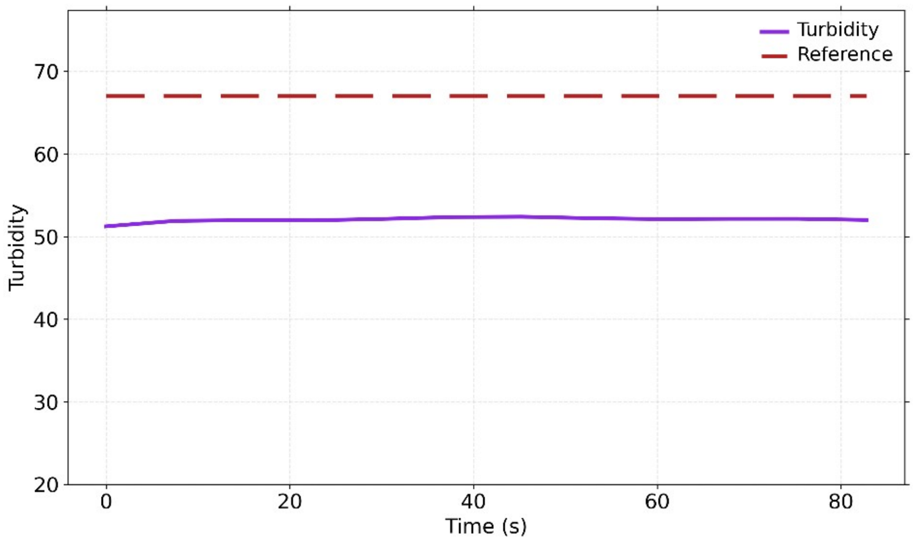

Figure S32: Turbidity vs time (s) for $\mathbf{C1_{[2+3]}}$

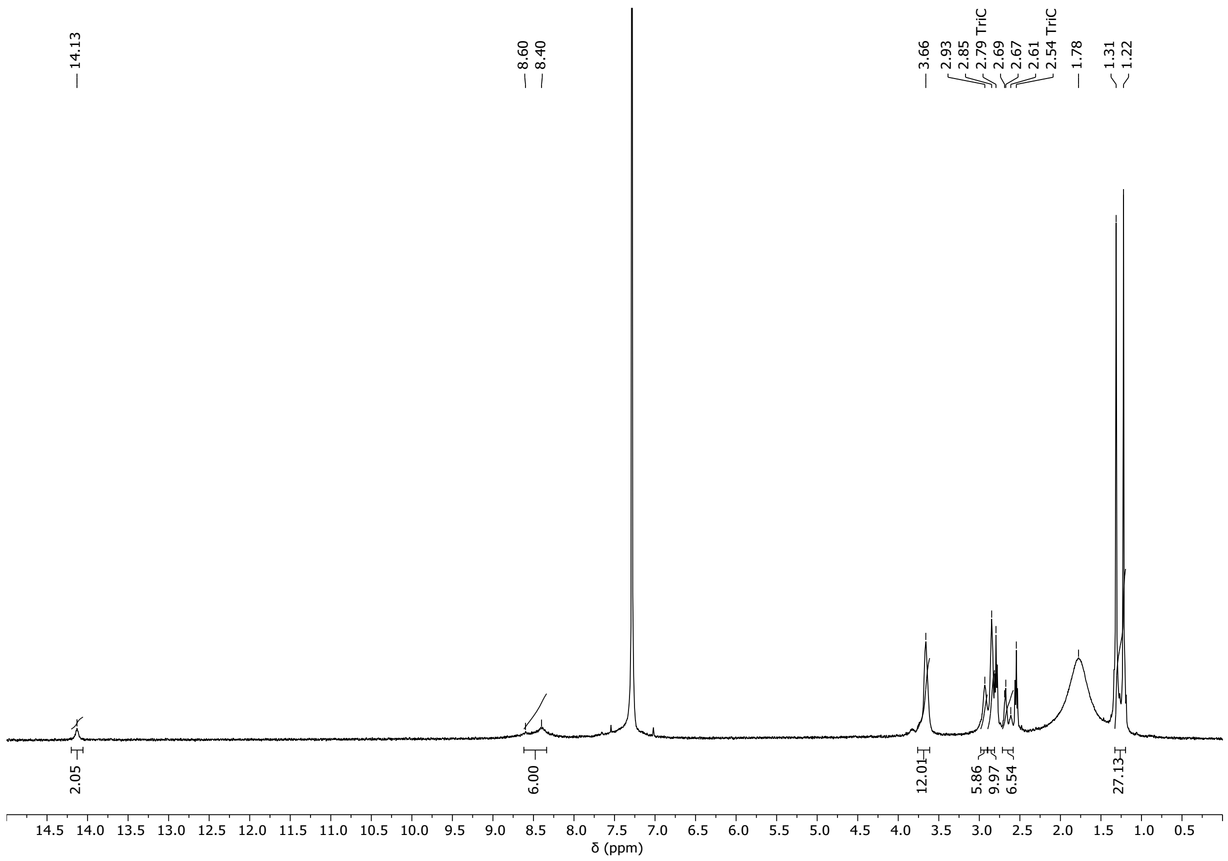

Figure S33: ^{1}H NMR ($CDCl_3$) spectrum for **$C2_{[2+3]}$**

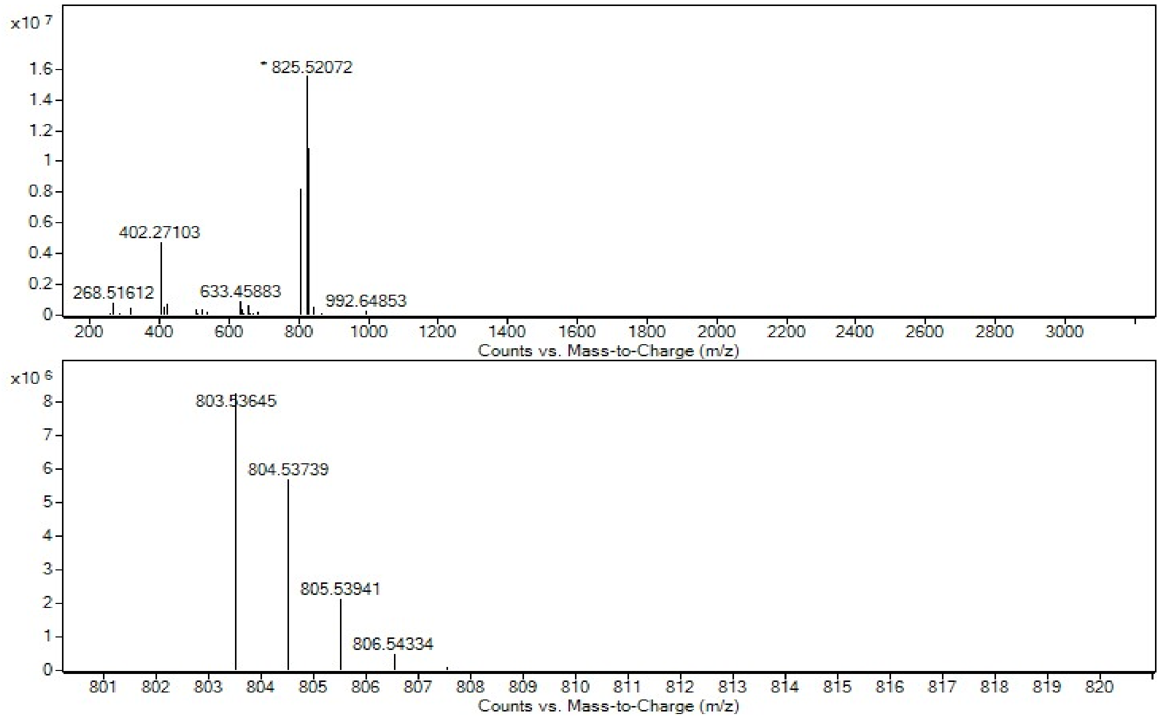

Figure S34: HRMS spectrum for **$C2_{[2+3]}$**

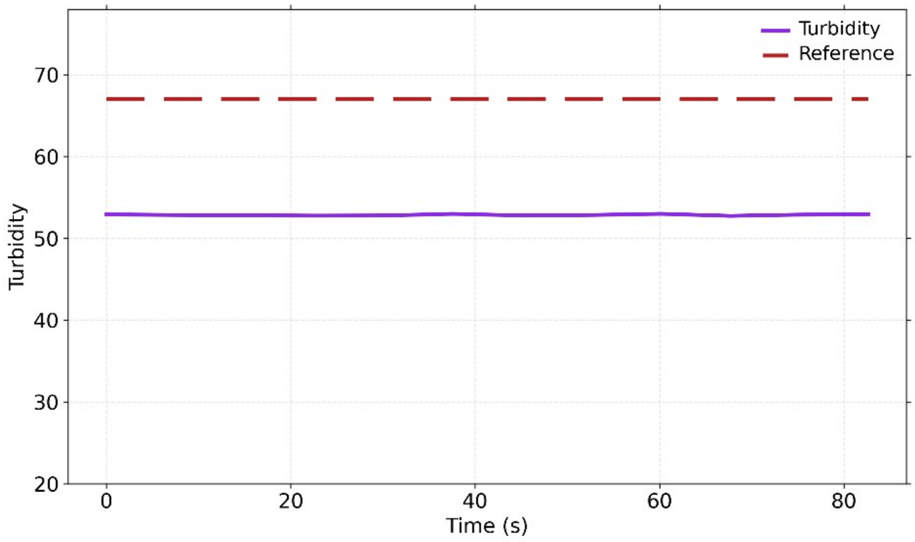

Figure S35: Turbidity vs time (s) for **$C2_{[2+3]}$**

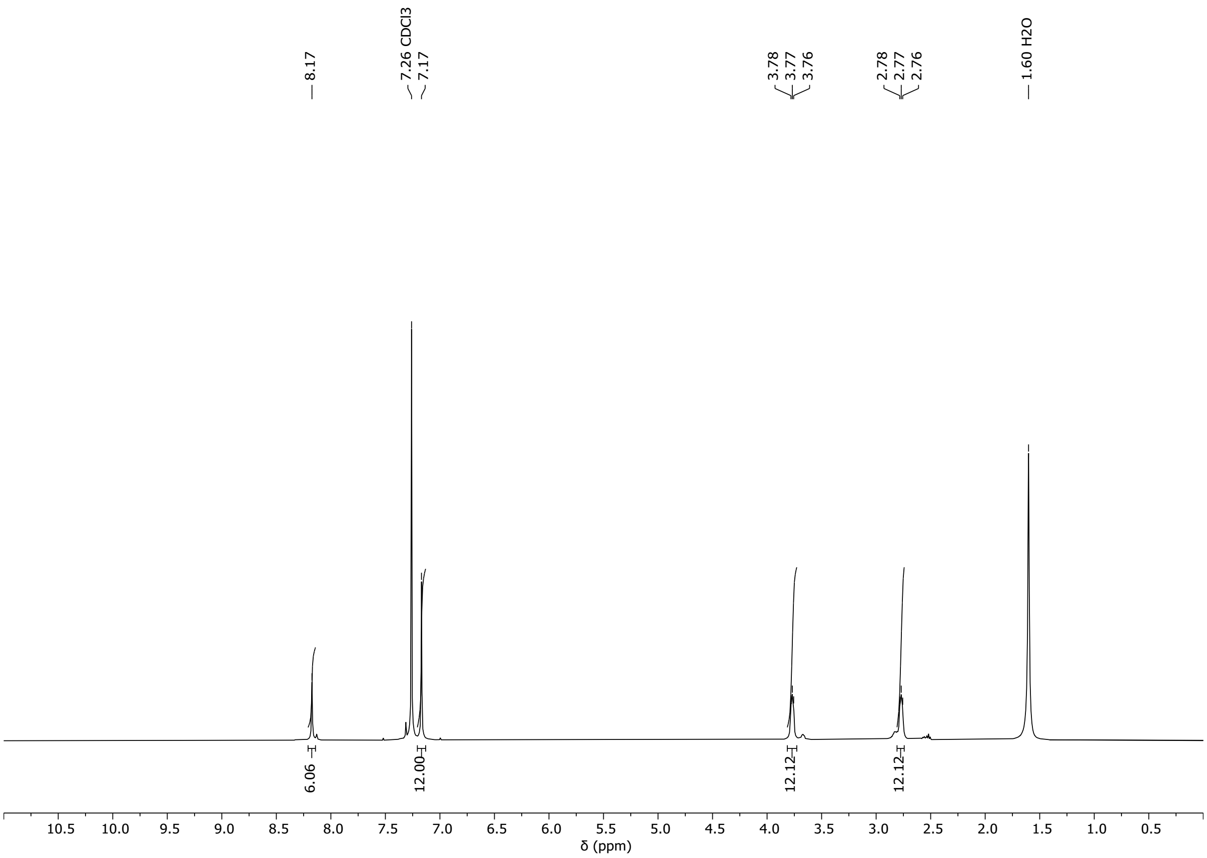

Figure S36: ^{1}H NMR ($CDCl_3$) spectrum for **$C4_{[2+3]}$**

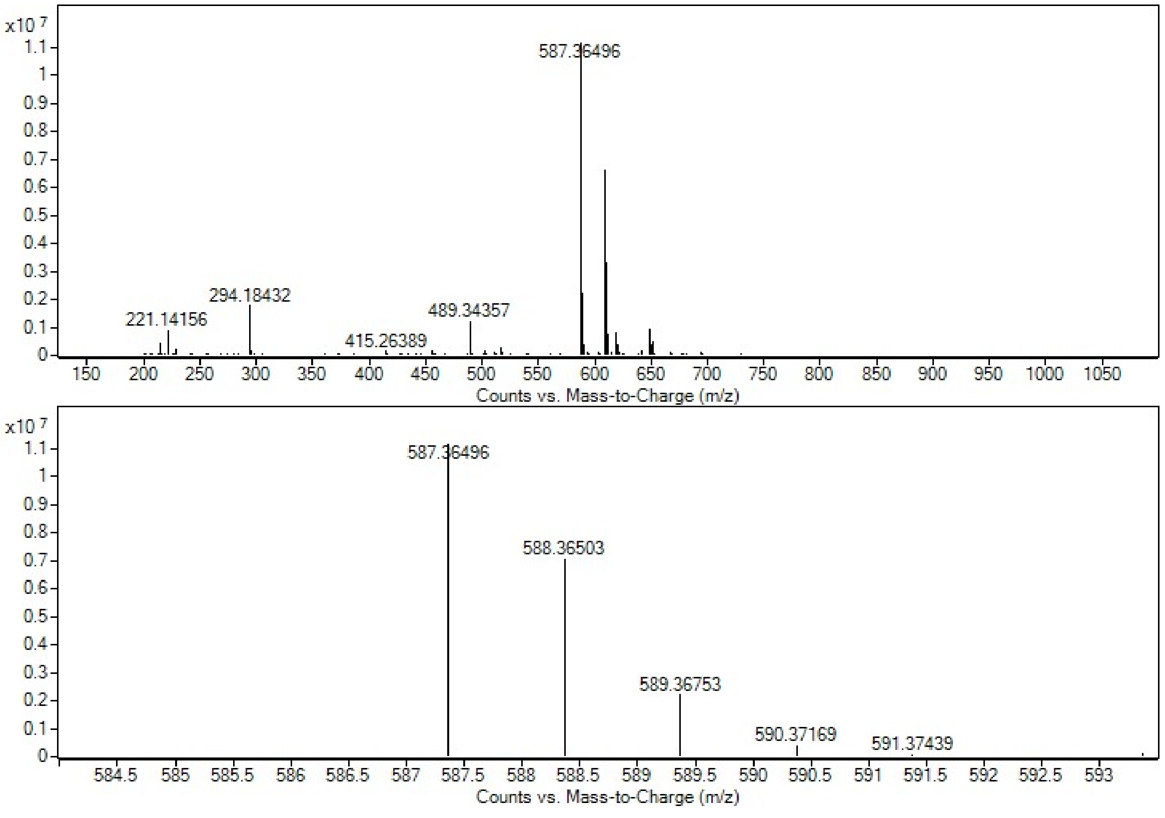

Figure S37: HRMS spectrum for **$C4_{[2+3]}$**

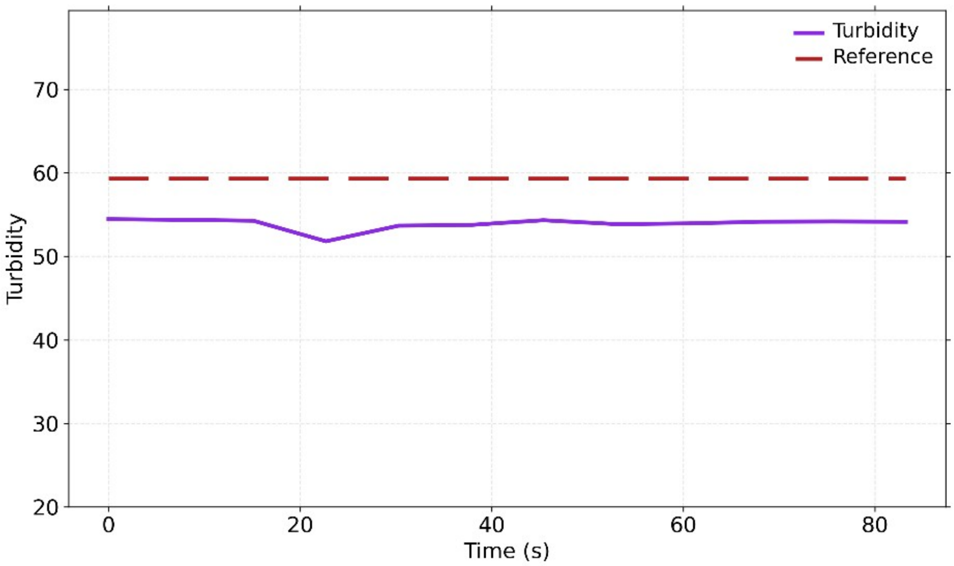

Figure S38: Turbidity vs time (s) for **C4$_{[2+3]}$**

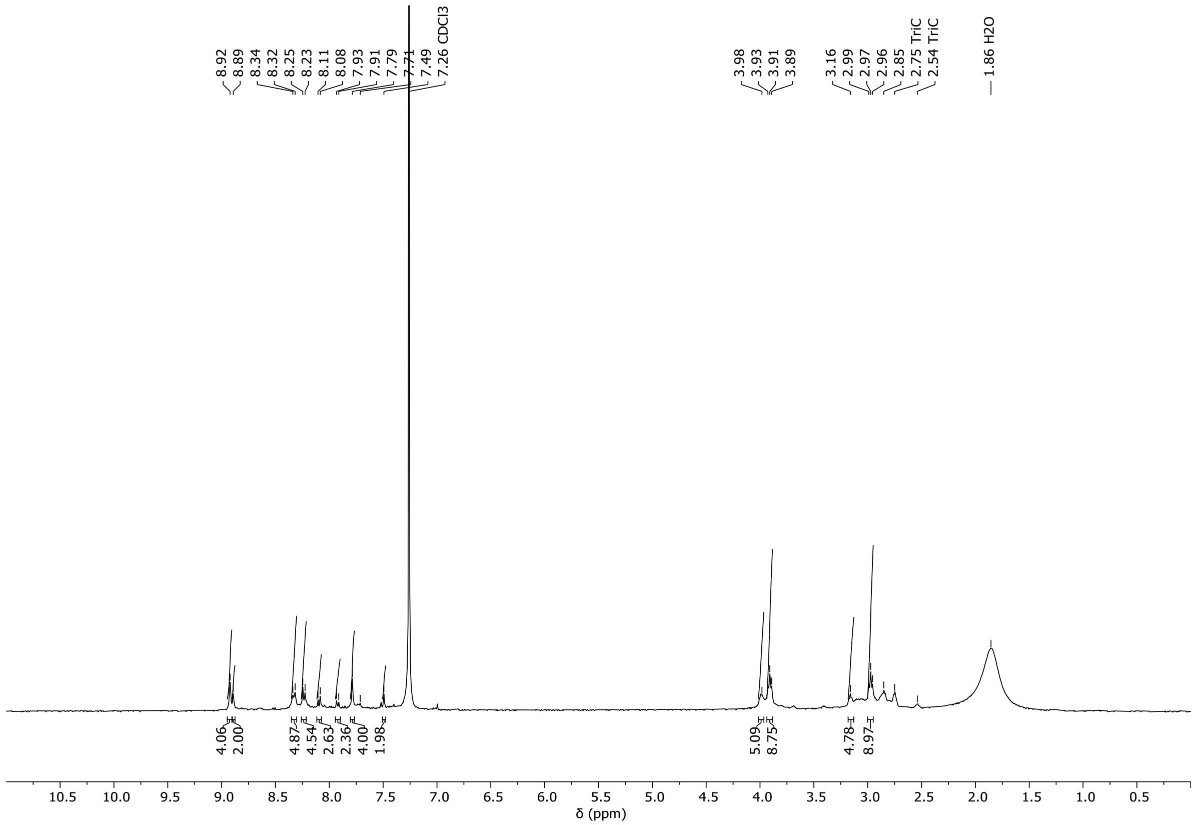

Figure S39: ^{1}H NMR ($CDCl_3$) spectrum for **$C8_{[2+3]}$**

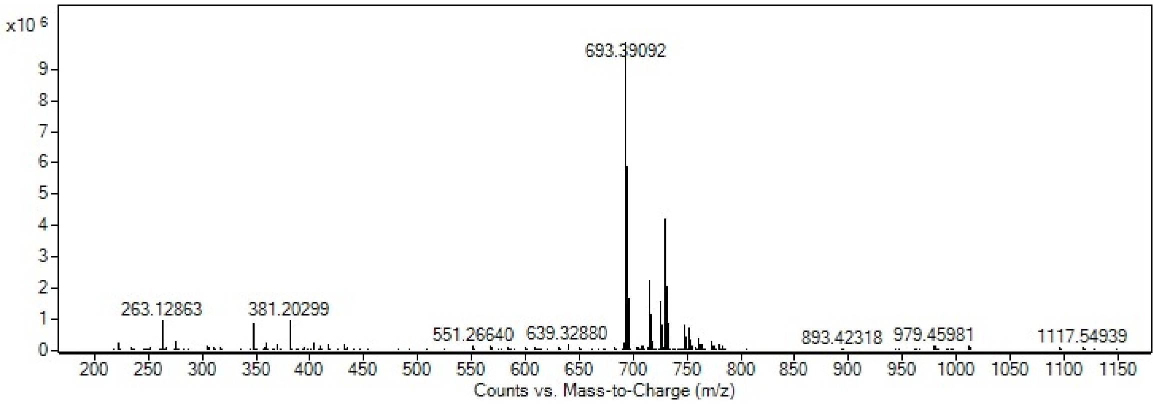

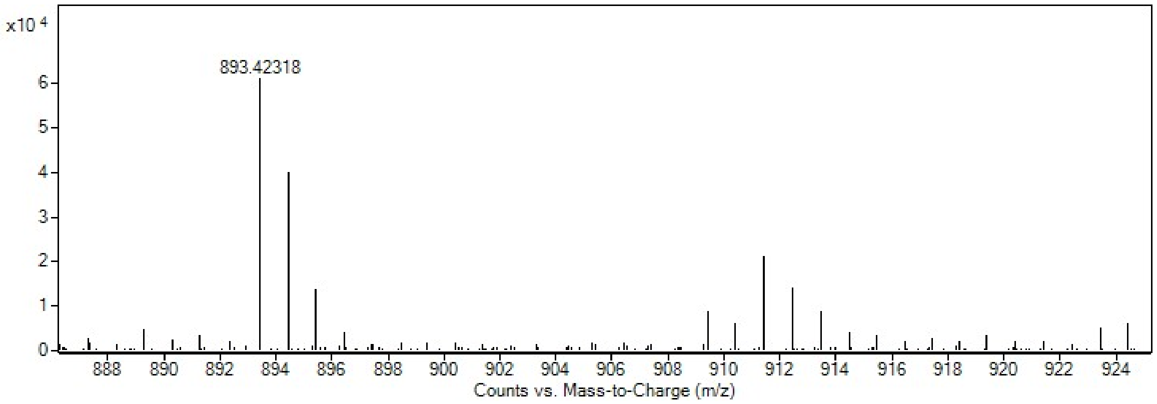

Figure S40: HRMS spectrum for **$C8_{[2+3]}$**

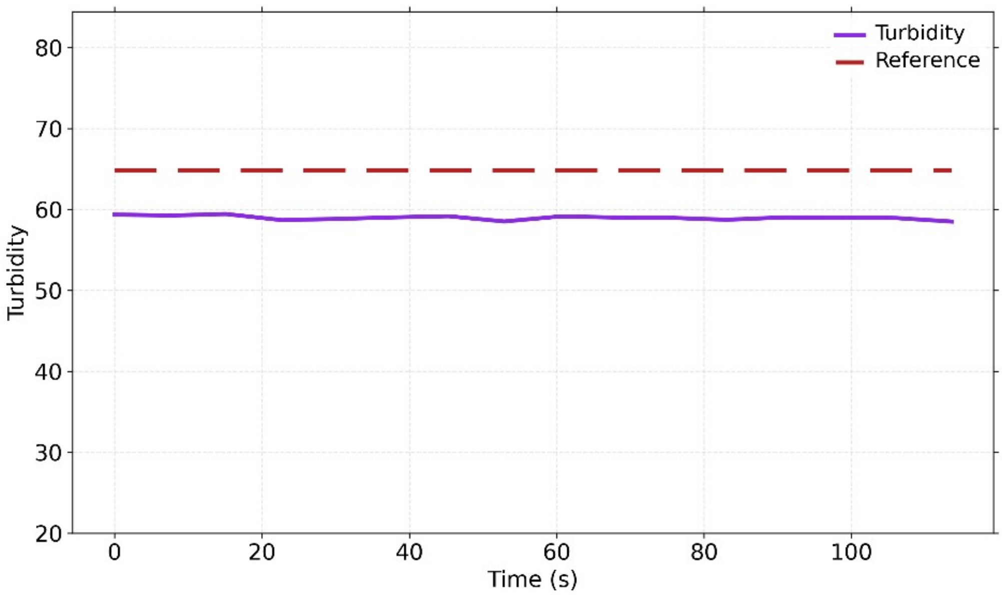

Figure S41: Turbidity vs time (s) for **C8$_{[2+3]}$**

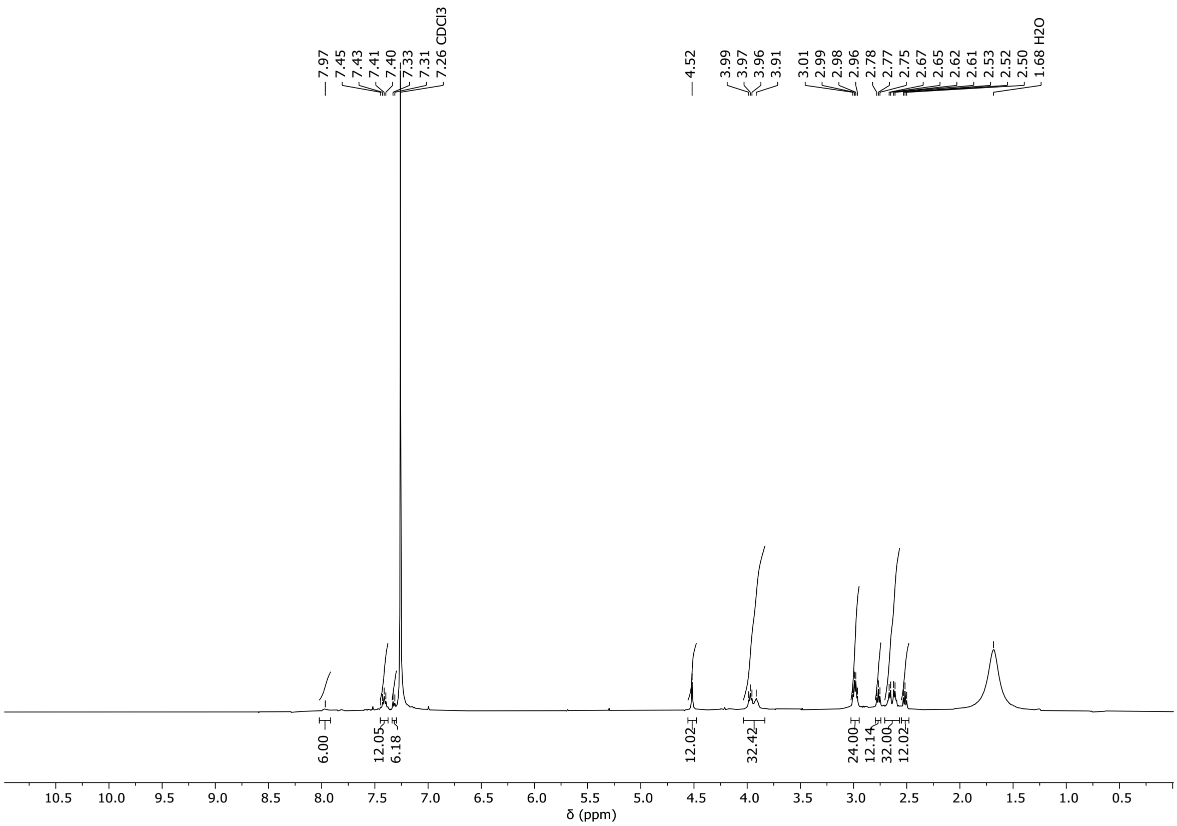

Figure S42: ^{1}H NMR ($CDCl_3$) spectrum for **$C10_{[2+3]}$**

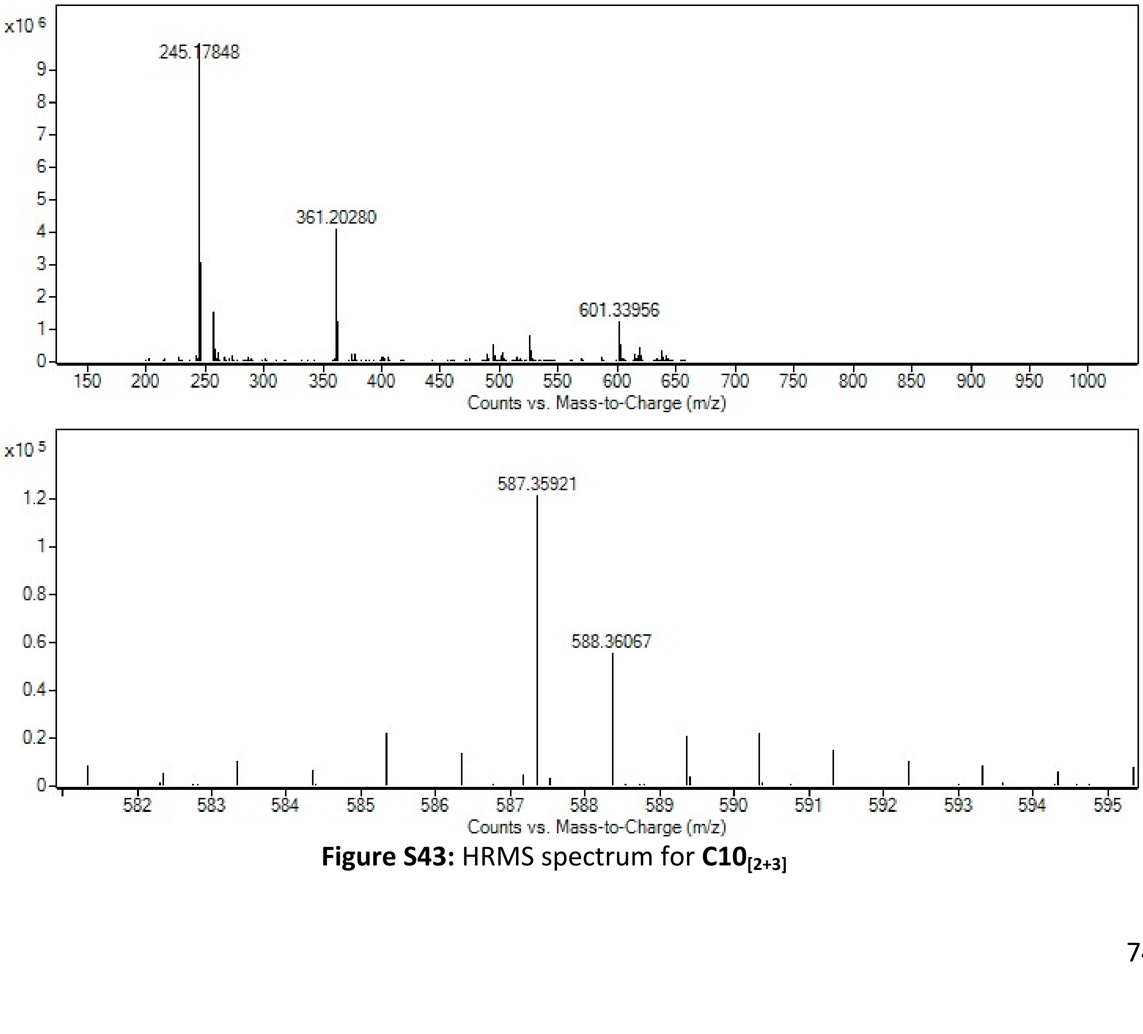

Figure S43: HRMS spectrum for **$C10_{[2+3]}$**

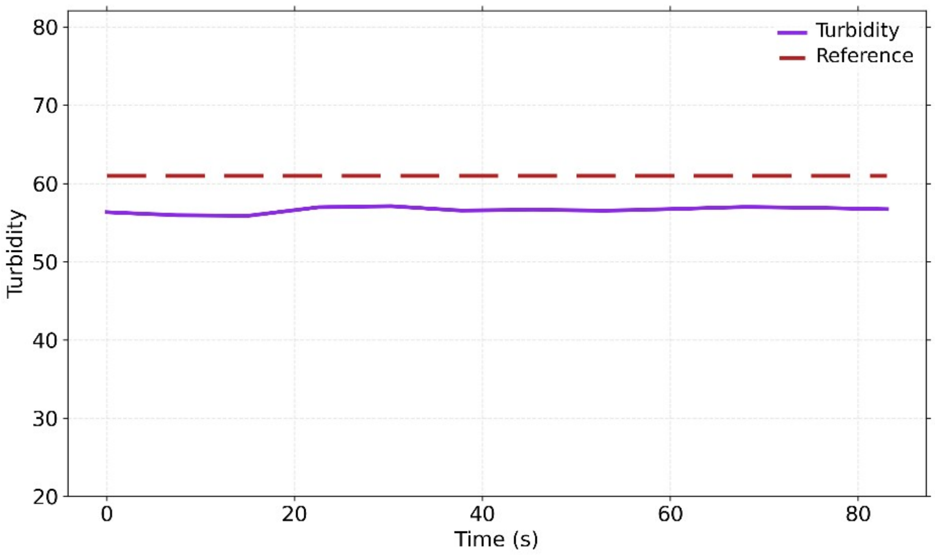

Figure S44: Turbidity vs time (s) for **C10$_{[2+3]}$**

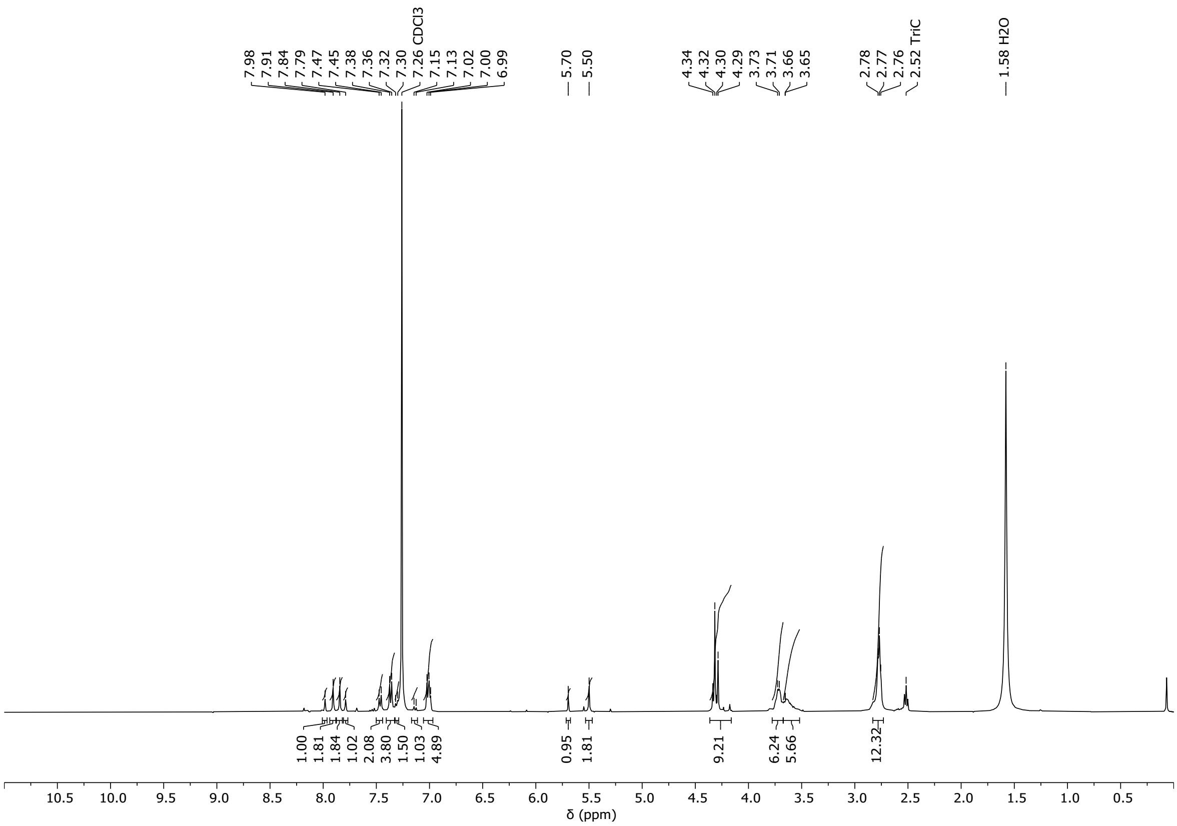

Figure S45: ^{1}H NMR ($CDCl_3$) spectrum for **$C11_{[2+3]}$**

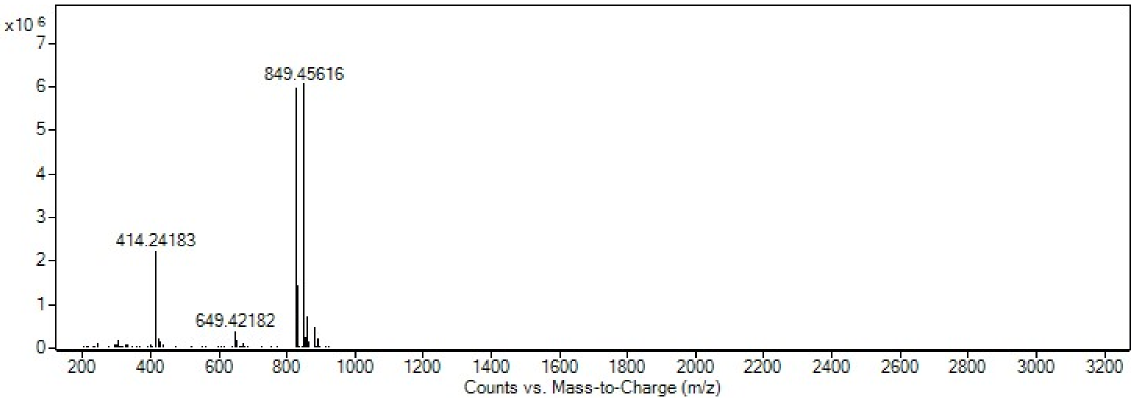

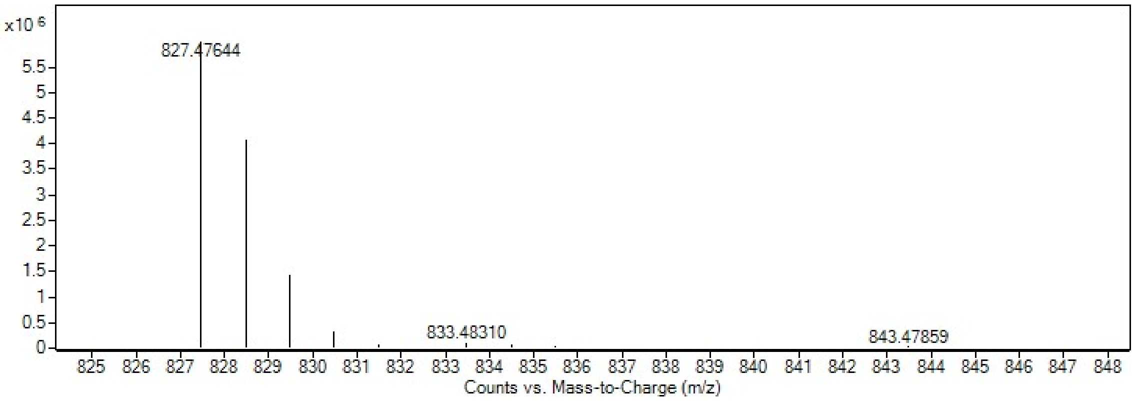

Figure S46: HRMS spectrum for **$C11_{[2+3]}$**

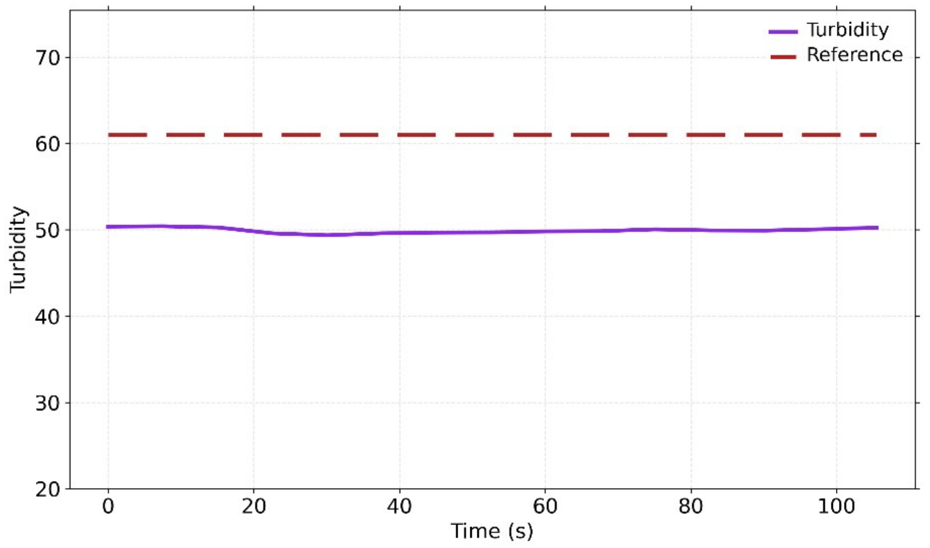

Figure S47: Turbidity vs time (s) for **C11$_{[2+3]}$**

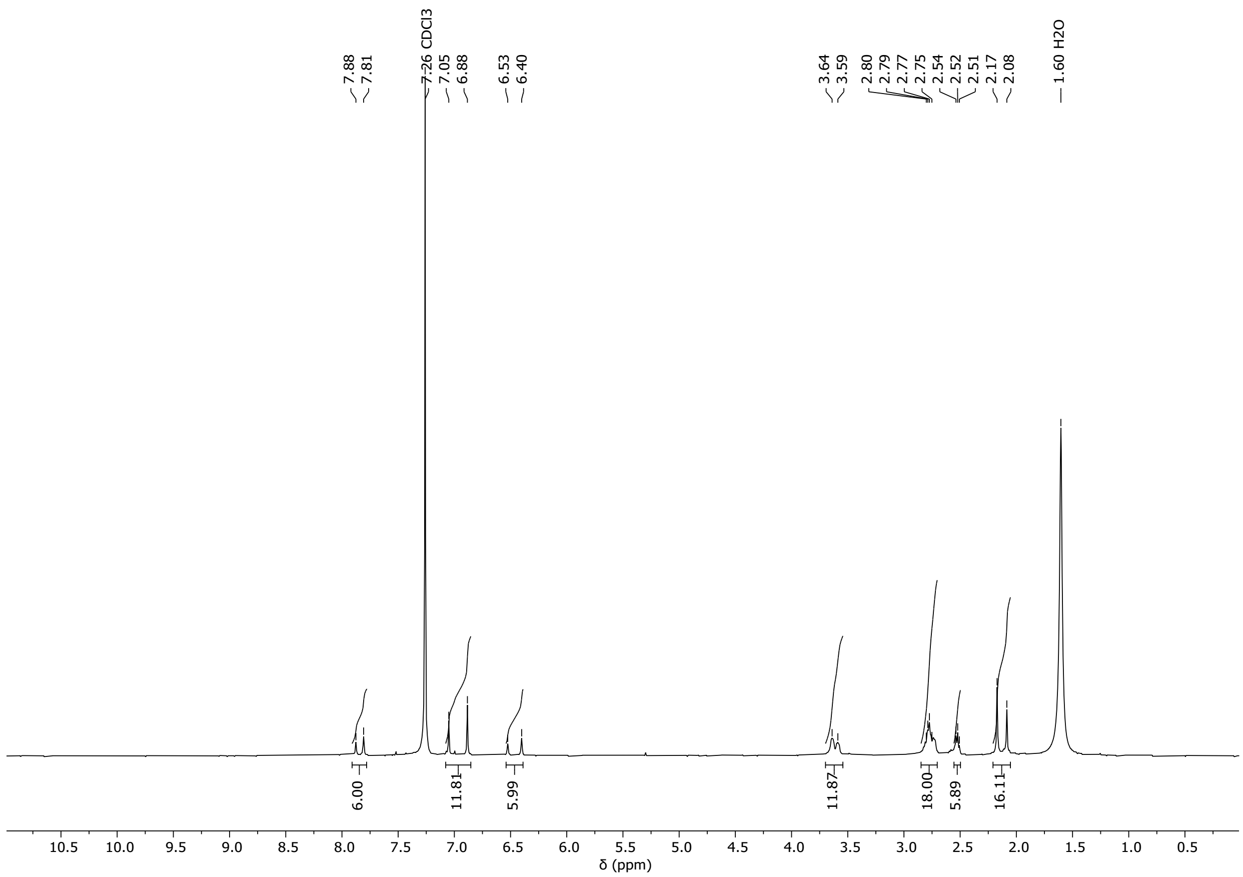

Figure S48: ^{1}H NMR ($CDCl_3$) spectrum for **C12**$_{[2+3]}$ - risk of competitive 1,4- vs 1,2-addition to the unsaturated aldehyde in **12**.

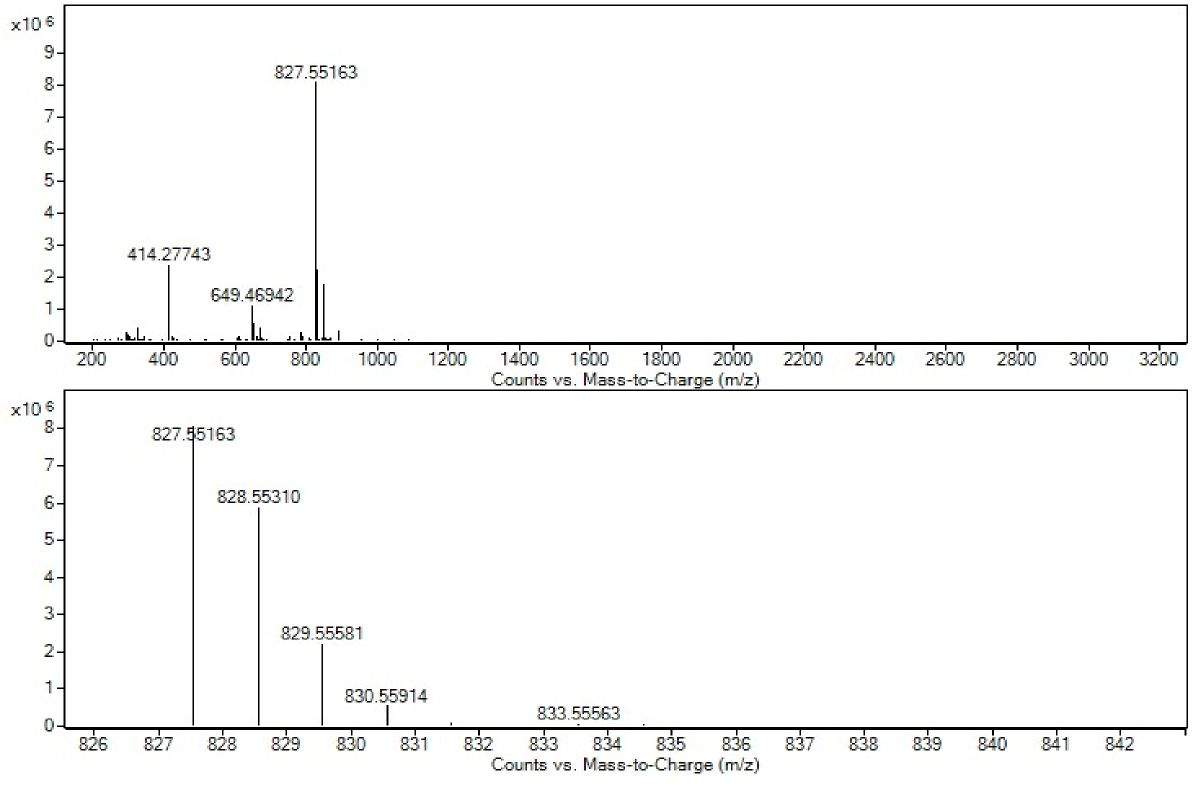

Figure S49: HRMS spectrum for $\mathbf{C12_{[2+3]}}$

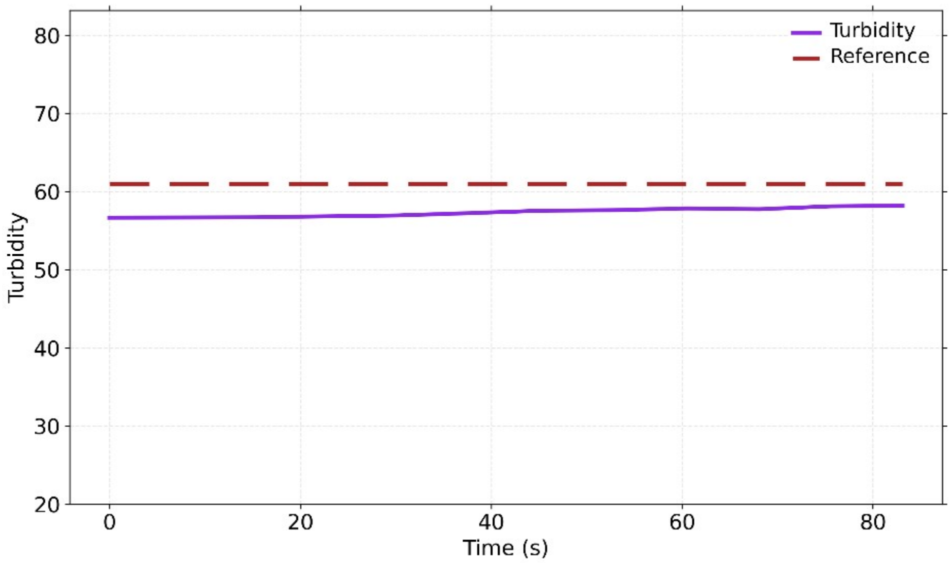

Figure S50: Turbidity vs time (s) for $\mathbf{C12_{[2+3]}}$

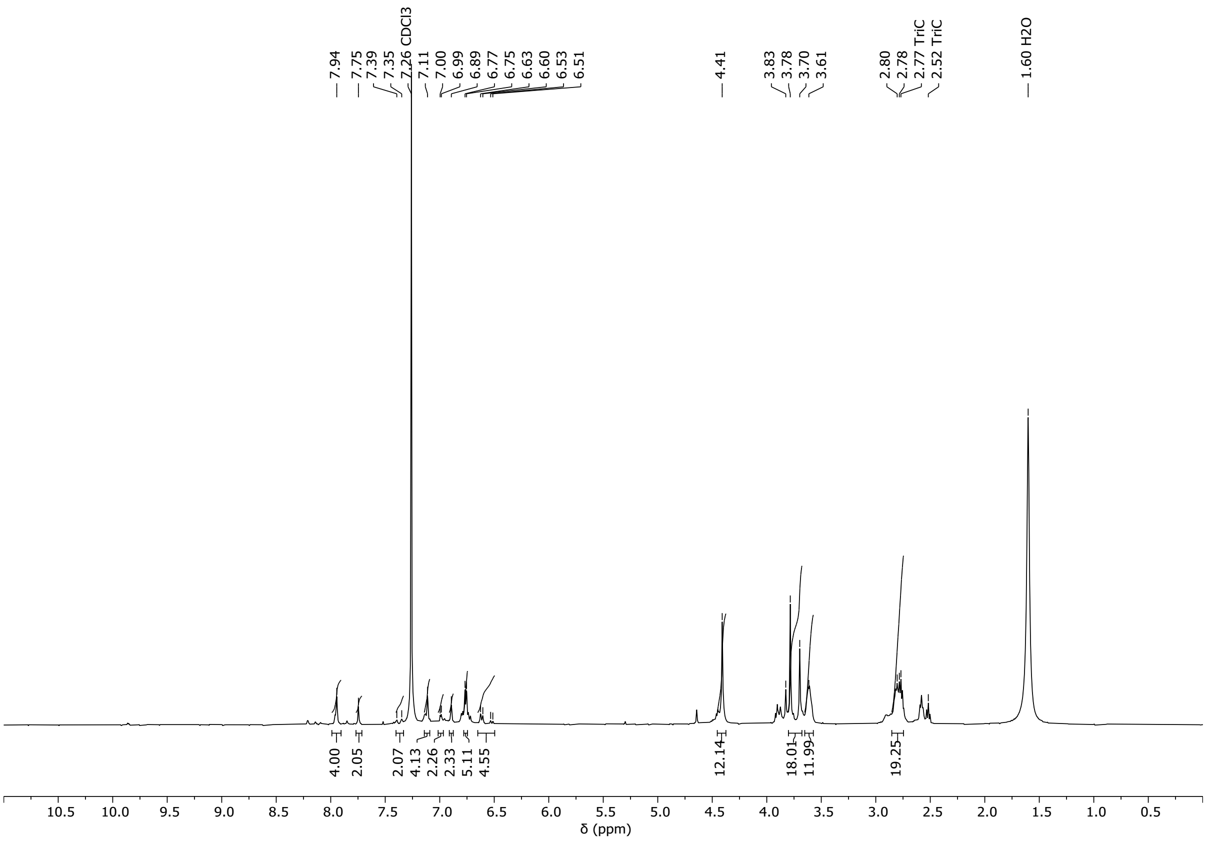

Figure S51: ^{1}H NMR ($CDCl_3$) spectrum for **C13$_{[2+3]}$**

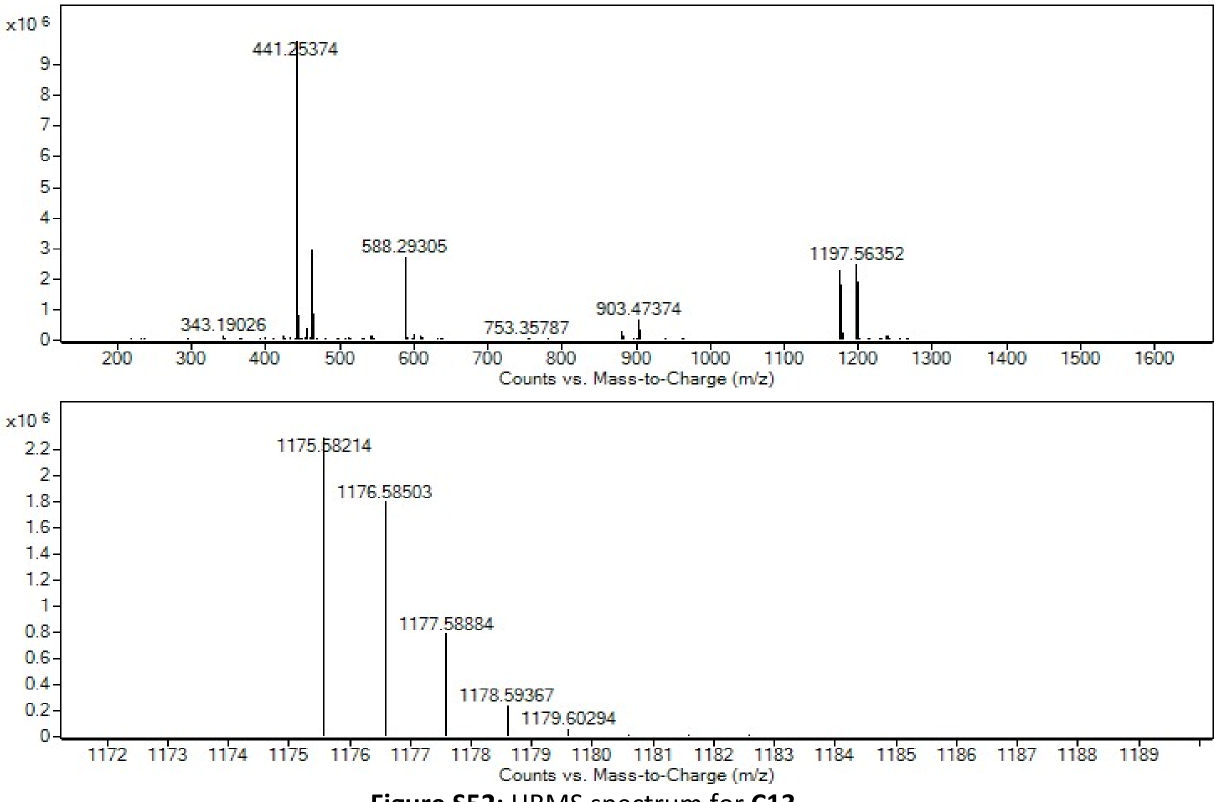

Figure S52: HRMS spectrum for **C13$_{[2+3]}$**

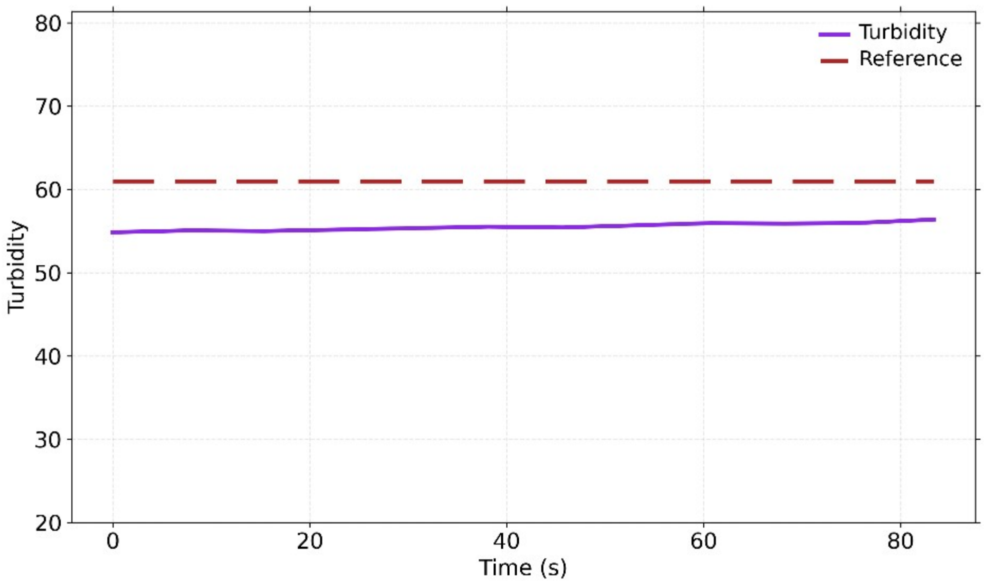

Figure S53: Turbidity vs time (s) for **C13**$_{[2+3]}$

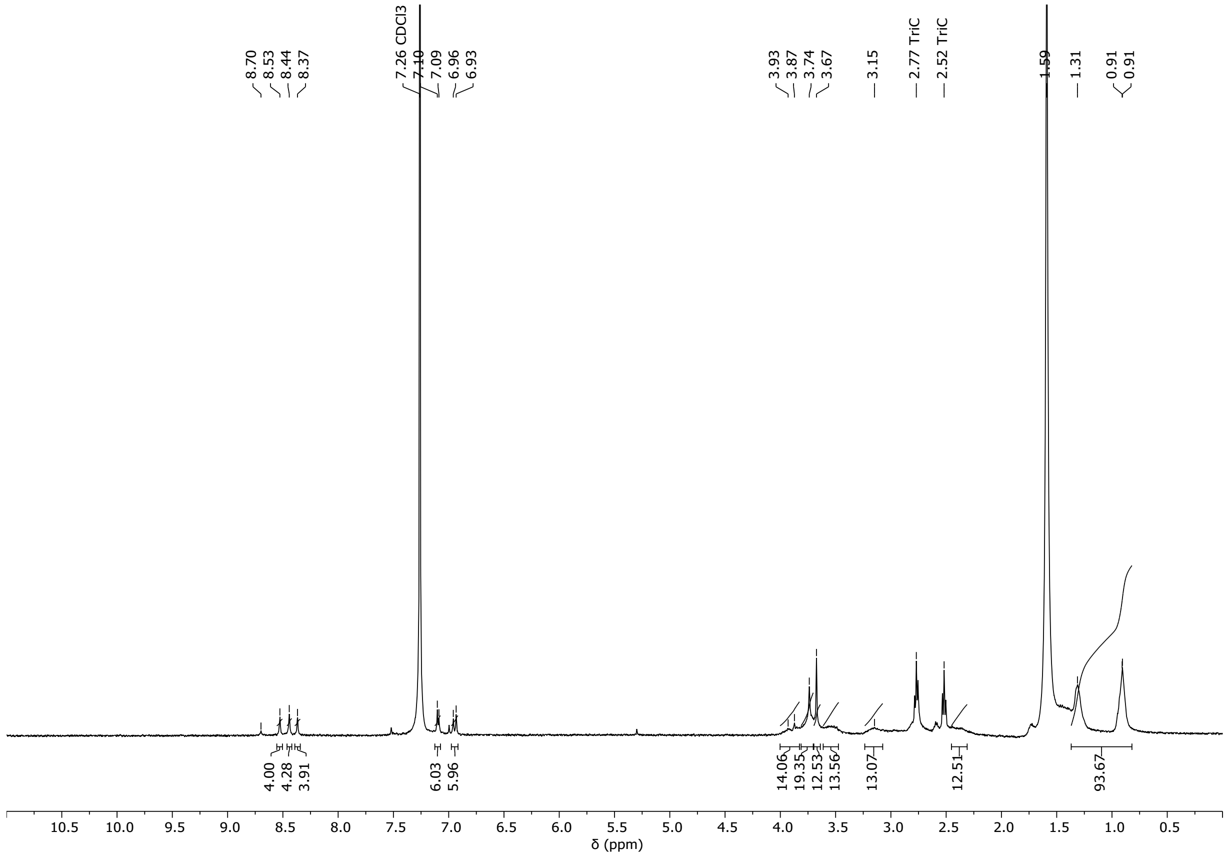

Figure S54: ^{1}H NMR ($CDCl_3$) spectrum for **C16$_{[2+3]}$**

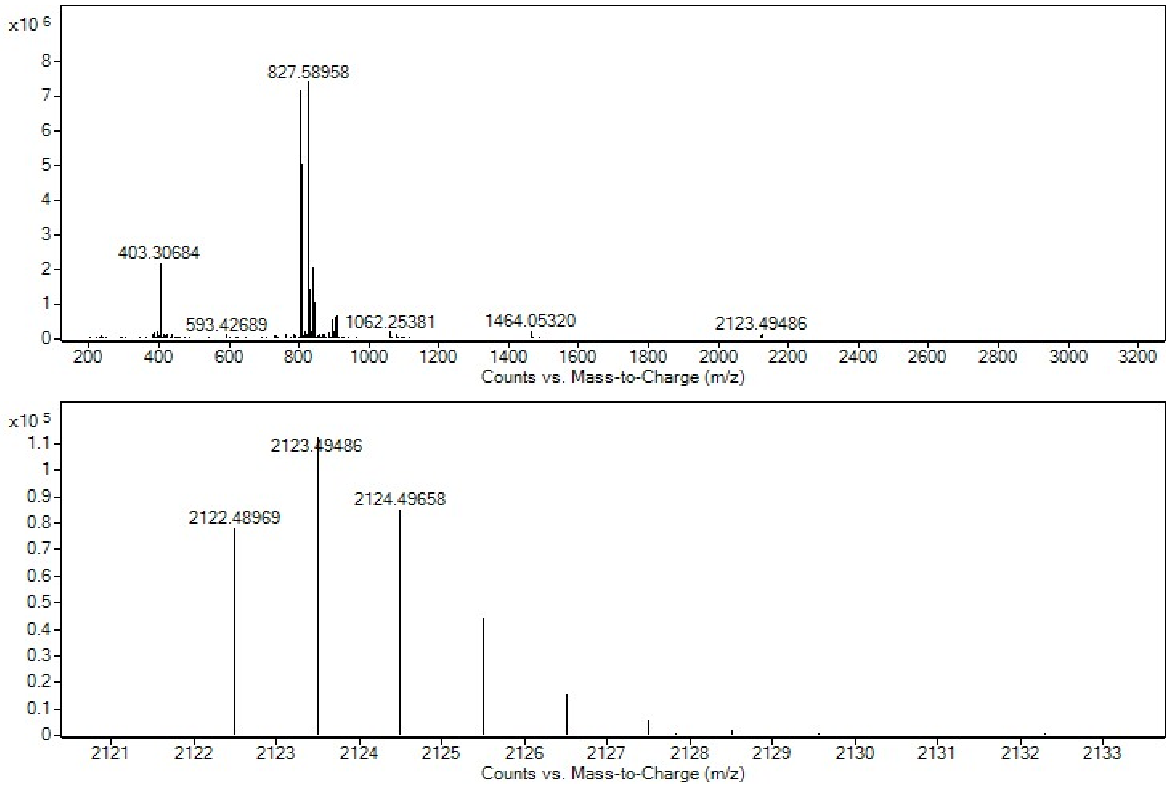

Figure S55: HRMS spectrum for **C16$_{[2+3]}$**

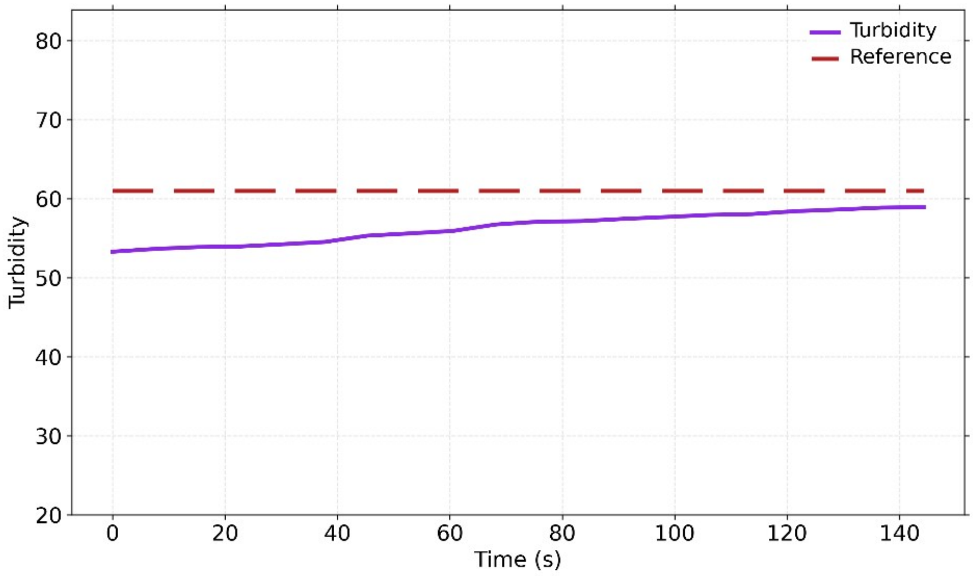

Figure S56: Turbidity vs time (s) for **C16$_{[4+6]}$**

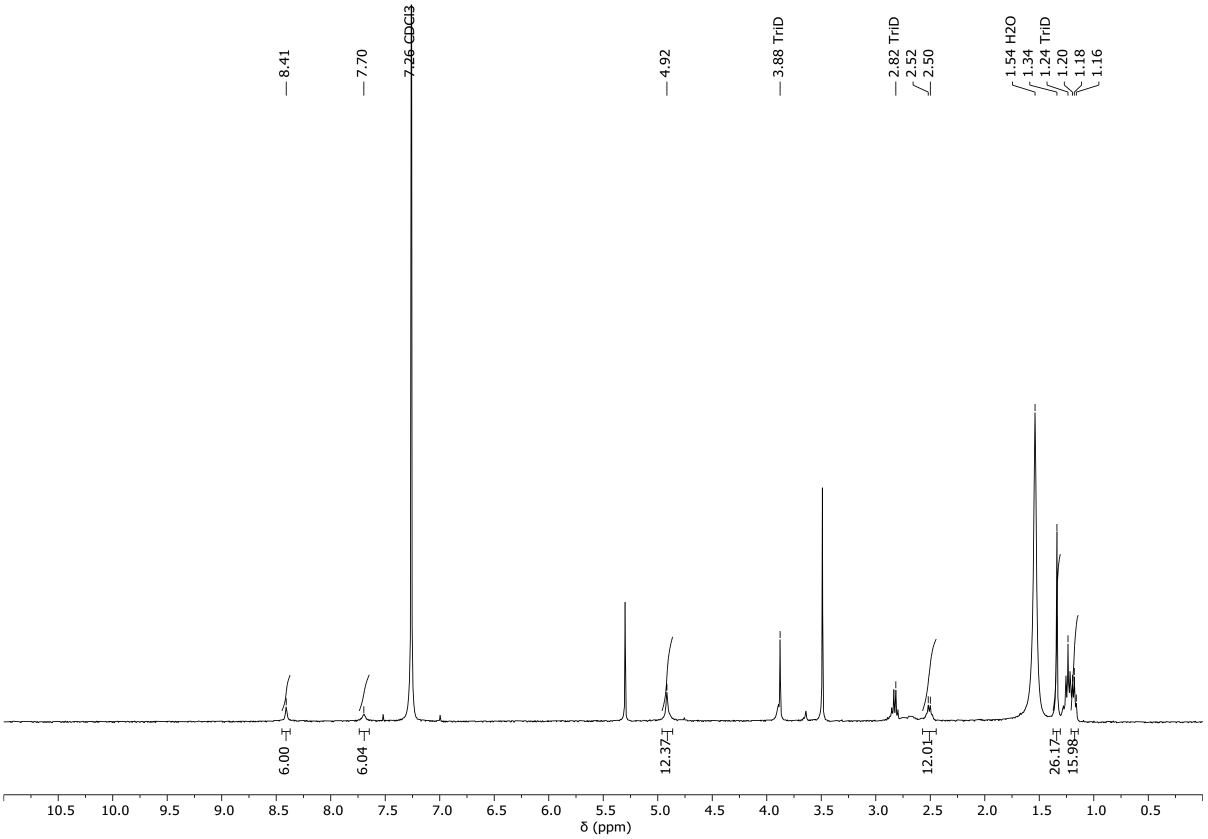

Figure S57: ^{1}H NMR ($CDCl_3$) spectrum for **$D2_{[2+3]}$**

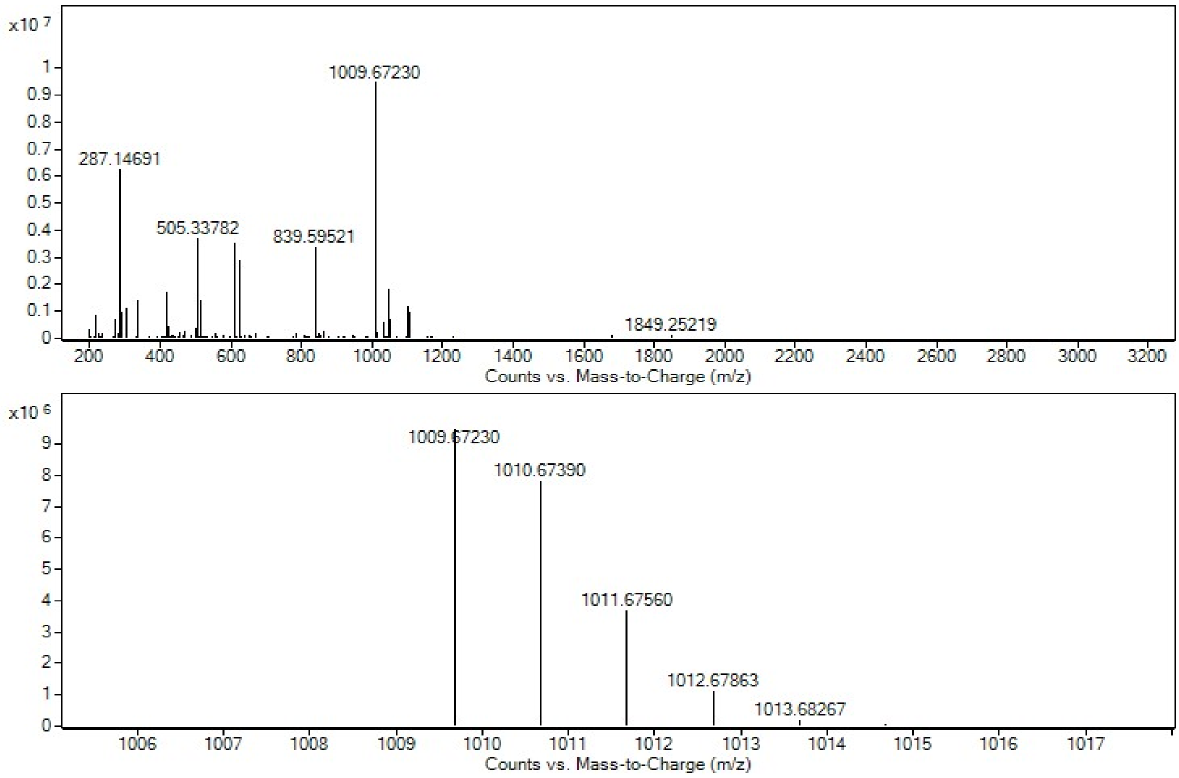

Figure S58: HRMS spectrum for **$D2_{[2+3]}$**

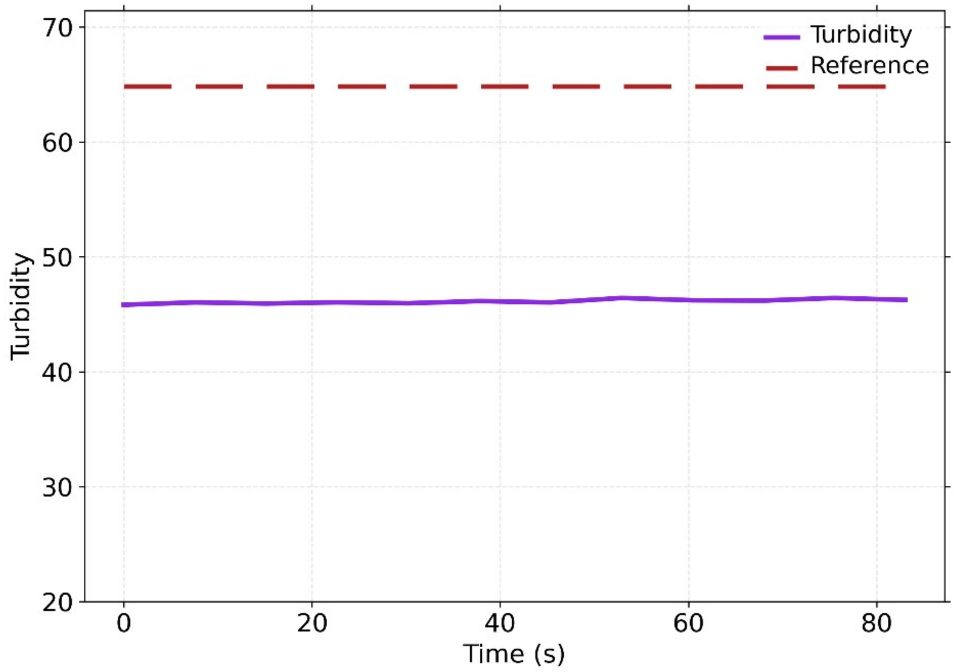

Figure S59: Turbidity vs time (s) for **D2$_{[2+3]}$**

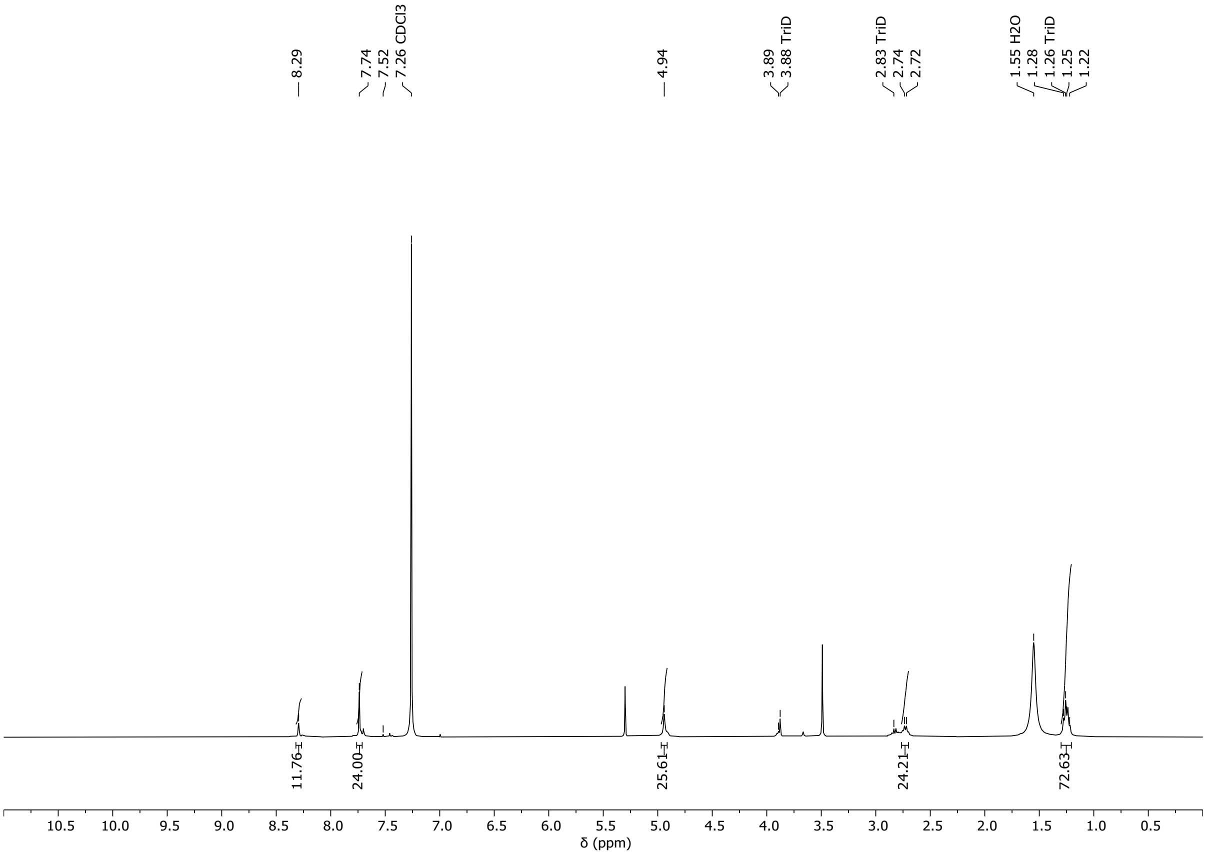

Figure S60: ^{1}H NMR ($CDCl_3$) spectrum for $\mathbf{D4_{[4+6]}}$

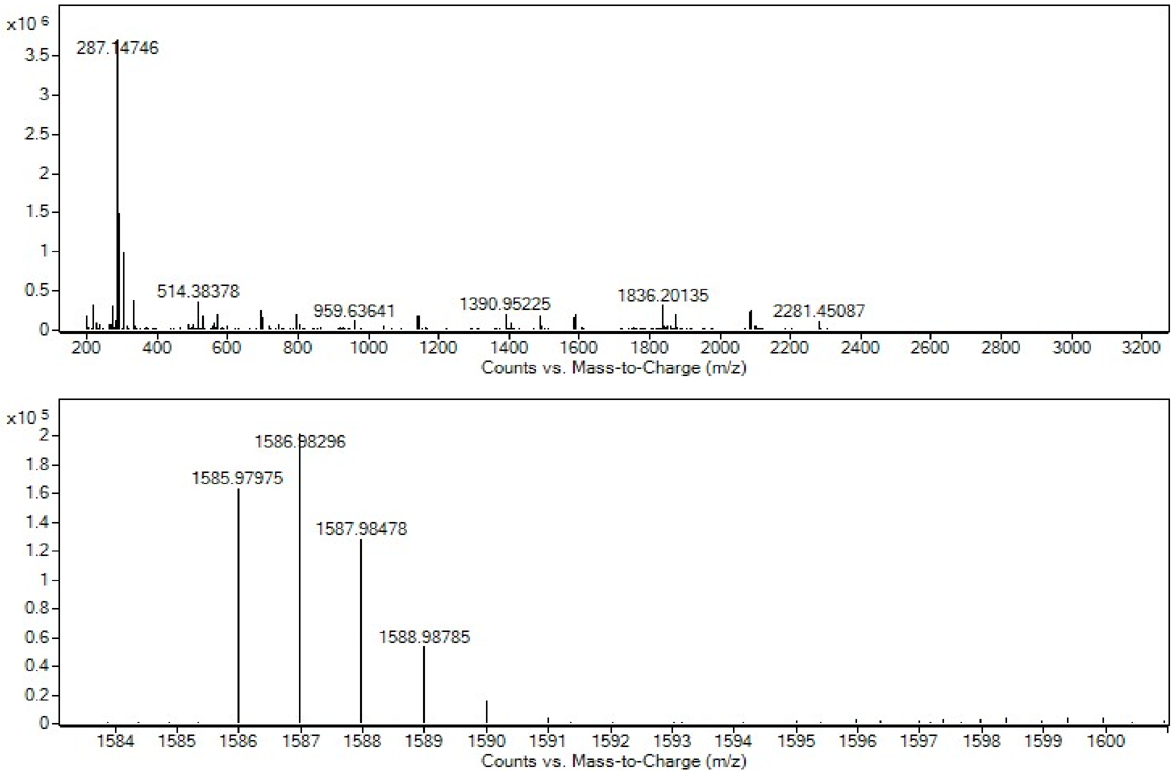

Figure S61: HRMS spectrum for $\mathbf{D4_{[4+6]}}$

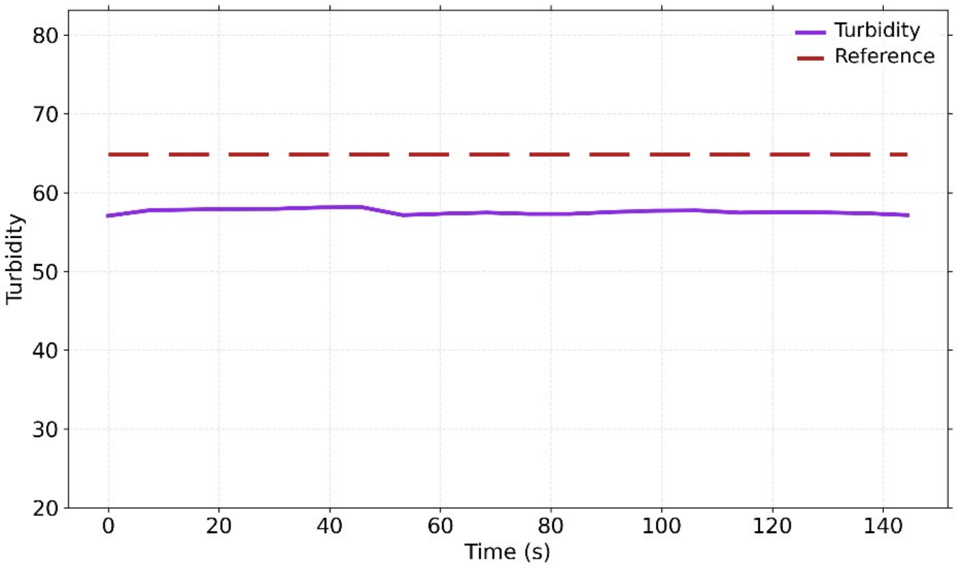

Figure S62: Turbidity vs time (s) for **D4**$_{\mathbf{[4+6]}}$

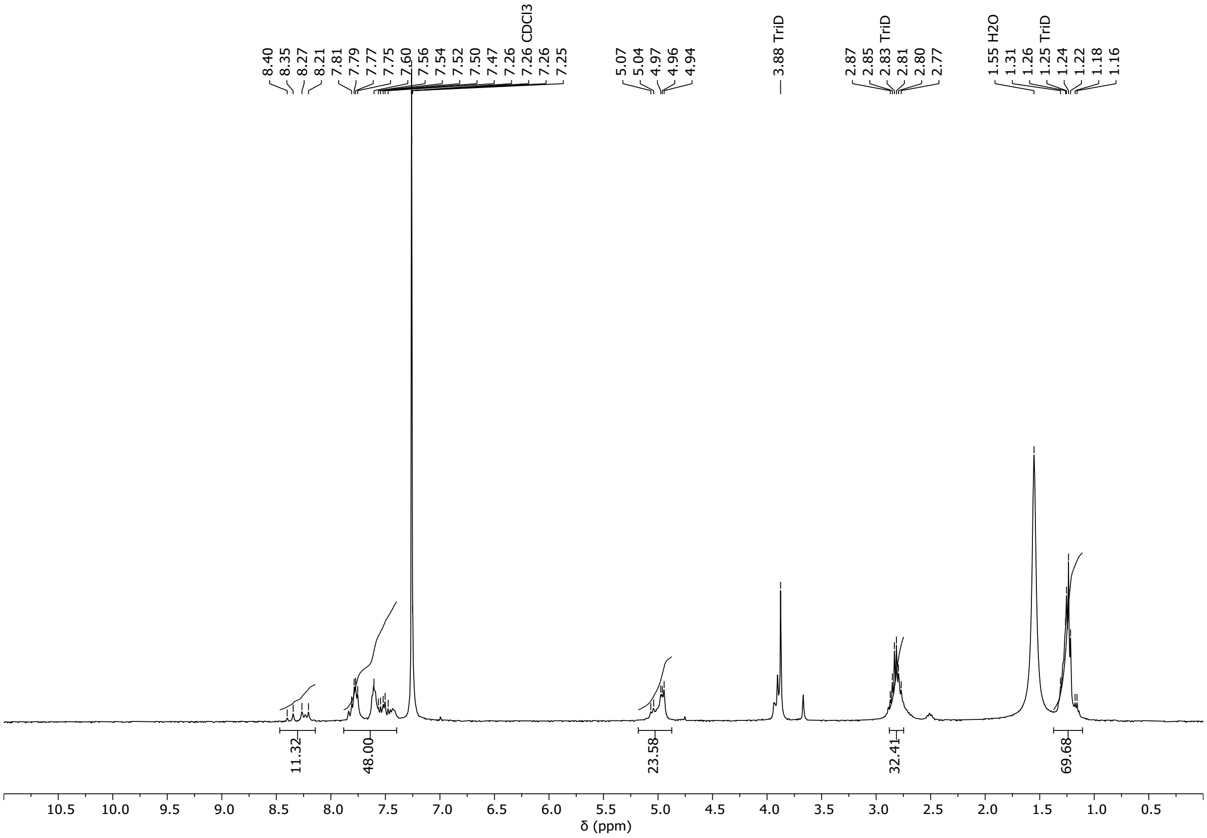

Figure S63: ^{1}H NMR ($CDCl_3$) spectrum for **D6$_{[4+6]}$**

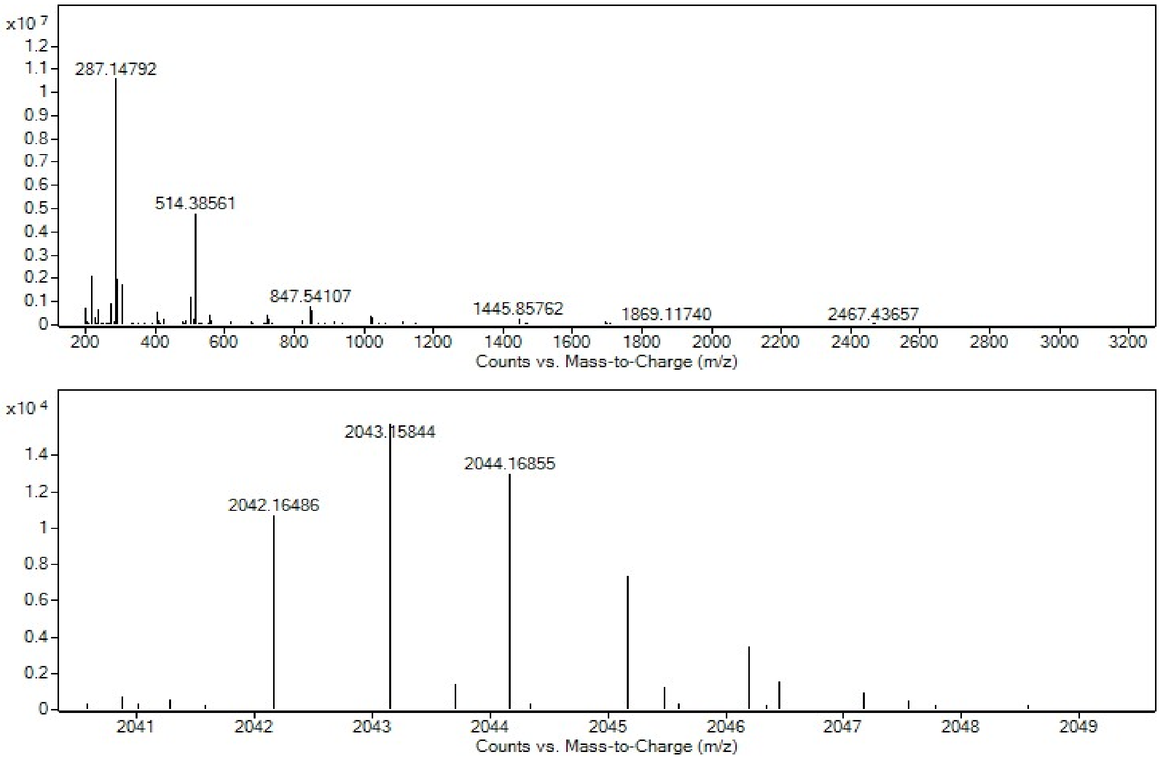

Figure S64: HRMS spectrum for **D6$_{[4+6]}$**

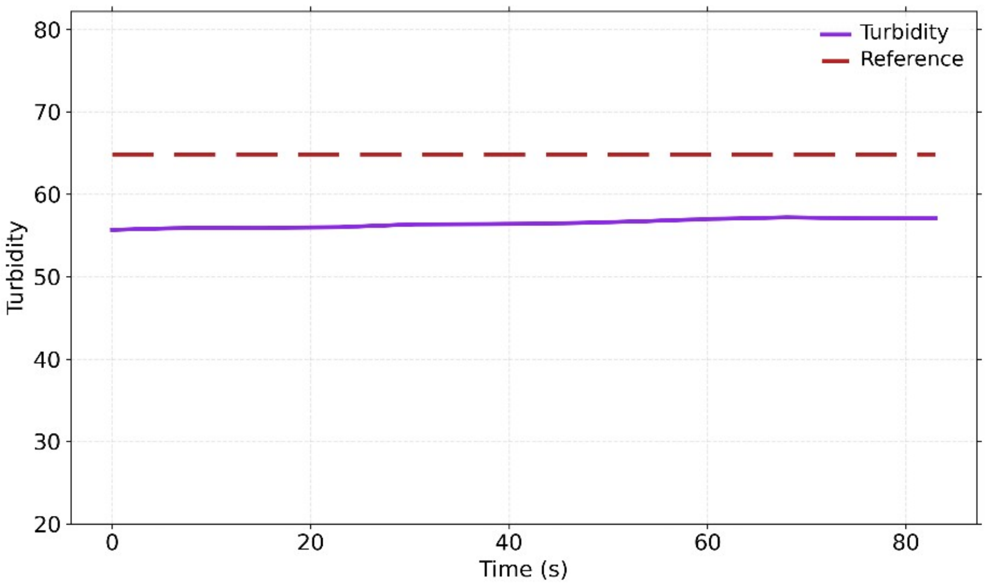

Figure S65: Turbidity vs time (s) for **$D6_{[4+6]}$**

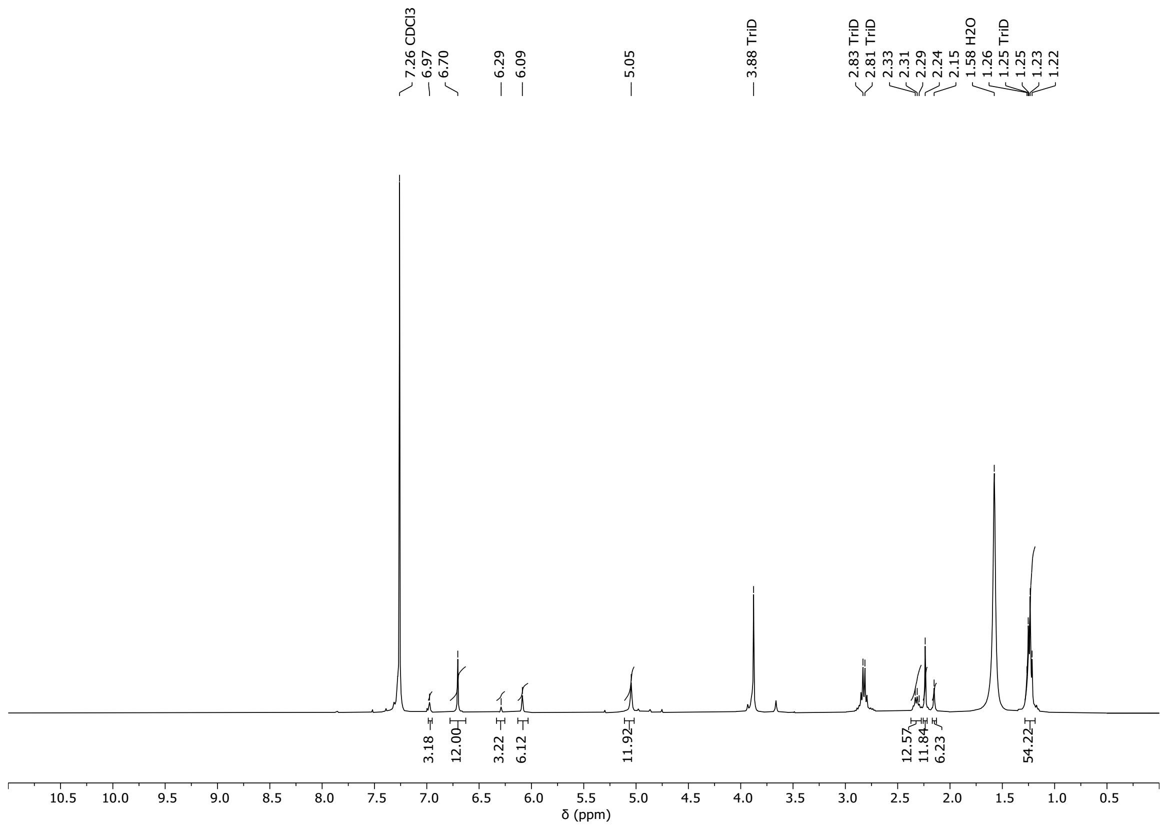

Figure S66: ^{1}H NMR ($CDCl_3$) spectrum for **D12$_{[2+3]}$** - risk of competitive 1,4- vs 1,2-addition to the unsaturated aldehyde in **12**.

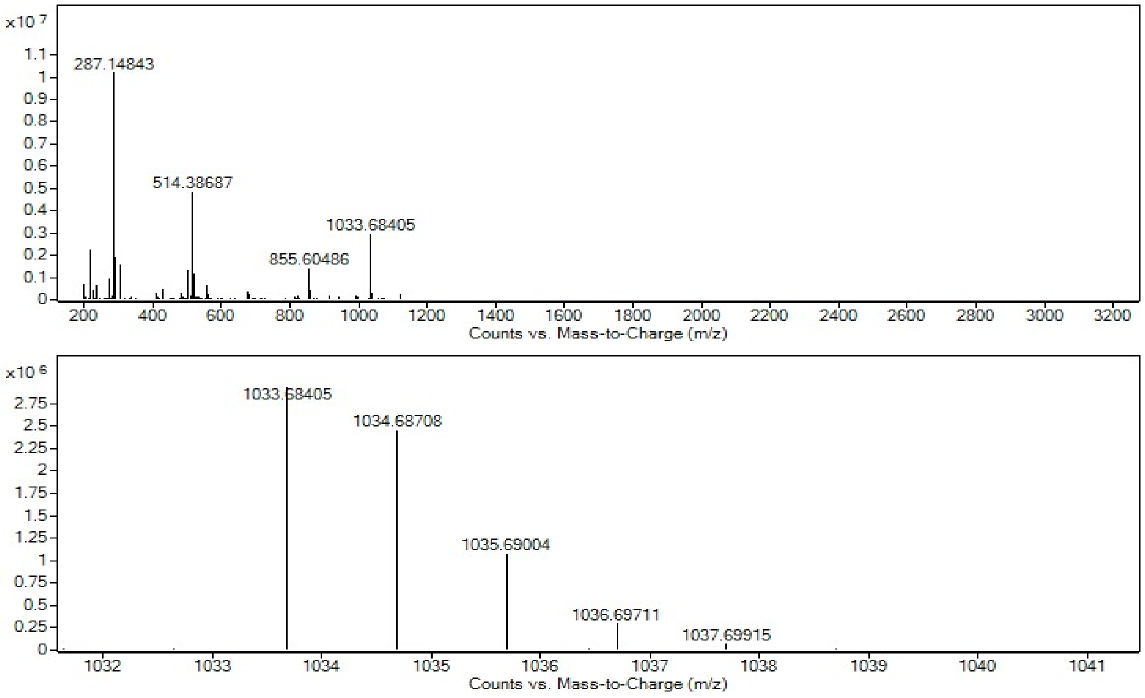

Figure S67: HRMS spectrum for **D12$_{[2+3]}$**

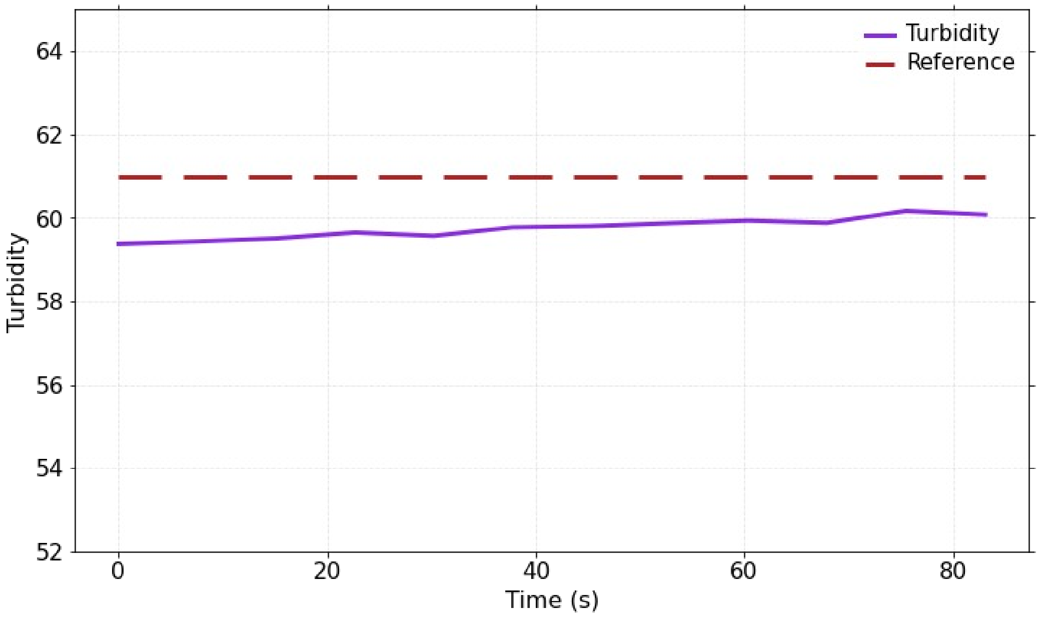

Figure S68: Turbidity vs time (s) for $\mathbf{D12}_{[2+3]}$

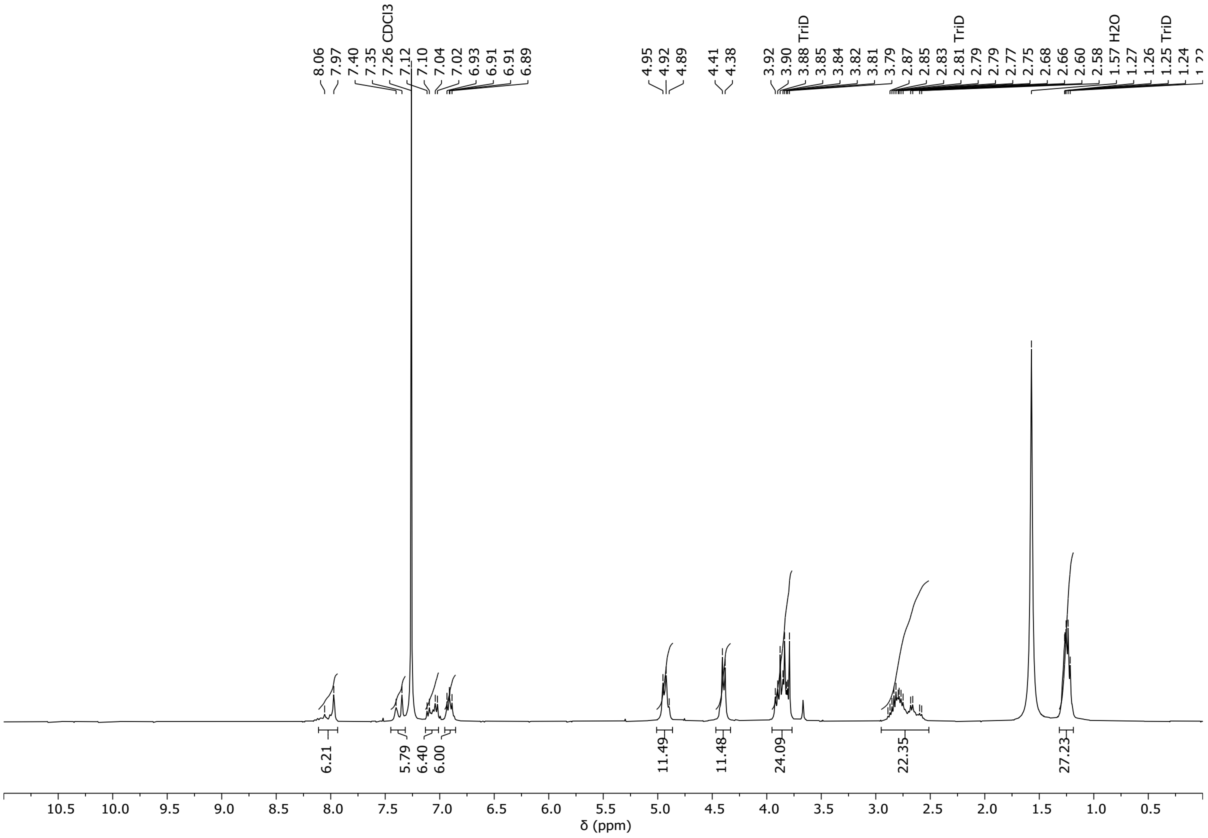

Figure S69: ^{1}H NMR ($CDCl_3$) spectrum for **$D13_{[2+3]}$**

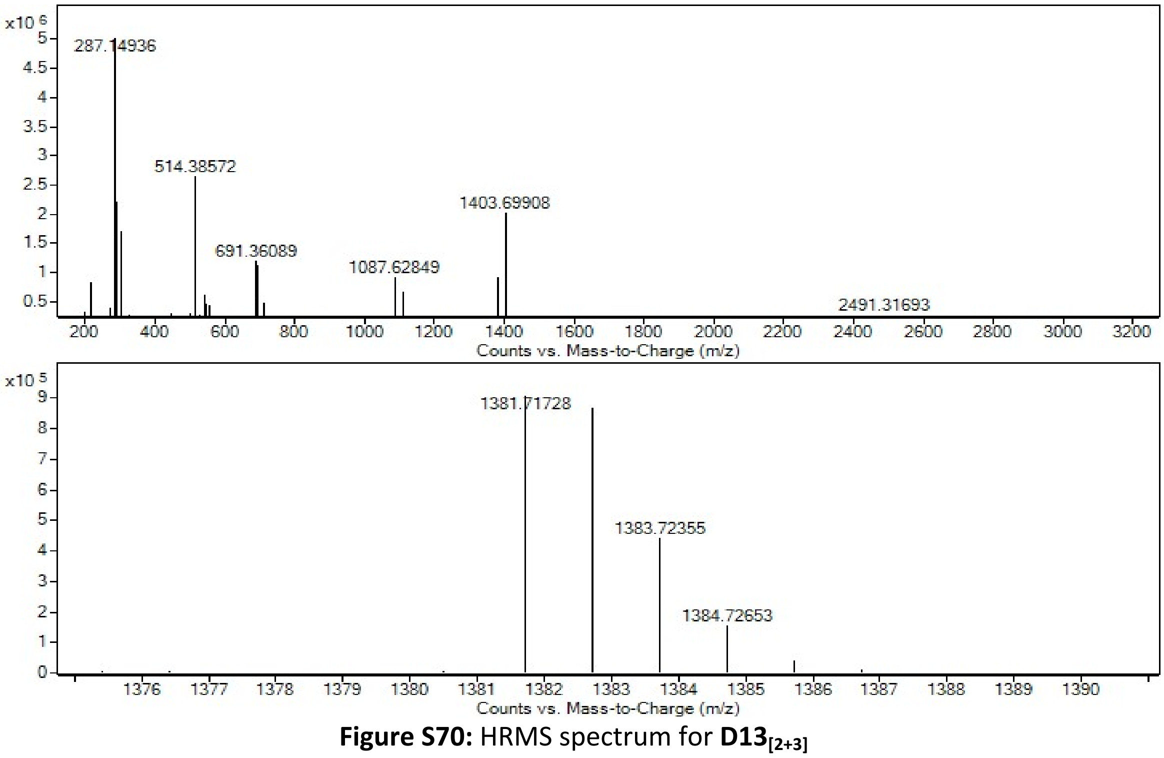

Figure S70: HRMS spectrum for **$D13_{[2+3]}$**

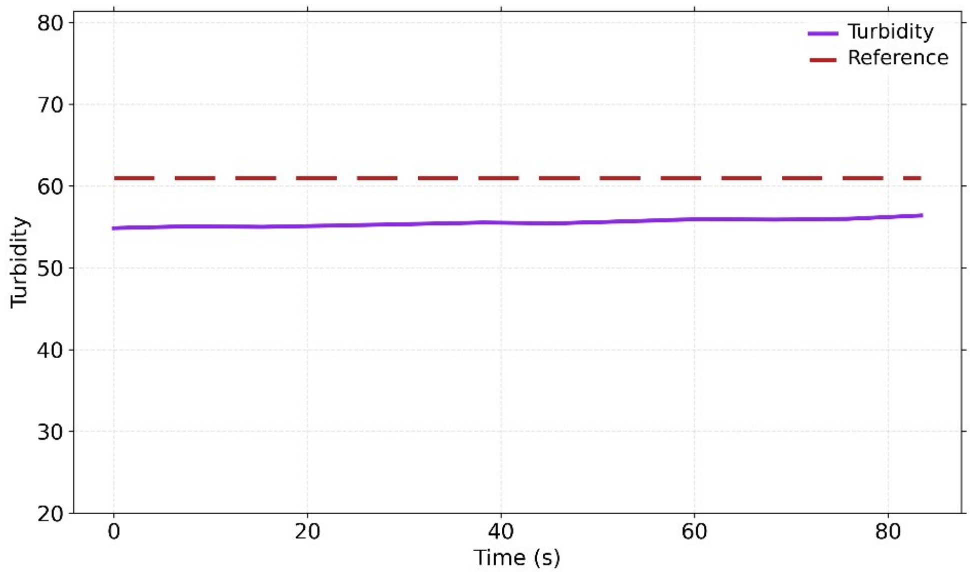

Figure S71: Turbidity vs time (s) for **D13$_{[2+3]}$**

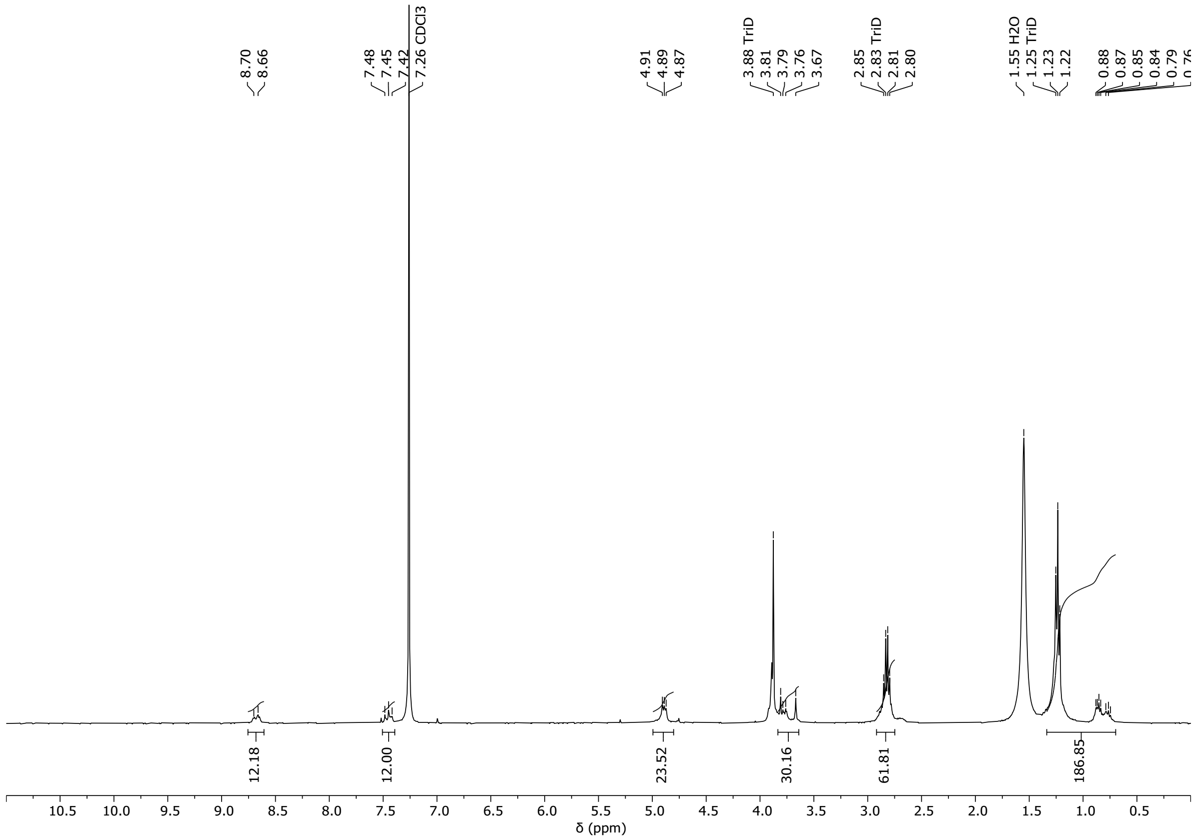

Figure S72: ^{1}H NMR ($CDCl_3$) spectrum for **D16$_{[4+6]}$**

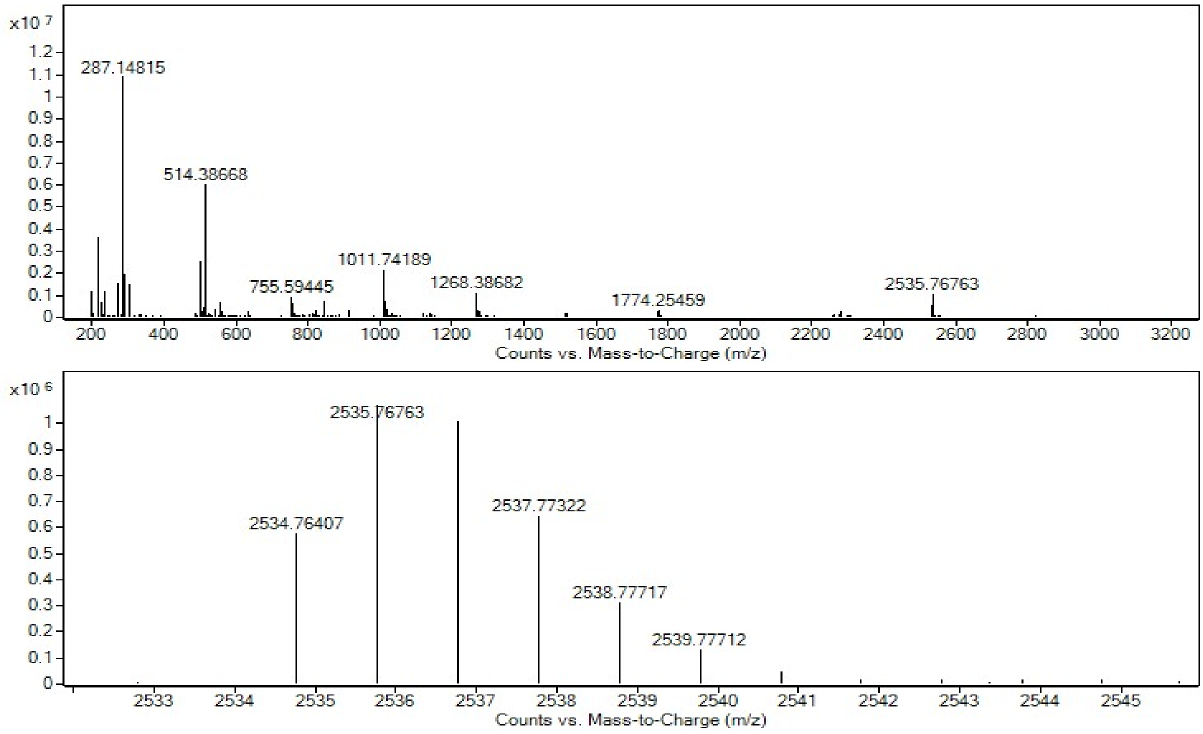

Figure S73: HRMS spectrum for **D16$_{[4+6]}$**

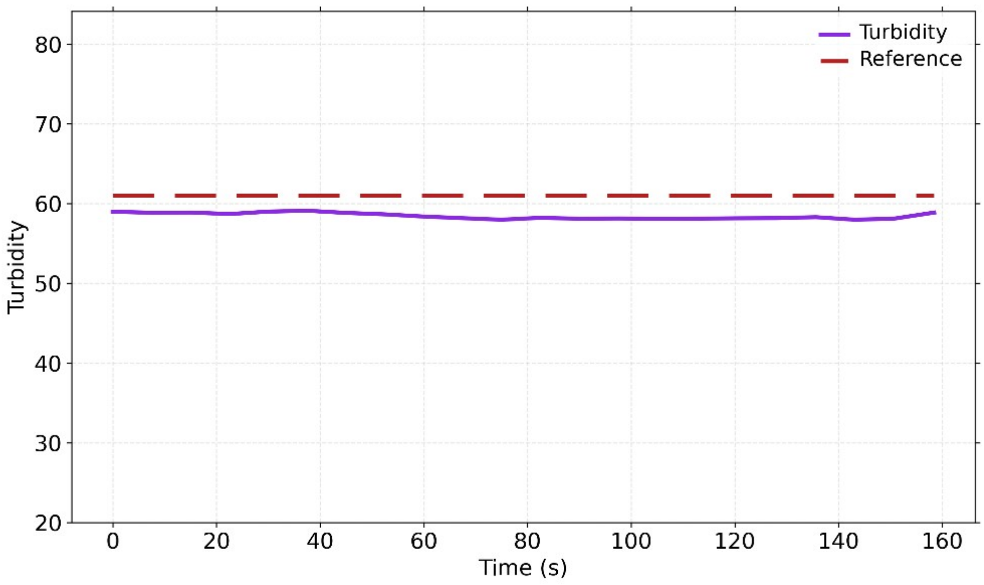

Figure S74: Turbidity vs time (s) for $\mathbf{D16}_{[4+6]}$

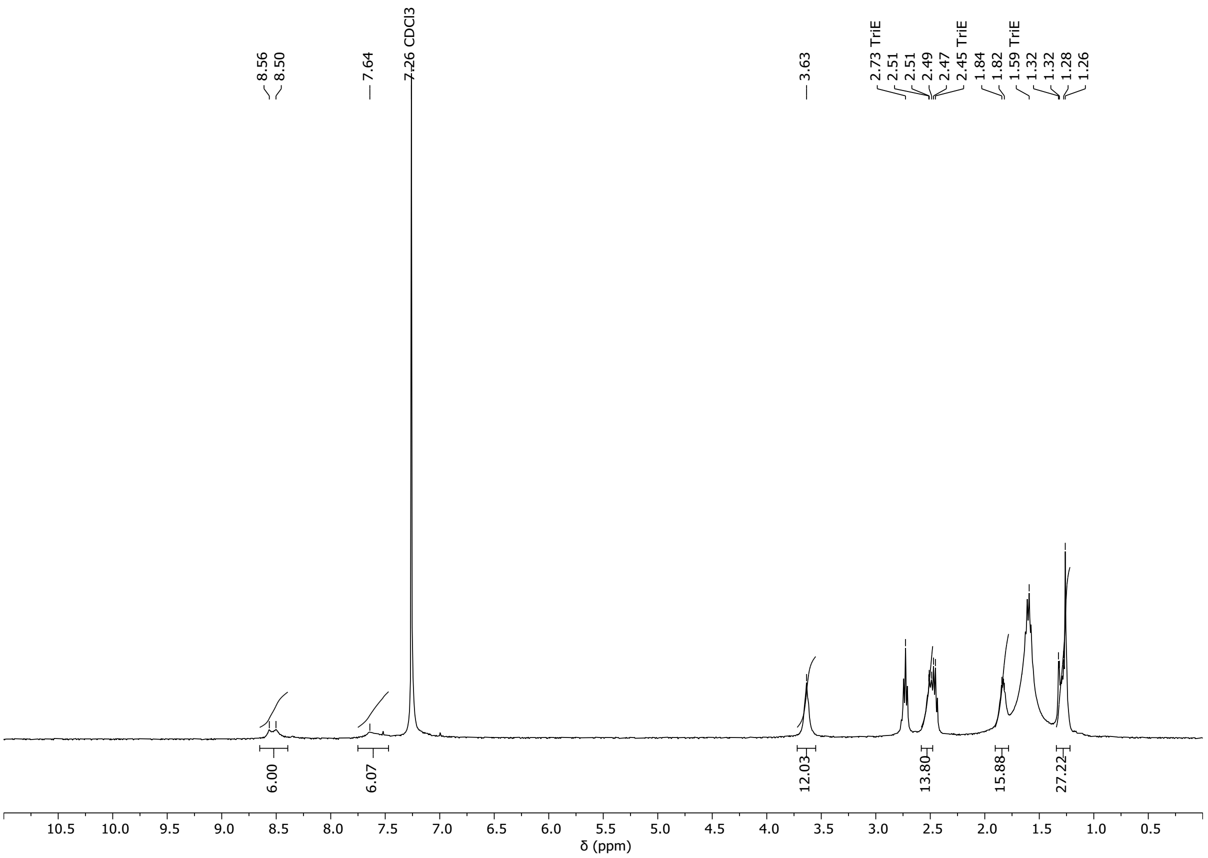

Figure S75: ^{1}H NMR ($CDCl_3$) spectrum for $\mathbf{E2_{[2+3]}}$

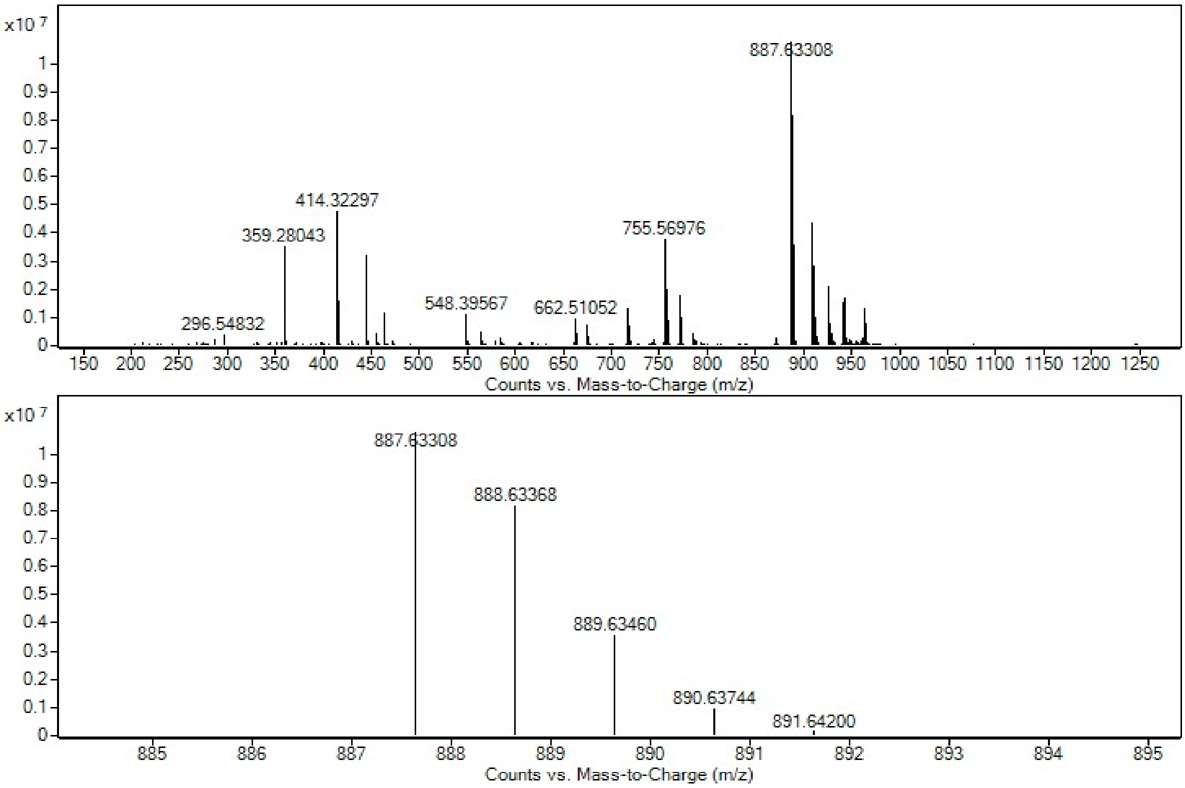

Figure S76: HRMS spectrum for $\mathbf{E2_{[2+3]}}$

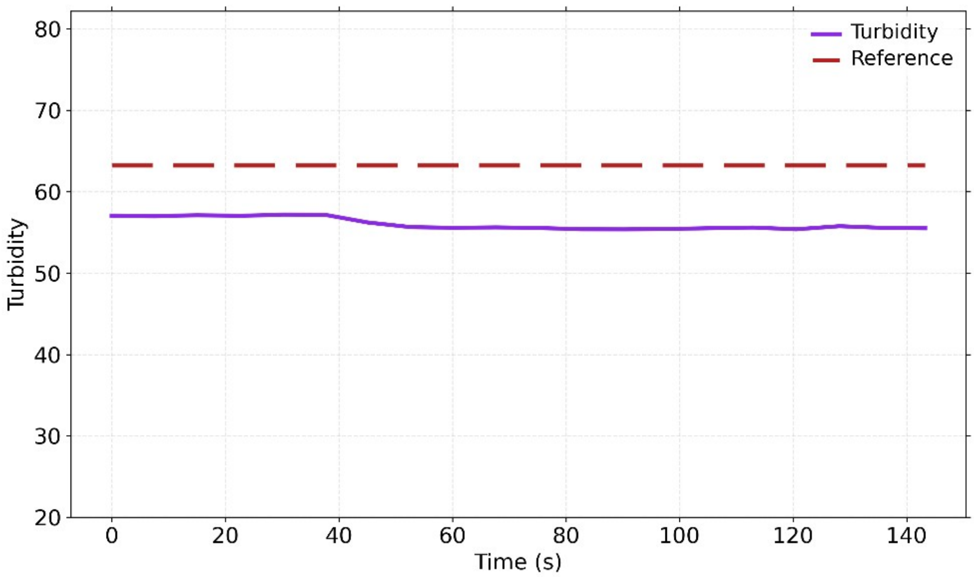

Figure S77: Turbidity vs time (s) for $\mathbf{E2}_{[2+3]}$

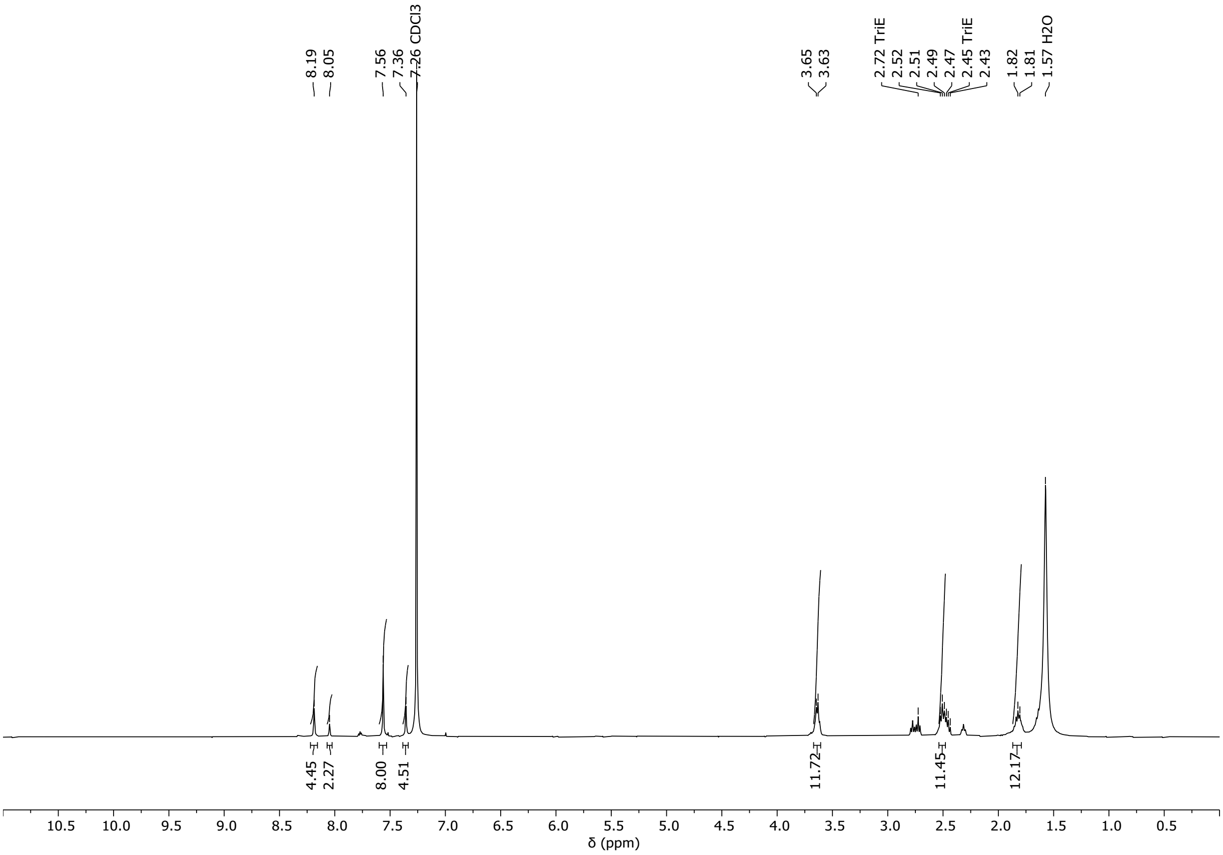

Figure S78: ^{1}H NMR ($CDCl_3$) spectrum for **E4$_{[2+3]}$**

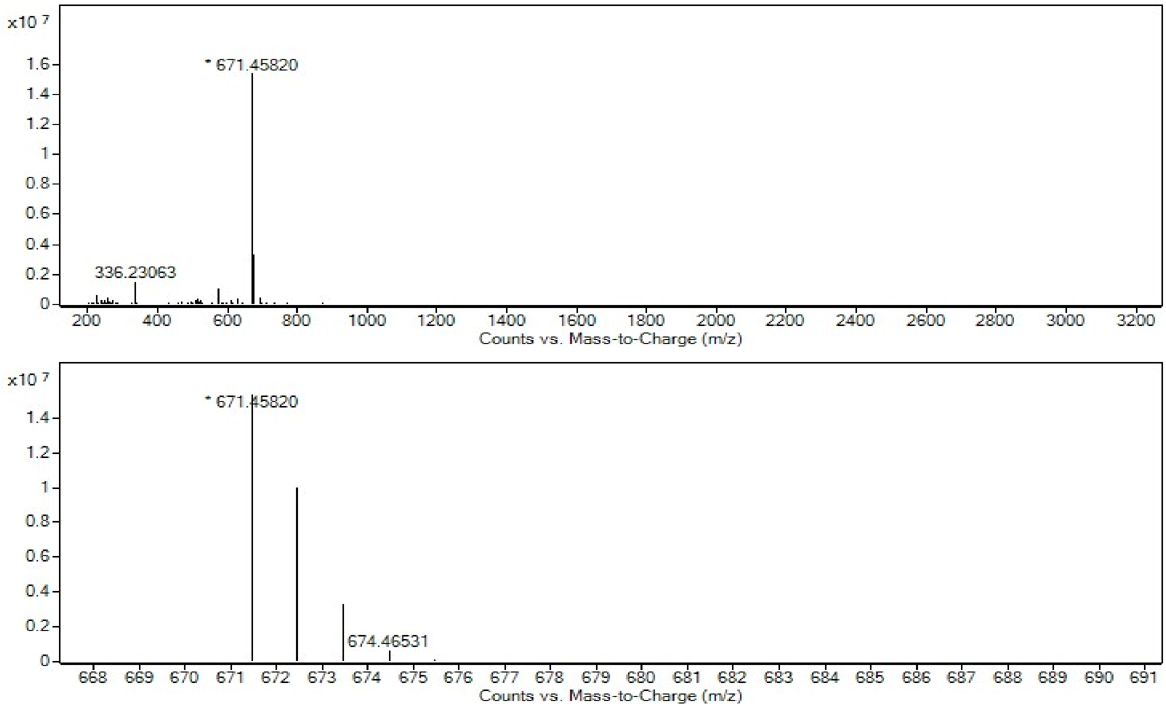

Figure S79: HRMS spectrum for **E4$_{[2+3]}$**

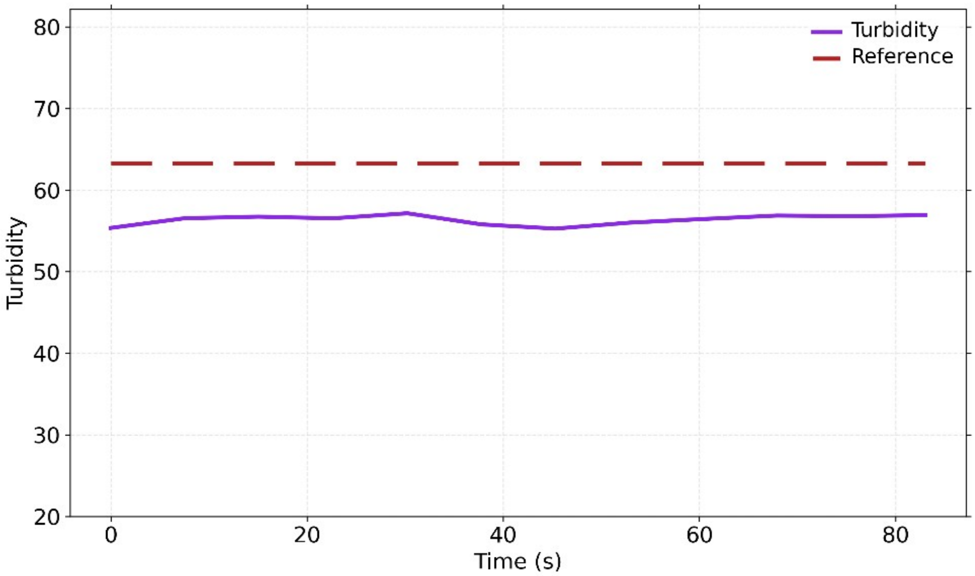

Figure S80: Turbidity vs time (s) for $\mathbf{E4}_{[2+3]}$

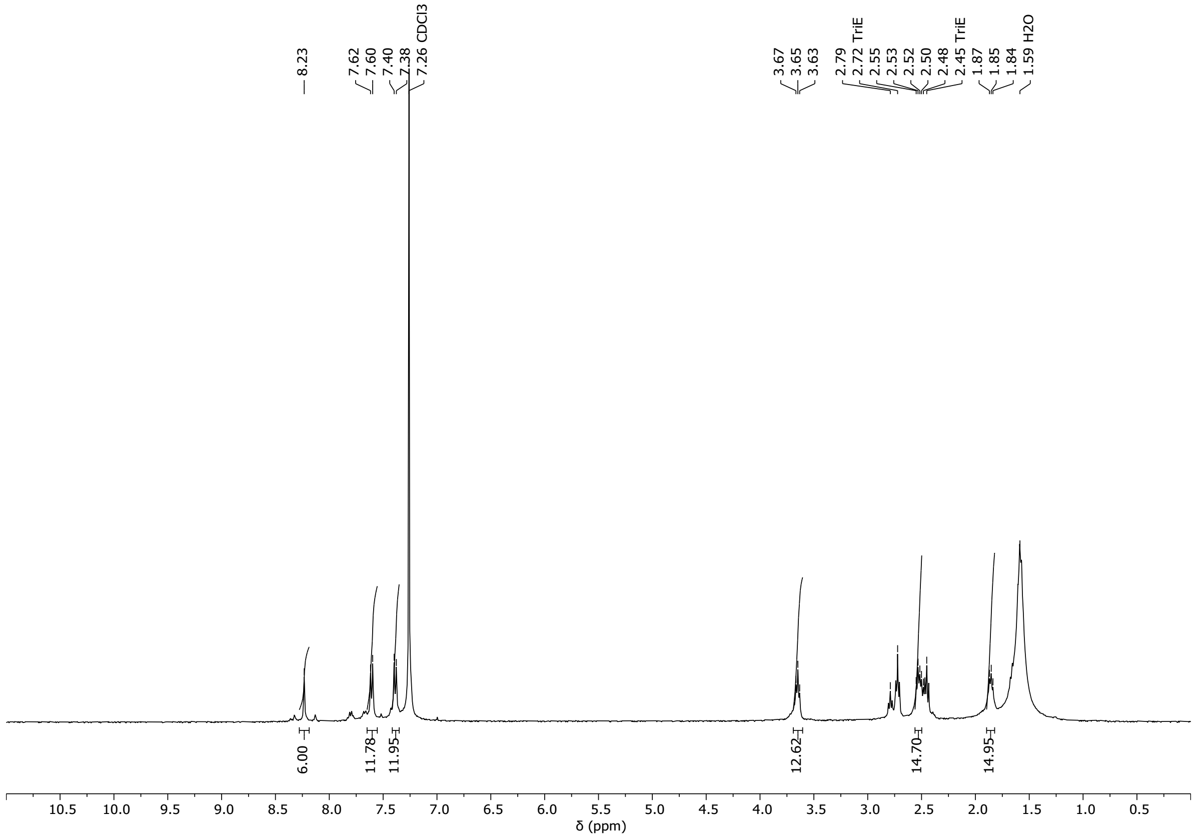

Figure S81: ^{1}H NMR ($CDCl_3$) spectrum for **E6$_{[2+3]}$**

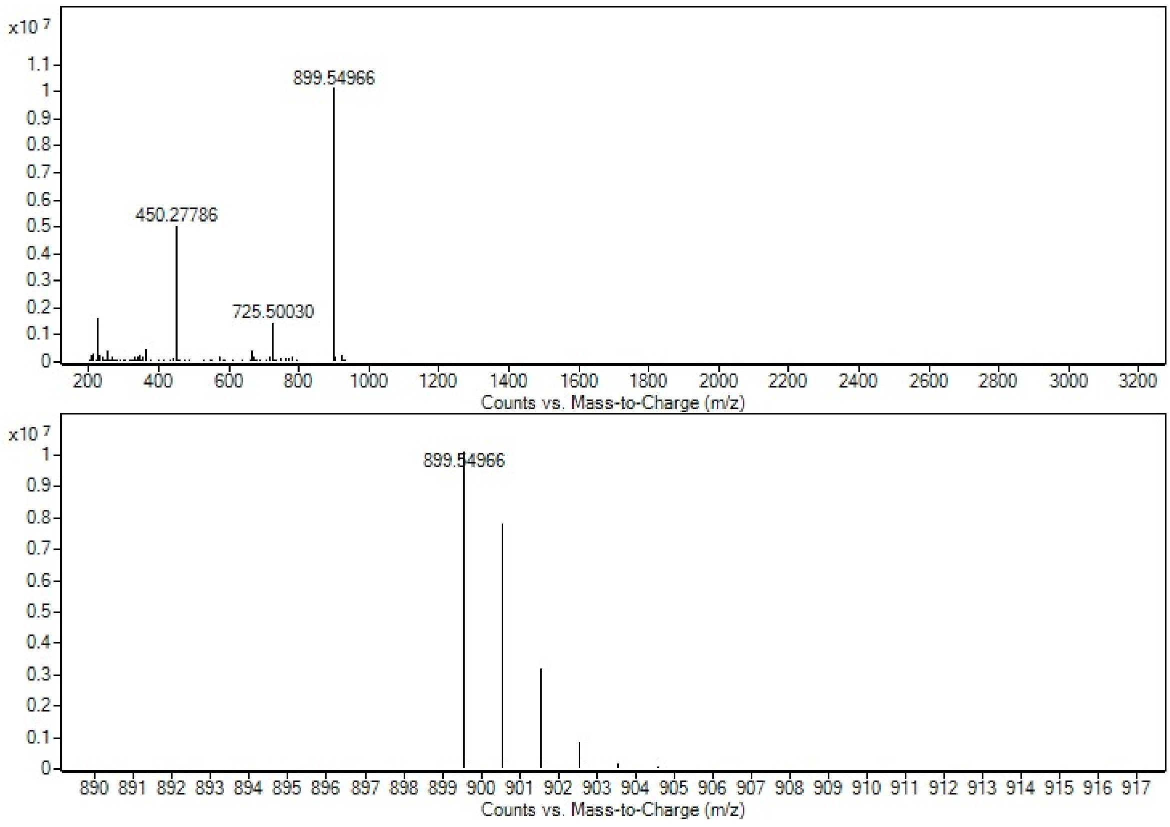

Figure S82: HRMS spectrum for **E6$_{[2+3]}$**

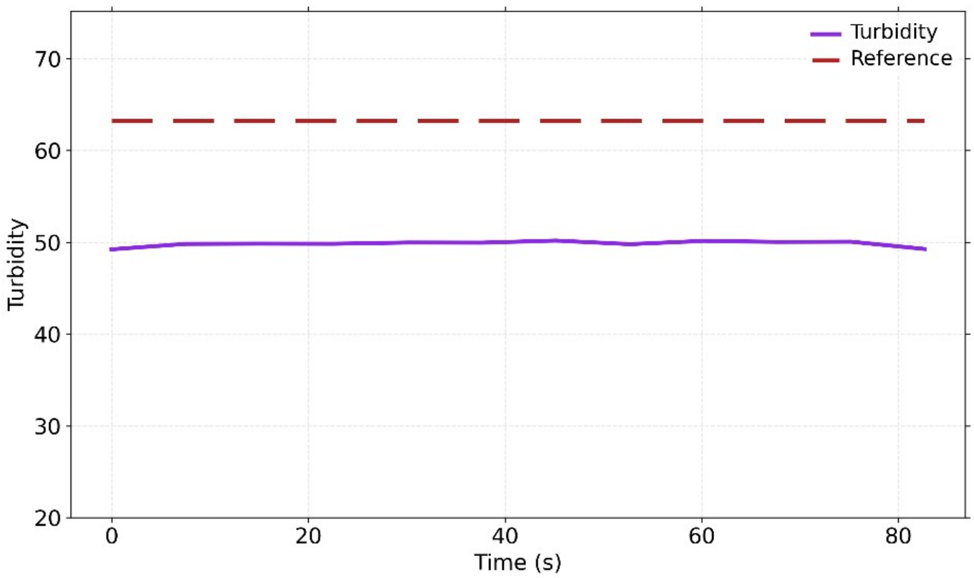

Figure S83: Turbidity vs time (s) for **E6$_{[2+3]}$**

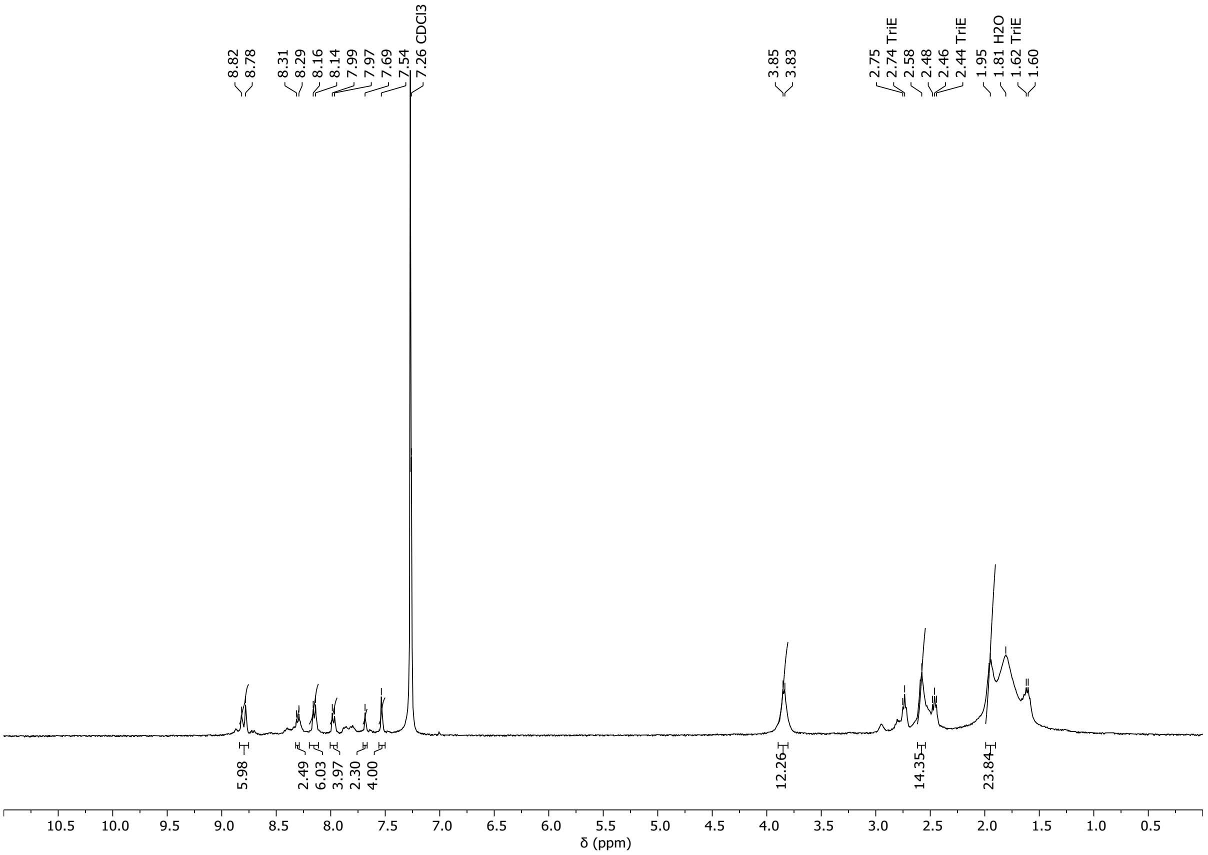

Figure S84: ^{1}H NMR ($CDCl_3$) spectrum for $\mathbf{E8_{[2+3]}}$

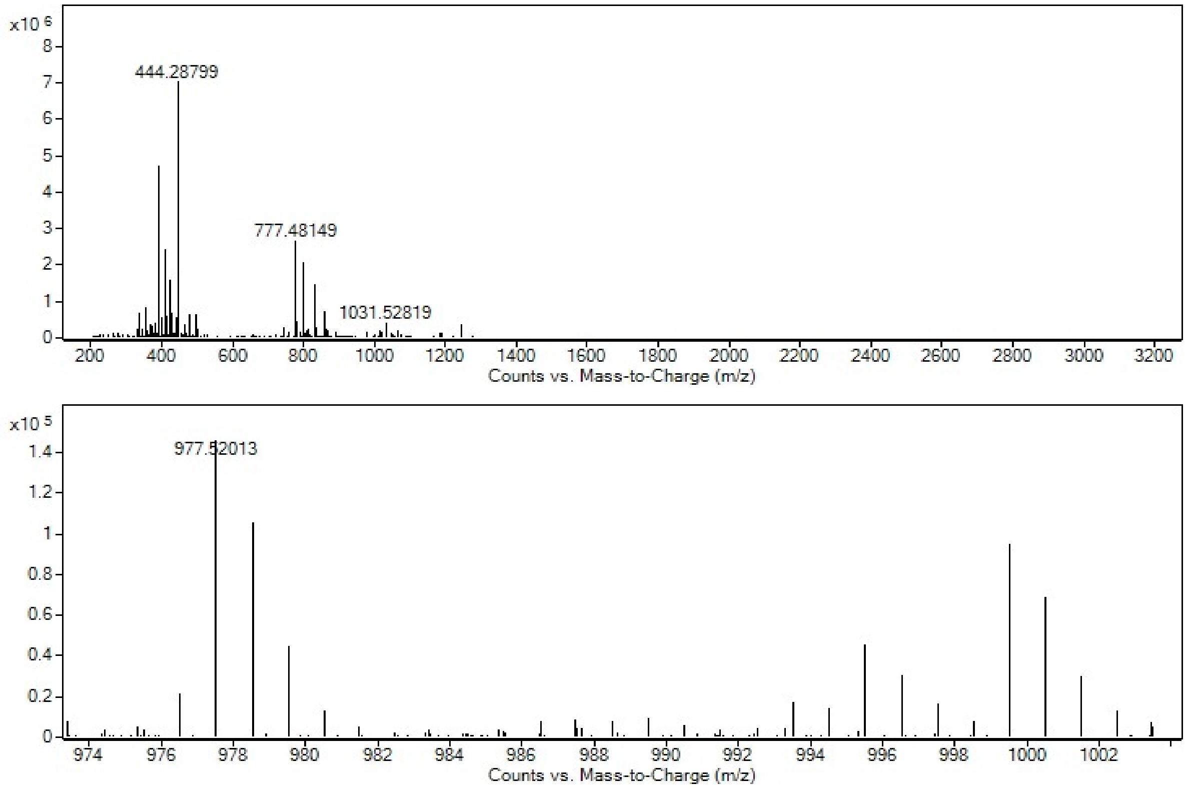

Figure S85: HRMS spectrum for $\mathbf{E8_{[2+3]}}$

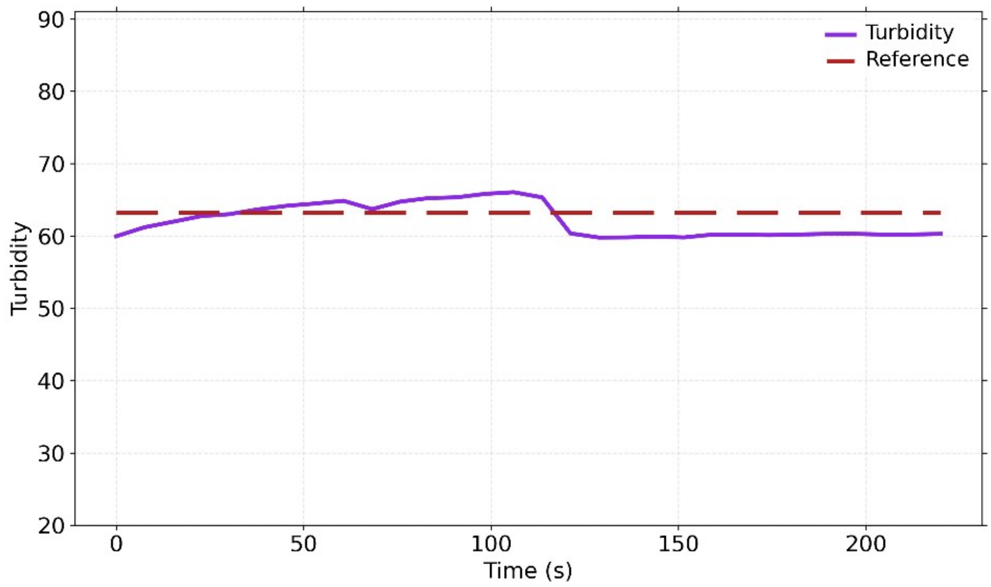

Figure S86: Turbidity vs time (s) for **E8$_{[2+3]}$**. Sample turbidity remained below reference between 120-240 seconds and passed turbidity check.

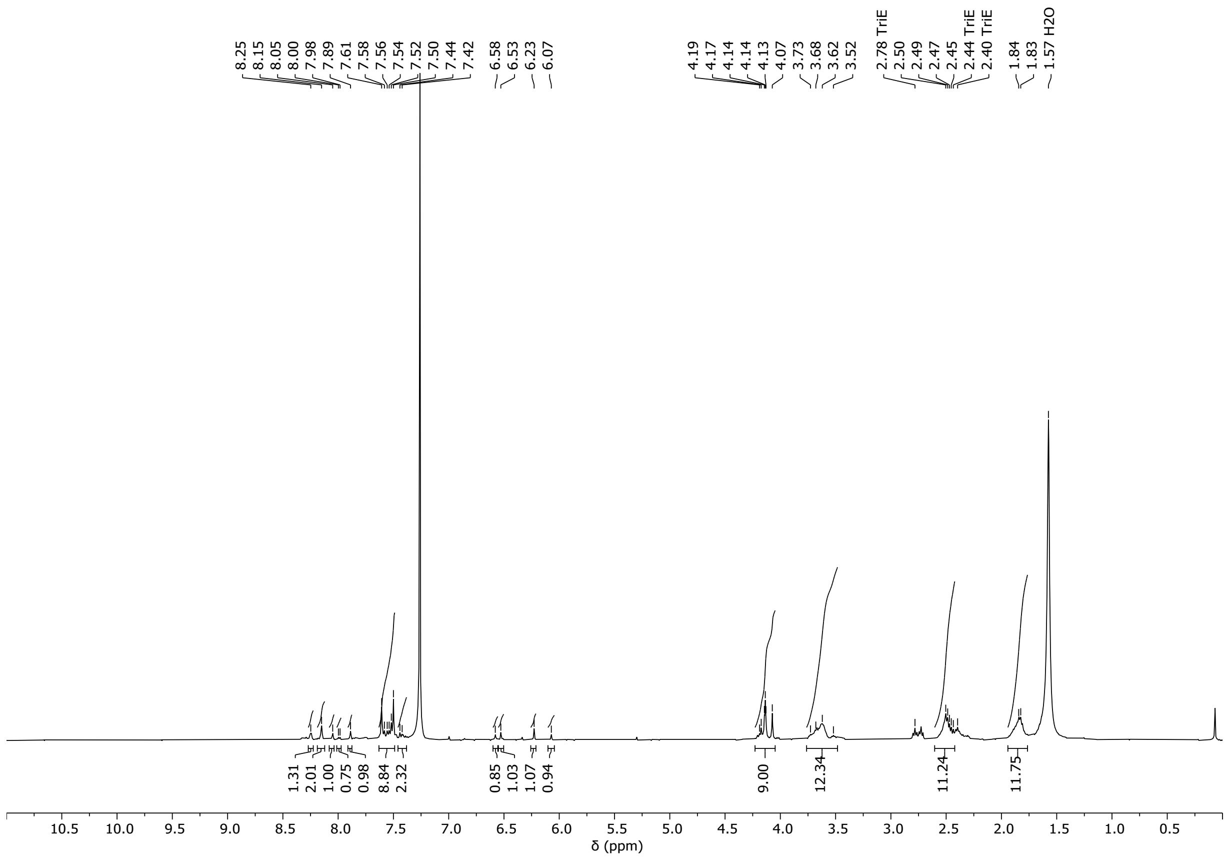

Figure S87: ^{1}H NMR ($CDCl_3$) spectrum for **E11$_{[2+3]}$**

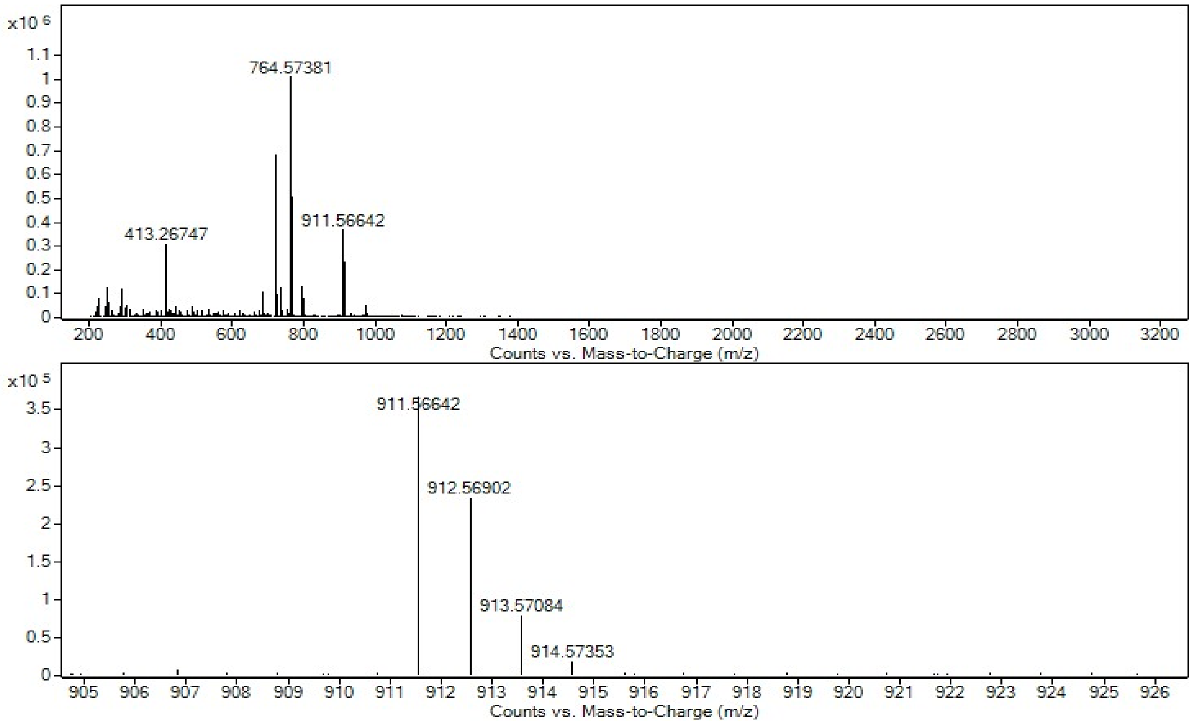

Figure S88: HRMS spectrum for **E11$_{[2+3]}$**

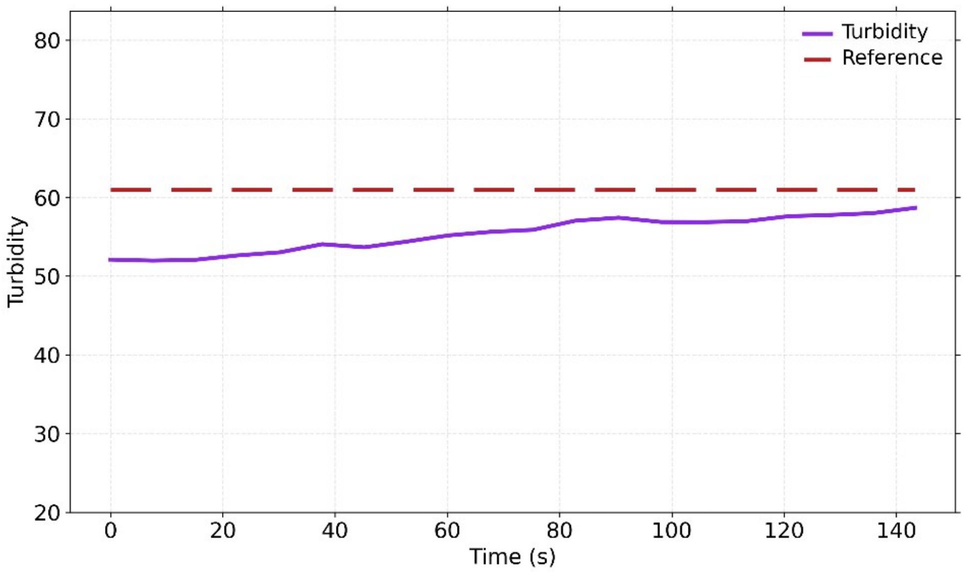

Figure S89: Turbidity vs time (s) for **E11$_{[2+3]}$**

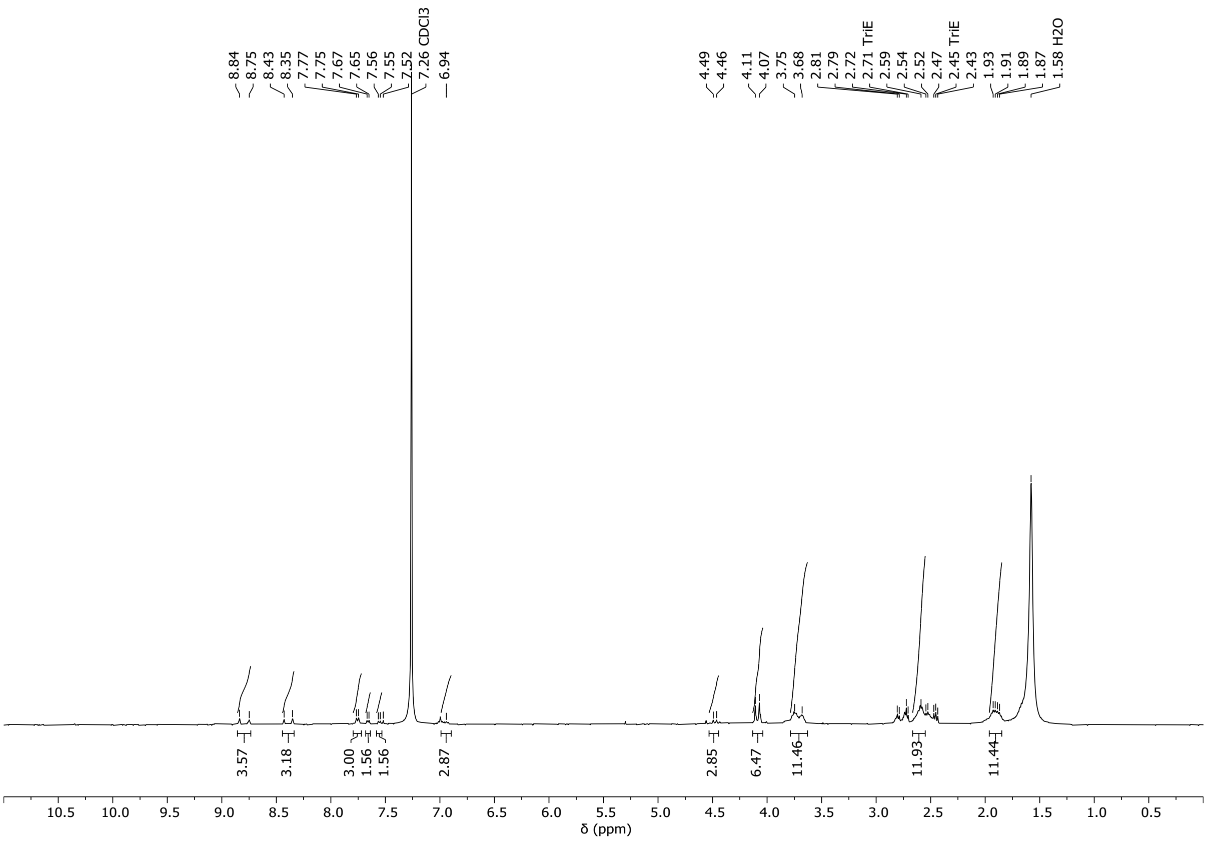

Figure S90: ^{1}H NMR ($CDCl_3$) spectrum for **$E14_{[2+3]}$**

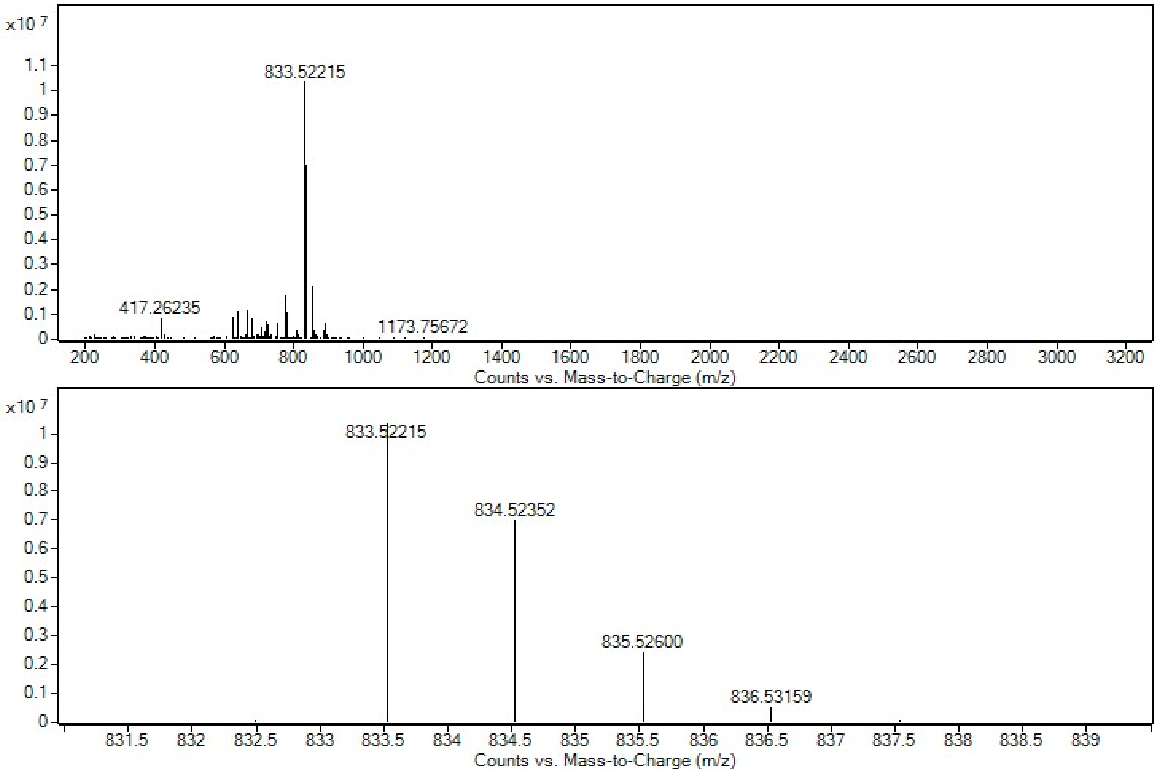

Figure S91: HRMS spectrum for **$E14_{[2+3]}$**

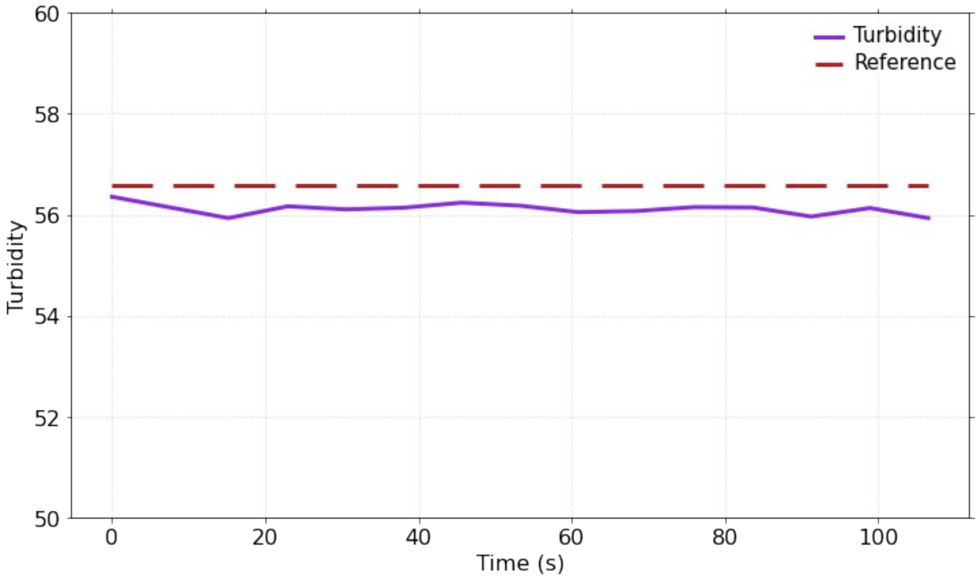

Figure S92: Turbidity vs time (s) for **E14**$_{\mathbf{[2+3]}}$

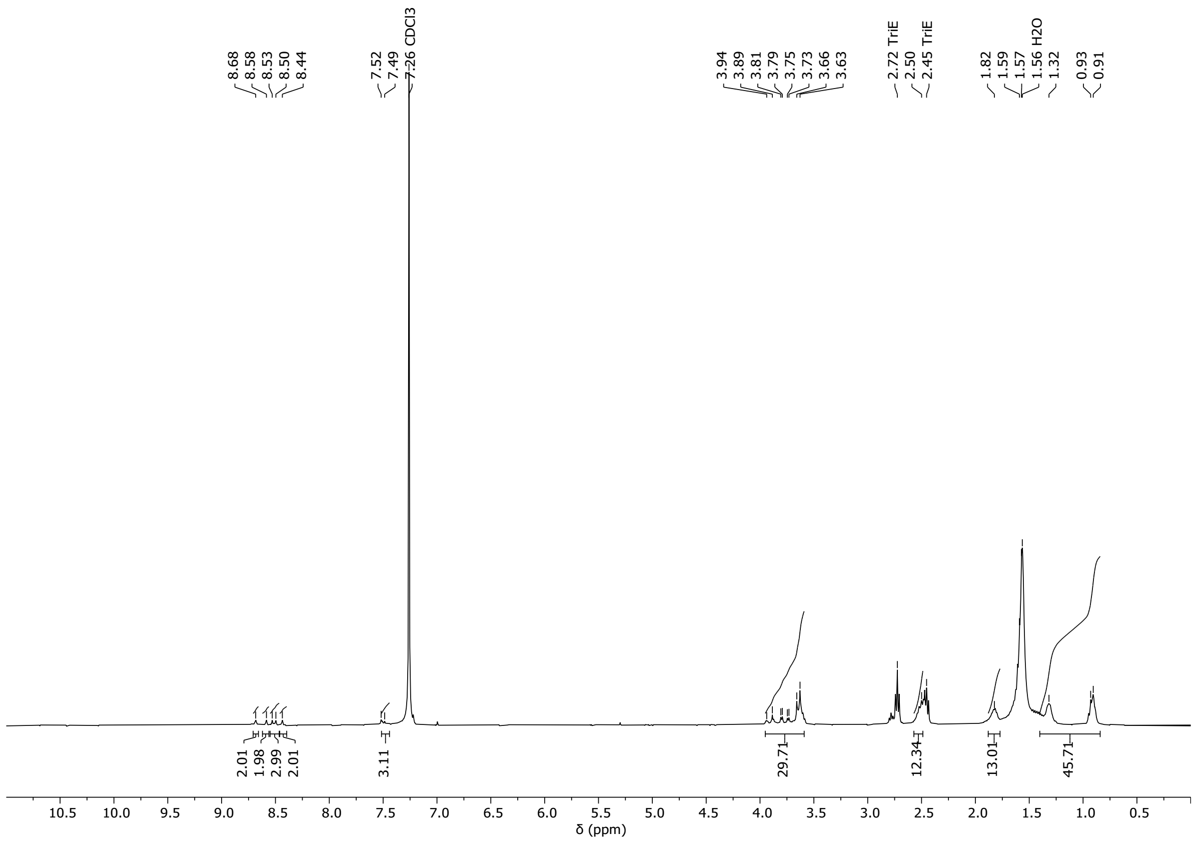

Figure S93: ^{1}H NMR ($CDCl_3$) spectrum for **E16$_{[2+3]}$**

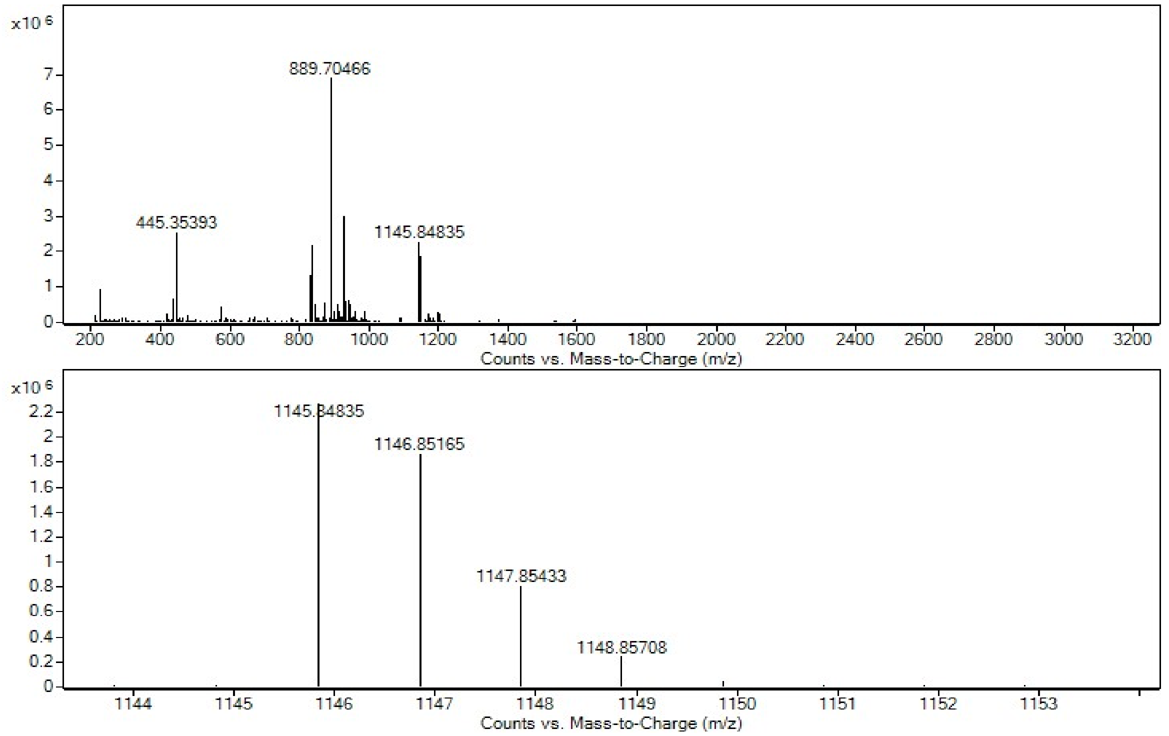

Figure S94: HRMS spectrum for **E16$_{[2+3]}$**

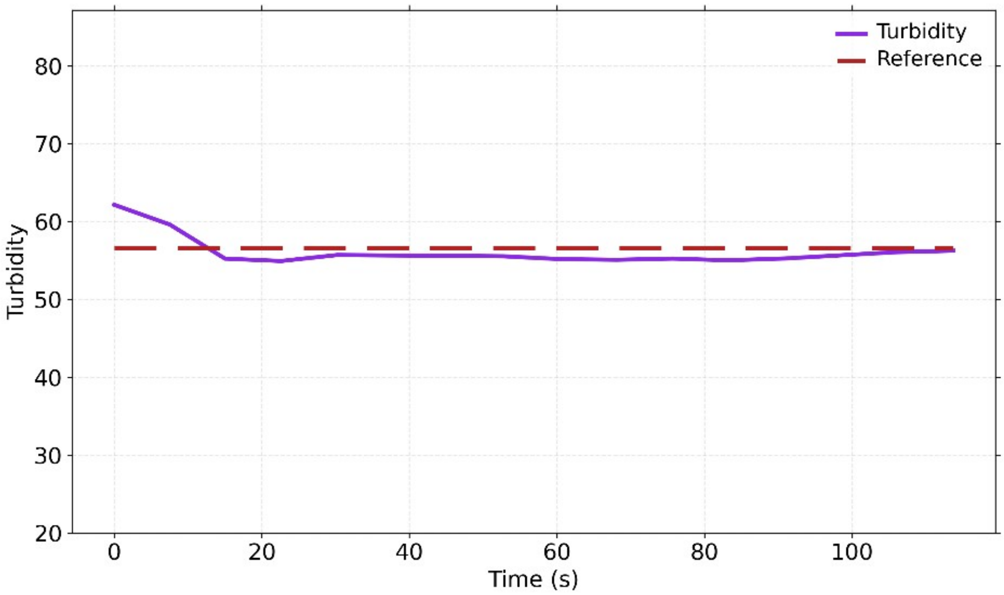

Figure S95: Turbidity vs time (s) for **E16$_{[2+3]}$**. Sample turbidity remained below reference between 20-140 seconds and passed turbidity check.

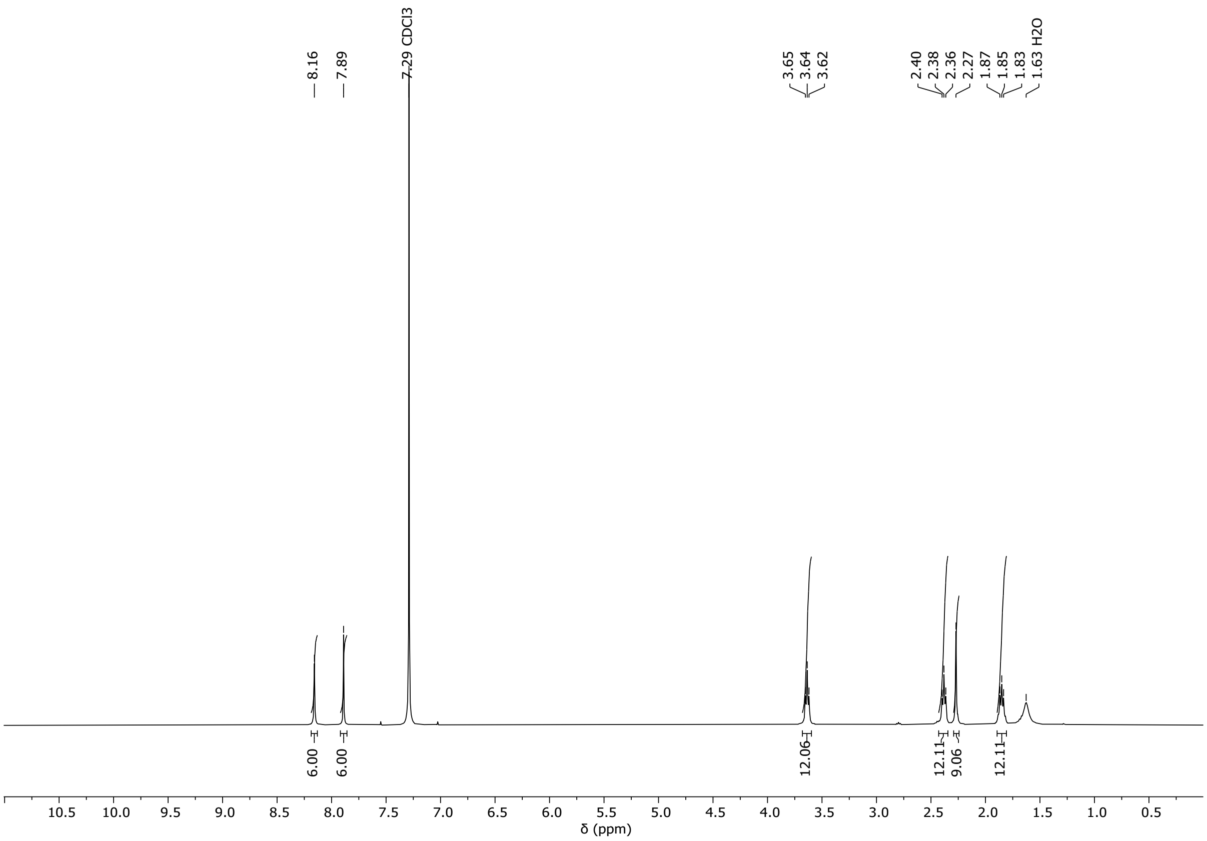

Figure S96: ^{1}H NMR ($CDCl_3$) spectrum for **$G29_{[2+3]}$**

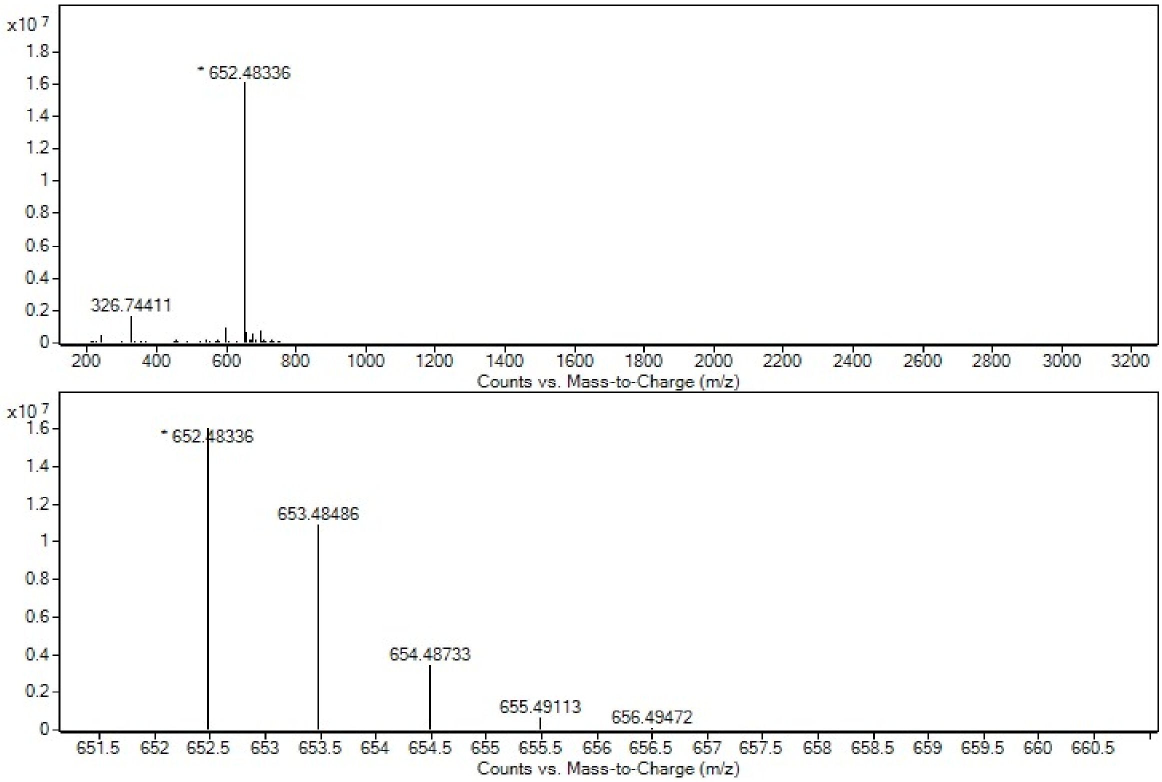

Figure S97: HRMS spectrum for **$G29_{[2+3]}$**

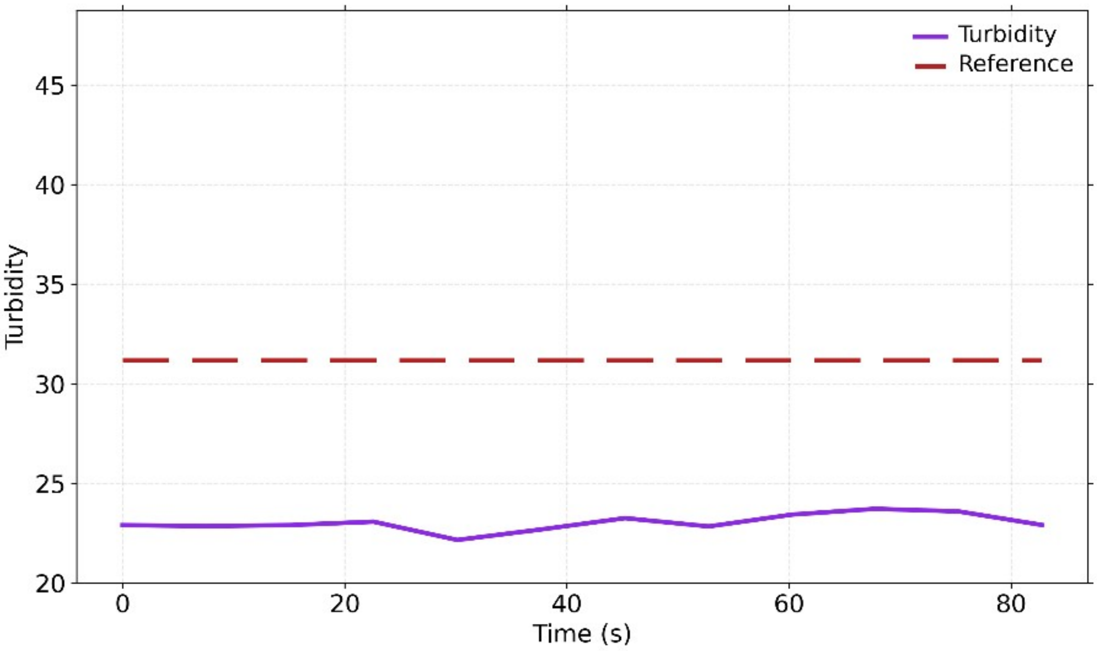

Figure S98: Turbidity vs time (s) for **G29$_{[2+3]}$**

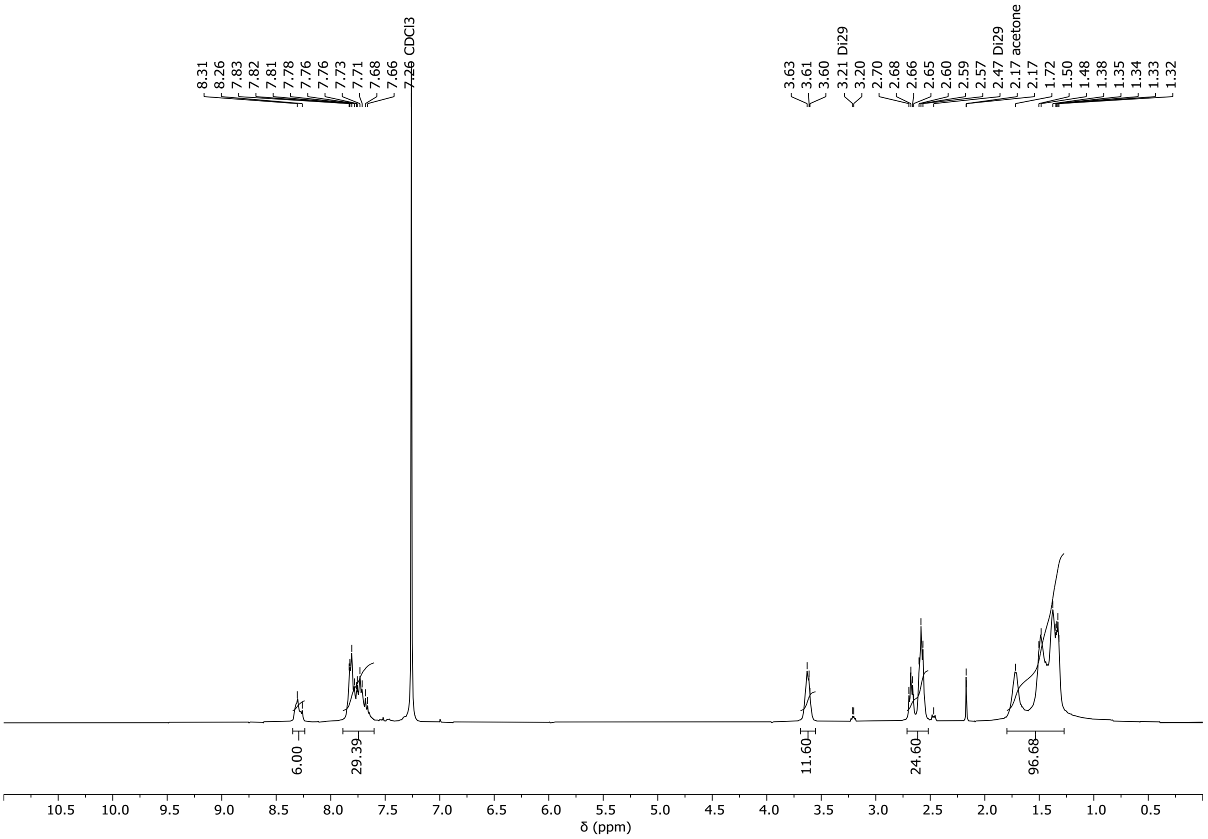

Figure S99: ^{1}H NMR ($CDCl_3$) spectrum for $\mathbf{H25_{[2+3]}}$

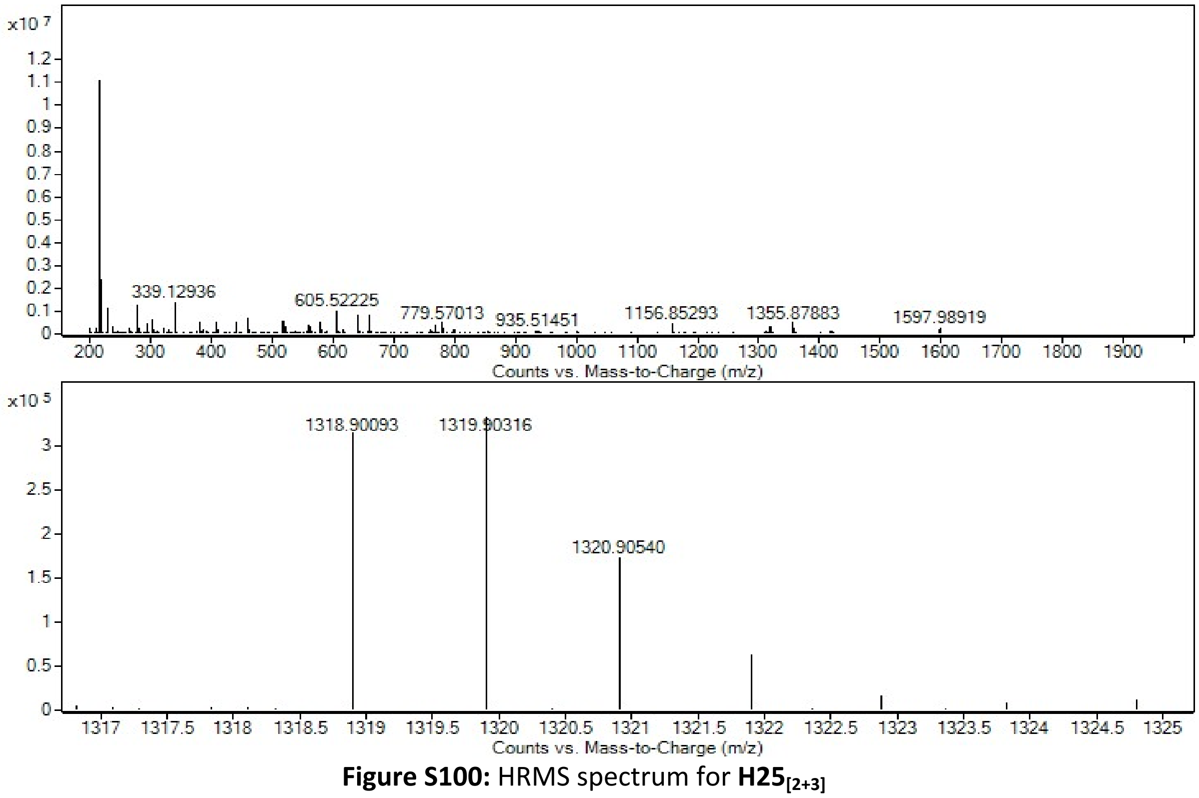

Figure S100: HRMS spectrum for $\mathbf{H25_{[2+3]}}$

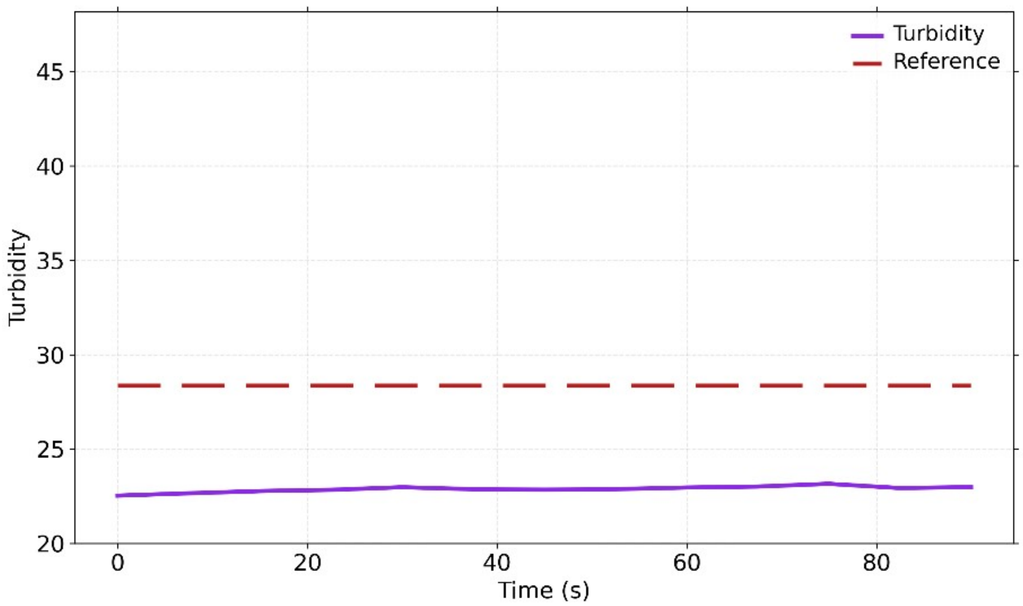

Figure S101: Turbidity vs time (s) for **H25$_{[2+3]}$**

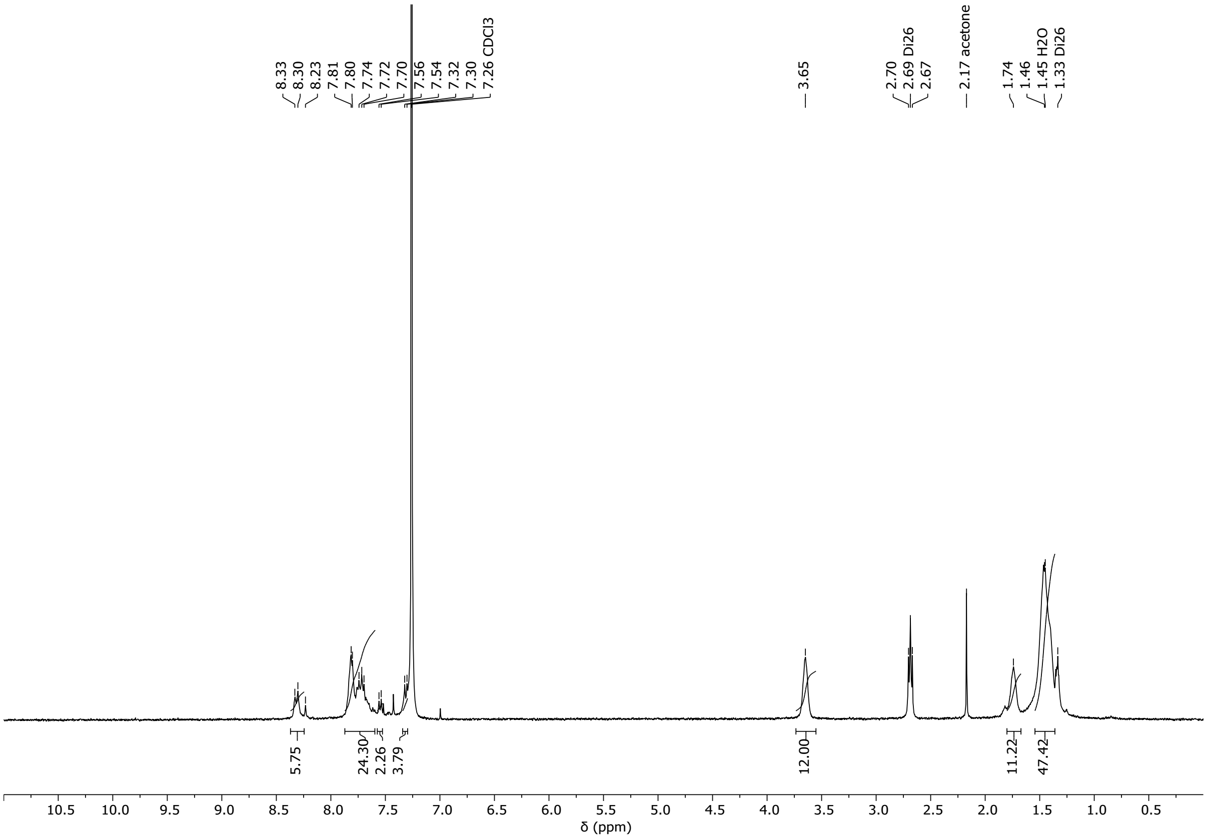

Figure S102: ^1H NMR ($CDCl_3$) spectrum for **H26[2+3]**

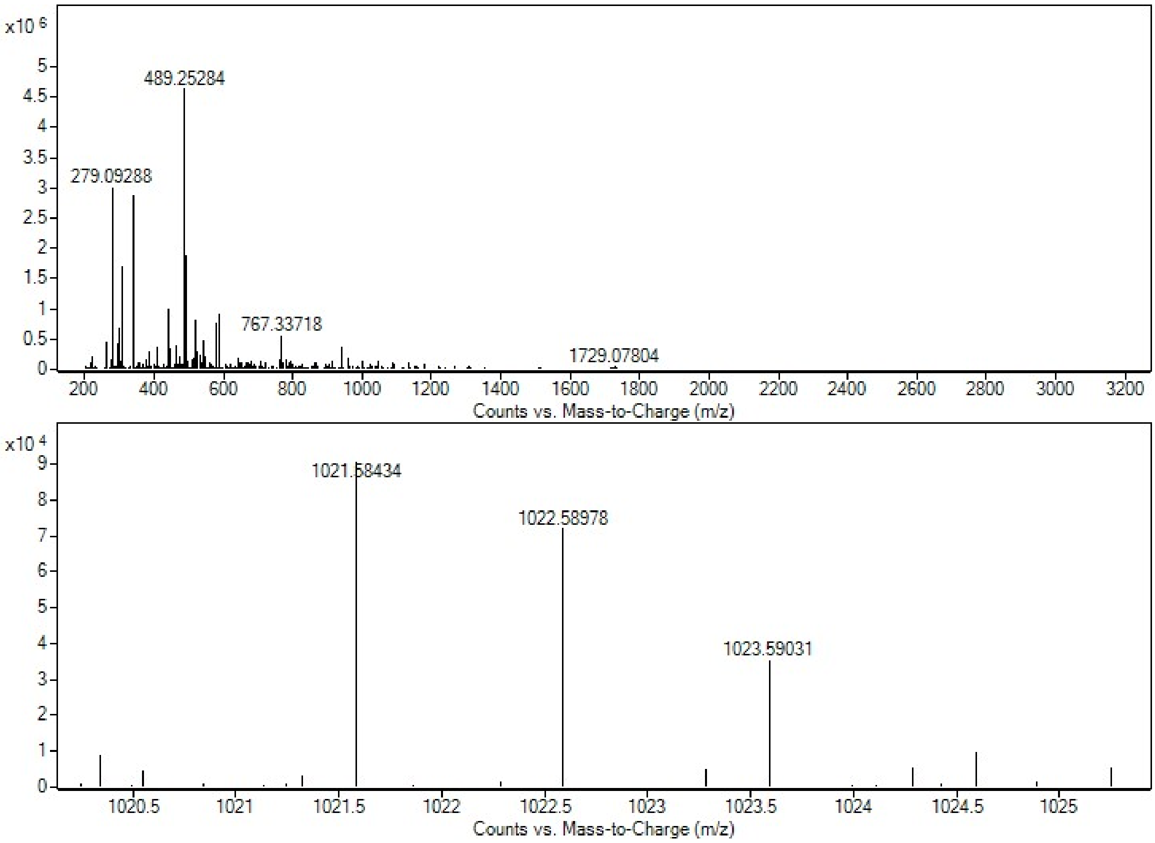

Figure S103: HRMS spectrum for **H26[2+3]**

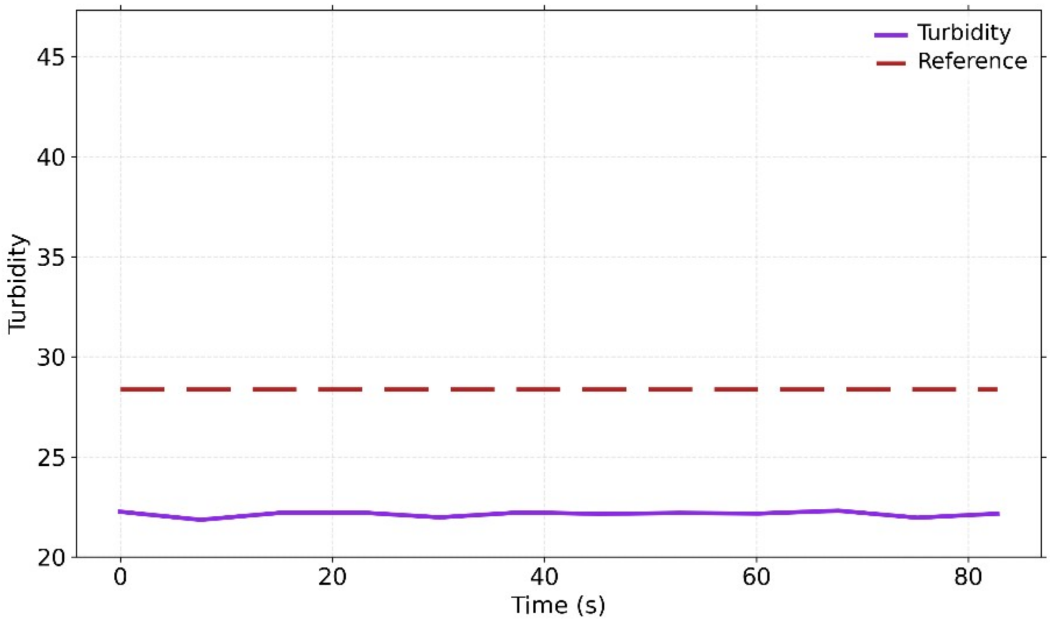

Figure S104: Turbidity vs time (s) for **H26$_{[2+3]}$**

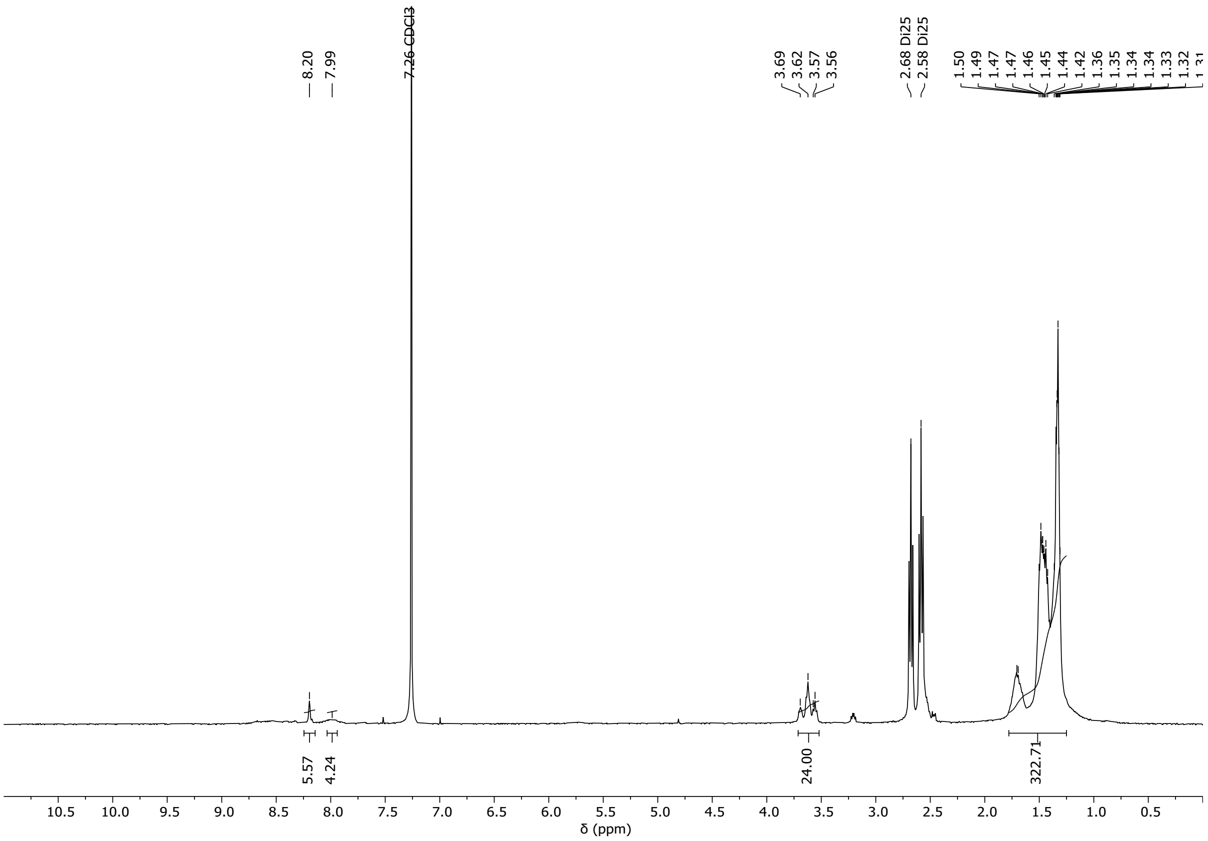

Figure S105: ^{1}H NMR ($CDCl_3$) spectrum for **I25$_{[2+3]}$**

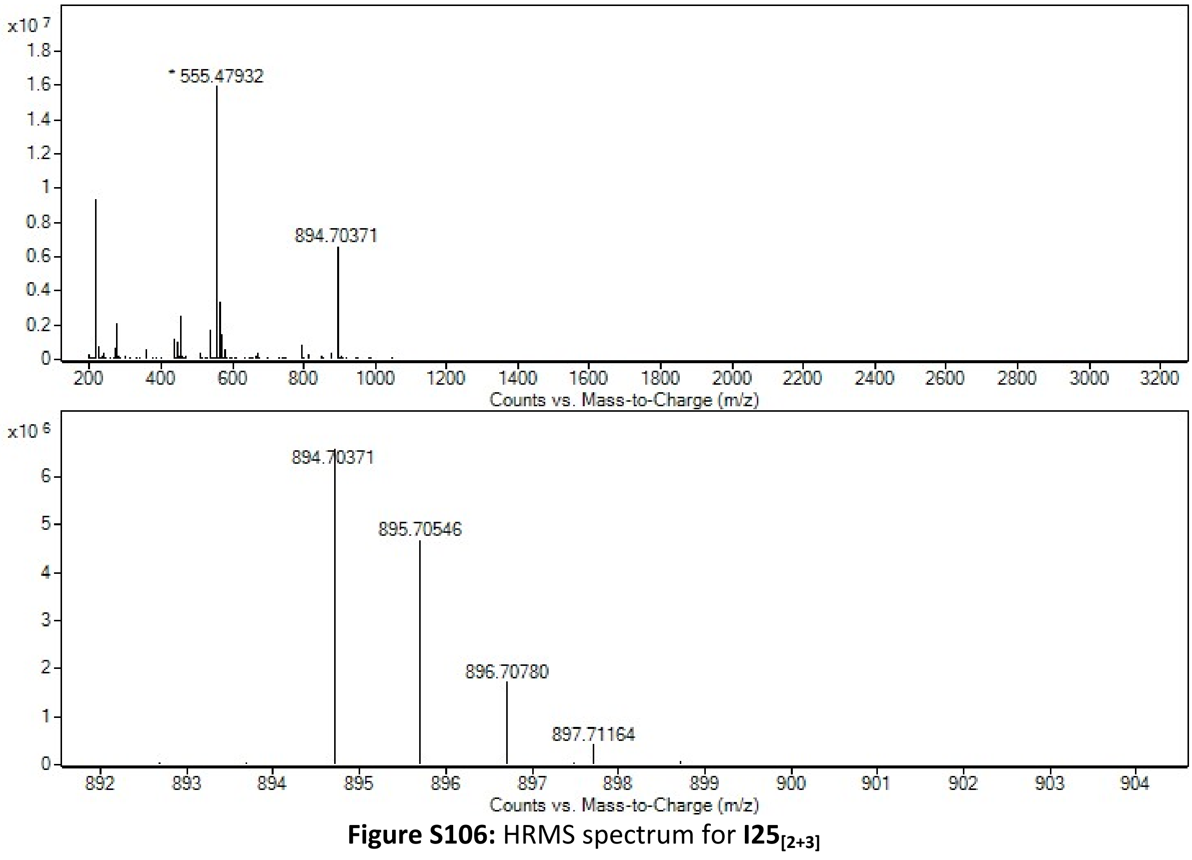

Figure S106: HRMS spectrum for **I25$_{[2+3]}$**

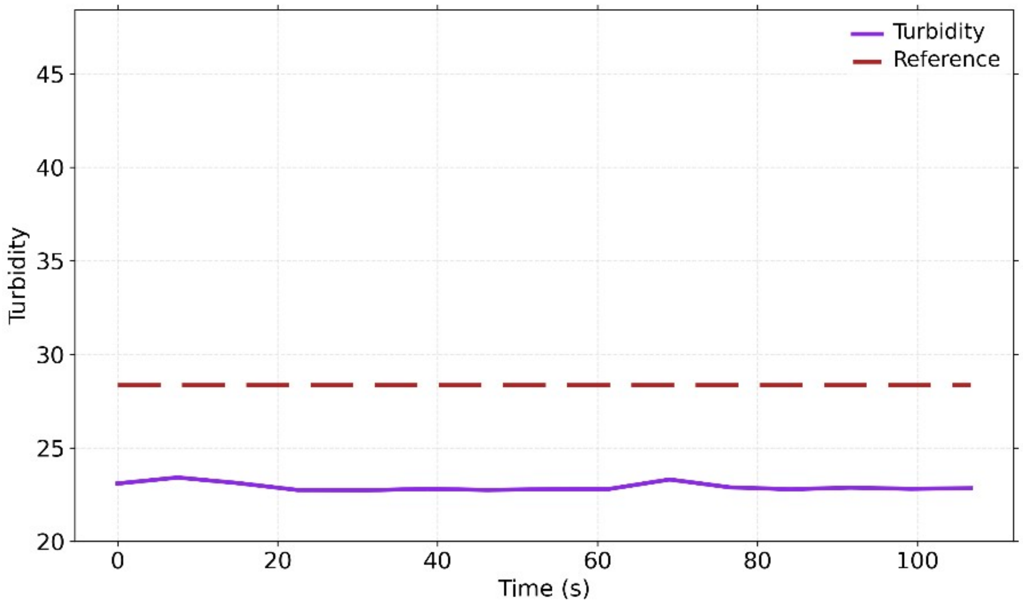

Figure S107: Turbidity vs time (s) for **I25$_{[2+3]}$**

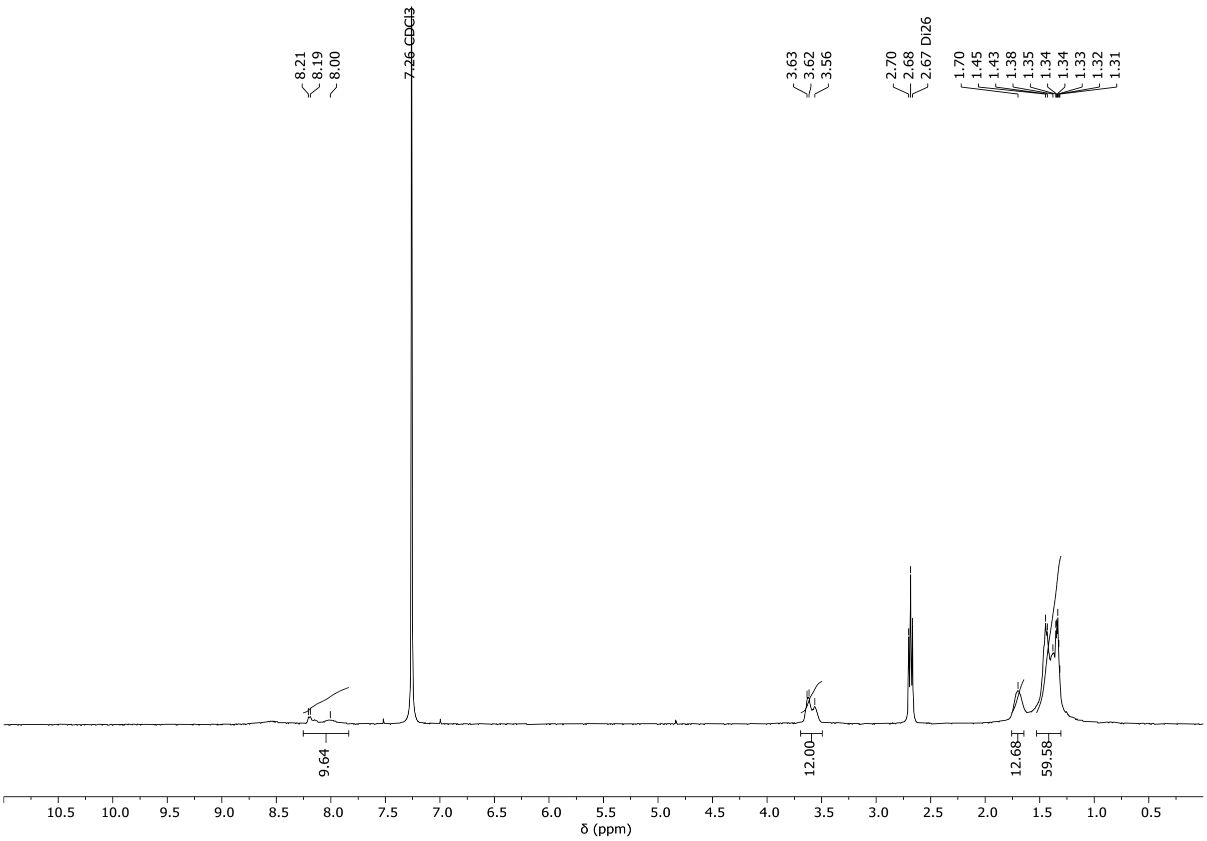

Figure S108: ^{1}H NMR ($CDCl_3$) spectrum for **I26$_{[2+3]}$**

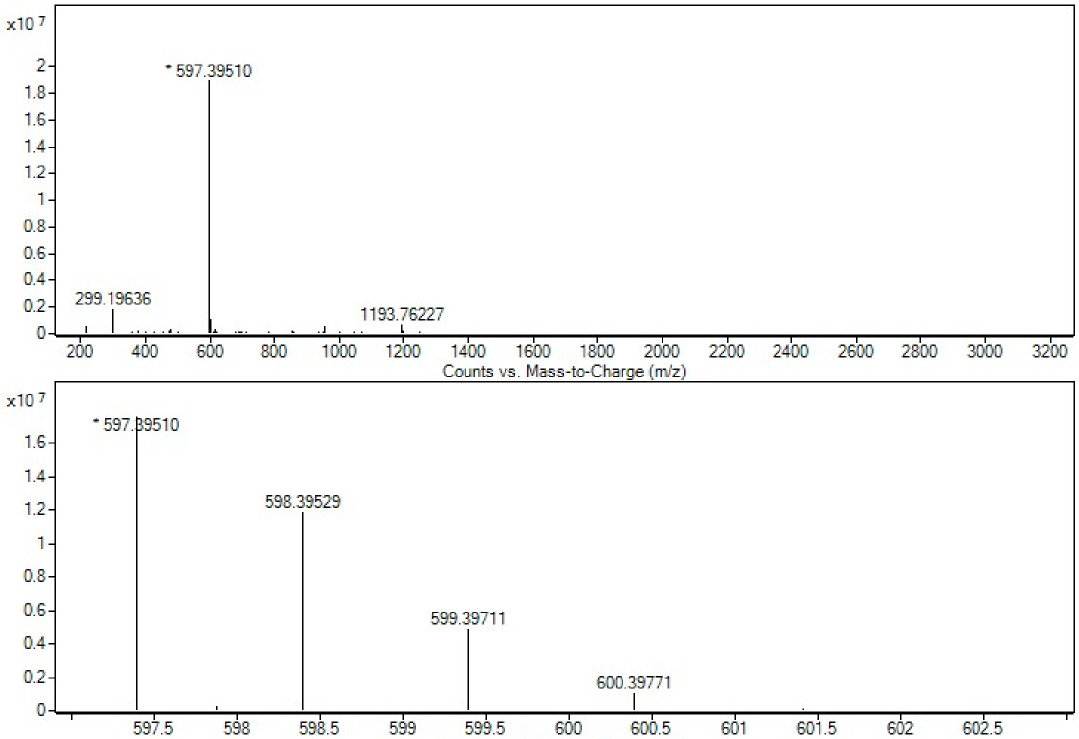

Figure S109: HRMS spectrum for **I26$_{[2+3]}$**

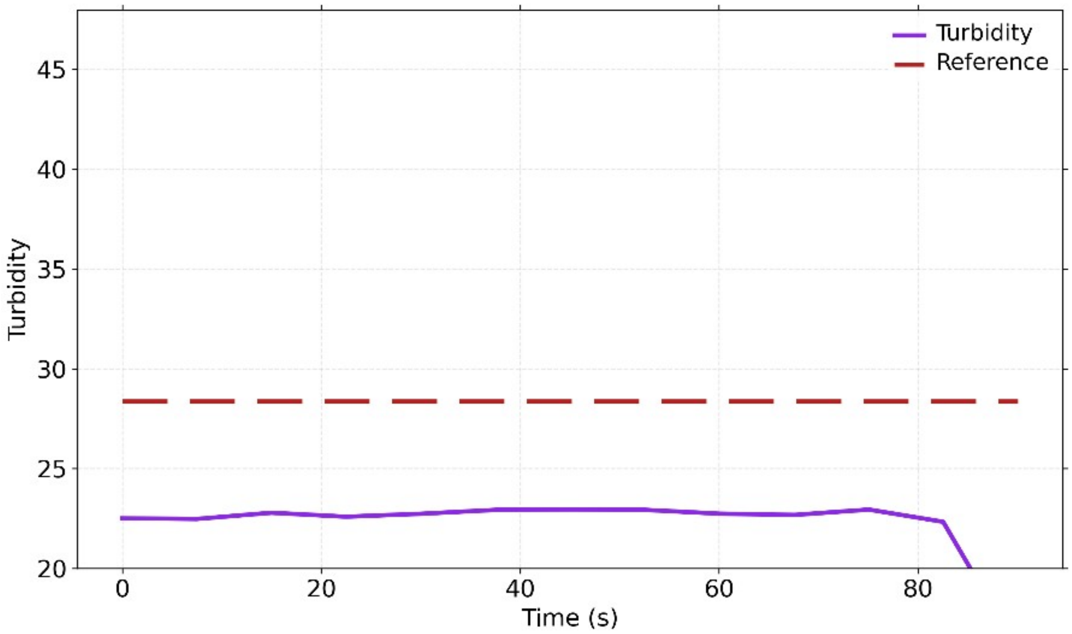

Figure S110: Turbidity vs time (s) for **I26$_{[2+3]}$**

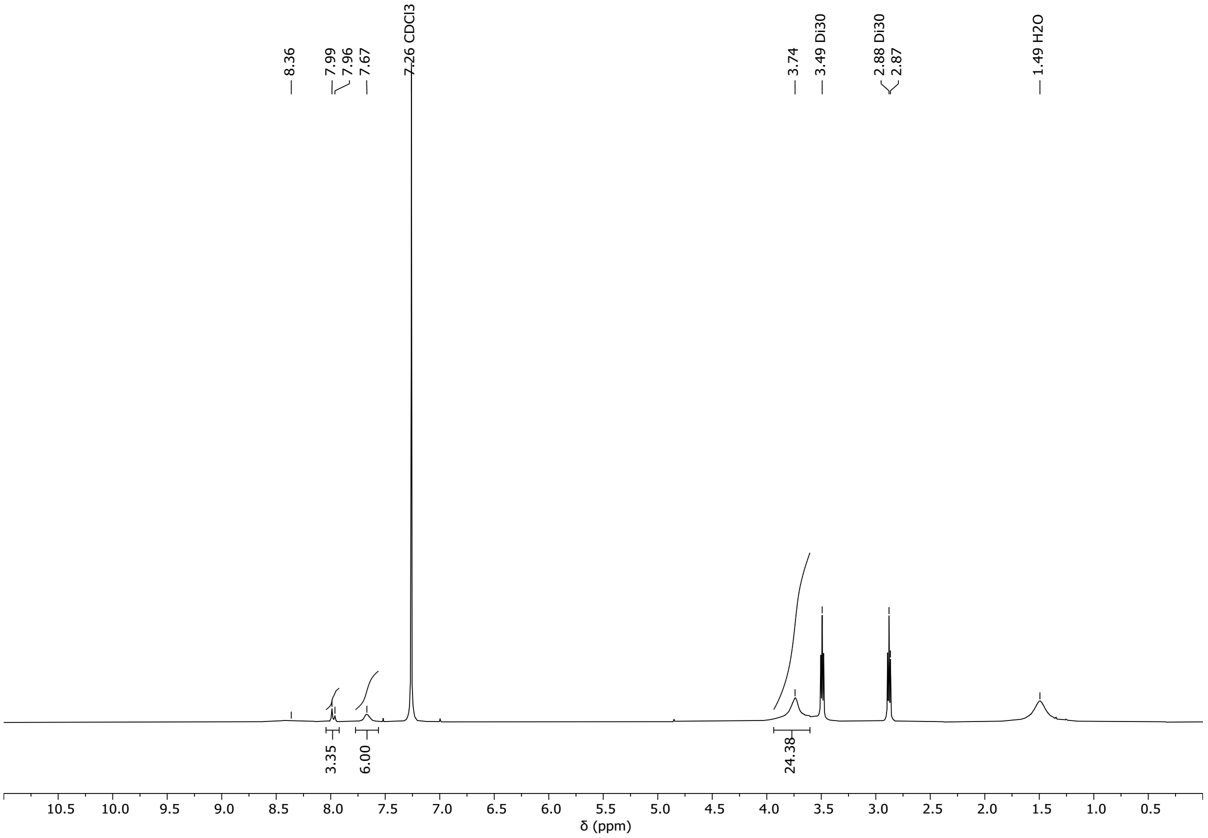

Figure S111: ^{1}H NMR ($CDCl_3$) spectrum for **I30$_{[2+3]}$**

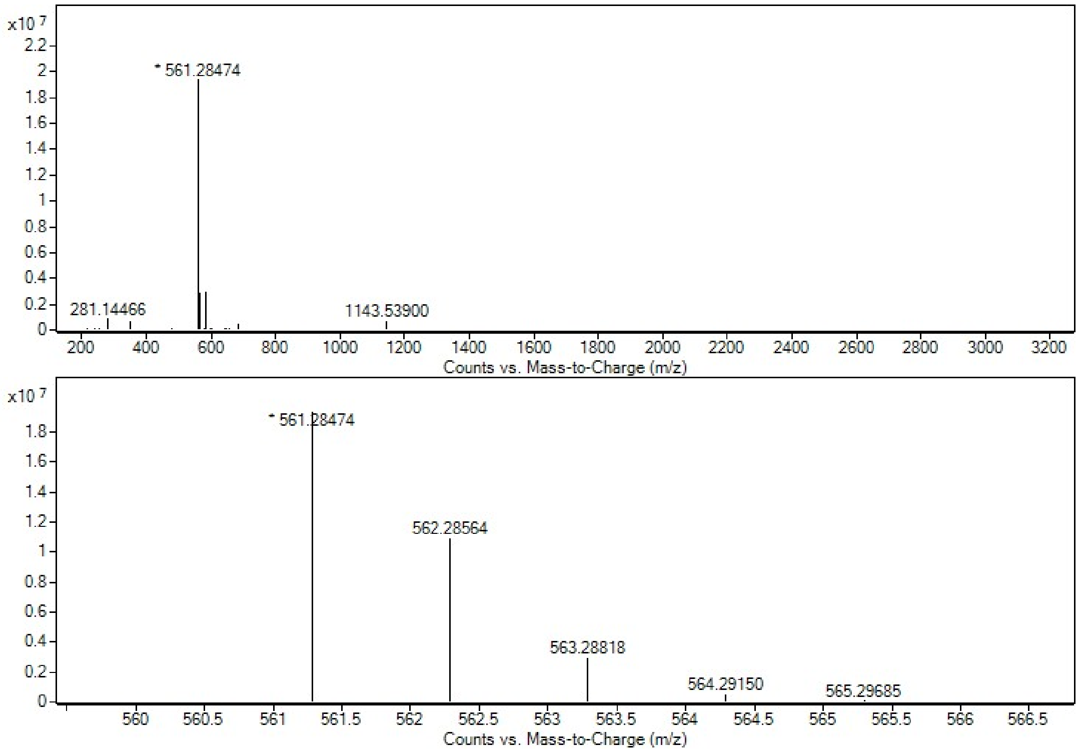

Figure S112: HRMS spectrum for **I30$_{[2+3]}$**

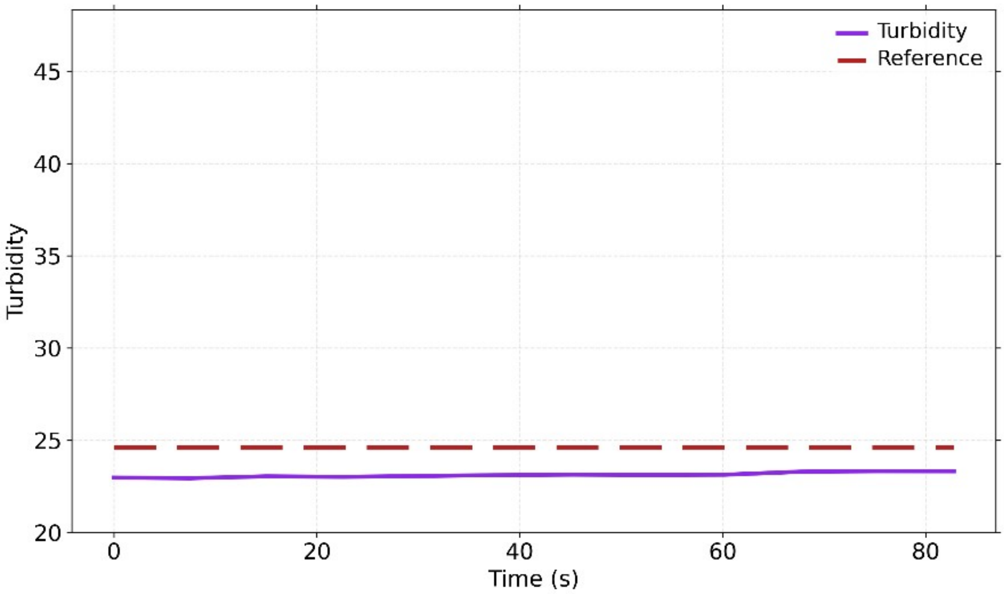

Figure S113: Turbidity vs time (s) for **I30$_{[2+3]}$**

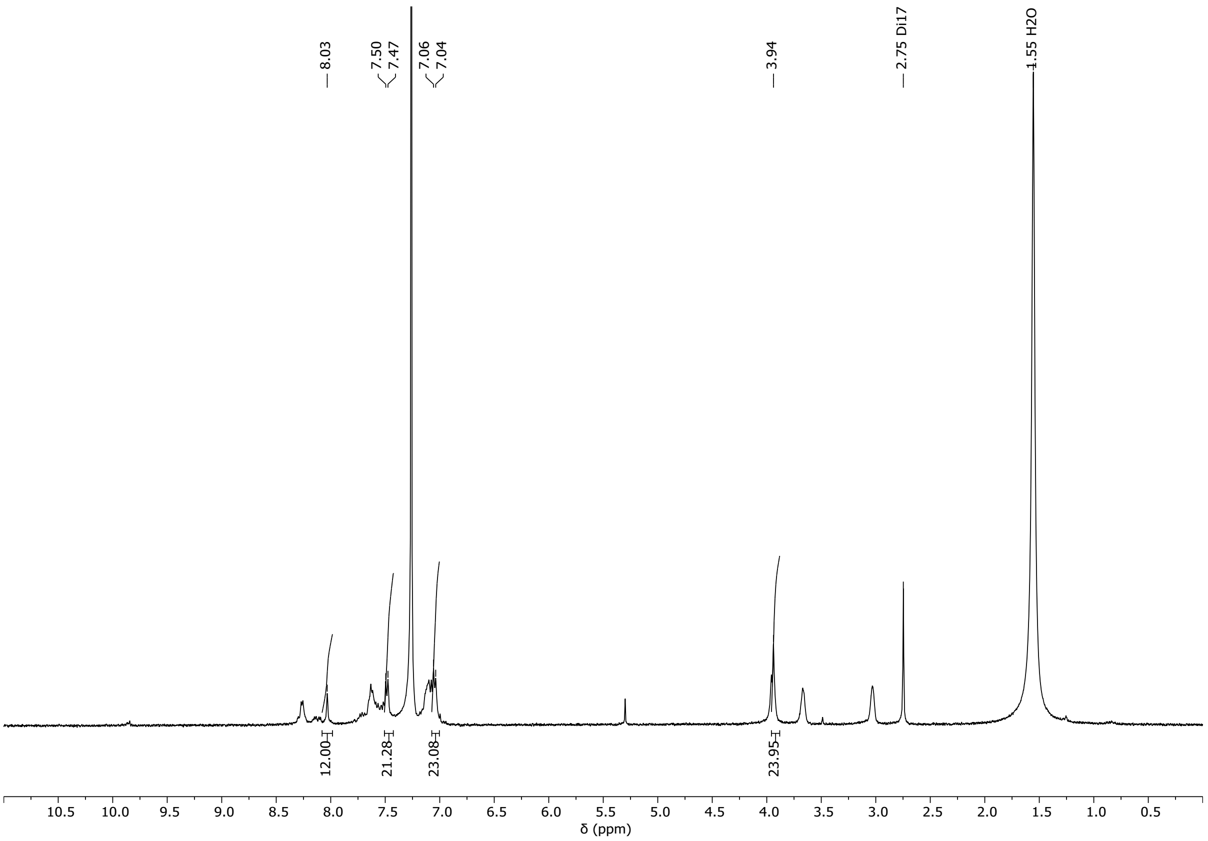

Figure S114: ^{1}H NMR ($CDCl_3$) spectrum for $\mathbf{J17}_{[4+6]}$

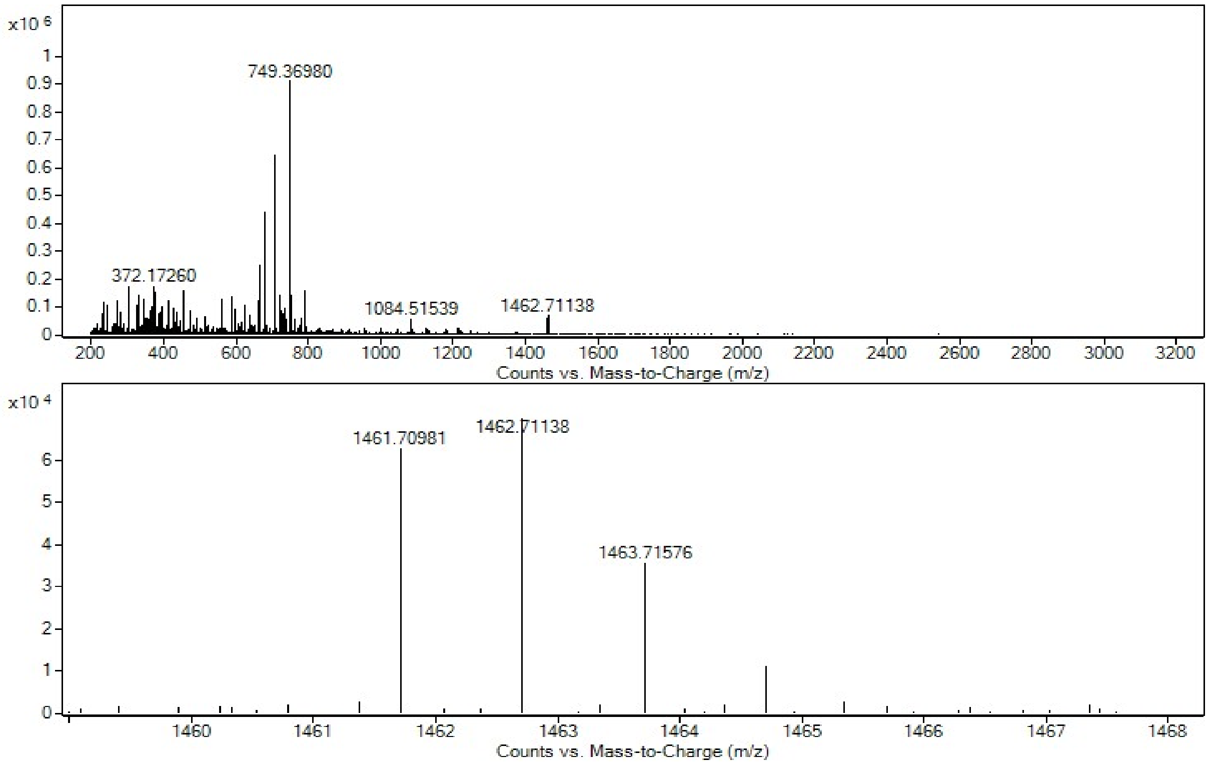

Figure S115: HRMS spectrum for $\mathbf{J17}_{[4+6]}$

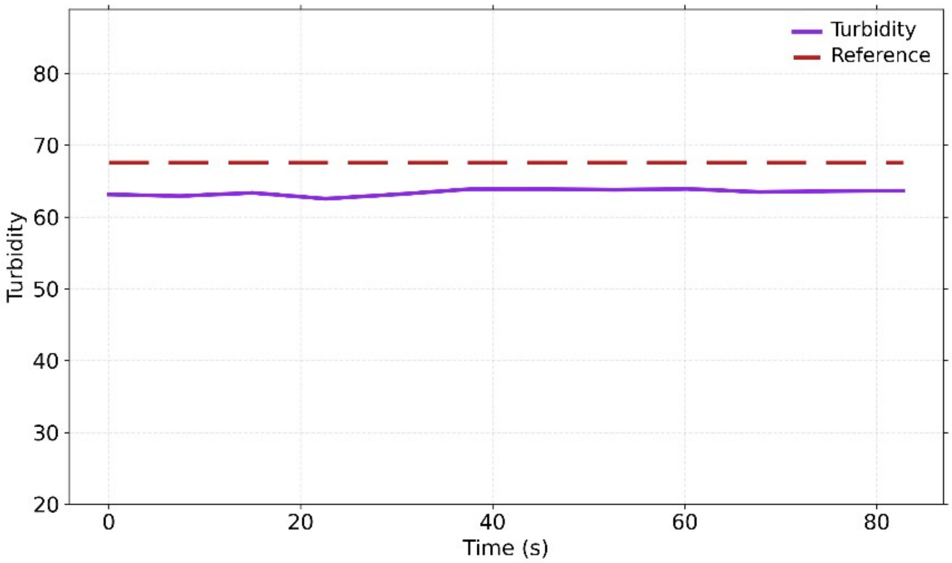

Figure S116: Turbidity vs time (s) for **J17$_{[4+6]}$**

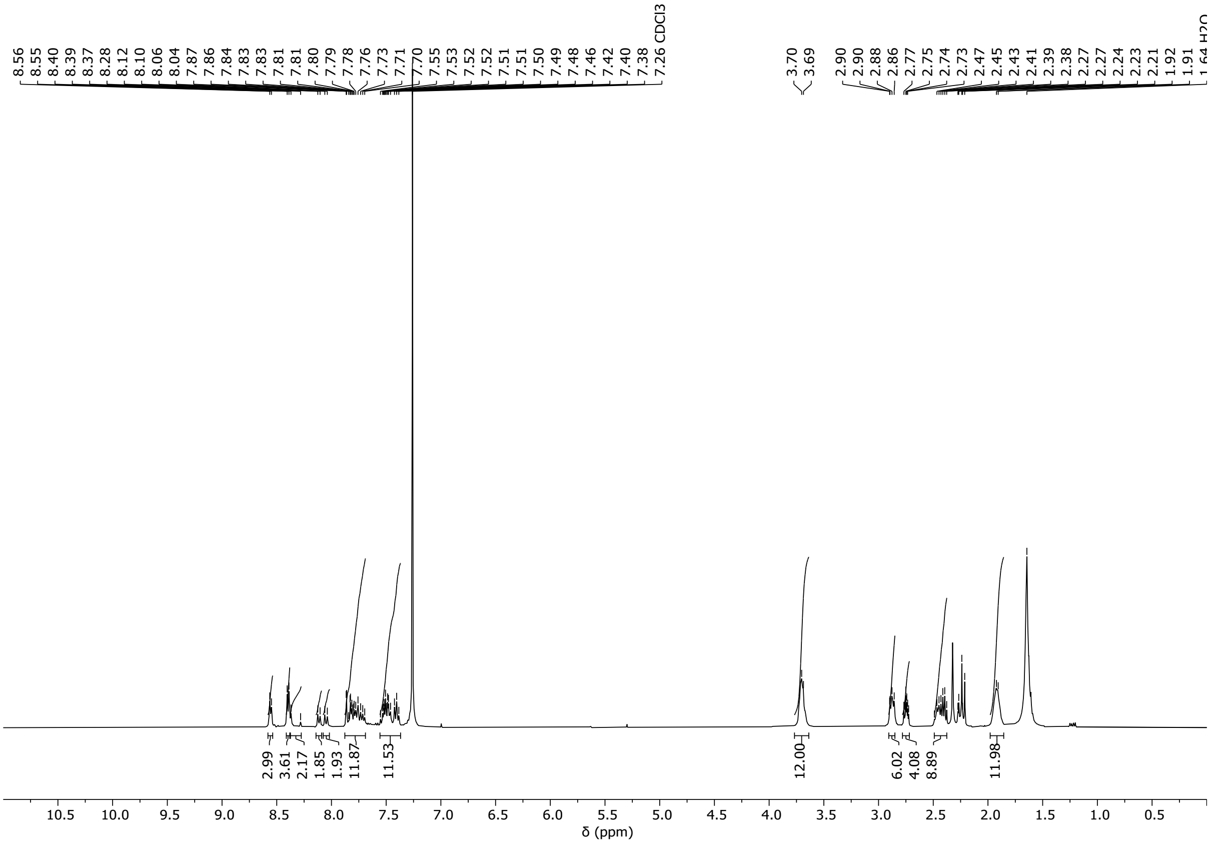

Figure S117: ^{1}H NMR ($CDCl_3$) spectrum for **M28$_{[2+3]}$**

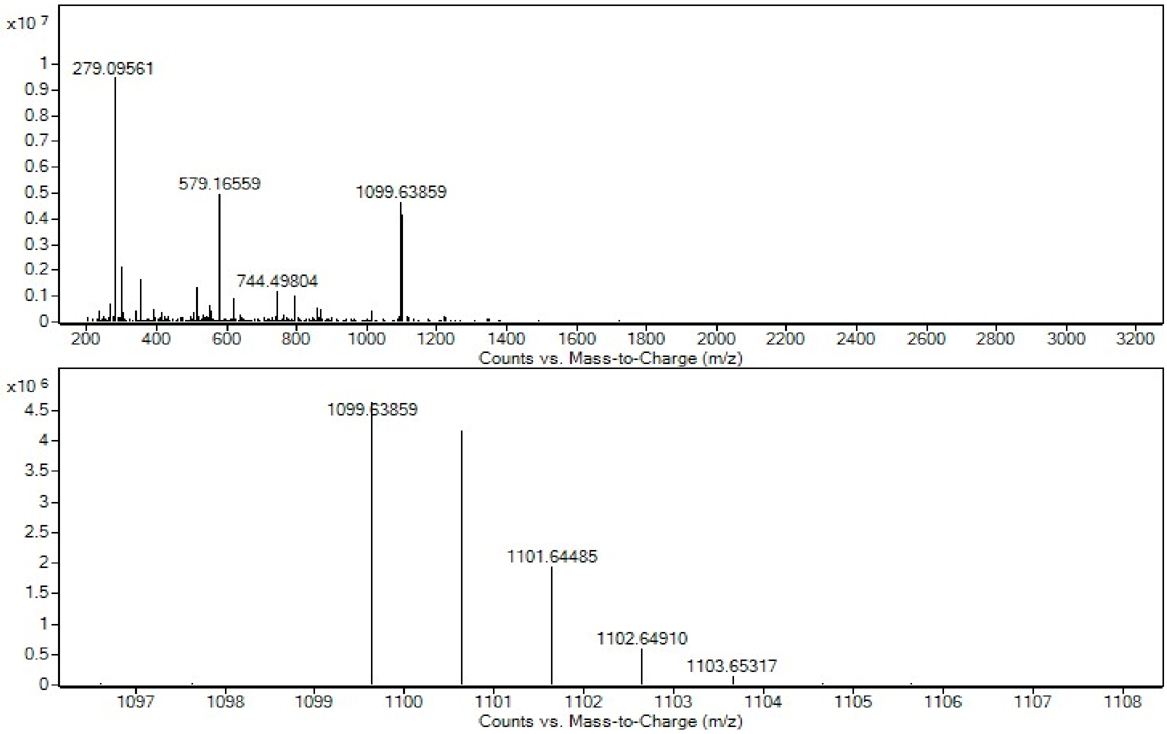

Figure S118: HRMS spectrum for **M28$_{[2+3]}$**

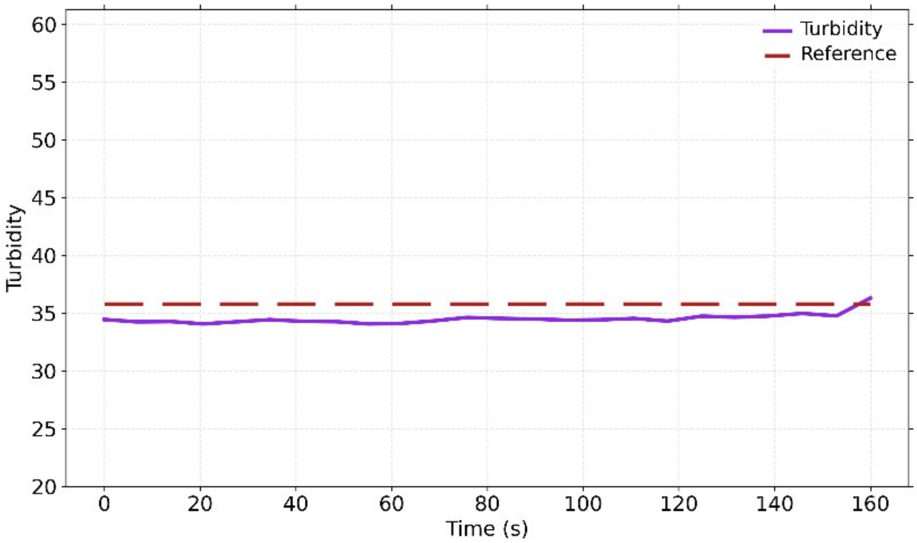

Figure S119: Turbidity vs time (s) for **M28**$_{[2+3]}$

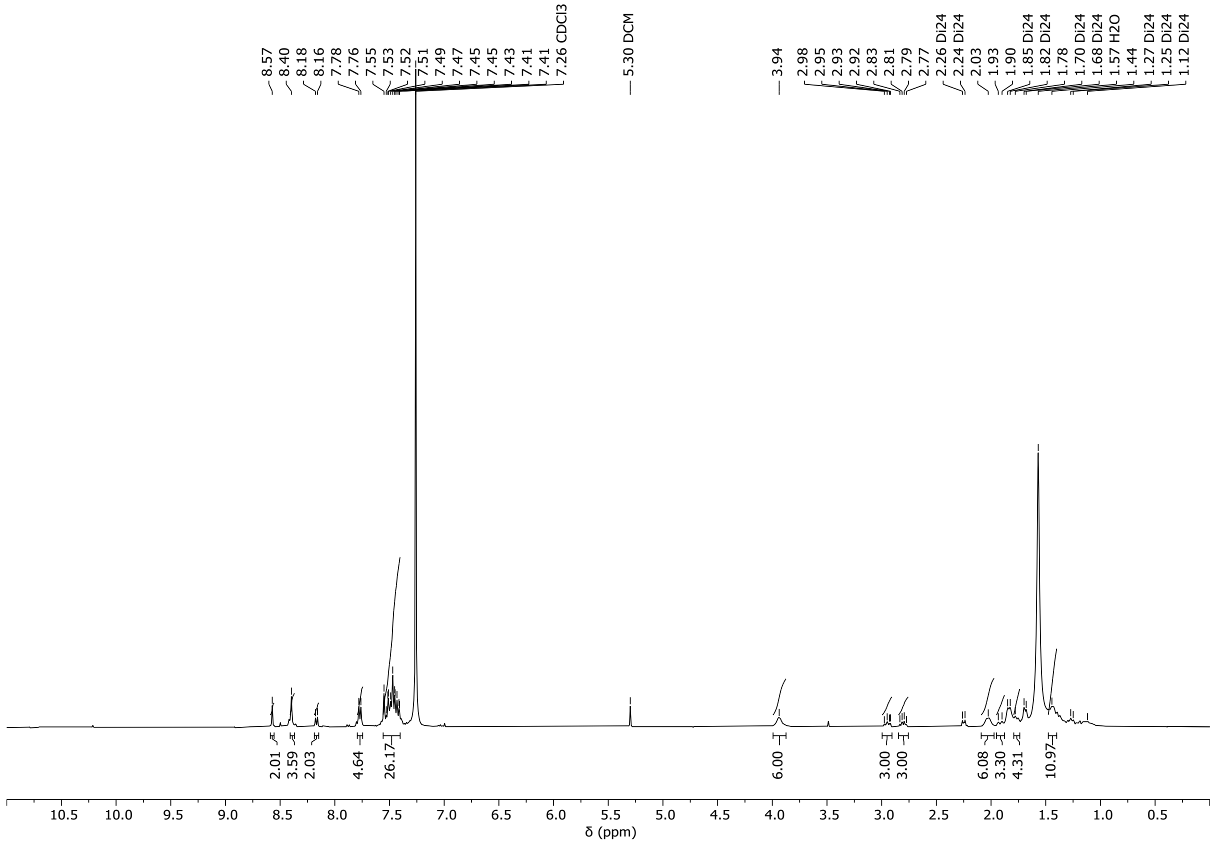

Figure S120: ^1H NMR ($CDCl_3$) spectrum for **N24$_{[2+3]}$**

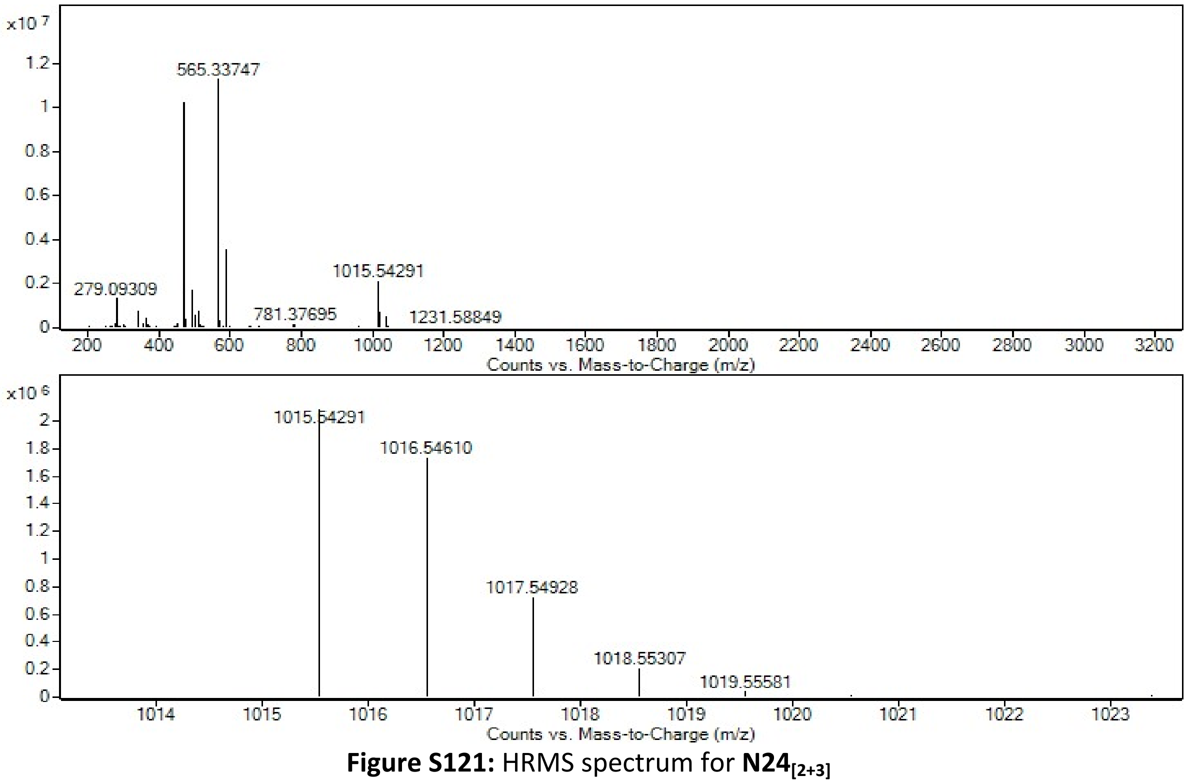

Figure S121: HRMS spectrum for **N24$_{[2+3]}$**

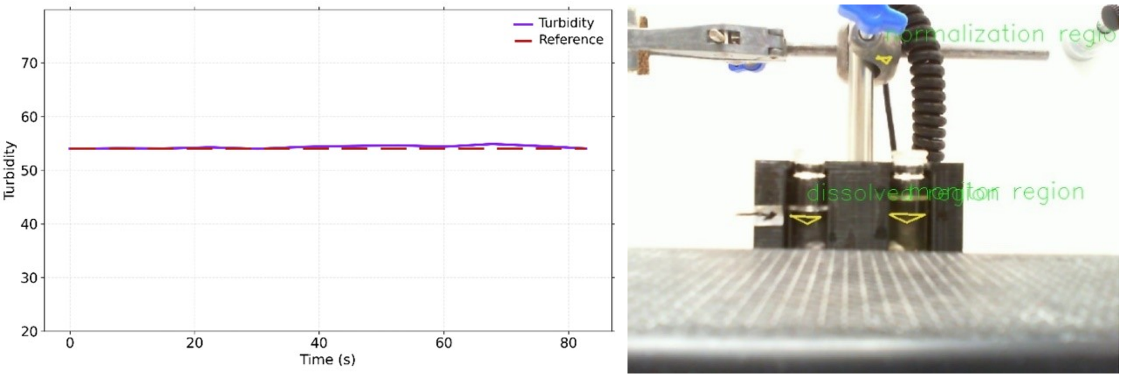

Figure S122: Turbidity vs time (s) for $\mathbf{N24}_{[2+3].}$ Sample turbidity remained within the standard error for the turbidity reference throughout the measurement, also confirmed by a human visual check, and therefore passed the turbidity check.

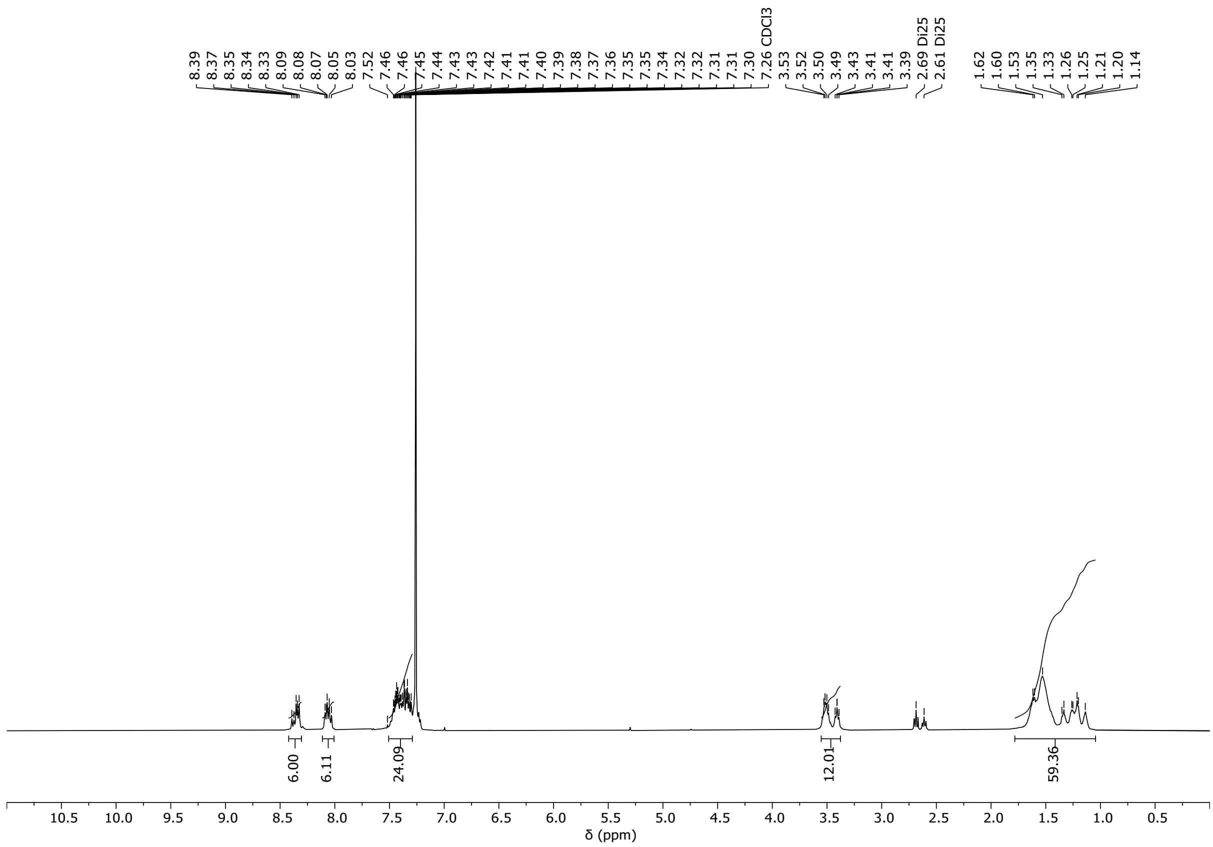

Figure S123: ^{1}H NMR ($CDCl_3$) spectrum for **N25$_{[2+3]}$**

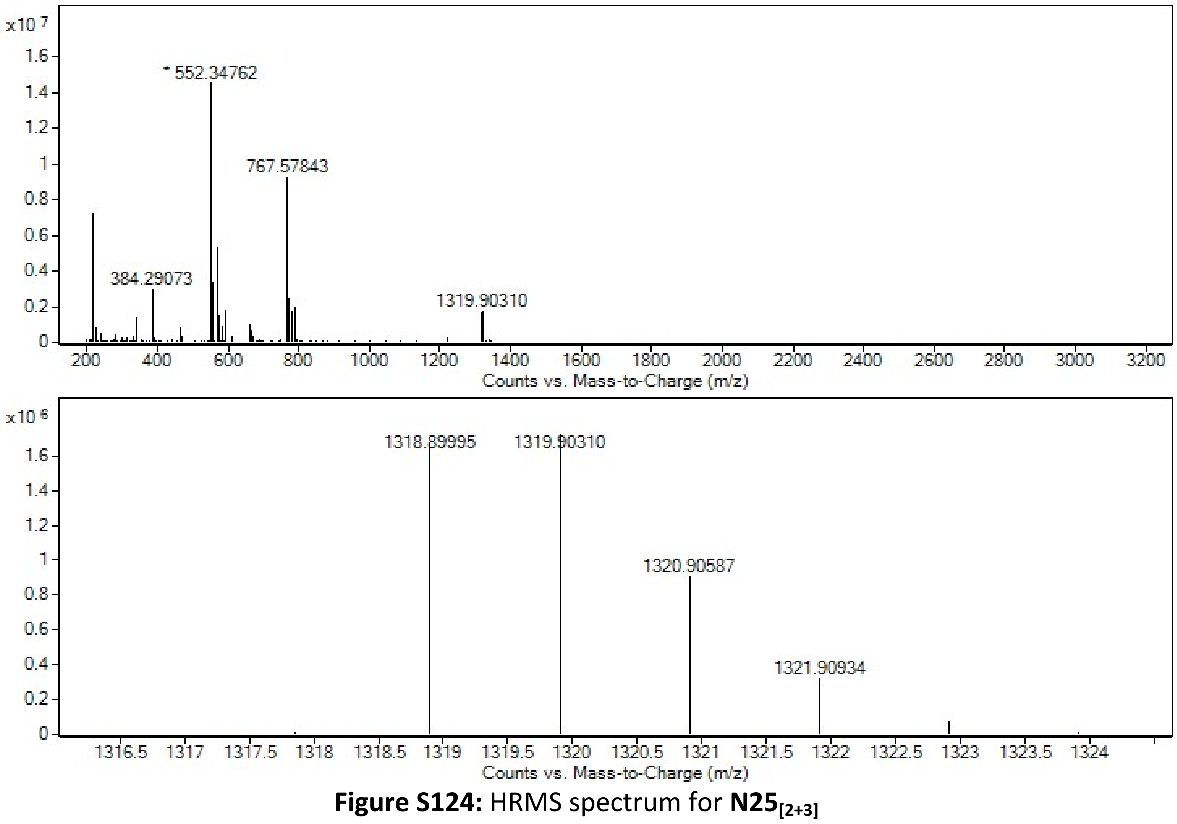

Figure S124: HRMS spectrum for **N25$_{[2+3]}$**

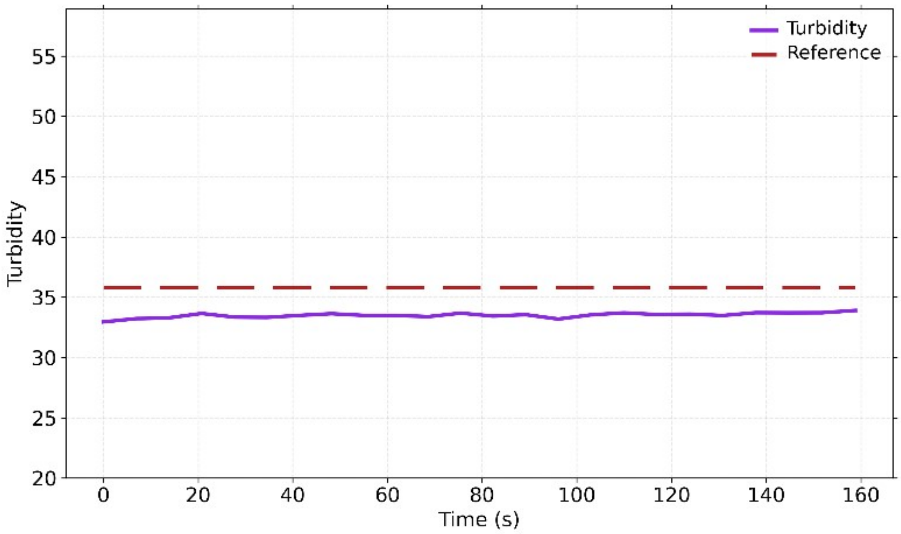

Figure S125: Turbidity vs time (s) for **N25**$_{\mathbf{[2+3]}}$

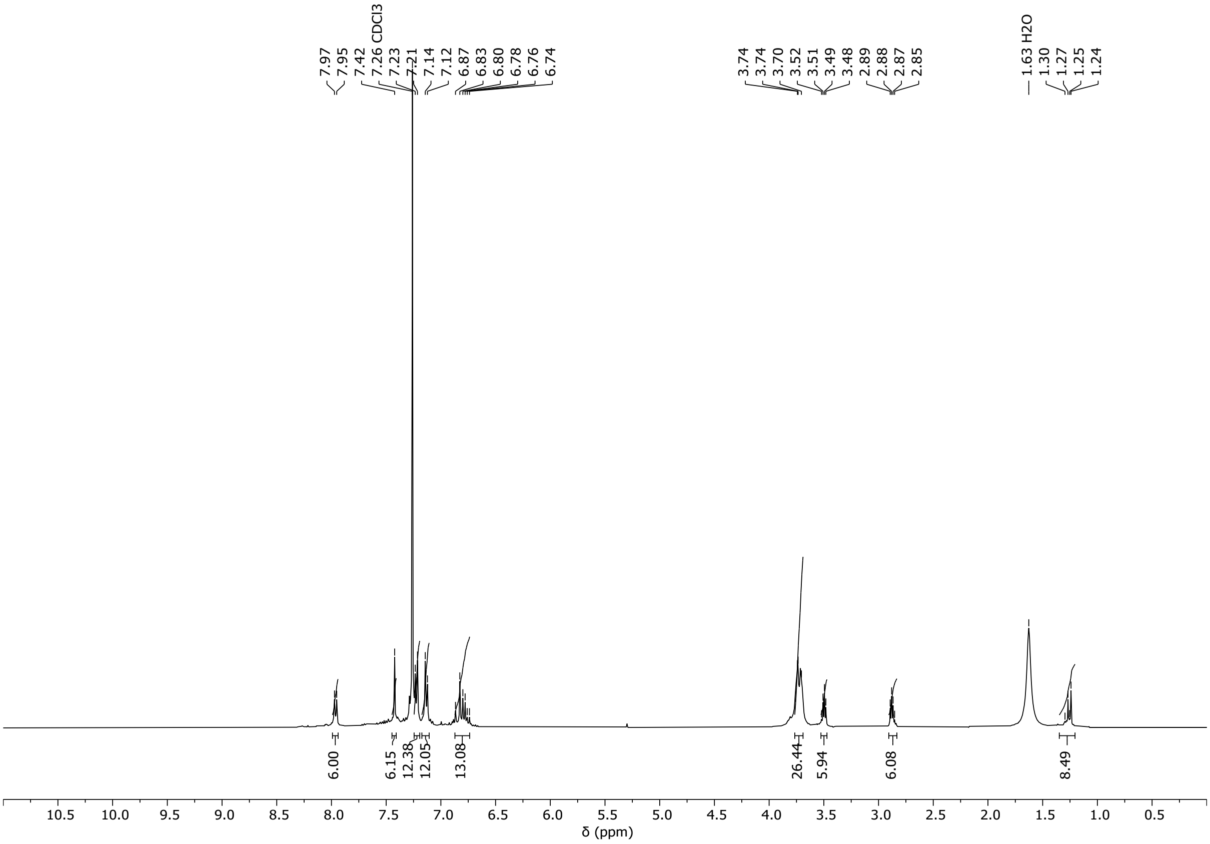

Figure S126: ^{1}H NMR ($CDCl_3$) spectrum for **Q29$_{[2+3]}$** - risk of competitive 1,4- vs 1,2-addition to the unsaturated aldehyde in **Q**.

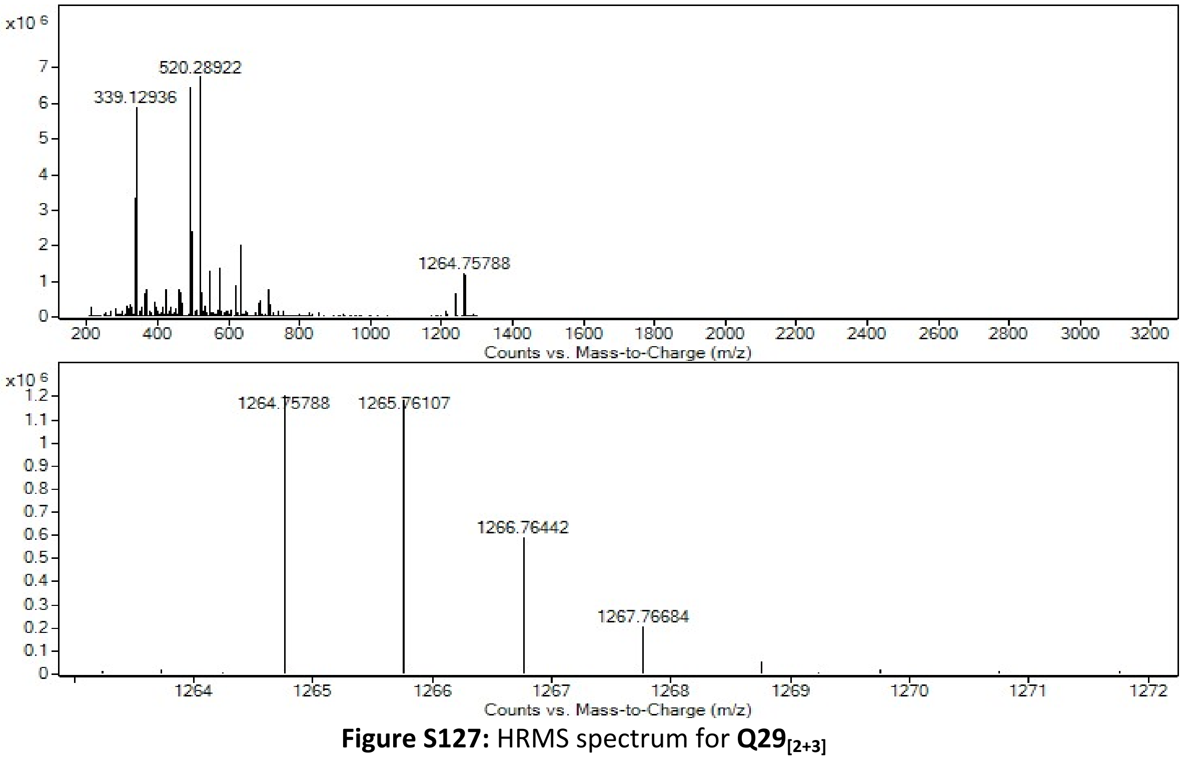

Figure S127: HRMS spectrum for **Q29$_{[2+3]}$**

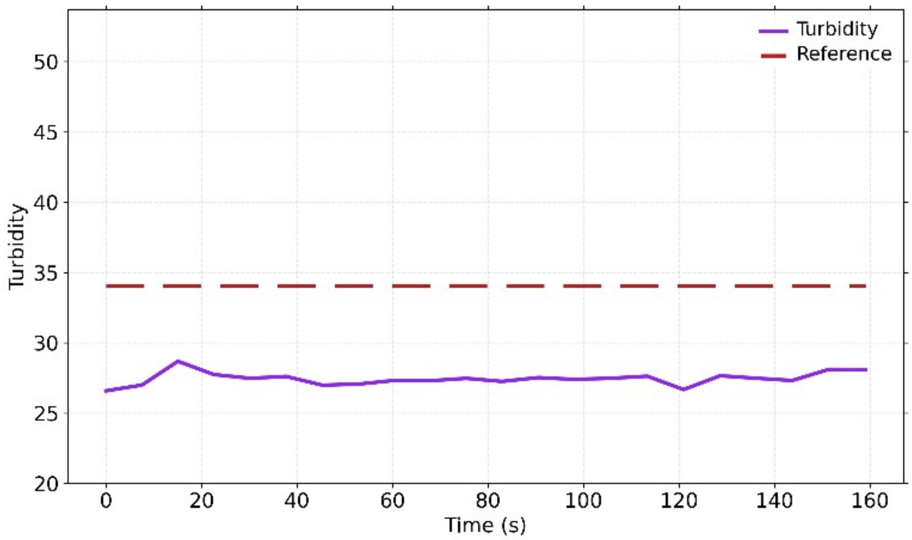

Figure S128: Turbidity vs time (s) for **Q29[2+3]**

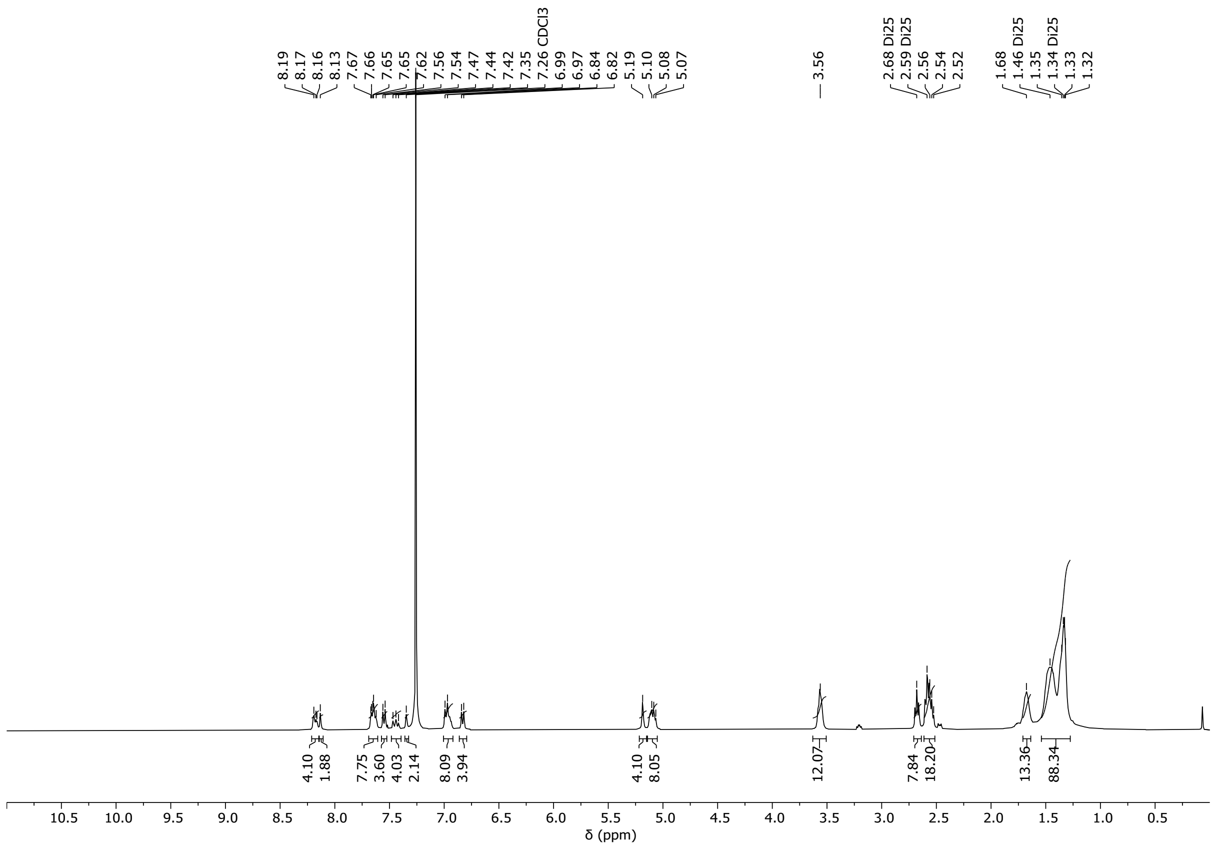

Figure S129: ^{1}H NMR ($CDCl_3$) spectrum for **$R25_{[2+3]}$**

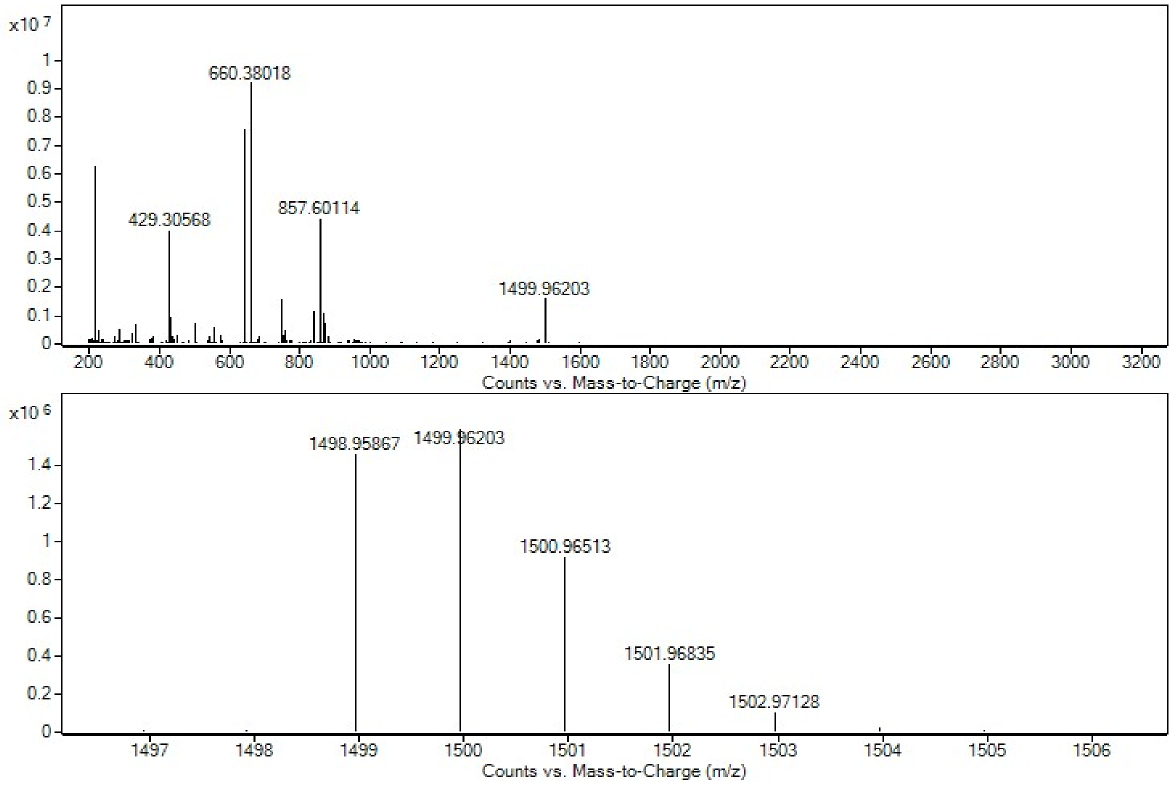

Figure S130: HRMS spectrum for **$R25_{[2+3]}$**

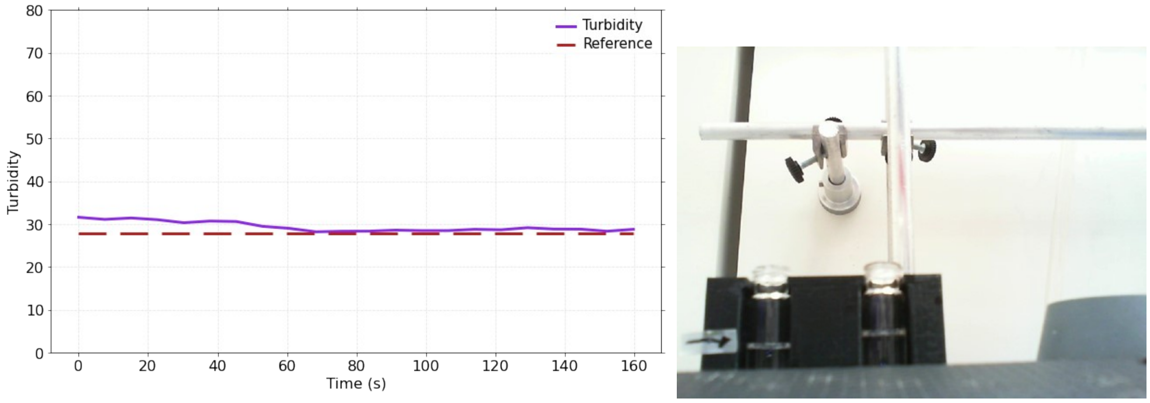

Figure S131: Turbidity vs time (s) for **R25**$_{[2+3]}$. Sample turbidity remained within the standard error for the turbidity reference throughout measurement, also confirmed by a human visual check, and therefore passed the turbidity check.

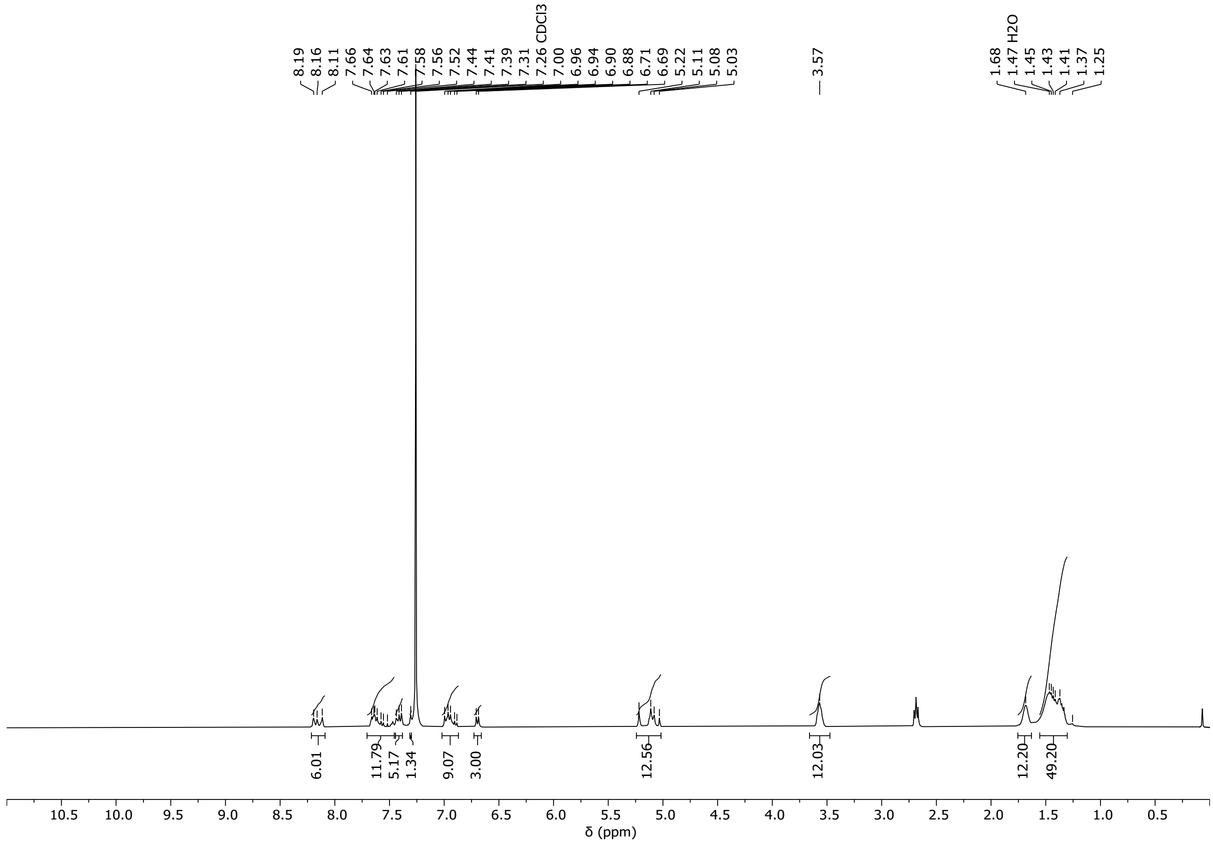

Figure S132: ^{1}H NMR ($CDCl_3$) spectrum for **R26$_{[2+3]}$**

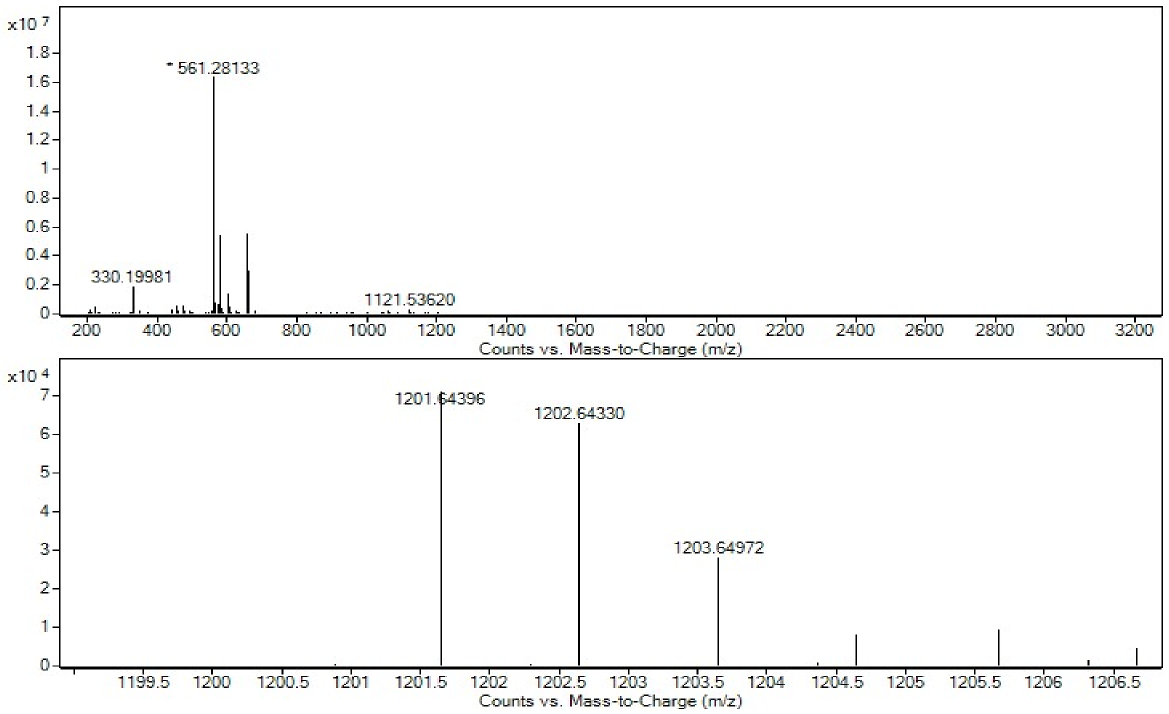

Figure S133: HRMS spectrum for **R26$_{[2+3]}$**

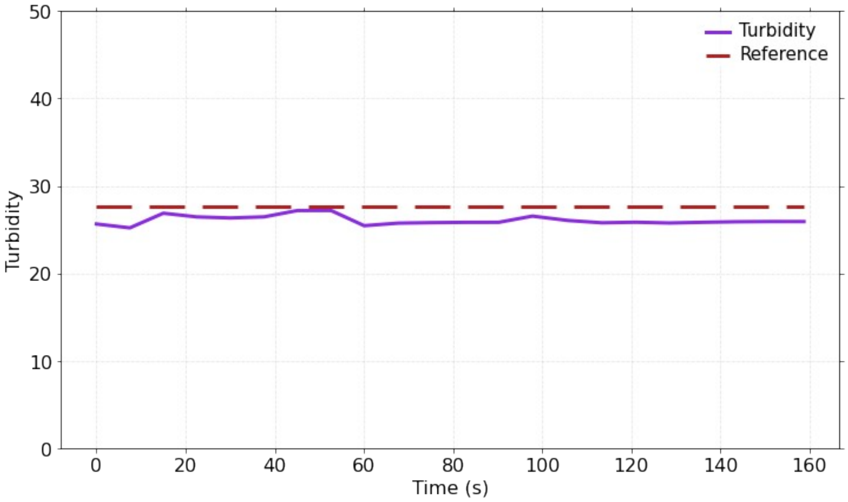

Figure S134: Turbidity vs time (s) for **R26$_{2+3]}$**

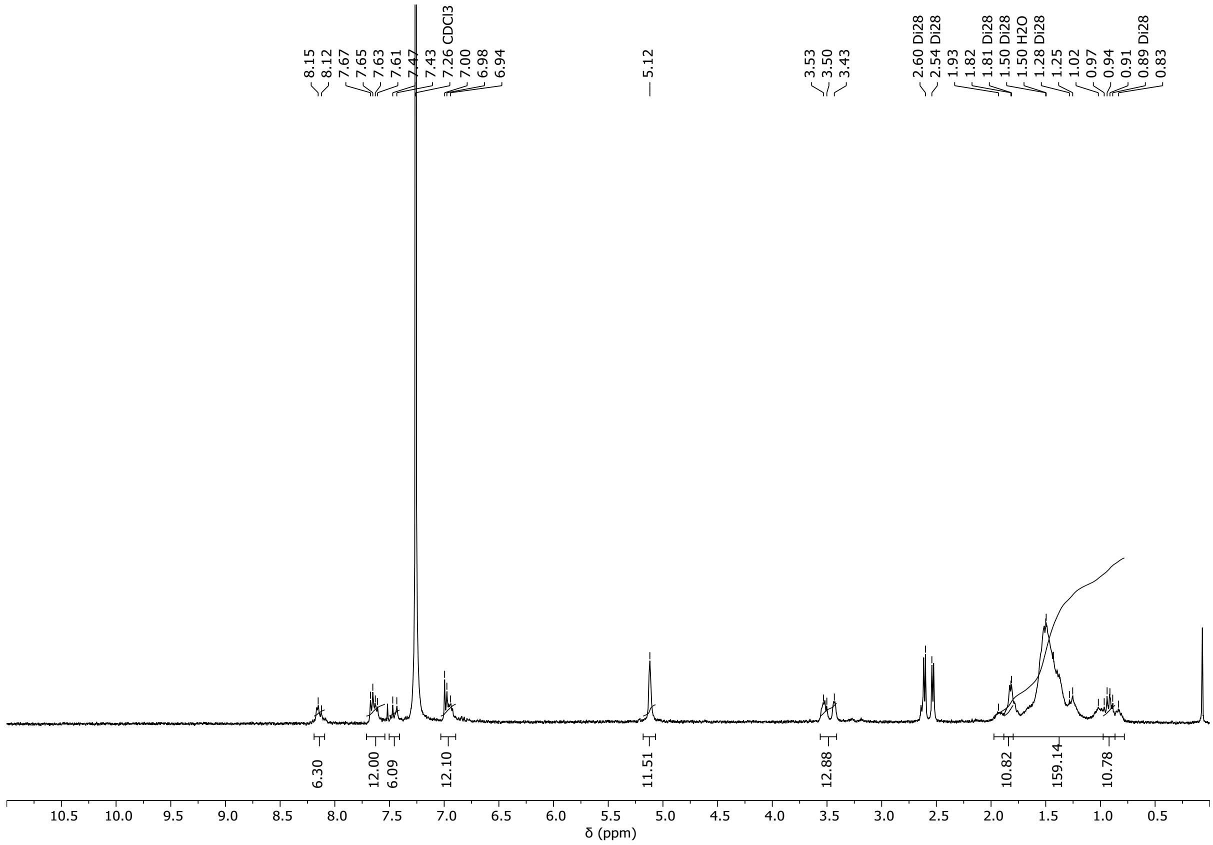

Figure S135: ^{1}H NMR ($CDCl_3$) spectrum for **R28$_{[2+3]}$**

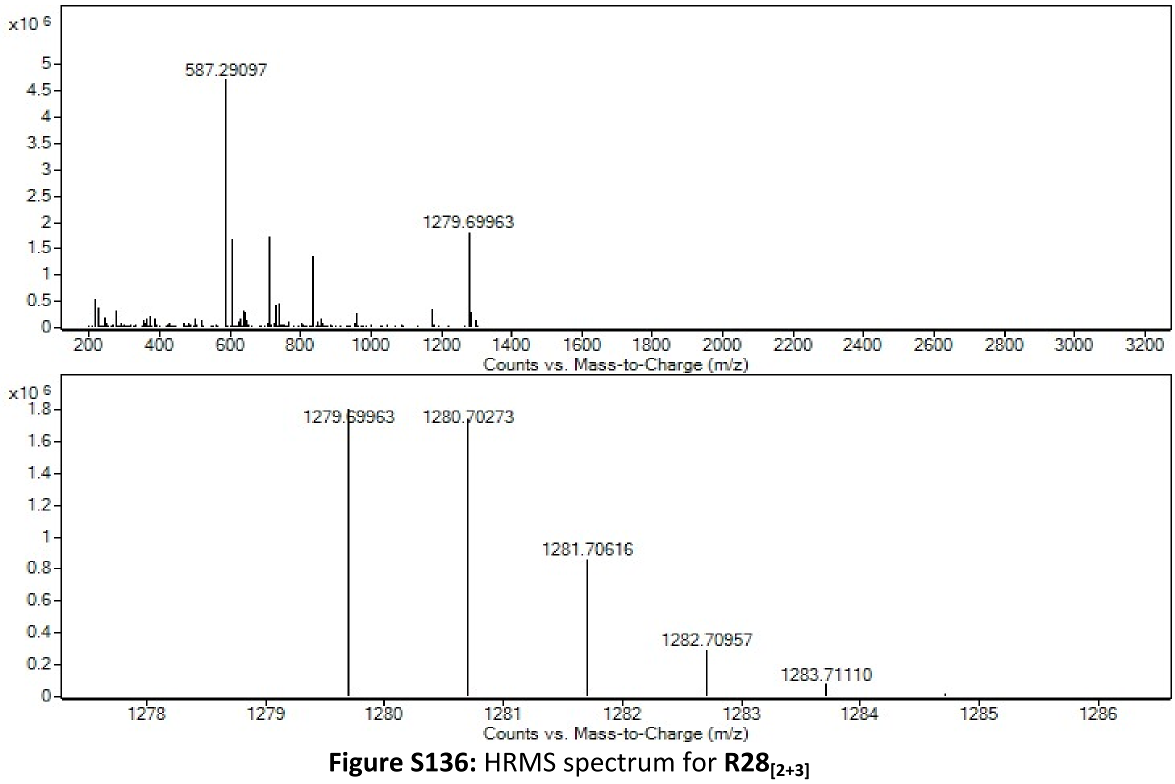

Figure S136: HRMS spectrum for **R28$_{[2+3]}$**

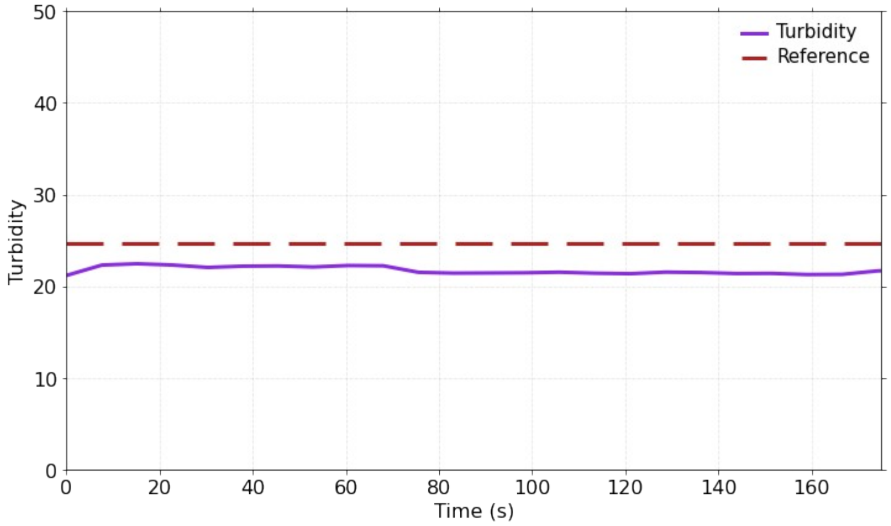

Figure S137: Turbidity vs time (s) for $\mathbf{R28}_{[2+3]}$.

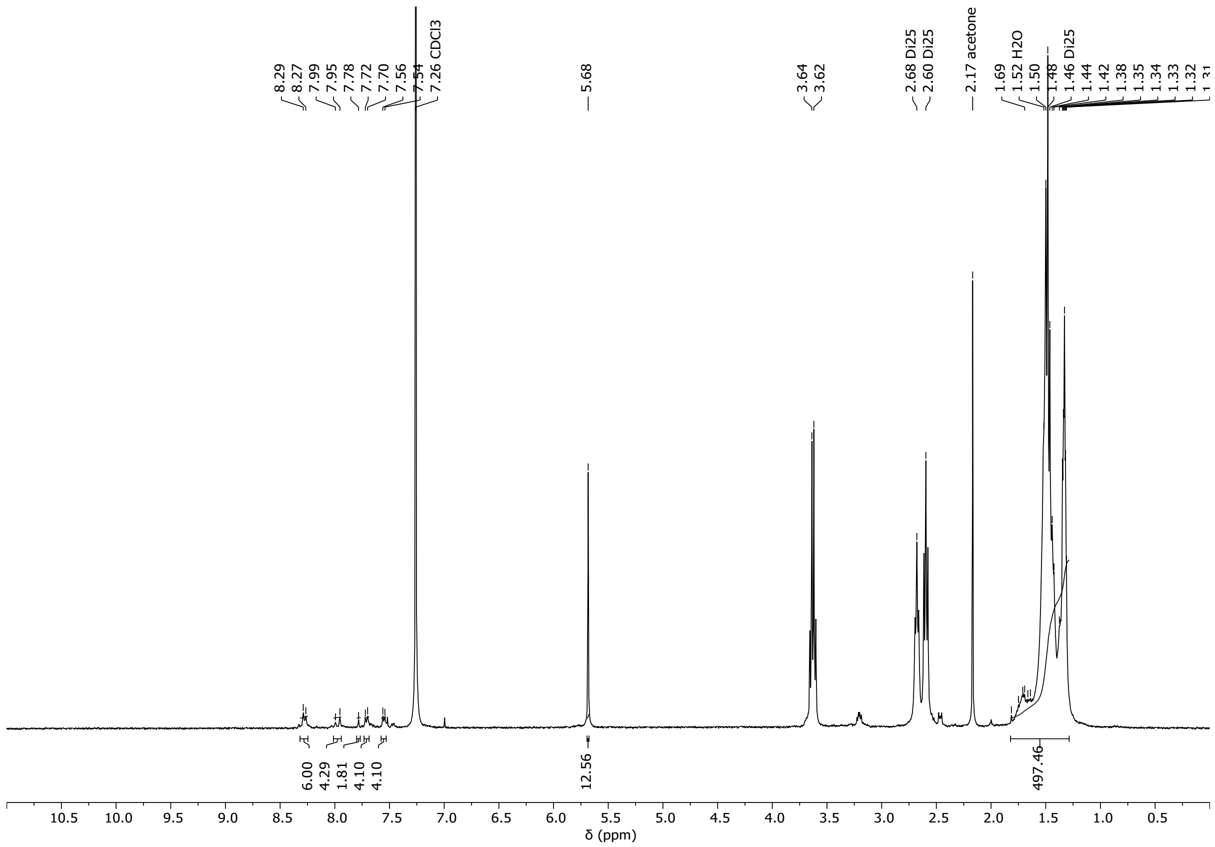

Figure S138: ^{1}H NMR ($CDCl_3$) spectrum for **S25[2+3]**

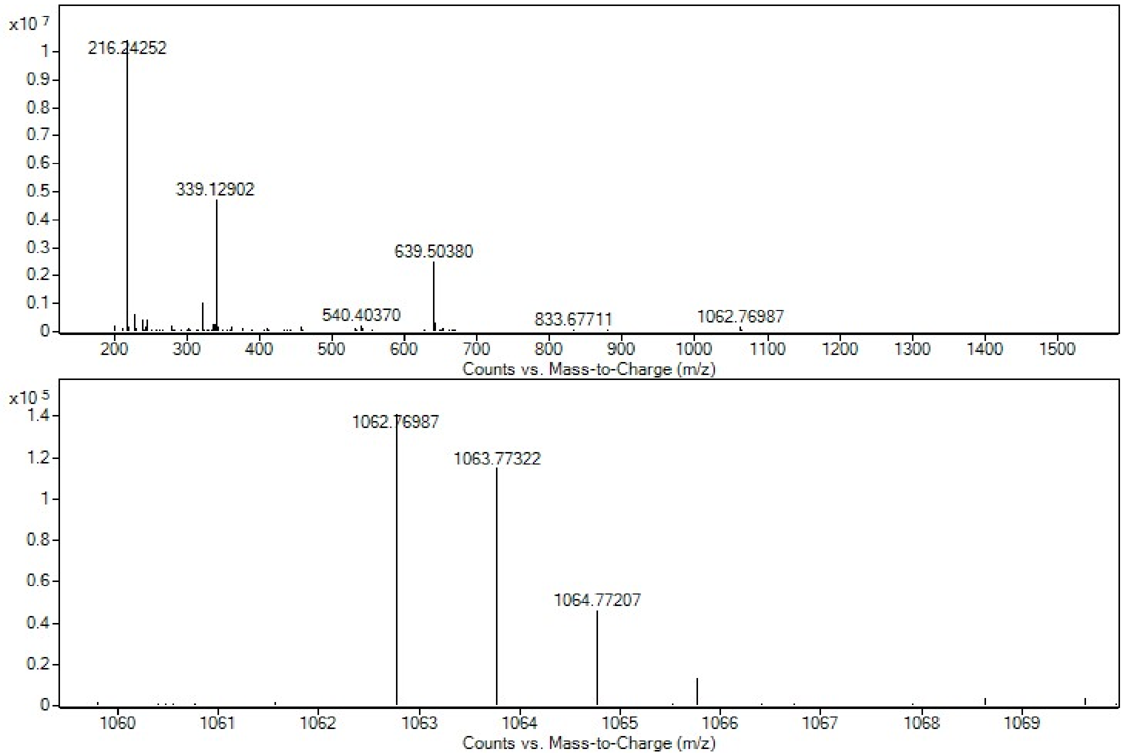

Figure S139: HRMS spectrum for **S25[2+3]**

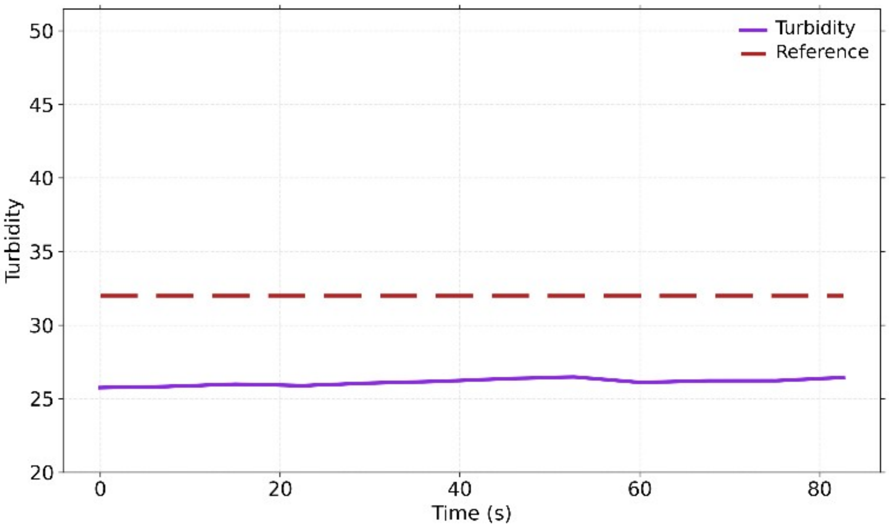

Figure S140: Turbidity vs time (s) for **S25$_{[2+3]}$**

As previously mentioned, while identified as clean hits using the automated analysis, with mass ions corresponding to specific topologies apparent and turbidity measurements confirming everything was in solution, some of the ^{1}H NMR spectra could not be fully assigned which are included here for reference:

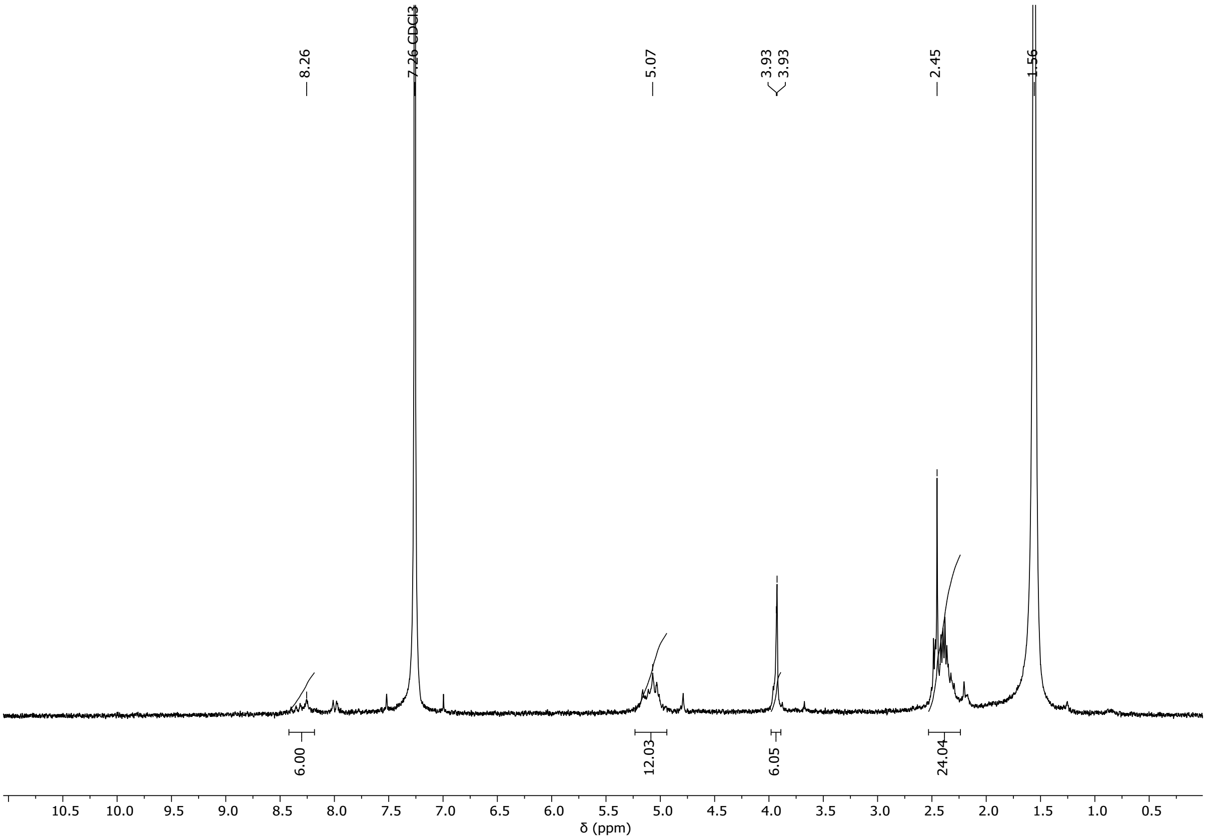

Figure S141: ^{1}H NMR ($CDCl_3$) spectrum for **B5$_{[2+3]}$**

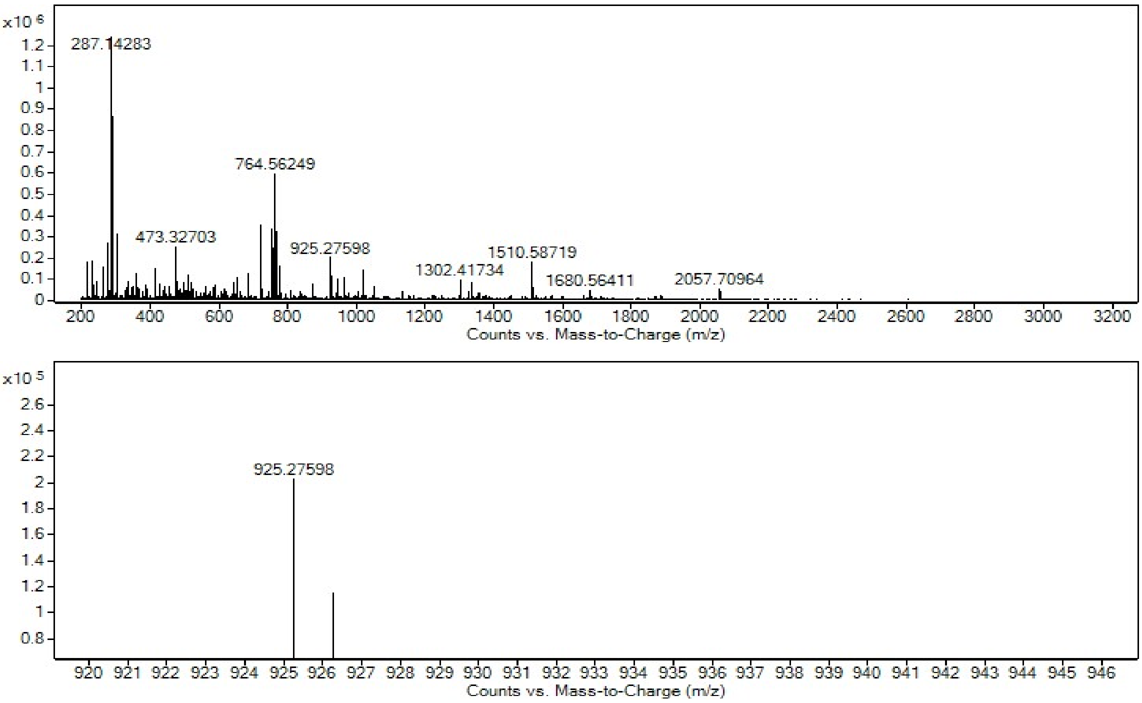

Figure S142: HRMS spectrum for **B5$_{[2+3]}$**

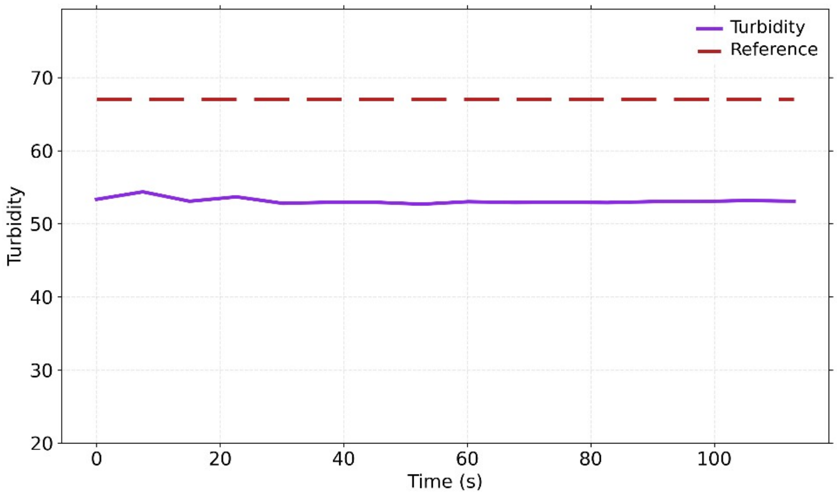

Figure S143: Turbidity vs time (s) for **B5$_{[2+3]}$**

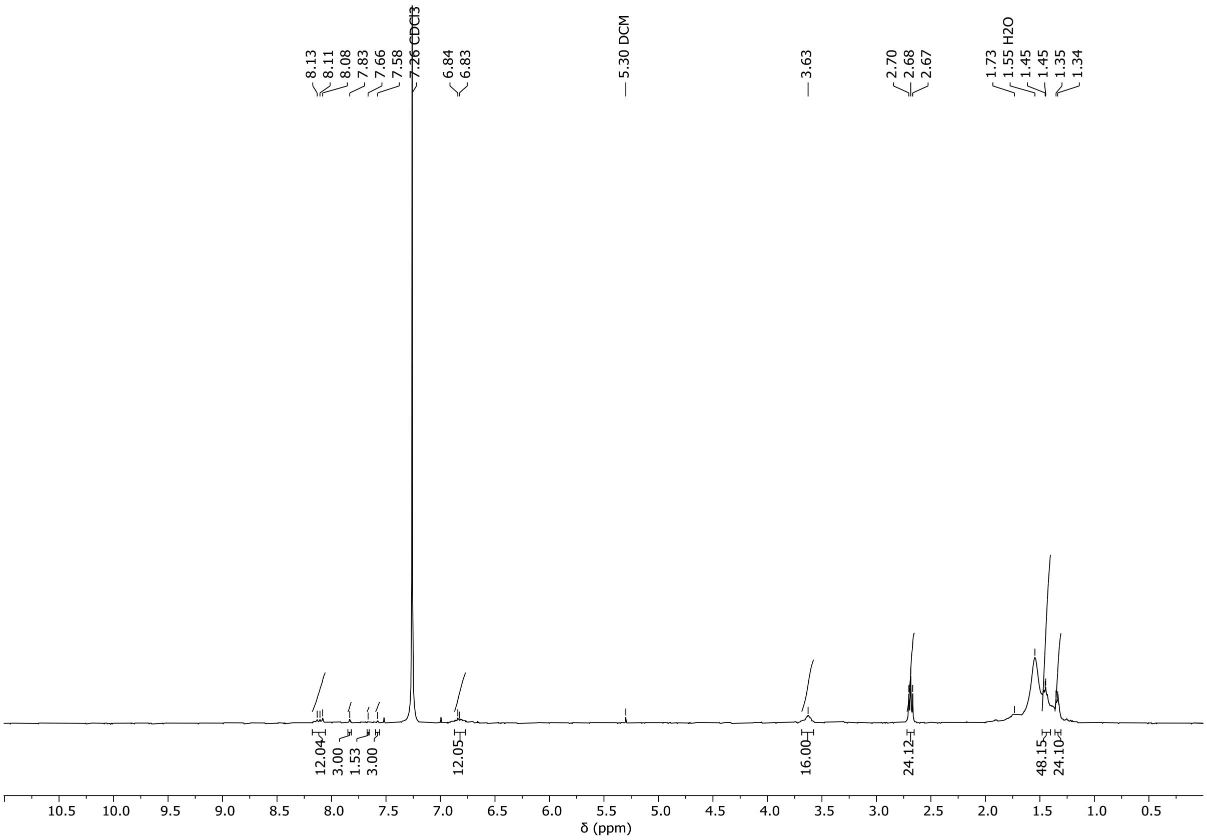

Figure S144: ^{1}H NMR ($CDCl_3$) spectrum for **P25$_{[2+3]}$**

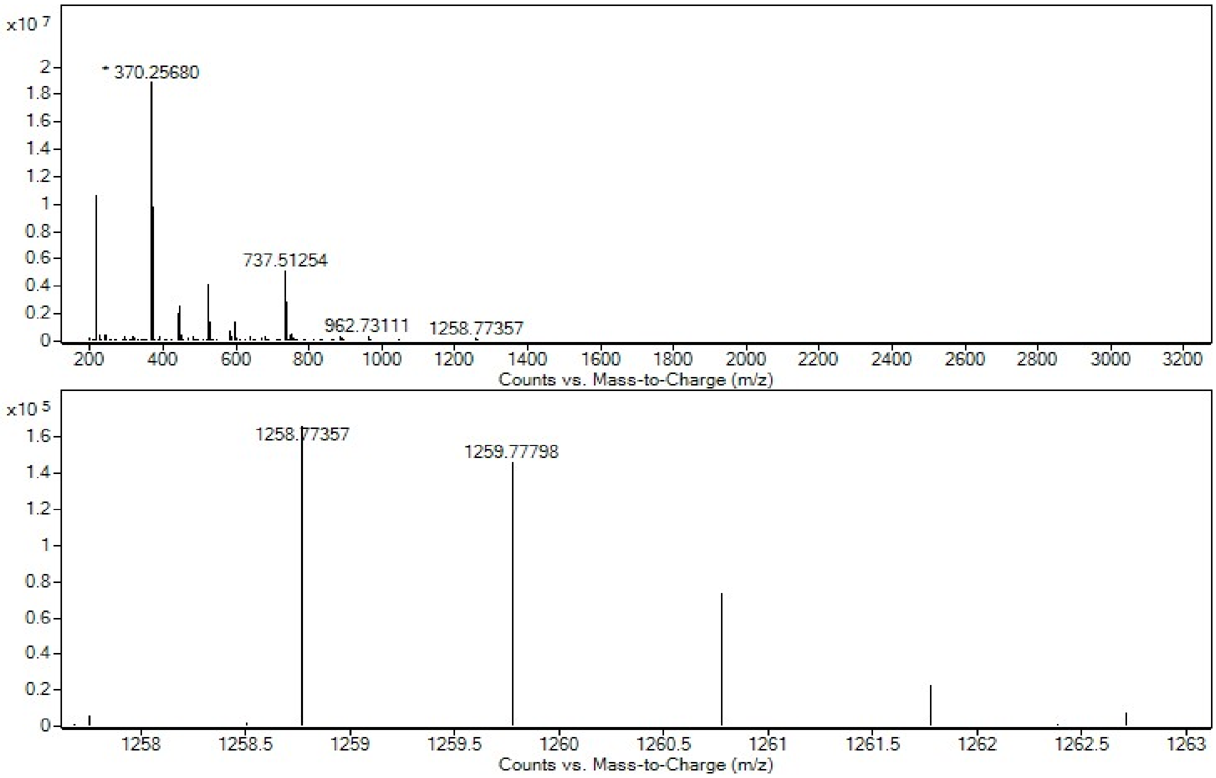

Figure S145: HRMS spectrum for **P25$_{[2+3]}$**

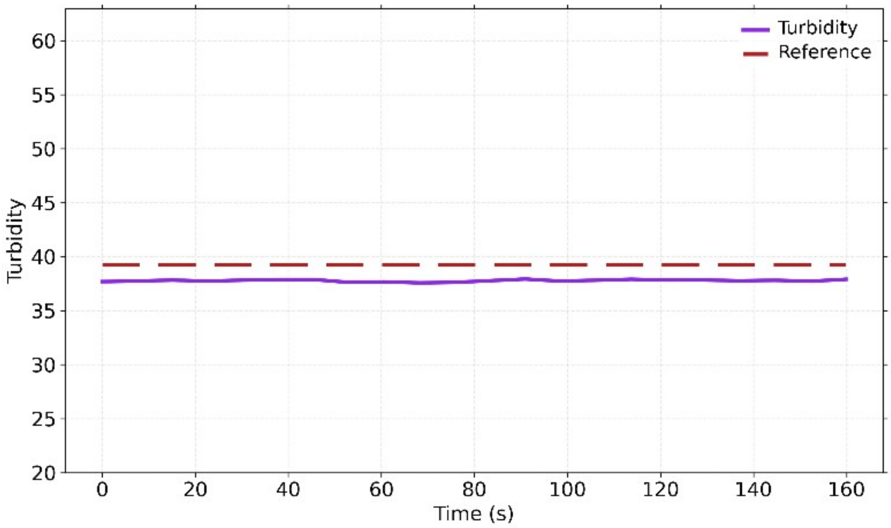

Figure S146: Turbidity vs time (s) for **P25$_{[2+3]}$**

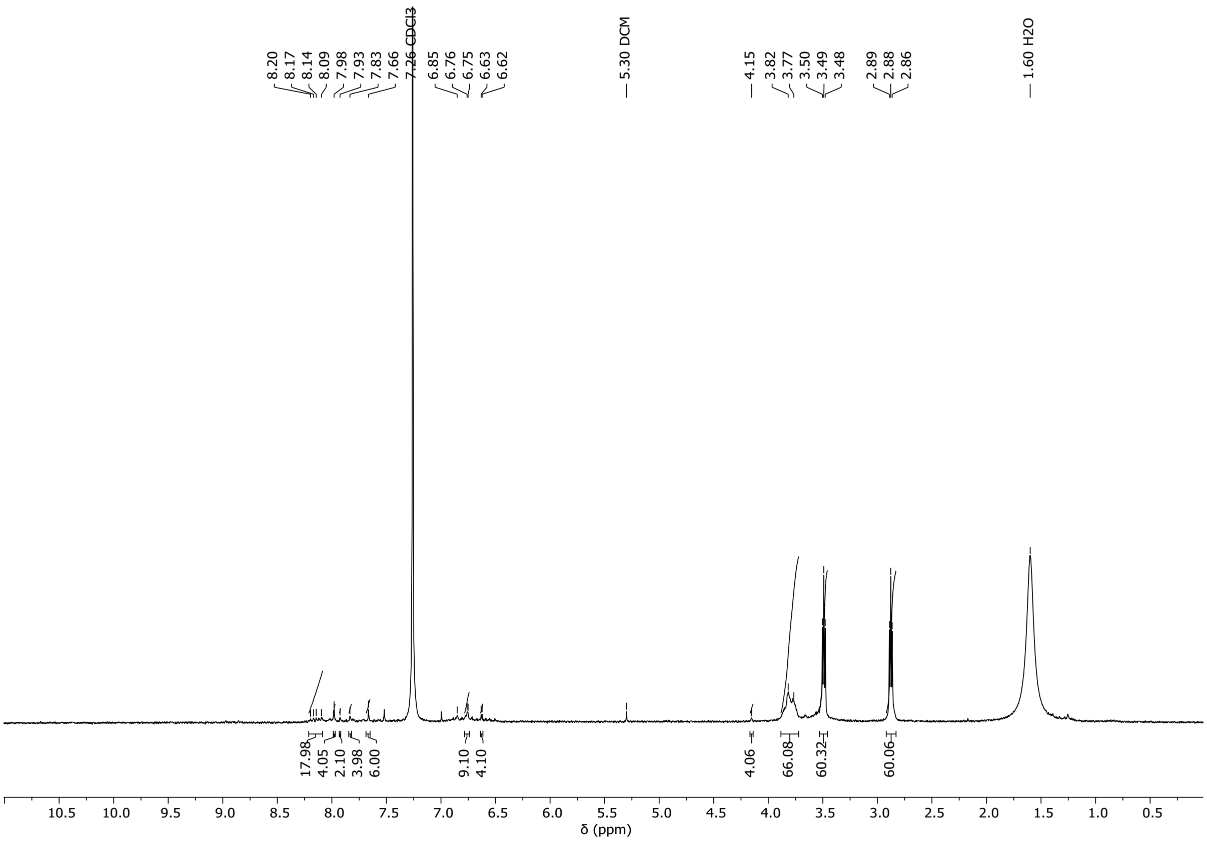

Figure S147: ^{1}H NMR ($CDCl_3$) spectrum for **$P29_{[2+3]}$**

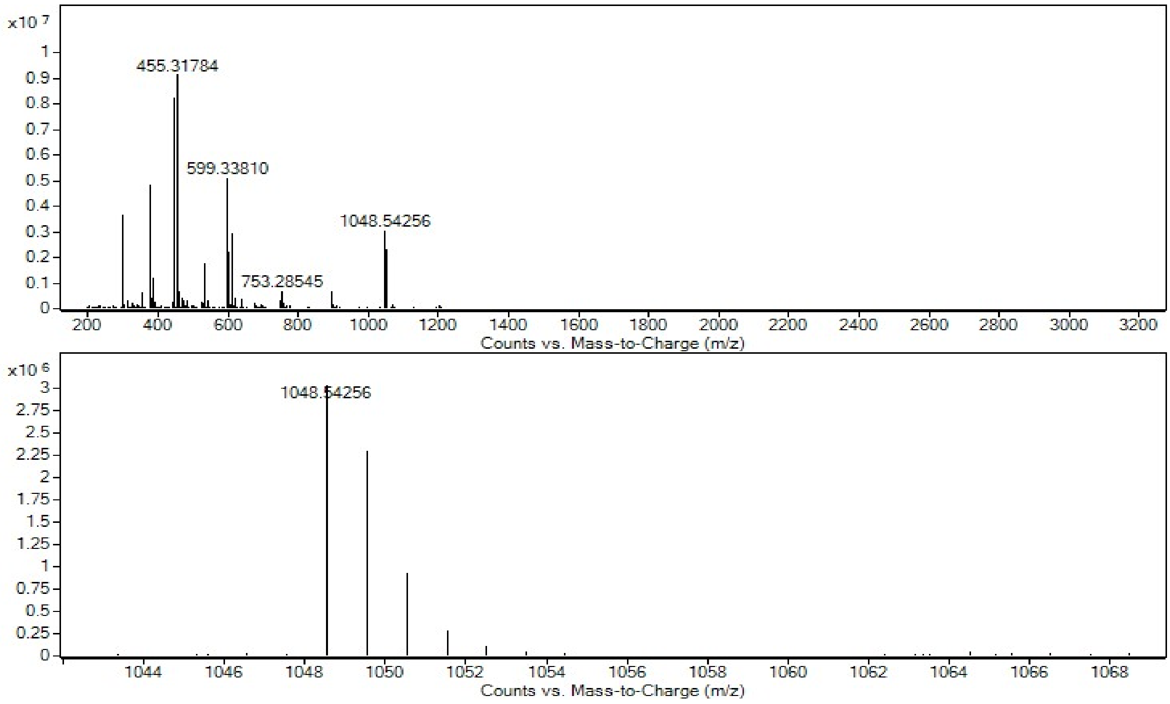

Figure S148: HRMS spectrum for **$P29_{[2+3]}$**

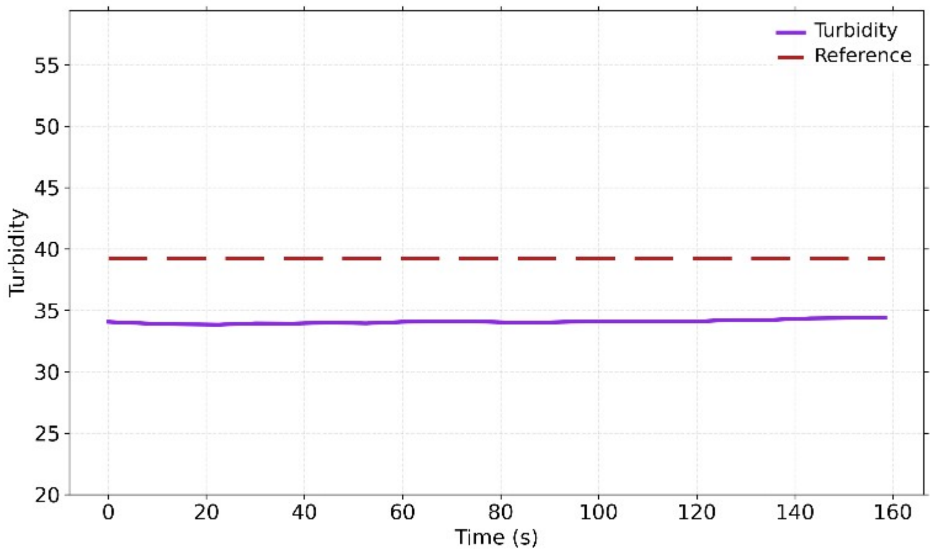

Figure S149: Turbidity vs time (s) for **P29$_{[2+3]}$**

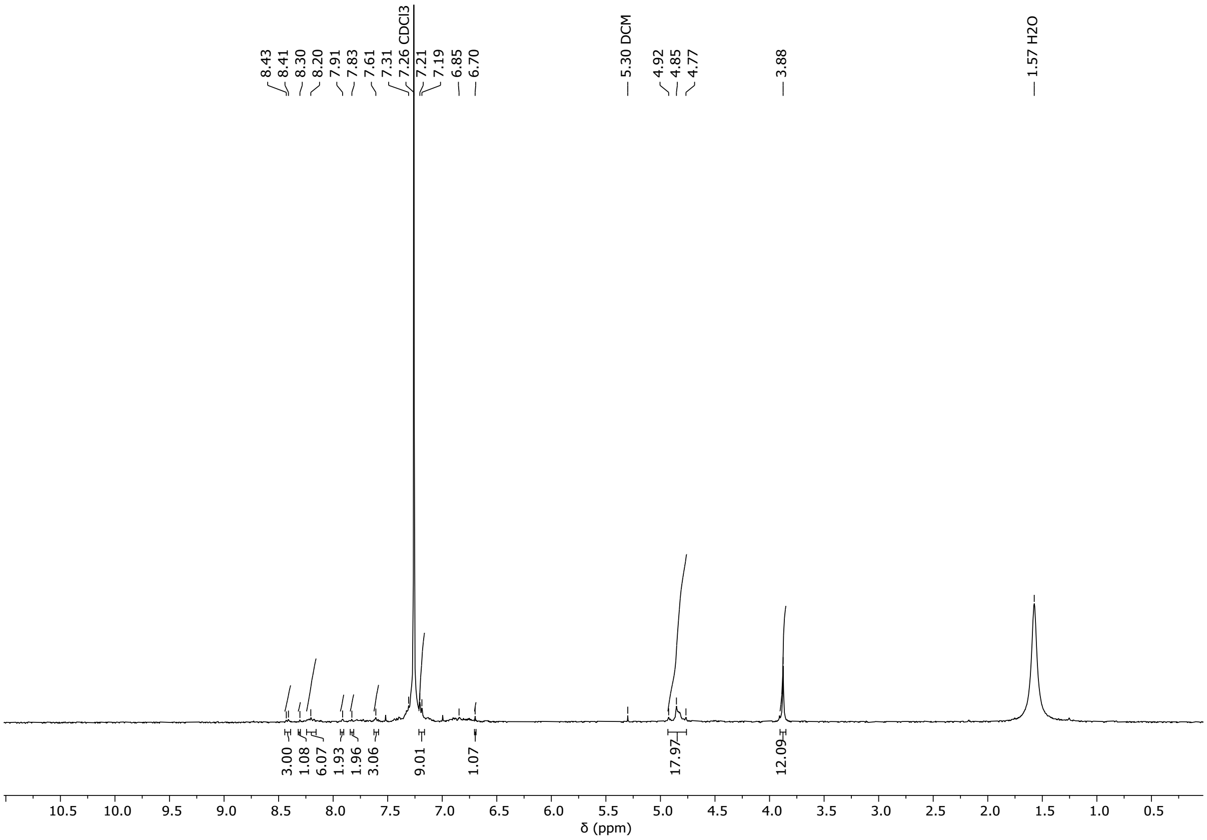

Figure S150: ^{1}H NMR ($CDCl_3$) spectrum for $\mathbf{P30_{[2+3]}}$

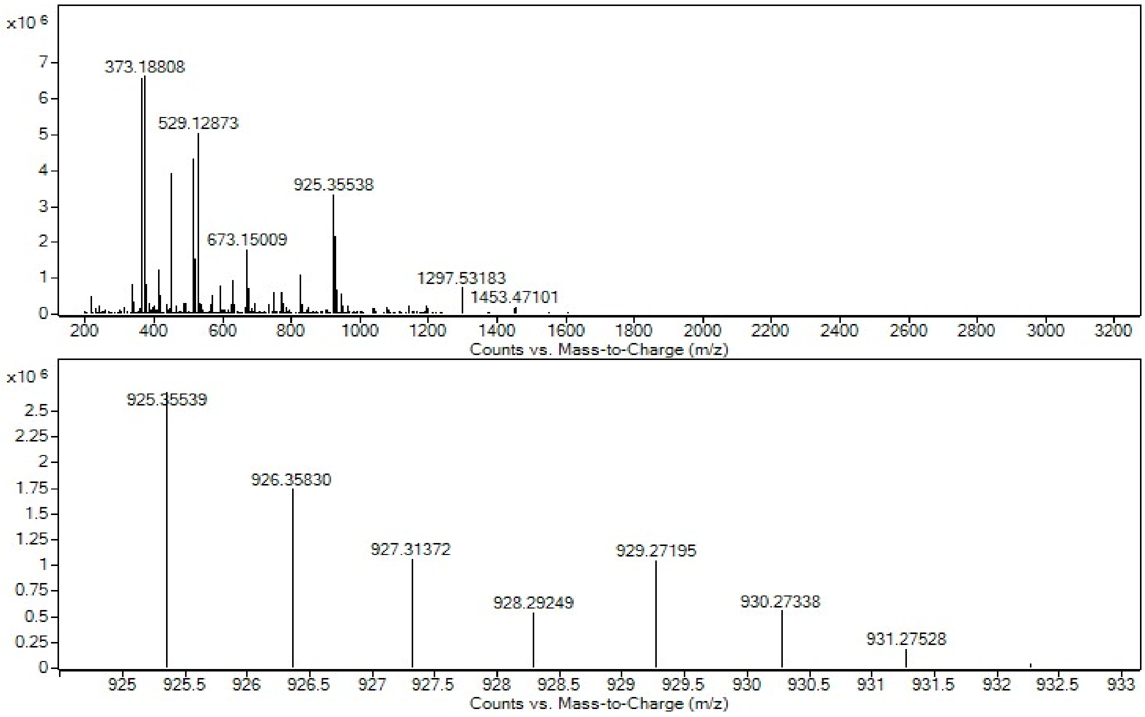

Figure S151: HRMS spectrum for $\mathbf{P30_{[2+3]}}$

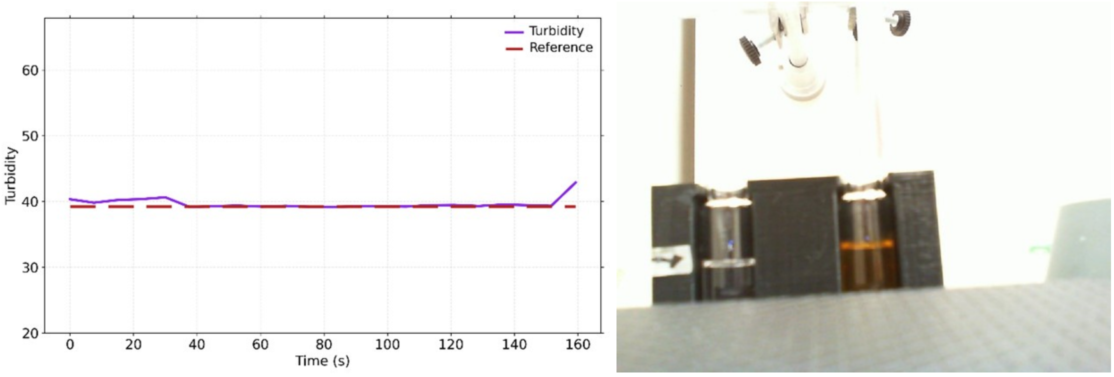

Figure S152: Turbidity vs time (s) for **P30$_{[2+3]}$.** Sample turbidity remained within the standard error for the turbidity reference throughout measurement, also confirmed by a human visual check, and therefore passed the turbidity check.

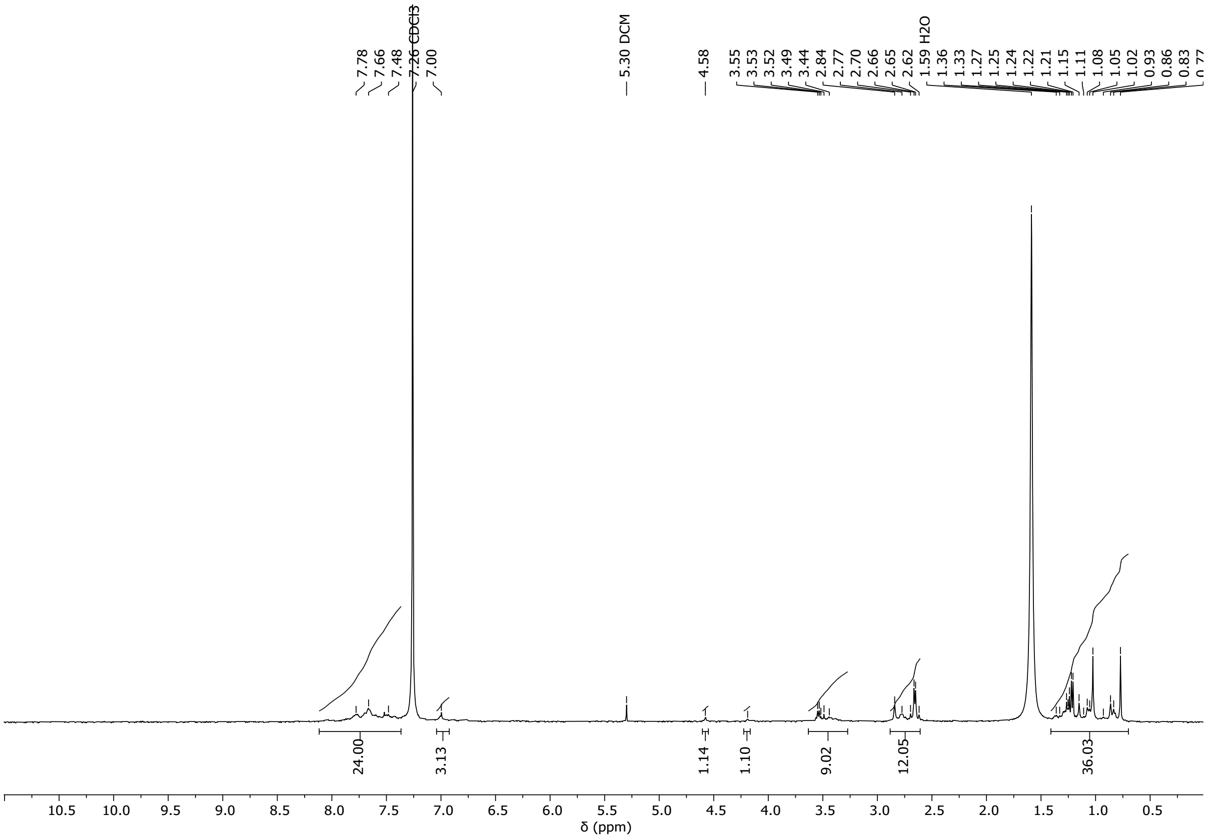

Figure S153: ^{1}H NMR ($CDCl_3$) spectrum for **$Q18_{[2+3]}$**

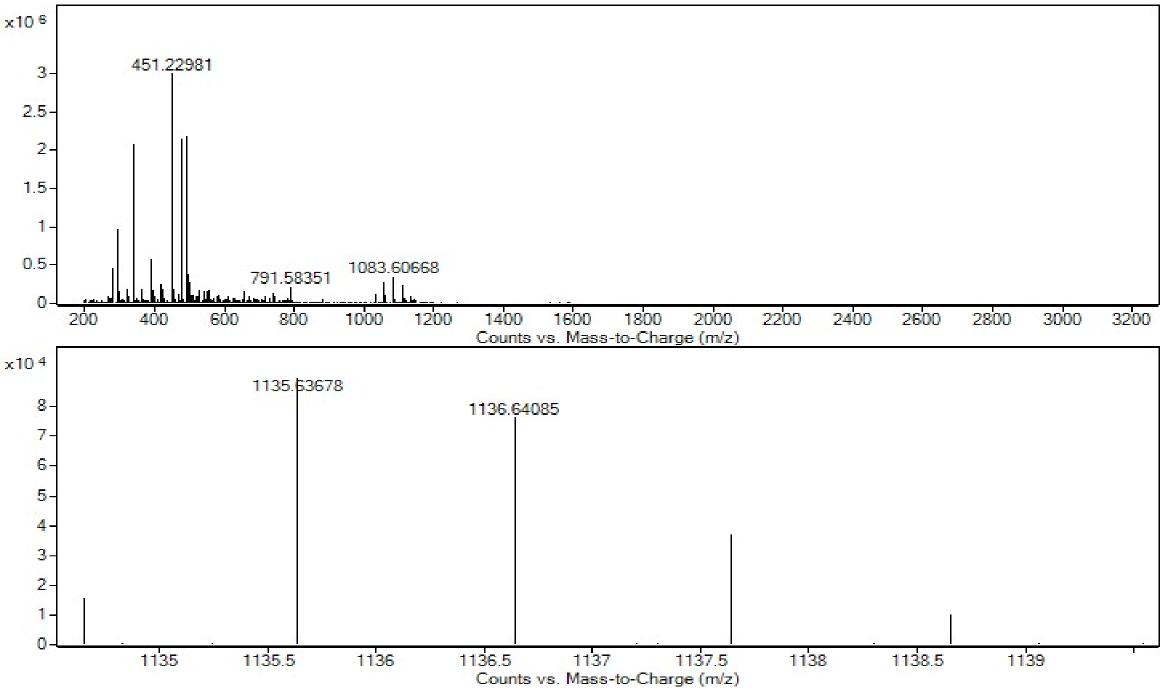

Figure S154: HRMS spectrum for **$Q18_{[2+3]}$**

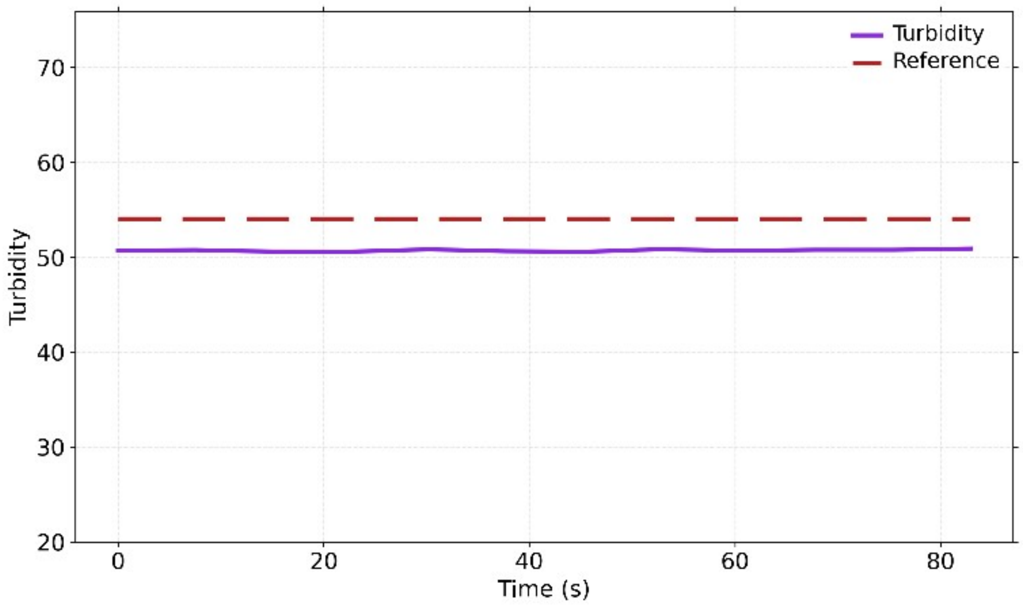

Figure S155: Turbidity vs time (s) for **Q18$_{[2+3]}$**

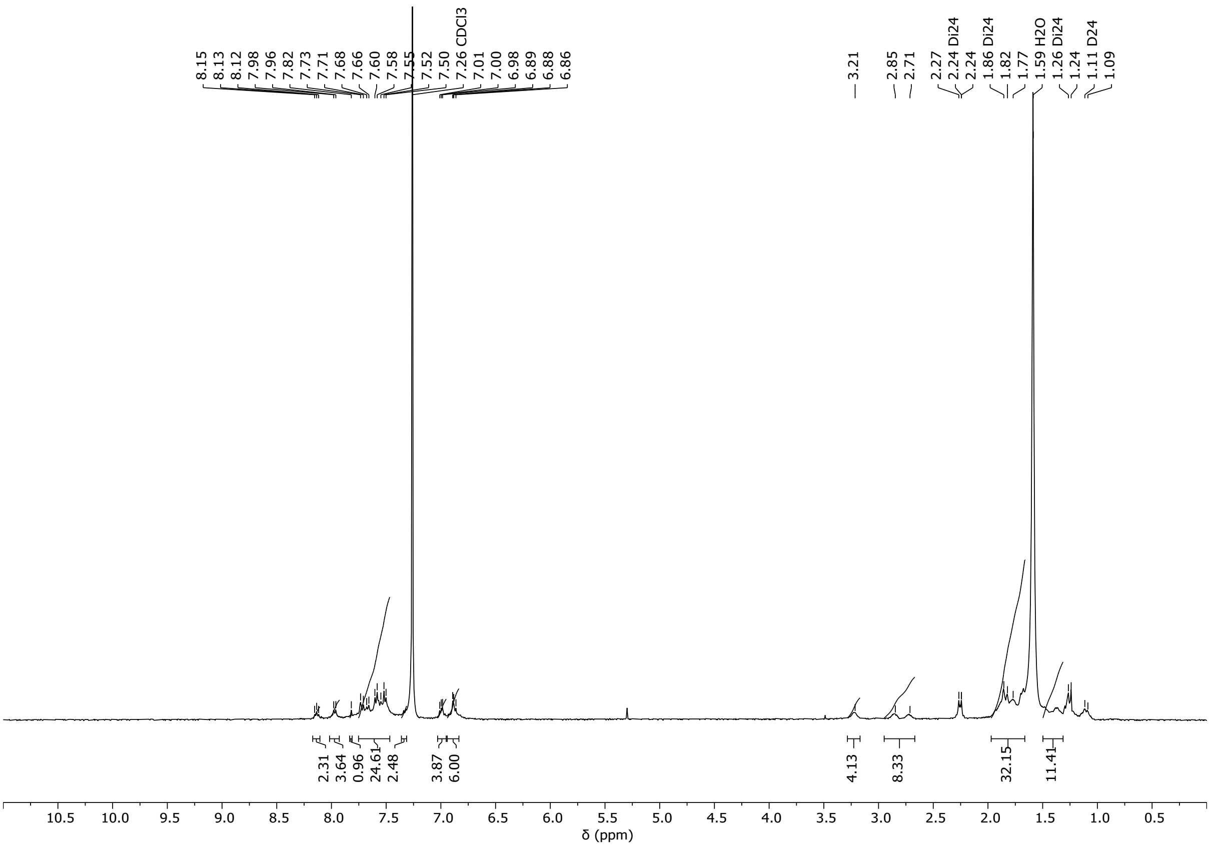

Figure S156: ^1H NMR ($CDCl_3$) spectrum for **Q24$_{[2+3]}$** - risk of competitive 1,4- vs 1,2-addition to the unsaturated aldehyde in **Q**.

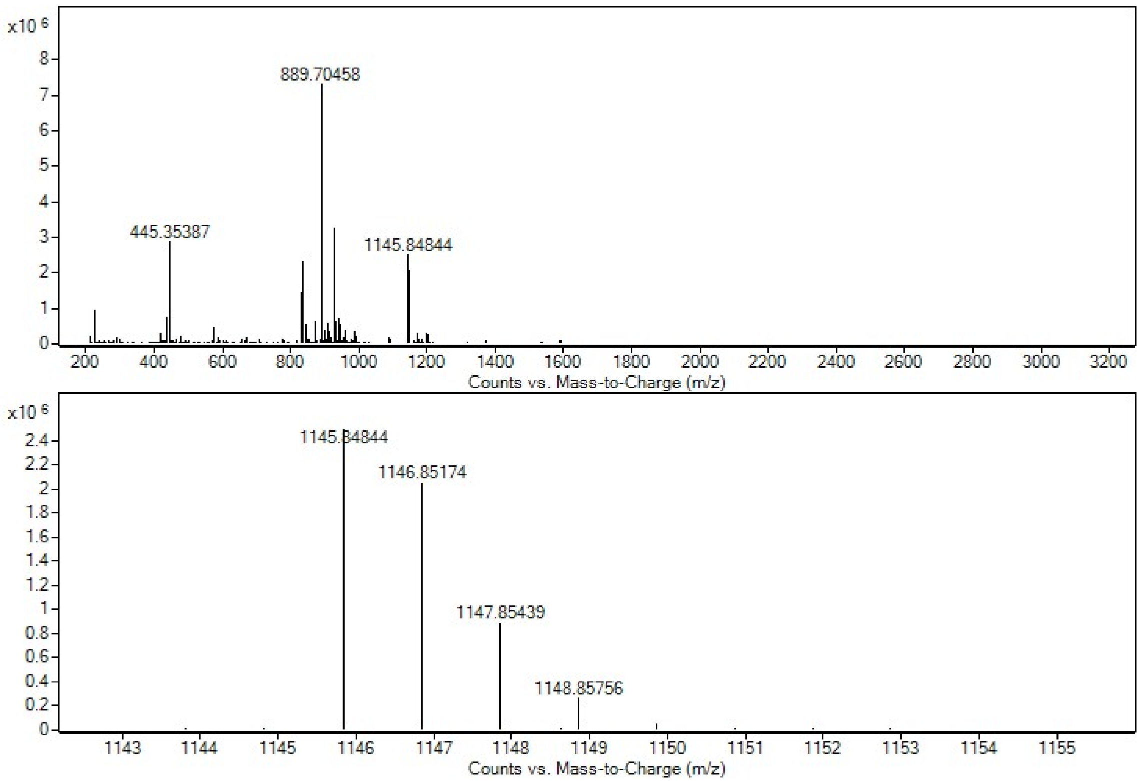

Figure S157: HRMS spectrum for **Q24$_{[2+3]}$**

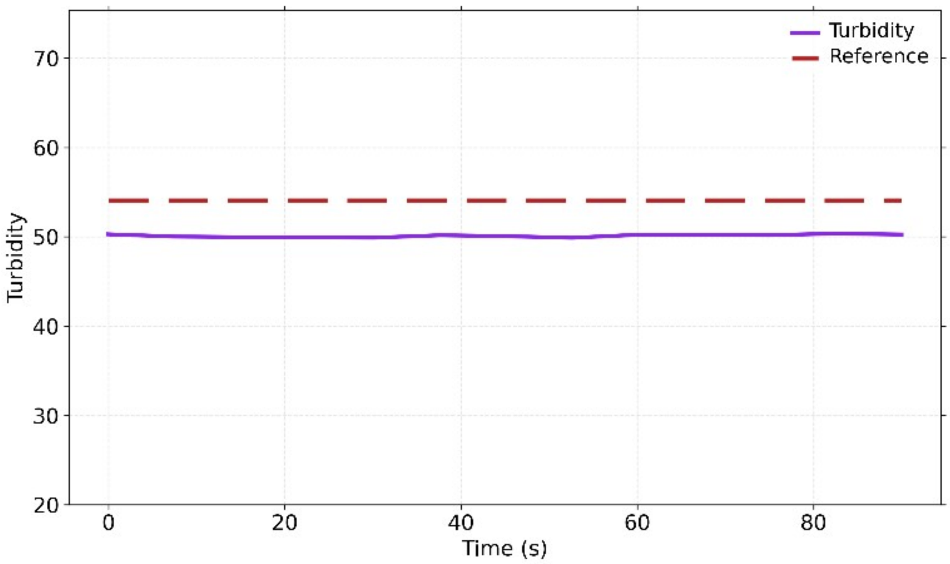

Figure S158: Turbidity vs time (s) for **Q24$_{[2+3]}$**

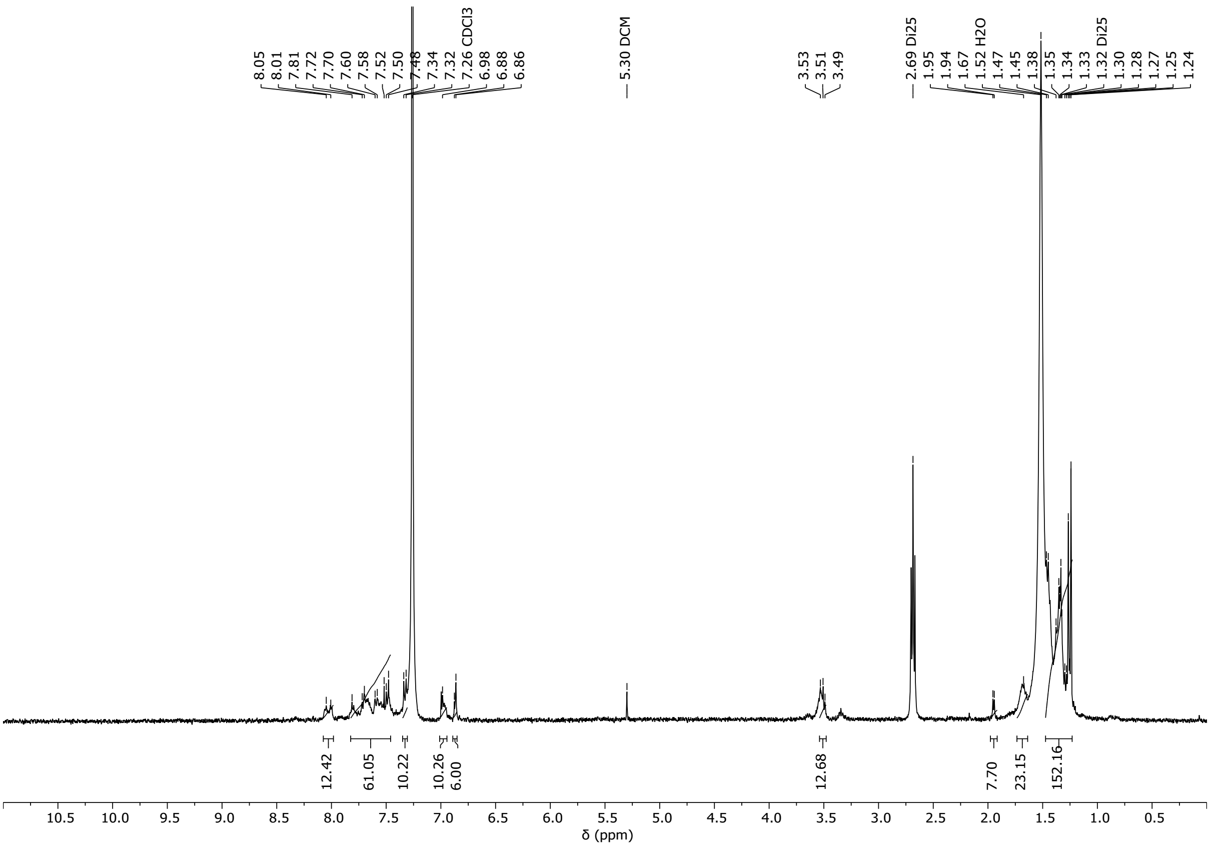

Figure S159: ^{1}H NMR ($CDCl_3$) spectrum for **Q25$_{[2+3]}$** - risk of competitive 1,4- vs 1,2-addition to the unsaturated aldehyde in **Q**.

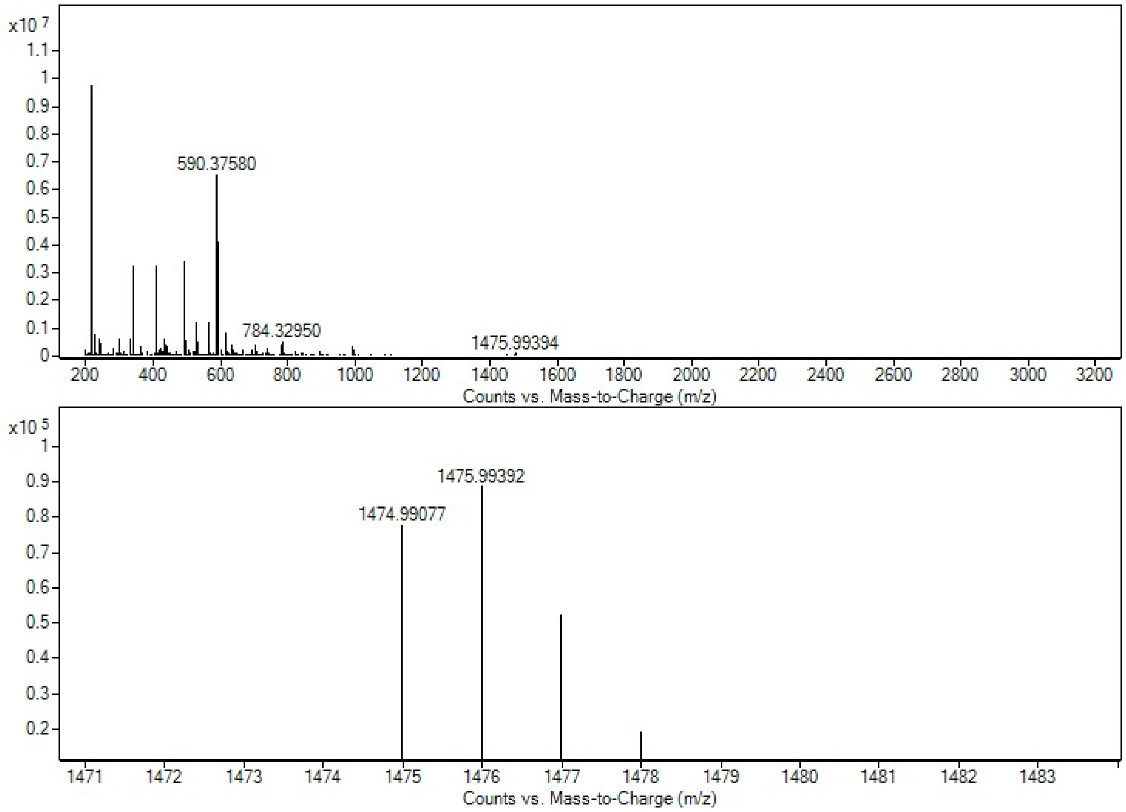

Figure S160: HRMS spectrum for **Q25$_{[2+3]}$**

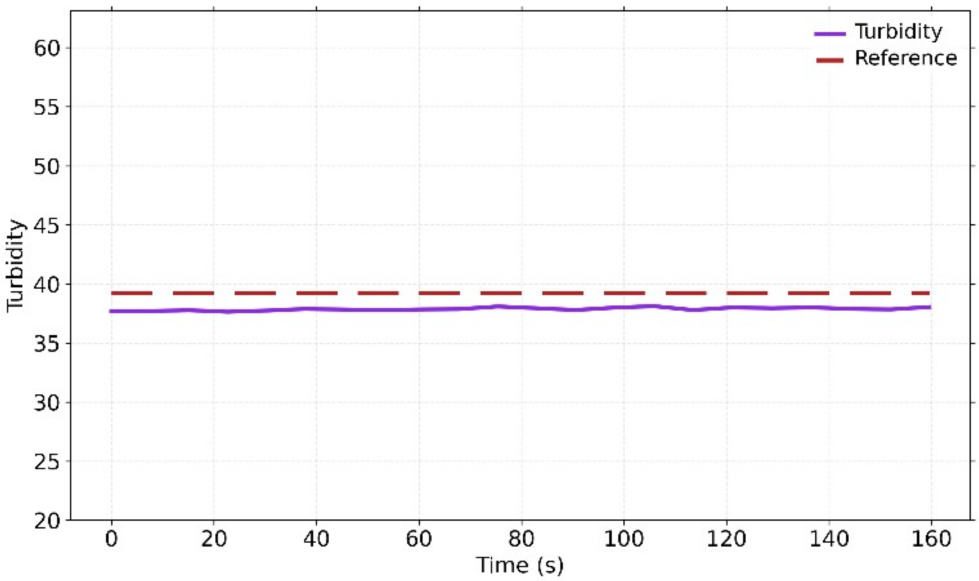

Figure S161: Turbidity vs time (s) for **Q25[2+3]**

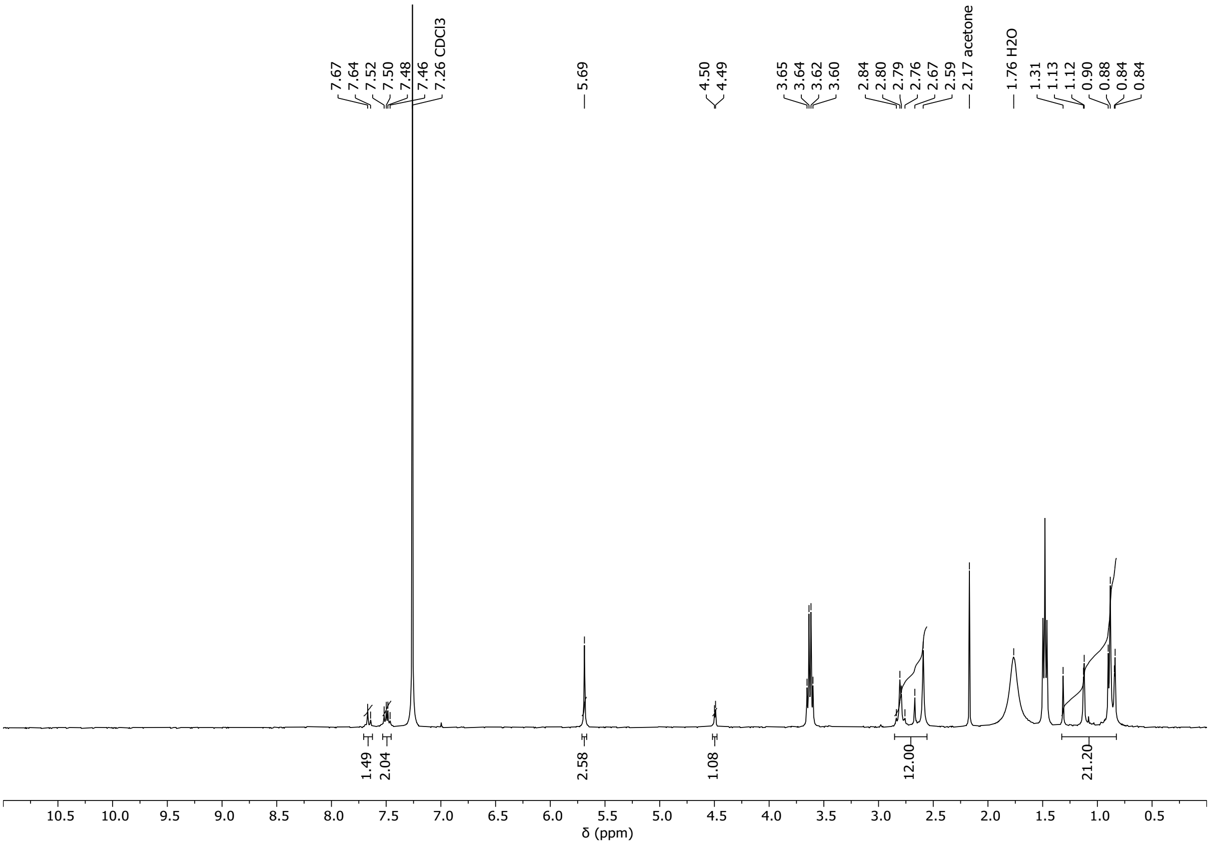

Figure S162: ^{1}H NMR ($CDCl_3$) spectrum for **$S18_{[2+3]}$** - while HRMS indicates the presence of a [2+3] species, the predominant mass ion indicates a [1+3] species, with the ^{1}H NMR spectra confirming competitive aminal formation.

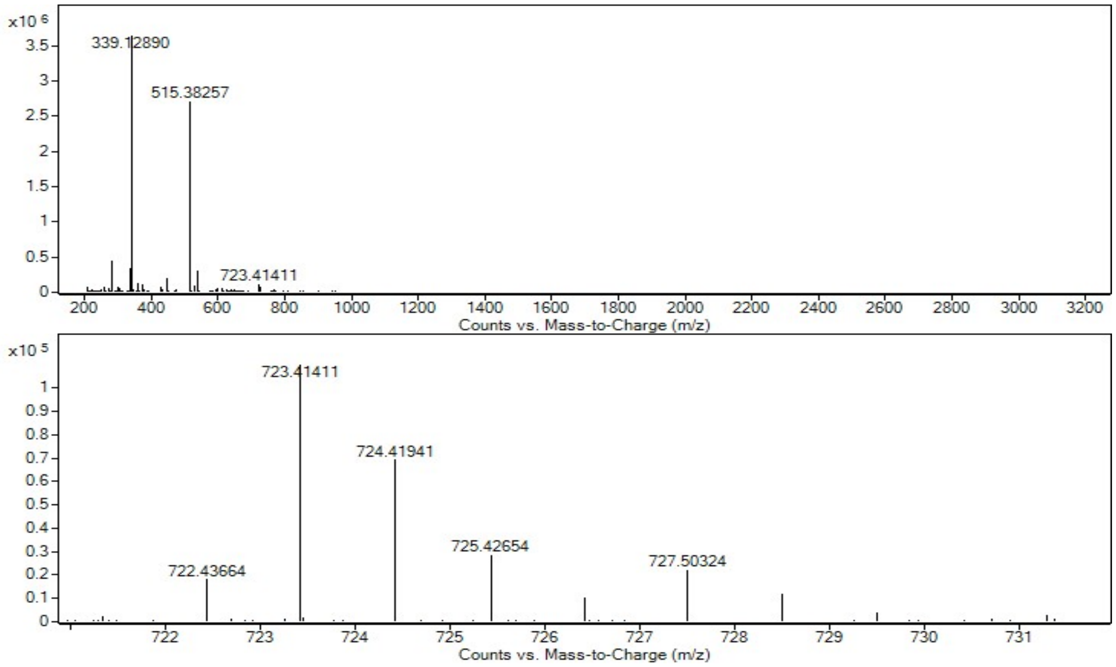

Figure S163: HRMS spectrum for **$S18_{[2+3]}$** - while HRMS indicates the presence of a [2+3] species, the predominant mass ion at 515.38257 indicates a [1+3] species.

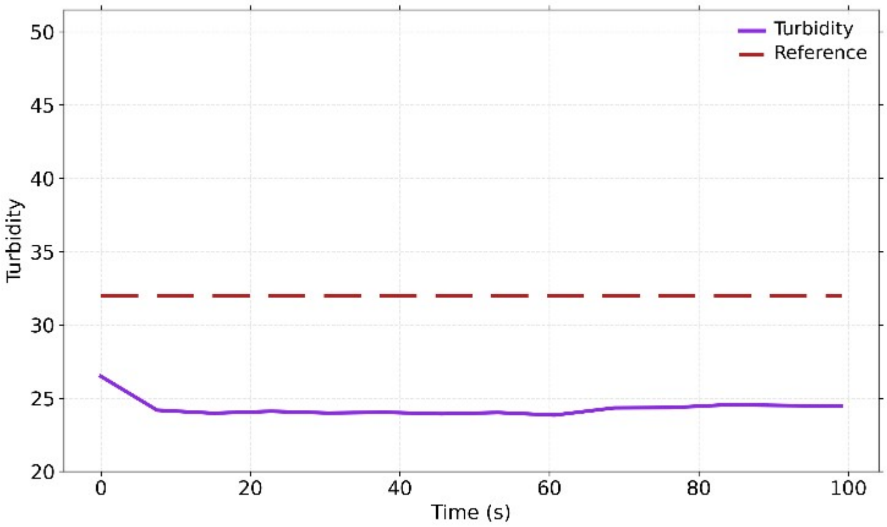

Figure S164: Turbidity vs time (s) for **S18$_{[2+3]}$**

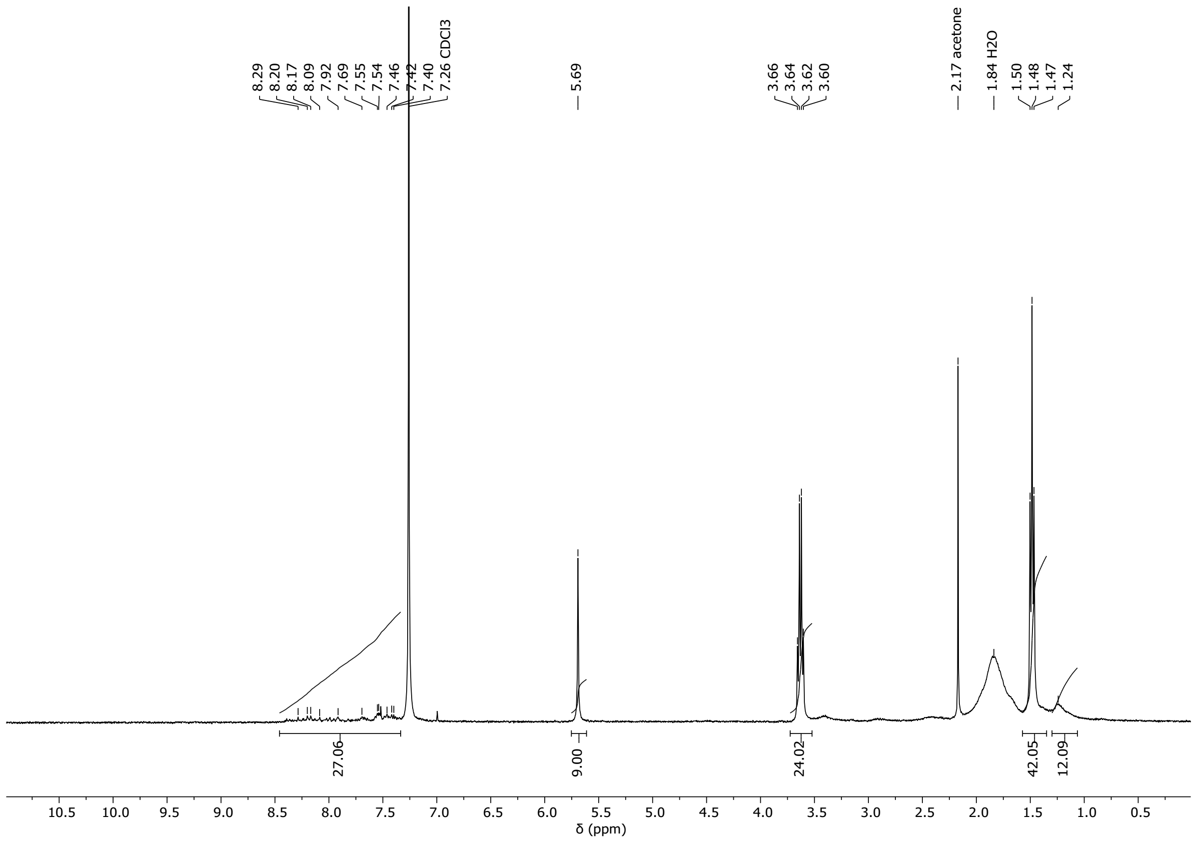

Figure S165: ^{1}H NMR ($CDCl_3$) spectrum for **S24$_{[2+3]}$**

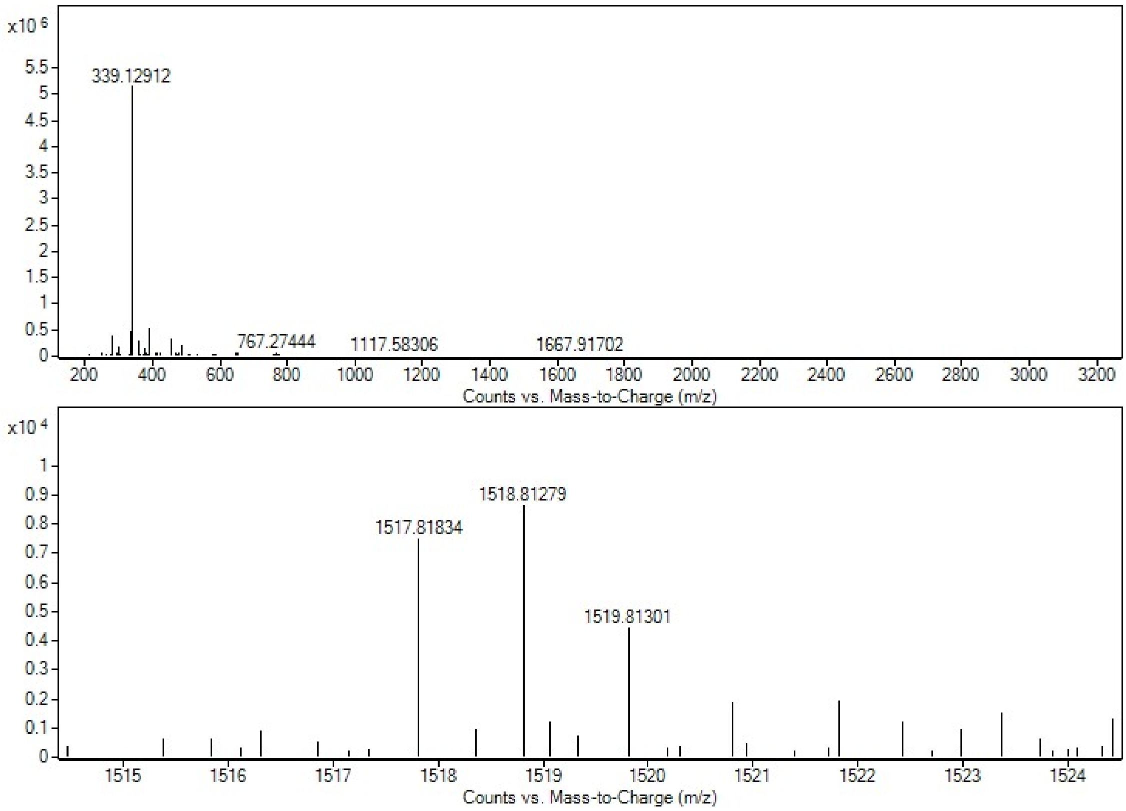

Figure S166: HRMS spectrum for **S24$_{[2+3]}$**

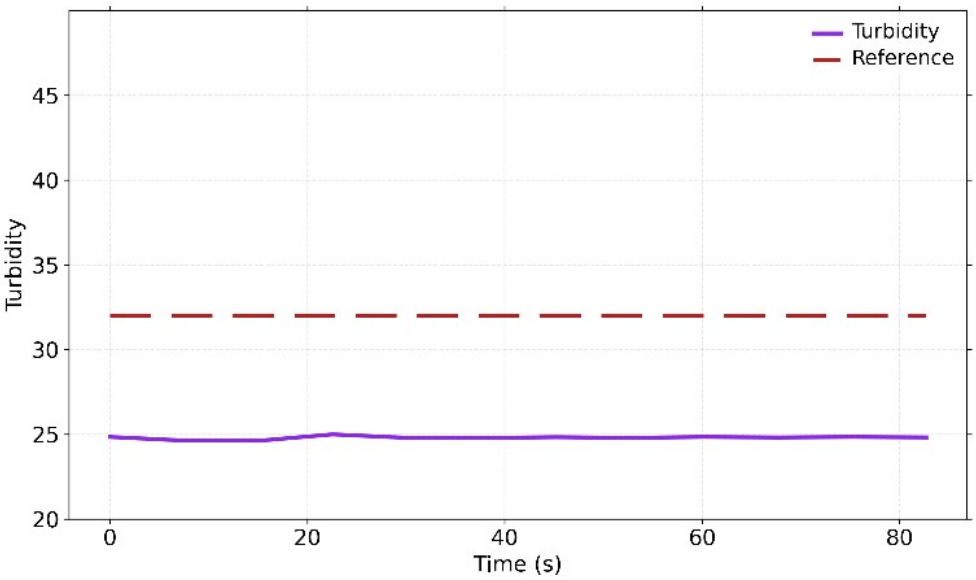

Figure S167: Turbidity vs time (s) for **S24**$_{[2+3]}$

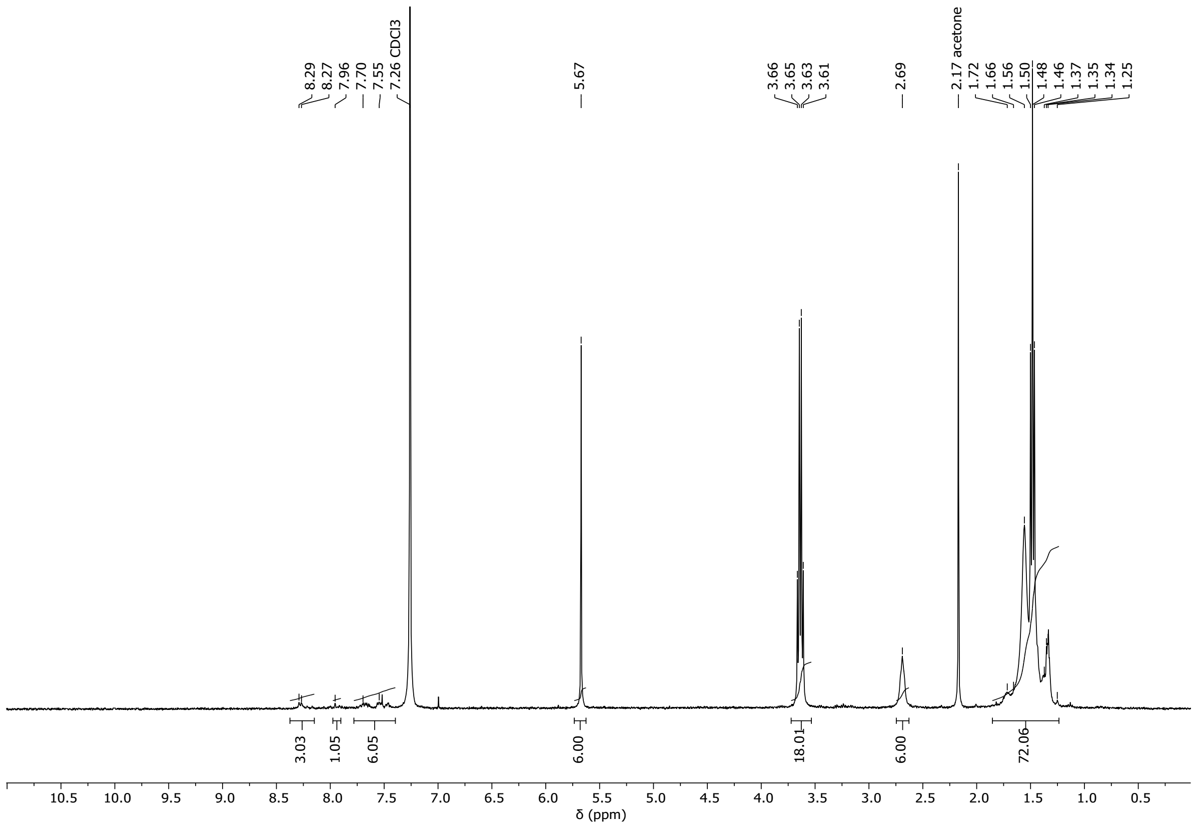

Figure S160: ^{1}H NMR ($CDCl_3$) spectrum for $\mathbf{S26_{[2+3]}}$

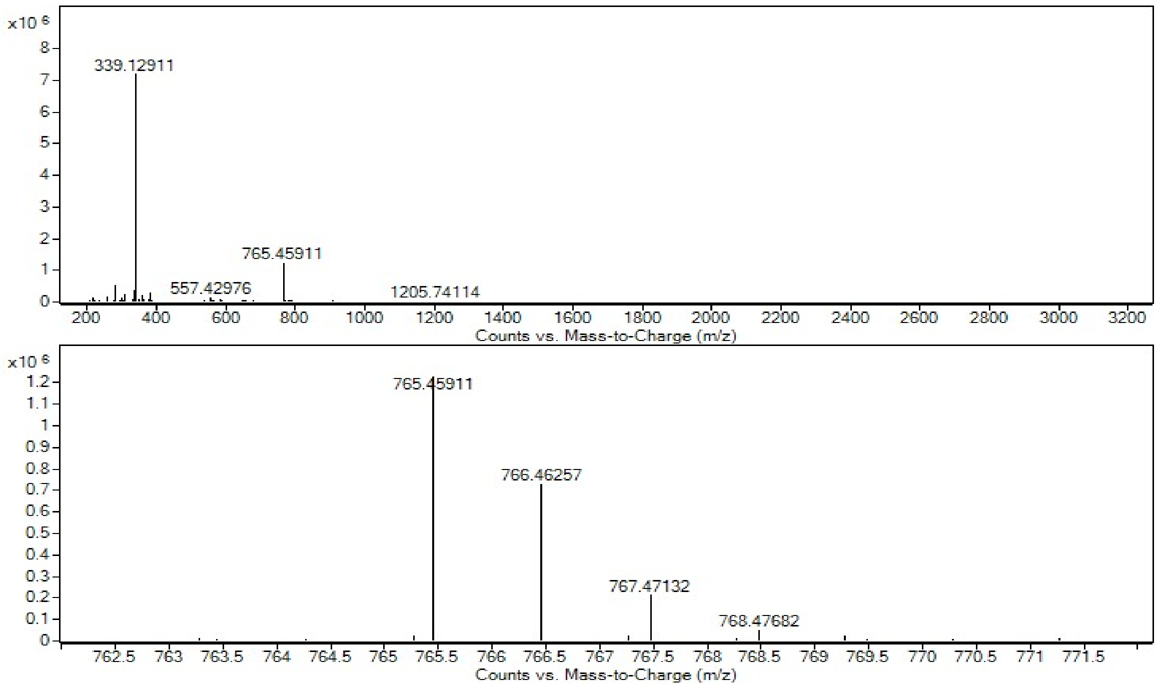

Figure S169: HRMS spectrum for $\mathbf{S26_{[2+3]}}$

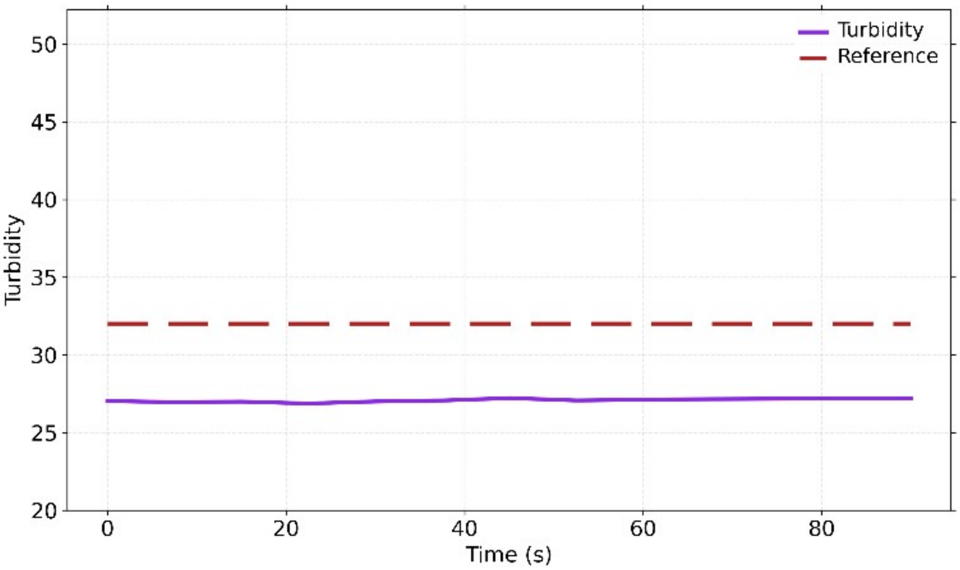

Figure S170: Turbidity vs time (s) for **S26**$_{\mathbf{[2+3]}}$

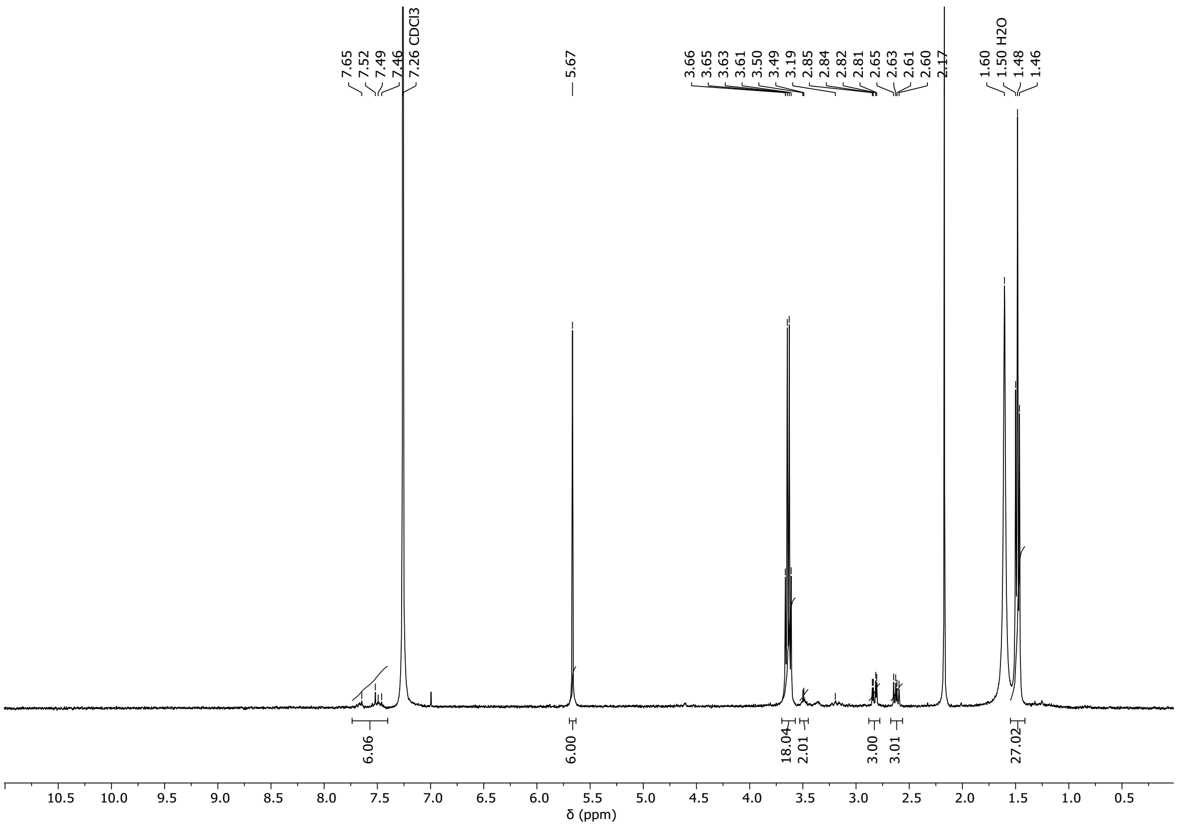

Figure S171: ^{1}H NMR ($CDCl_3$) spectrum for **$S32_{[2+3]}$**

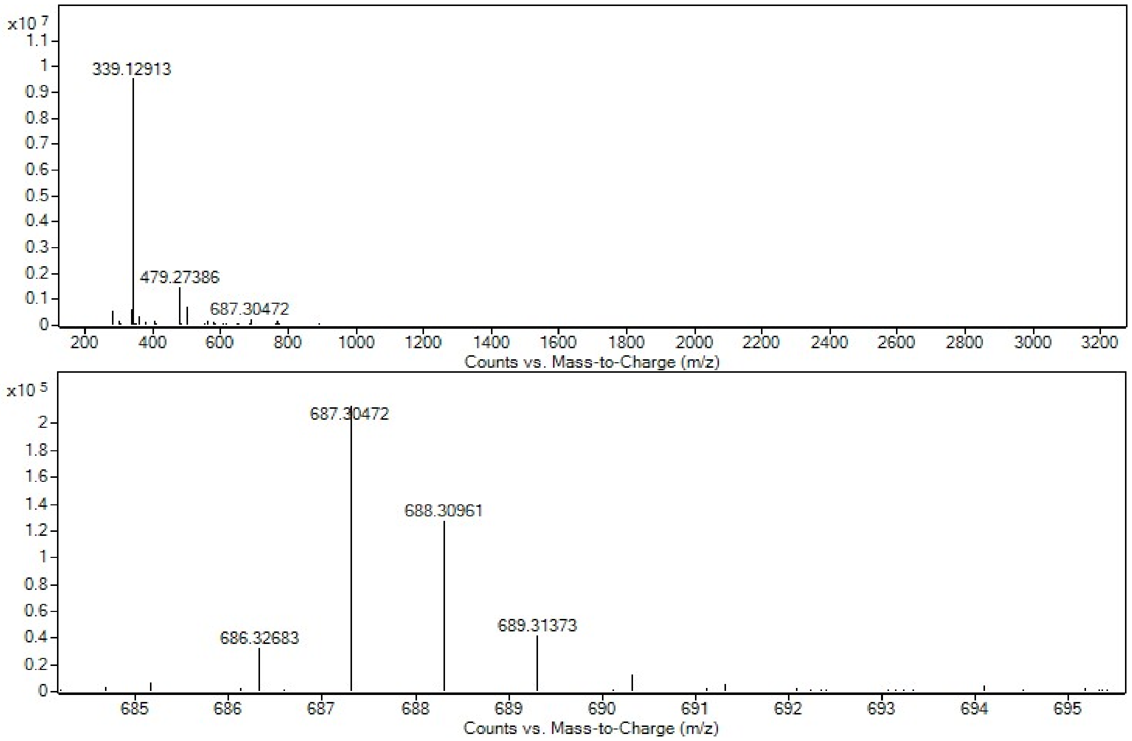

Figure S172: HRMS spectrum for **$S32_{[2+3]}$**

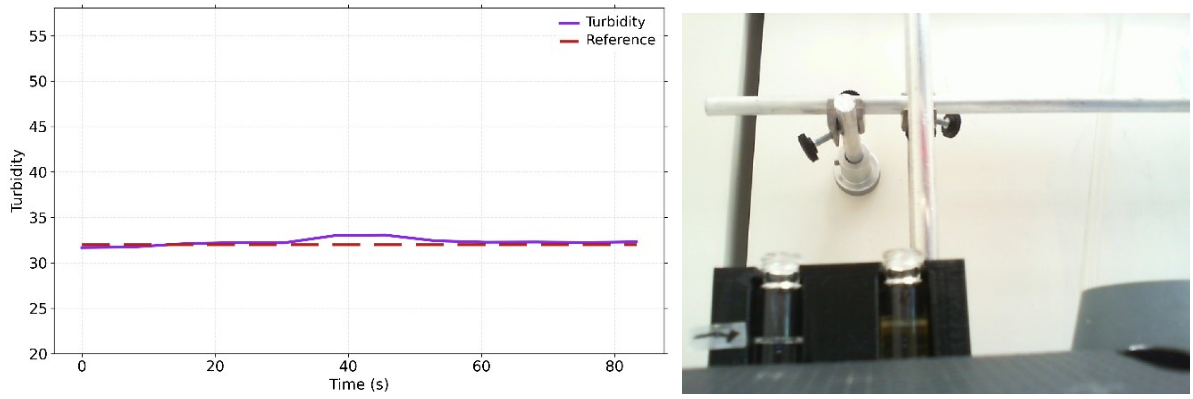

Figure S173: Turbidity vs time (s) for **S32**$_{\mathbf{[2+3]}}$. Sample turbidity remained within the standard error for the turbidity reference throughout measurement, also confirmed by a human visual check, and therefore passed the turbidity check.

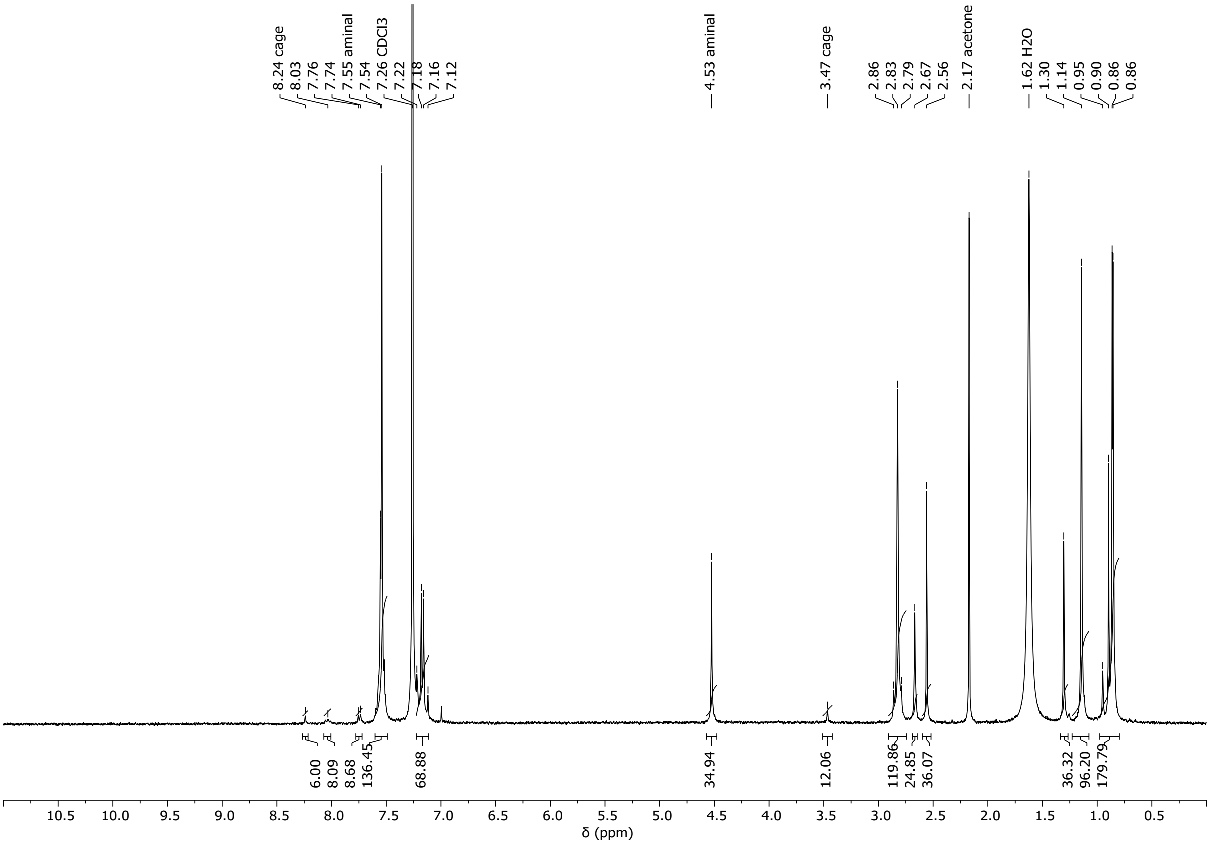

Figure S174: ^{1}H NMR ($CDCl_3$) spectrum for **U18$_{[2+3]}$** - while HRMS indicates the presence of a [2+3] species, another mass ion indicates a [1+3] species, with the ^{1}H NMR spectra confirming competitive aminal formation alongside imine cage formation.

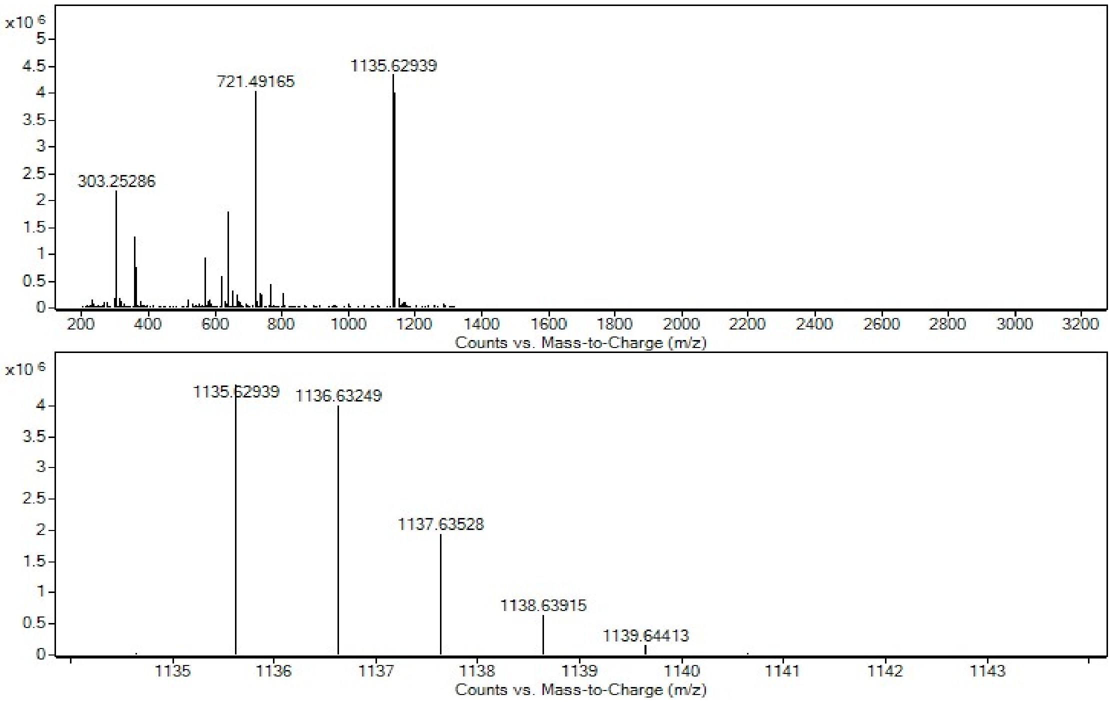

Figure S175: HRMS spectrum for **U18$_{[2+3]}$** - while HRMS indicates the presence of a [2+3] species at 1135.62939, a [1+3] species is indicated by the mass ion 721.49165.

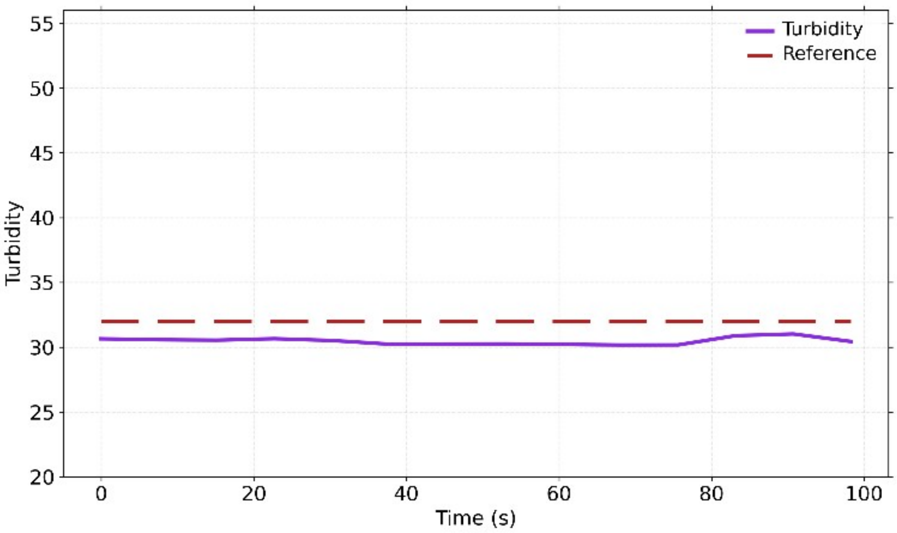

Figure S176: Turbidity vs time (s) for **U18**$_{\mathbf{[2+3]}}$

S7. Computational Modelling and Automated Analysis

S7.1 Cage Construction and Conformational Searching

First, each precursor was input using their simplified input line entry system (SMILES) code, that contains information of atom and bond types only. A lowest conformer search was conducted on each precursor using a molecular mechanics force field (MMFF), where the lowest energy conformer was then stored in a NoSQL MongoDB molecule database with their associated standardized SMILES code stored as a key.[19,20] Each precursor combination was constructed as a cage using *stk* by placing and aligning the precursors on the vertices of a predefined topology graph (**Tri^2Di^3** [2+3], **Tri^4Di^6** [4+6], **Tri^6Di^9** [6+9] and **Tri^8Di^{12}** [8+12]), resulting in the assembly of 1464 cages.[21] *In silico* 'reactions' were performed to connect functional groups assigned to the same edge, resulting in the formation of imine bonds from aldehyde and amine functional groups. The initial assembly of cages using *stk* results in structures with long unphysical bond lengths between precursors. To obtain more realistic and chemically meaningful structures with a realistic low energy conformation, a three-step conformer search procedure using the OPLS3 force field implemented in Schrödinger's MacroModel software, with *stko*.[22–24] In the first step, only the imine bonds created by *stk* were optimised, while the geometry of the atoms in each precursor placed on the vertices and edges of the cage remained fixed. In the second step, the geometries of all atoms were optimised, and the atoms in the precursors were unconstrained. Finally, molecular dynamics (MD) simulations were performed in the NVE ensemble for 100 ns, sampling 50 conformers along the trajectory using a time step of 1 fs and a temperature of 700 K. Each sampled conformer underwent further optimisation at the force field level, and the lowest energy conformation was selected for further property analysis. The force field optimisation employed a gradient convergence criterion of 0.05 kJ^{-1} A^{-1} with 2,500 as the maximum number of optimisation steps, using the Polak-Ribiere Conjugate Gradient minimisation algorithm.[25] The lowest energy conformation was then selected for subsequent property analysis. If a simulation failed, the time step was increased to 0.7 fs and then 0.5 fs if the simulation still failed. The lowest energy conformer of the cage in the chosen topology was selected and stored in a 'constructed cage' MongoDB, alongside a key of its catenated canonical SMILES of its building block precursors. A well-organised and retrievable dataset was imperative for the streamlining of this workflow. This was repeated for all precursor combinations across all four topologies, resulting in 1464 modelled cages.

All relevant Python scripts and the computational models of all the precursor combinations across all four topologies are available on the GitHub repository (https://github.com/GreenawayLab/Streamlining-Automated-Discovery-POCs/tree/main/HT_Cage_Assembly_Optimisation).

S7.2 Shape-Persistence Evaluation

The Python library *pyWindow* was selected for the analysis and structural determination of each cages molecular pore, which has previously been reported to accurately predict and reproduce POCs pore sizes across different topologies.[26] *pyWindow* encapsulates the modelled cage in a sphere of points with vectors pointing into the centre of mass of the cage. The pore cavity and windows are determined as regions of space where the vectors were not intercepted by atoms before reaching the centre of mass. Pore volume as an extension was calculated as the maximum sphere size on those vector points

that did not have any overlap with the van der Waals radii of the atoms of the cage. The number of windows and the average window diameter was computationally predicted to indicate how a guest molecule may diffuse into the cage and to indicate its structural stability. If a cage possessed a low number of windows, or a broad set of window sizes, it may be asymmetric. A lower symmetry may also be observed in cages formed from unsymmetrical precursor building blocks. A cage of lower symmetry indicates lower structural stability, that would likely result in an unstable cage that may collapse, reducing its pore volume, and therefore reducing its ability to host a guest.

Investigation into all 366 precursor combinations across the four topologies (total 1464 molecules) sought the identification of shape persistent cages with the correct number of windows and a cavity greater than 1 Å, which would be sufficient to host hypothetical spherical guests of significant size. If it was identified as collapsed it was disregarded from further calculation. The cavity size minimum was reduced to 0.1 Å for the **Tri^2Di^3** [2+3] topology.

S8. References

1 OT-2 Liquid Handler | Opentrons Lab Automation from $10,000 | Opentrons, https://opentrons.com/products/robots/ot-2/, (accessed 24 October 2023).

2 EquaVAP® 48-Well Evaporator (23048), https://www.analytical-sales.com/product/equavap-48-well-evaporator-23048/, (accessed 1 June 2023).

3 Sample Handling | Lab Automation | Bruker, https://www.bruker.com/en/products-and-solutions/mr/nmr/nmr-automation/samplejet.html?gclid=CjwKCAjwr56IBhAvEiwA1fuqGu1RBONTvHdIOQYsH4N6rJrS__5NcF7qioM0sY_EO4iX-u-ydVJI8RoCkbcQAvD_BwE, (accessed 2 August 2021).

4 Hein Group / heinsight_liquid_level_H1 · GitLab, https://gitlab.com/heingroup/heinsight_liquid_level_h1, (accessed 20 October 2023).

5 P. Shiri, V. Lai, T. Zepel, D. Griffin, J. Reifman, S. Clark, S. Grunert, L. P. E. Yunker, S. Steiner, H. Situ, F. Yang, P. L. Prieto and J. E. Hein, *iScience*, 2021, **24**, 102176.

6 R. L. Greenaway, V. Santolini, M. J. Bennison, B. M. Alston, C. J. Pugh, M. A. Little, M. Miklitz, E. G. B. Eden-Rump, R. Clowes, A. Shakil, H. J. Cuthbertson, H. Armstrong, M. E. Briggs, K. E. Jelfs and A. I. Cooper, *Nat. Commun.*, 2018, **9**, 2849.

7 L. Grunenberg, G. Savasci, M. W. Terban, V. Duppel, I. Moudrakovski, M. Etter, R. E. Dinnebier, C. Ochsenfeld and B. V. Lotsch, *J. Am. Chem. Soc.*, 2021, **143**, 3430–3438.

8 A. Woiczechowski-Pop, I. L. Dobra, G. D. Roiban, A. Terec and I. Grosu, *Synth. Commun.*, 2012, **42**, 3579–3588.

9 M. J. Plater and M. Praveen, *Tetrahedron Lett.*, 1997, **38**, 1081–1082.

10 S. Kotha, S. Todeti, M. B. Gopal and A. Datta, *ACS Omega*, 2017, **2**, 6291–6297.

11 K. Nowosinski, L. K. S. von Krbek, N. L. Traulsen and C. A. Schalley, *Org. Lett.*, 2015, **17**, 5076–5079.

12 E. Berardo, R. L. Greenaway, L. Turcani, B. M. Alston, M. J. Bennison, M. Miklitz, R. Clowes, M. E. Briggs, A. I. Cooper and K. E. Jelfs, *Nanoscale*, 2018, **10**, 22381–22388.

13 E. Díez-Barra, J. C. García-Martínez, S. Merino, R. del Rey, J. Rodríguez-López, P. Sánchez-Verdú and J. Tejeda, *J. Org. Chem.*, 2001, **66**, 5664–5670.

14 J. R. Holst, A. Trewin and A. I. Cooper, *Nat. Chem.*, 2010, **2**, 915–920.

15 J. J. Helmus and C. P. Jaroniec, *J. Biomol. NMR*, 2013, **55**, 355–367.

16 D. Kessner, M. Chambers, R. Burke, D. Agus and P. Mallick, *Bioinformatics*, 2008, **24**, 2534–2536.

17 R. Schmid, S. Heuckeroth, A. Korf, A. Smirnov, O. Myers, T. S. Dyrlund, R. Bushuiev, K. J. Murray, N. Hoffmann, M. Lu, A. Sarvepalli, Z. Zhang, M. Fleischauer, K. Dührkop, M. Wesner, S. J. Hoogstra, E. Rudt, O. Mokshyna, C. Brungs, K. Ponomarov, L. Mutabdžija, T. Damiani, C. J. Pudney, M. Earll, P. O. Helmer, T. R. Fallon, T. Schulze, A. Rivas-Ubach, A. Bilbao, H. Richter, L.-F. Nothias, M. Wang, M. Orešič, J.-K. Weng, S. Böcker, A. Jeibmann, H. Hayen, U. Karst, P. C. Dorrestein, D. Petras, X. Du and T. Pluskal, *Nat. Biotechnol.*, 2023, **41**, 447–449.

18 pyOpenMS: A Python-based interface to the OpenMS mass-spectrometry algorithm library - Röst - 2014 - PROTEOMICS - Wiley Online Library, https://analyticalsciencejournals.onlinelibrary.wiley.com/doi/full/10.1002/pmic.201300246, (accessed 25 July 2023).

19 P. Tosco, N. Stiefl and G. Landrum, *J. Cheminformatics*, 2014, **6**, 37.

20 S. Bradshaw, E. Brazil and K. Chodorow, *MongoDB: The Definitive Guide: Powerful and Scalable Data Storage*, O’Reilly Media, Inc., 2019.

21 L. Turcani, E. Berardo and K. E. Jelfs, *J. Comput. Chem.*, 2018, **39**, 1931–1942.

22 E. Harder, W. Damm, J. Maple, C. Wu, M. Reboul, J. Y. Xiang, L. Wang, D. Lupyan, M. K. Dahlgren, J. L. Knight, J. W. Kaus, D. S. Cerutti, G. Krilov, W. L. Jorgensen, R. Abel and R. A. Friesner, *J. Chem. Theory Comput.*, 2016, **12**, 281–296.

23 M. Schrödinger, Schrödinger Release 2023-3 Schrödinger, LLC, New York, NY 2023.

24 JelfsMaterialsGroup/stko Computational Supramolecular Materials Discovery 2023.

25 E. Polak and G. Ribiere, *Rev. Fr. Inform. Rech. Opérationnelle Sér. Rouge*, 1969, **3**, 35–43.
26 M. Miklitz and K. E. Jelfs, *J. Chem. Inf. Model.*, 2018, **58**, 2387–2391.
